# Supplementary figures and images for: Exposure image correction of electrical equipment nameplate based on the LMPEC algorithm (part 2 of 2)
Source: PLoS One. 2024 Jun 27;19(6):e0300792. doi: 10.1371/journal.pone.0300792 (PMC11210806; doi:10.1371/journal.pone.0300792)

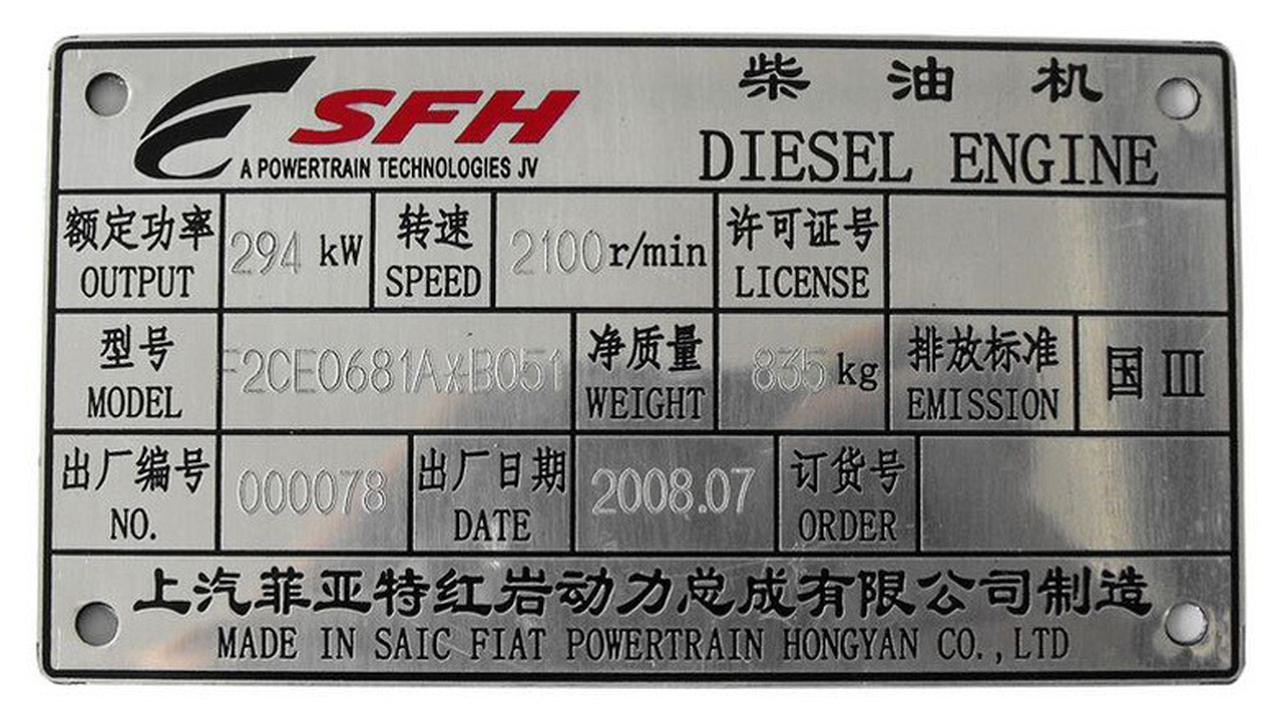

Supplement: S1 Dataset — (ZIP) [file pone.0300792.s001.zip › minimal data set/gt_img_0021_0.jpg]

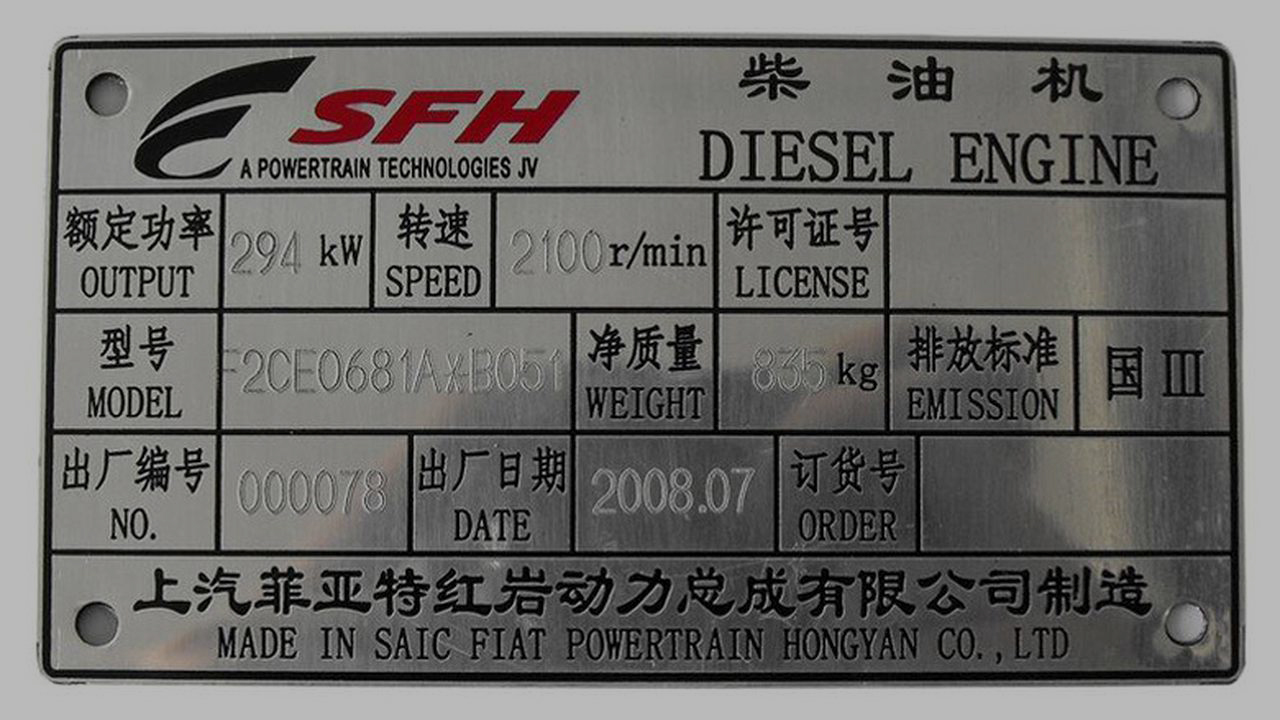

Supplement: S1 Dataset — (ZIP) [file pone.0300792.s001.zip › minimal data set/gt_img_0021_N1.0.jpg]

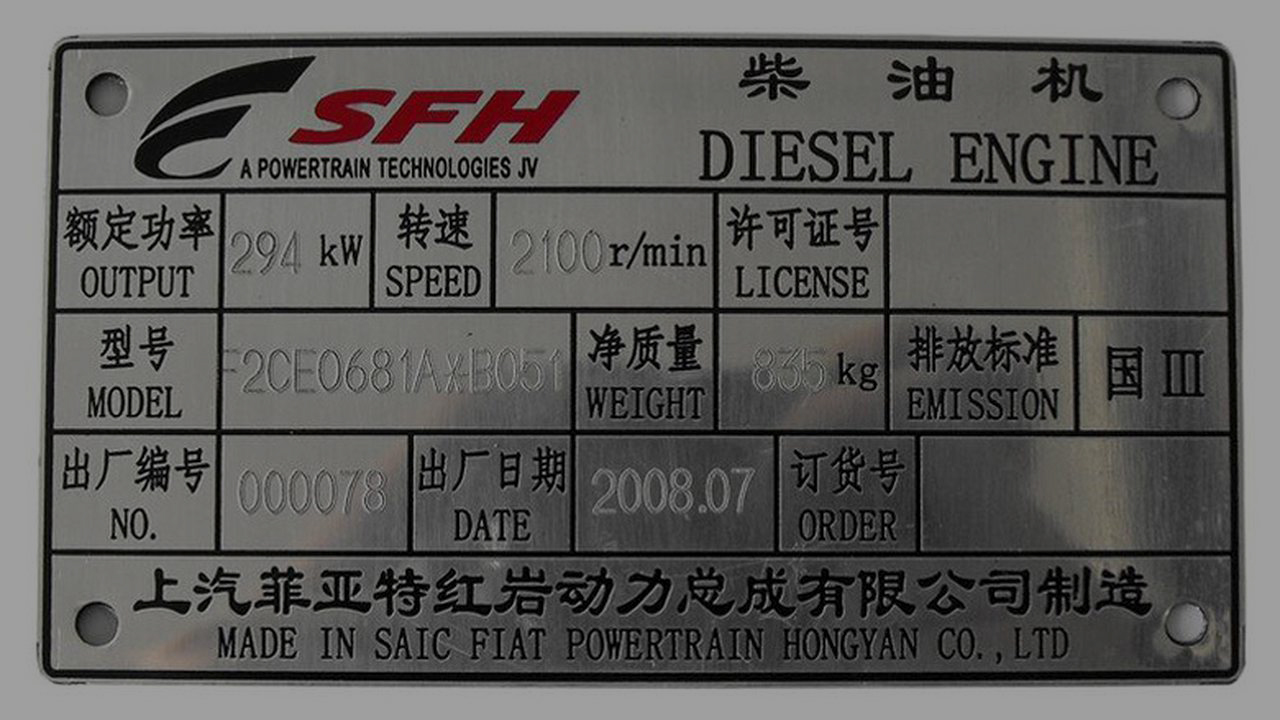

Supplement: S1 Dataset — (ZIP) [file pone.0300792.s001.zip › minimal data set/gt_img_0021_N1.5.jpg]

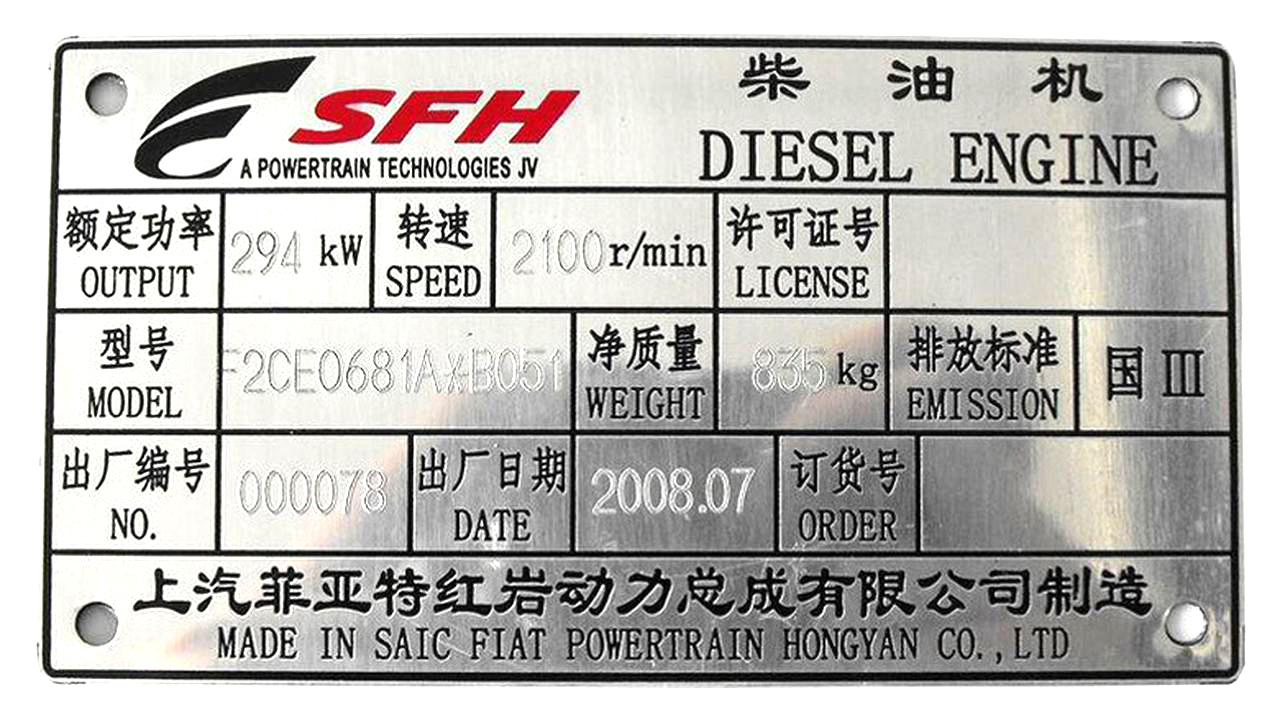

Supplement: S1 Dataset — (ZIP) [file pone.0300792.s001.zip › minimal data set/gt_img_0021_P1.0.jpg]

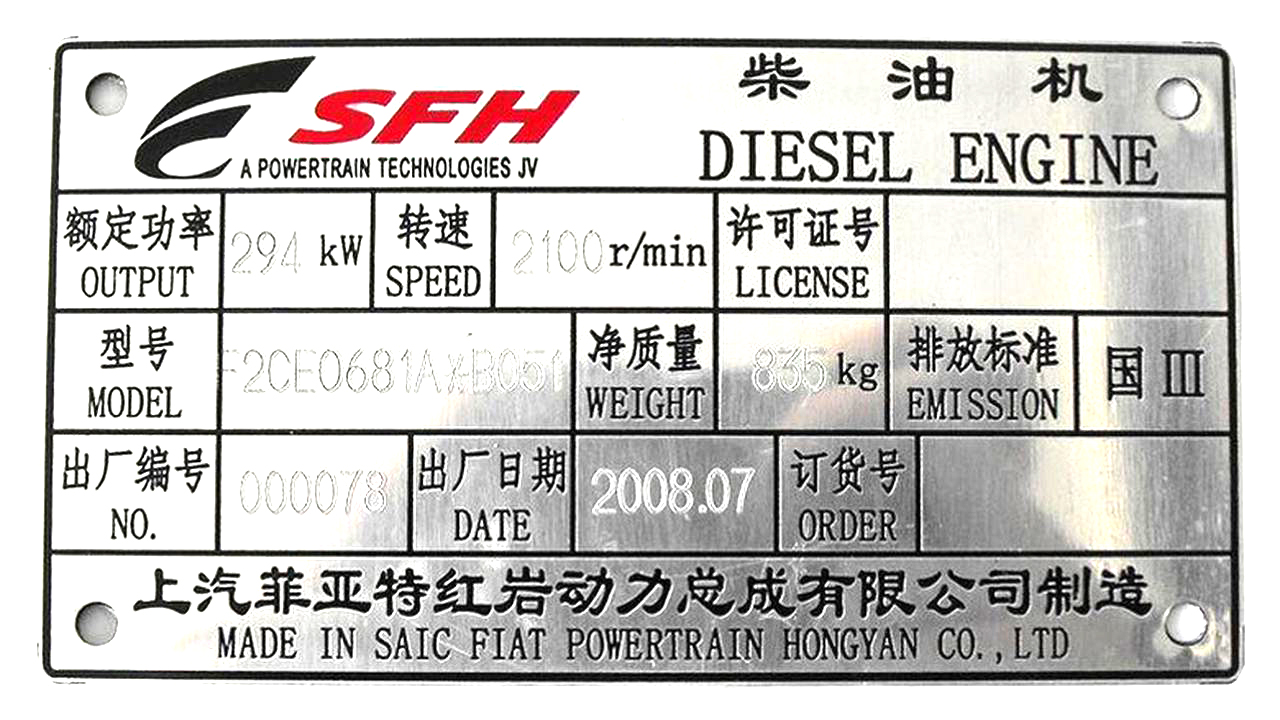

Supplement: S1 Dataset — (ZIP) [file pone.0300792.s001.zip › minimal data set/gt_img_0021_P1.5.jpg]

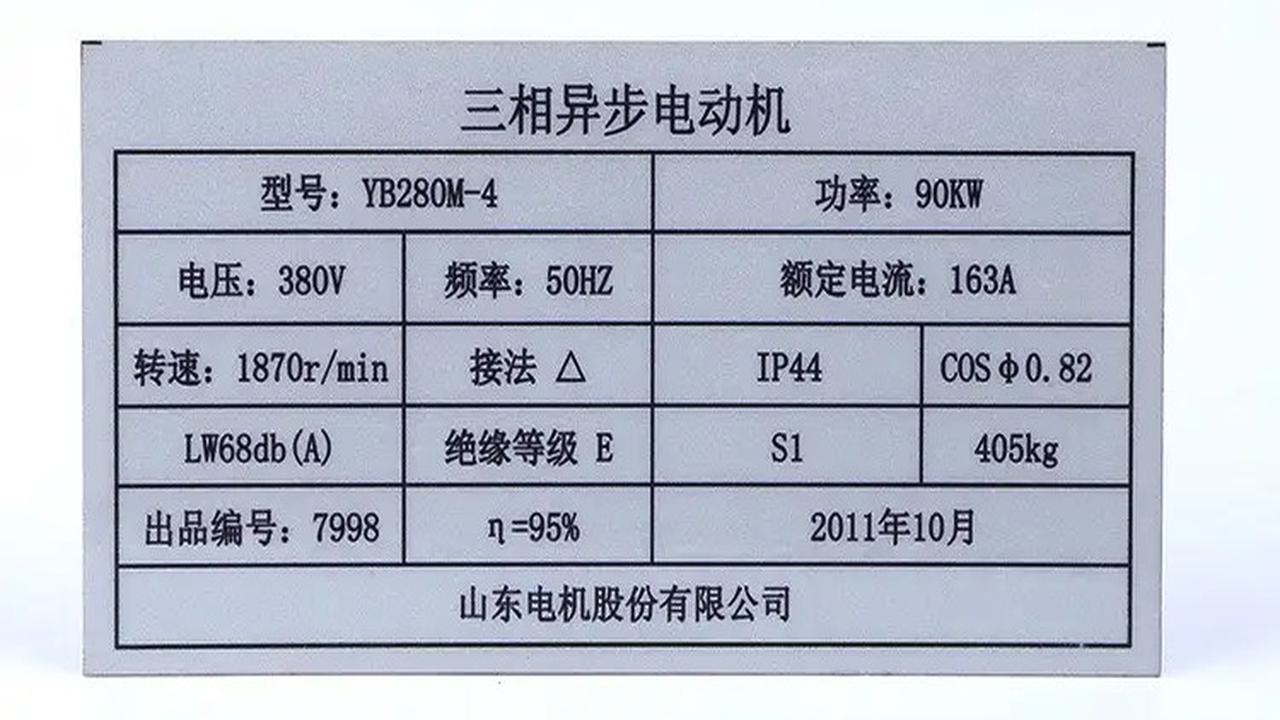

Supplement: S1 Dataset — (ZIP) [file pone.0300792.s001.zip › minimal data set/gt_img_0022_0.jpg]

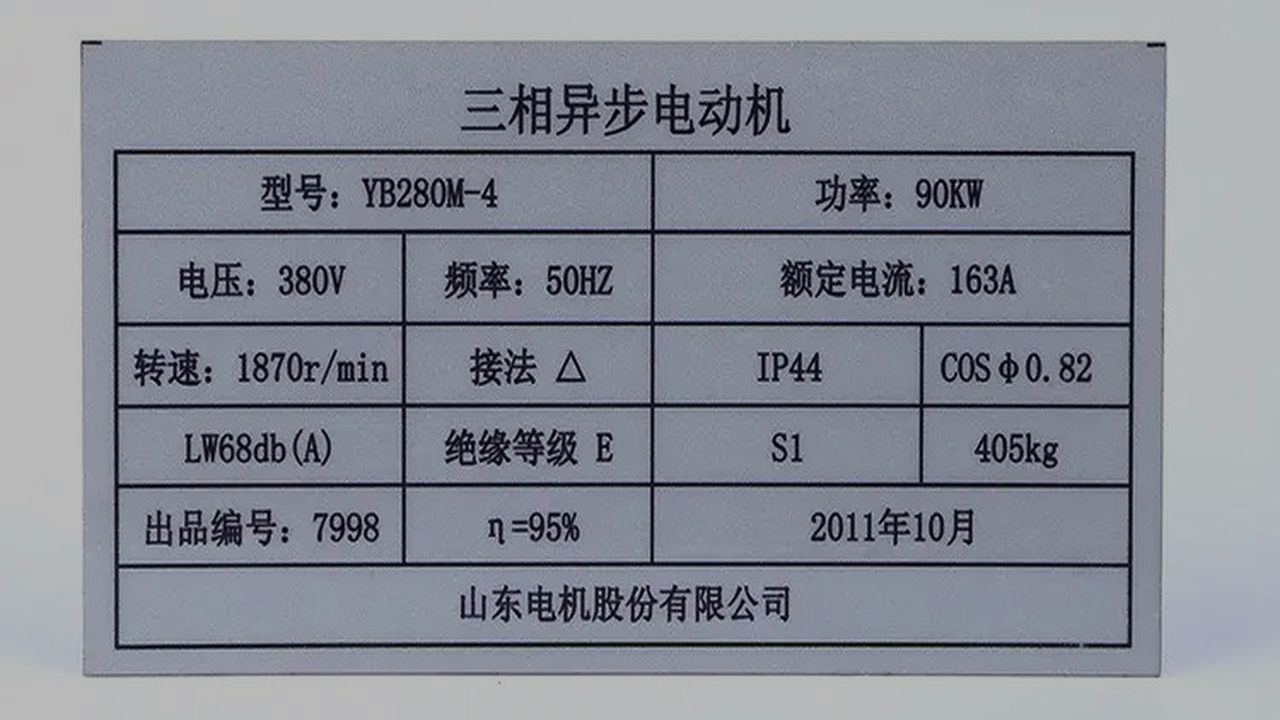

Supplement: S1 Dataset — (ZIP) [file pone.0300792.s001.zip › minimal data set/gt_img_0022_N1.0.jpg]

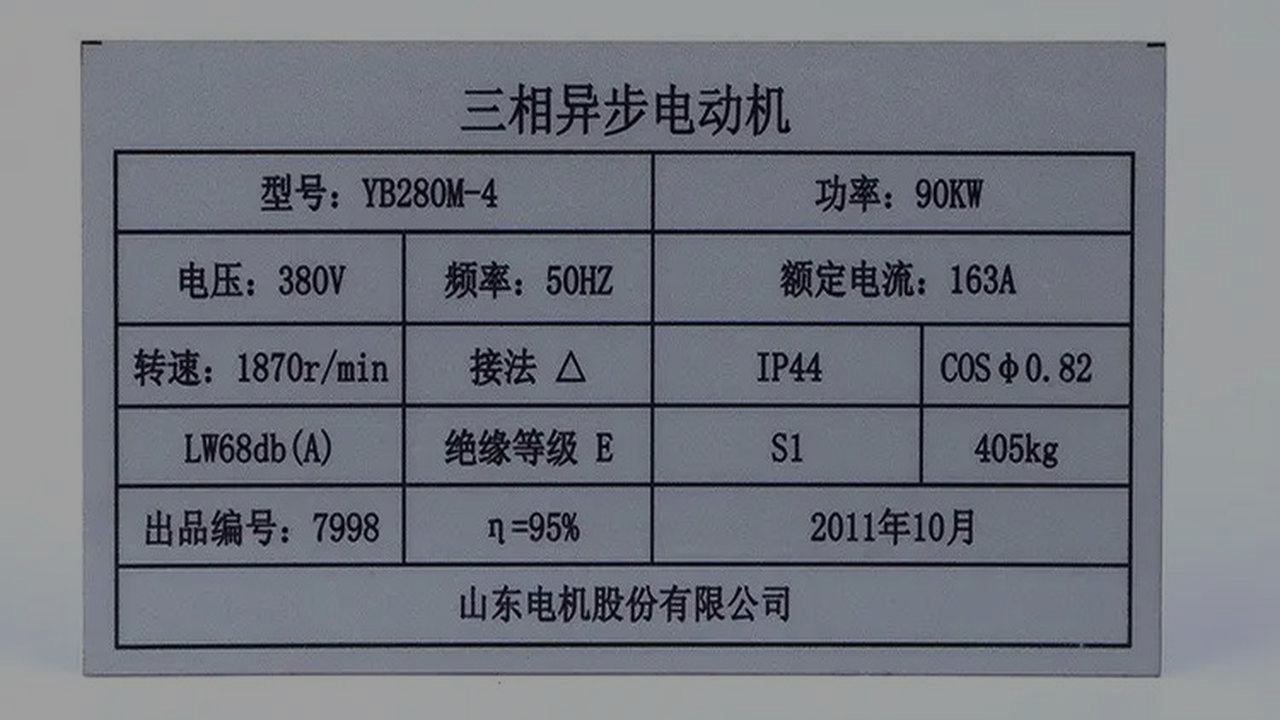

Supplement: S1 Dataset — (ZIP) [file pone.0300792.s001.zip › minimal data set/gt_img_0022_N1.5.jpg]

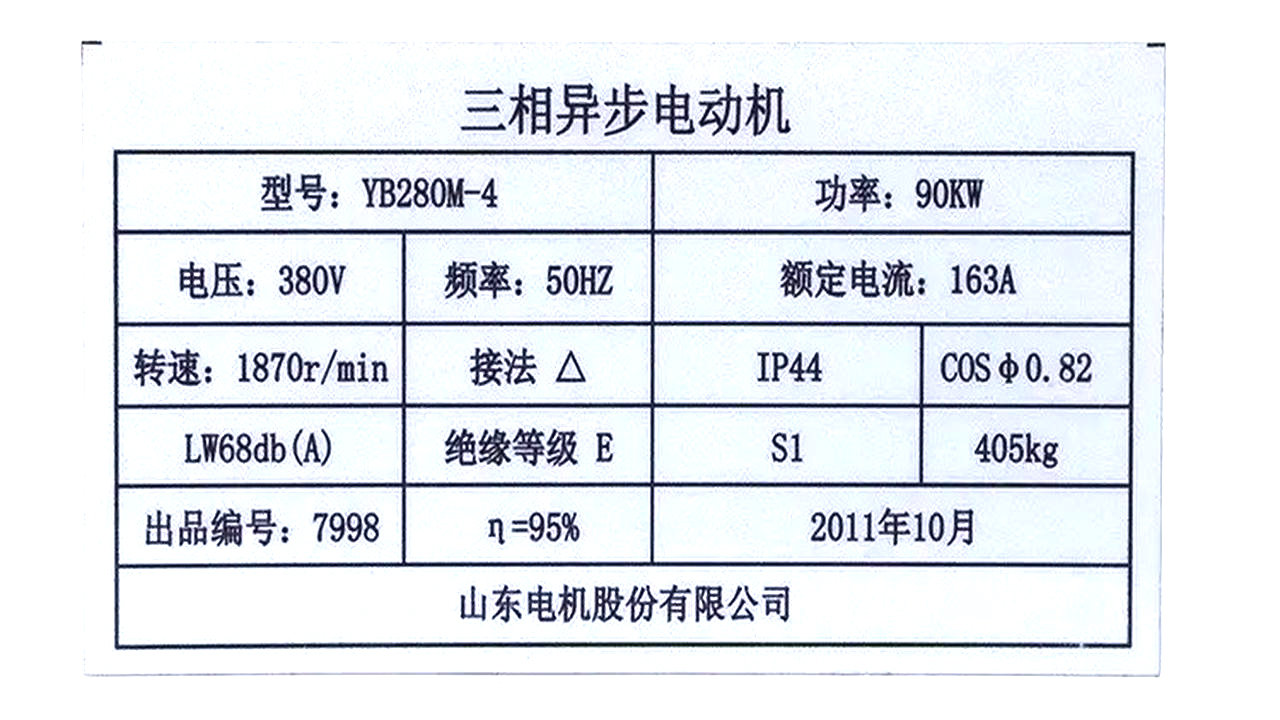

Supplement: S1 Dataset — (ZIP) [file pone.0300792.s001.zip › minimal data set/gt_img_0022_P1.0.jpg]

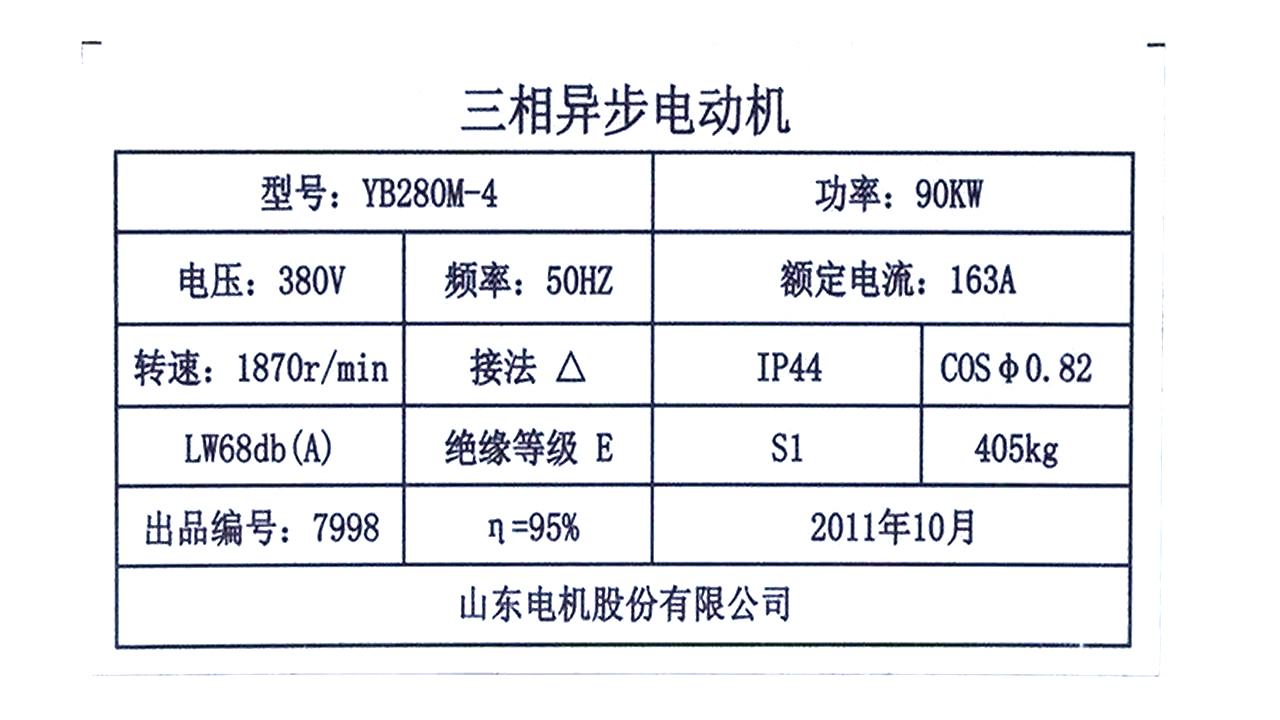

Supplement: S1 Dataset — (ZIP) [file pone.0300792.s001.zip › minimal data set/gt_img_0022_P1.5.jpg]

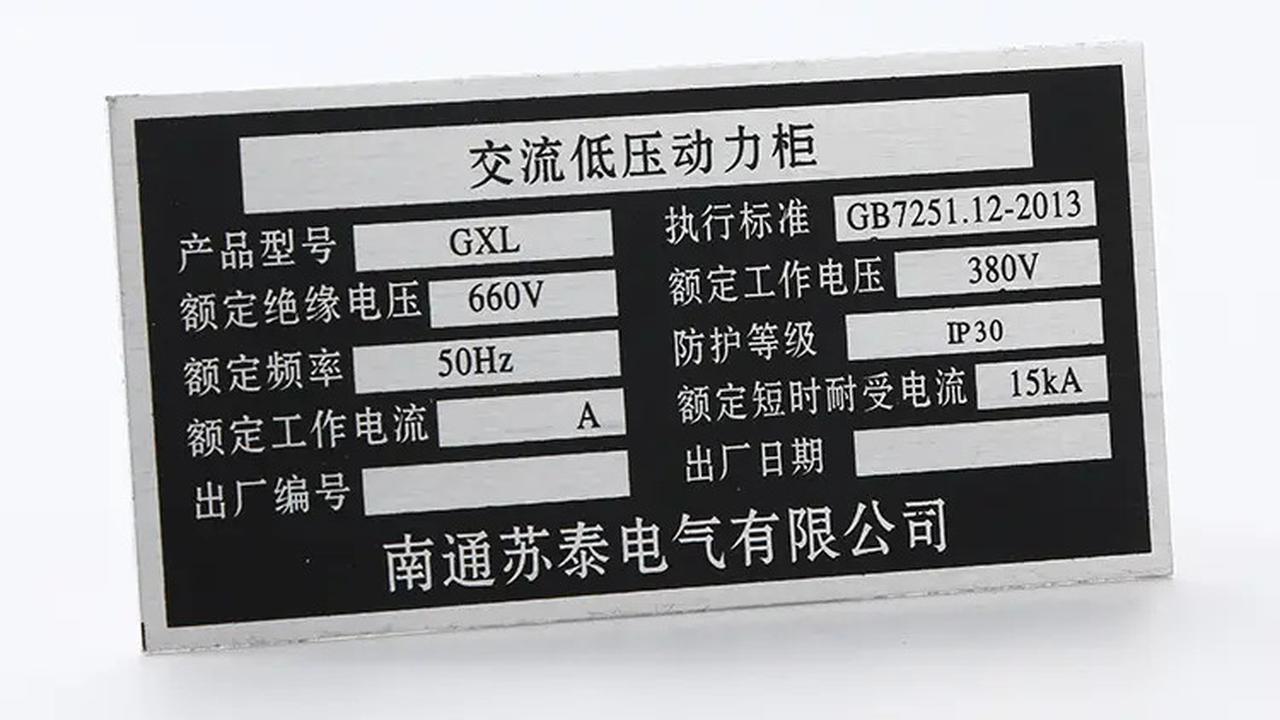

Supplement: S1 Dataset — (ZIP) [file pone.0300792.s001.zip › minimal data set/gt_img_0023_0.jpg]

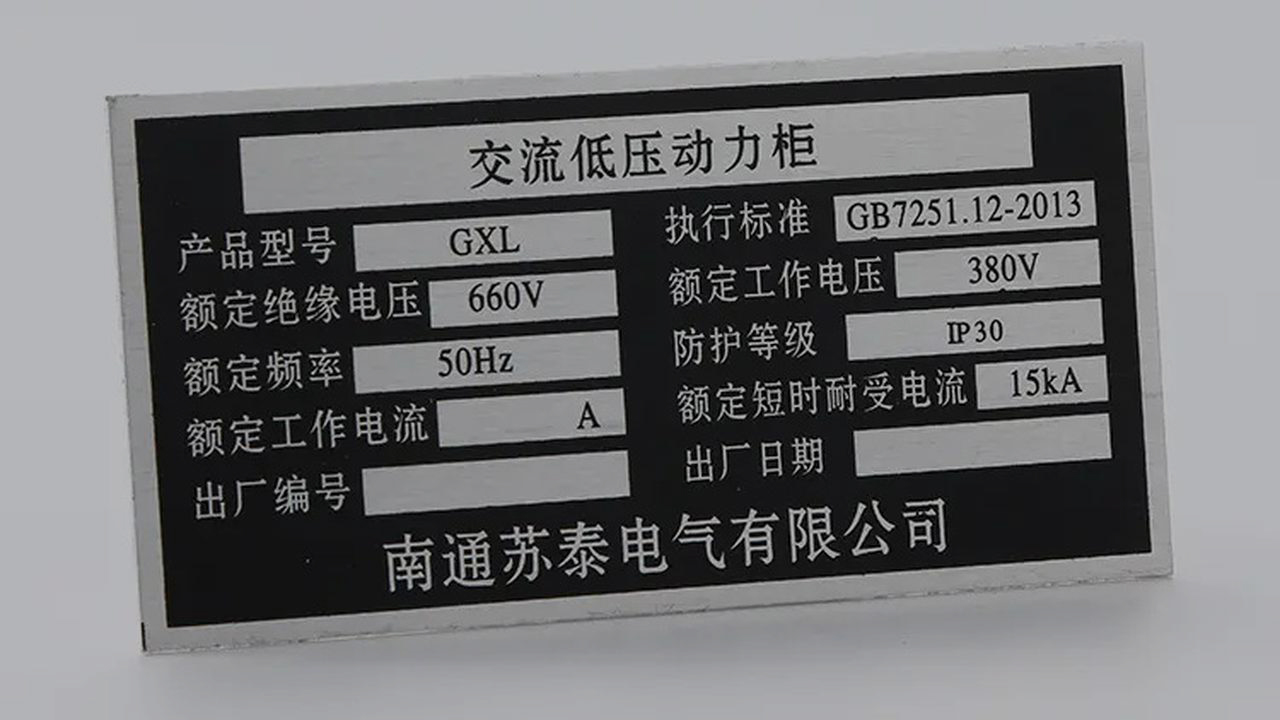

Supplement: S1 Dataset — (ZIP) [file pone.0300792.s001.zip › minimal data set/gt_img_0023_N1.0.jpg]

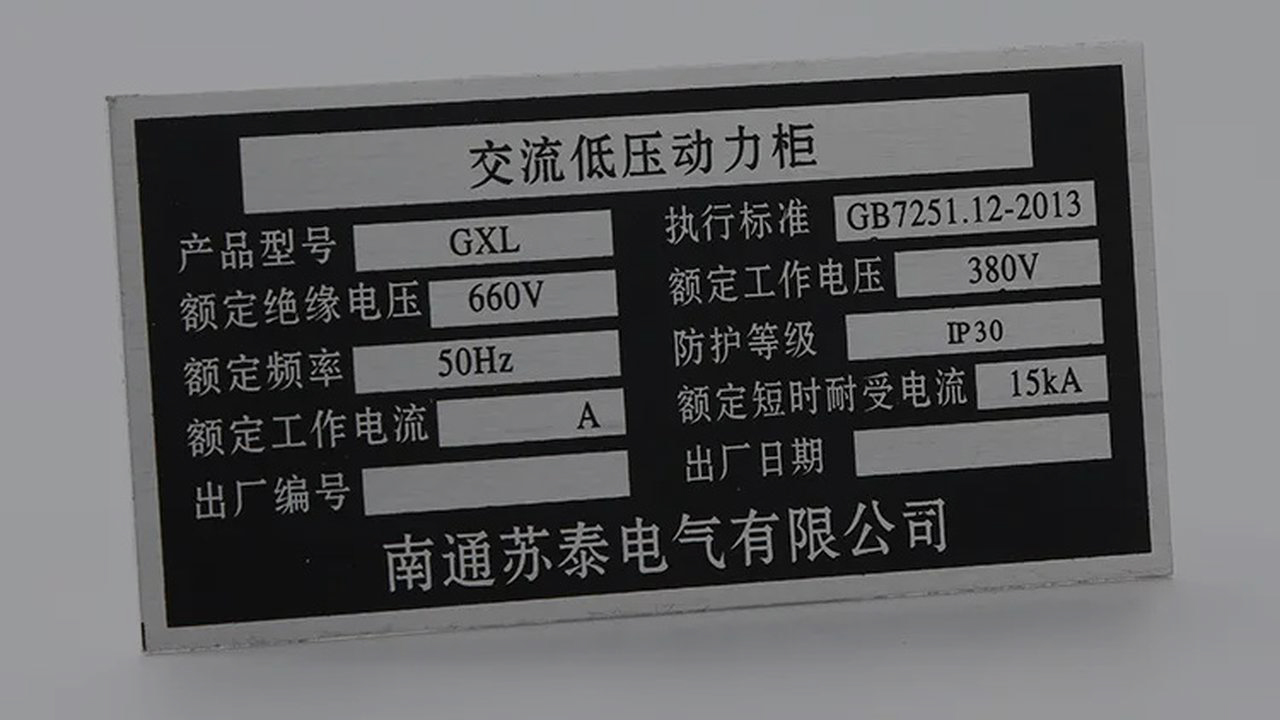

Supplement: S1 Dataset — (ZIP) [file pone.0300792.s001.zip › minimal data set/gt_img_0023_N1.5.jpg]

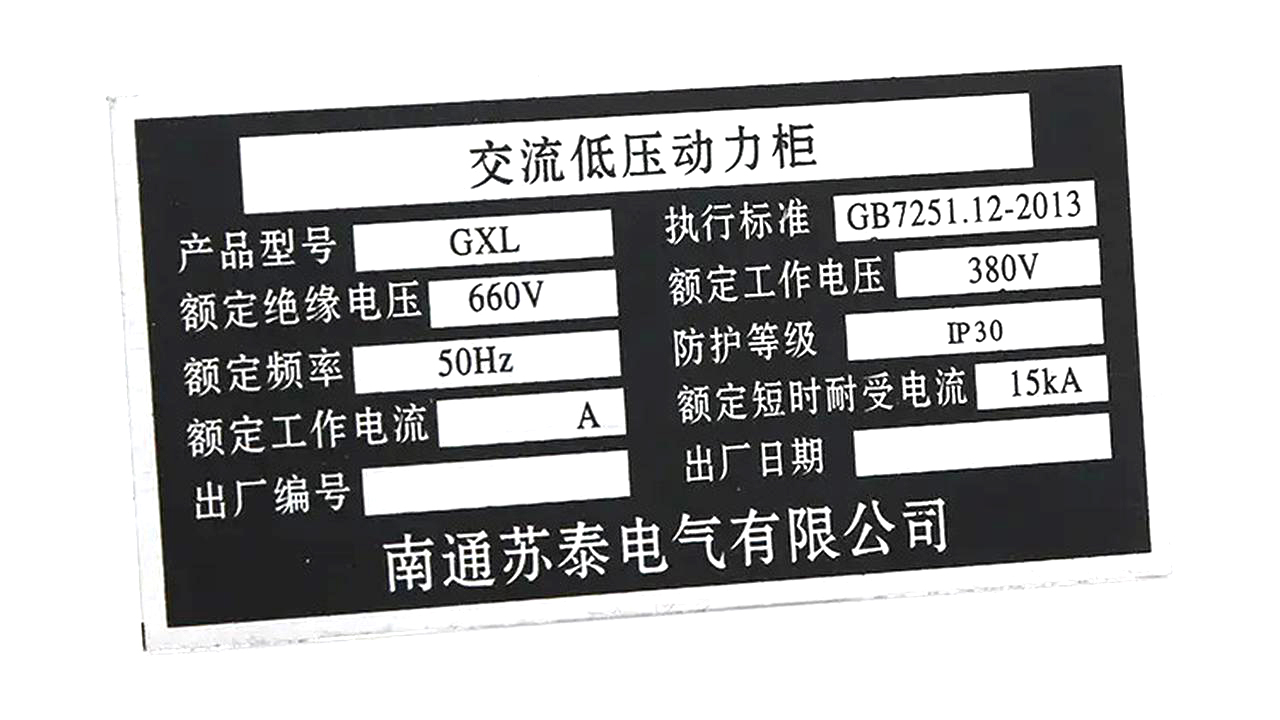

Supplement: S1 Dataset — (ZIP) [file pone.0300792.s001.zip › minimal data set/gt_img_0023_P1.0.jpg]

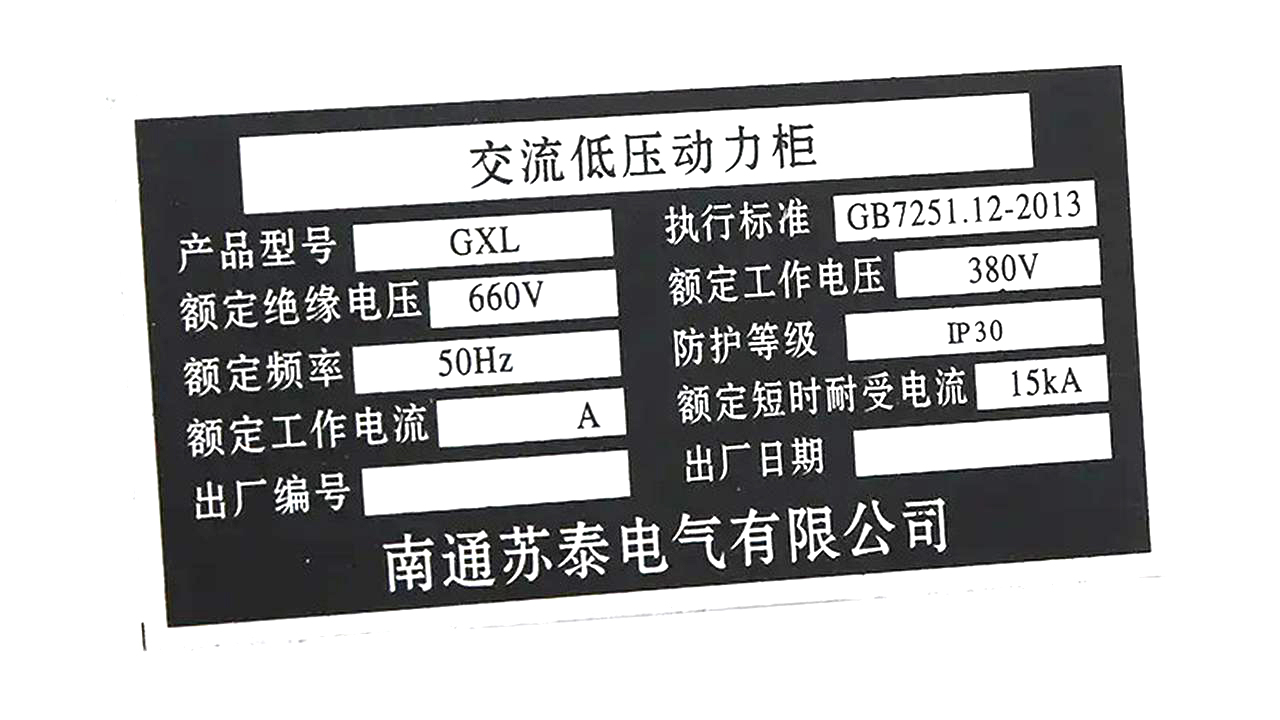

Supplement: S1 Dataset — (ZIP) [file pone.0300792.s001.zip › minimal data set/gt_img_0023_P1.5.jpg]

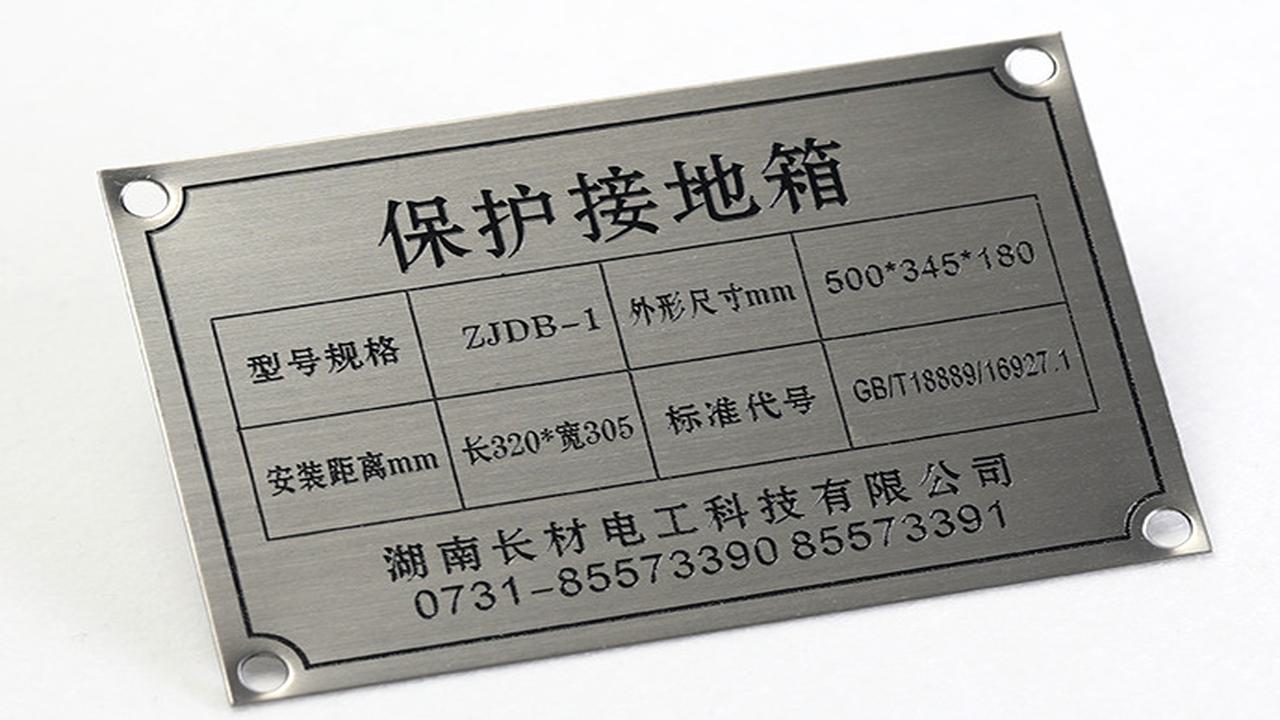

Supplement: S1 Dataset — (ZIP) [file pone.0300792.s001.zip › minimal data set/gt_img_0024_0.jpg]

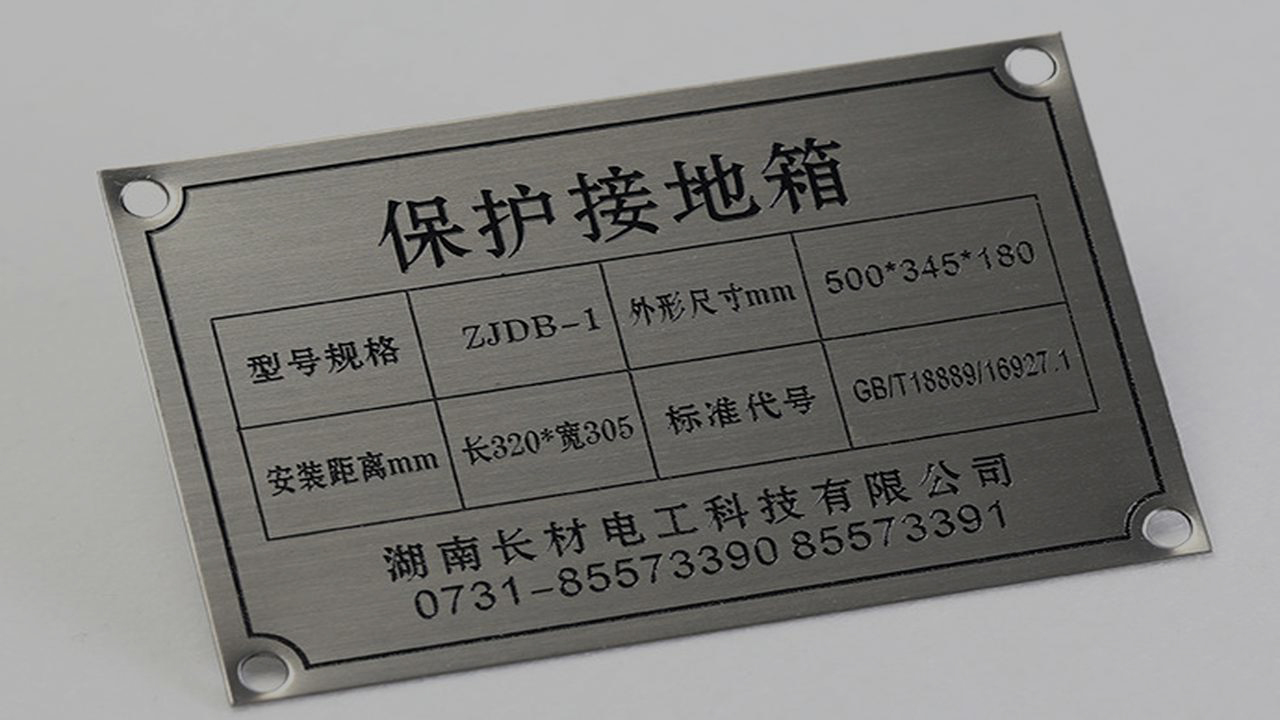

Supplement: S1 Dataset — (ZIP) [file pone.0300792.s001.zip › minimal data set/gt_img_0024_N1.0.jpg]

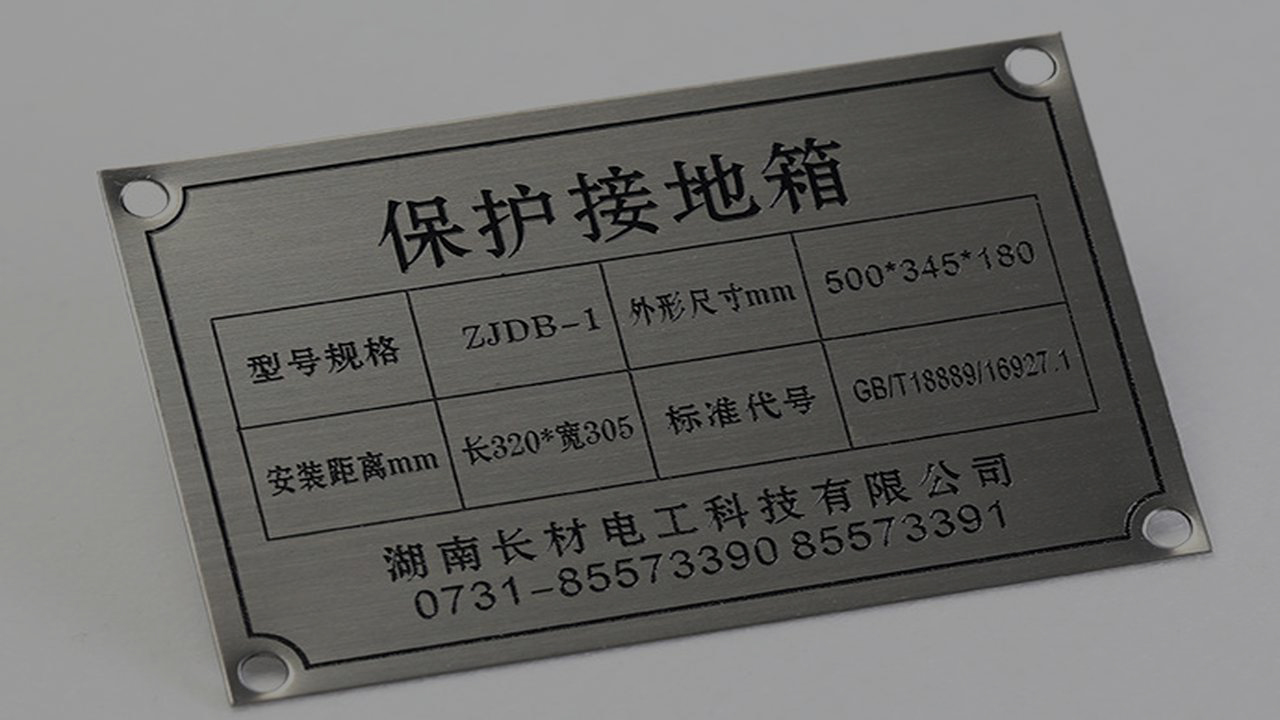

Supplement: S1 Dataset — (ZIP) [file pone.0300792.s001.zip › minimal data set/gt_img_0024_N1.5.jpg]

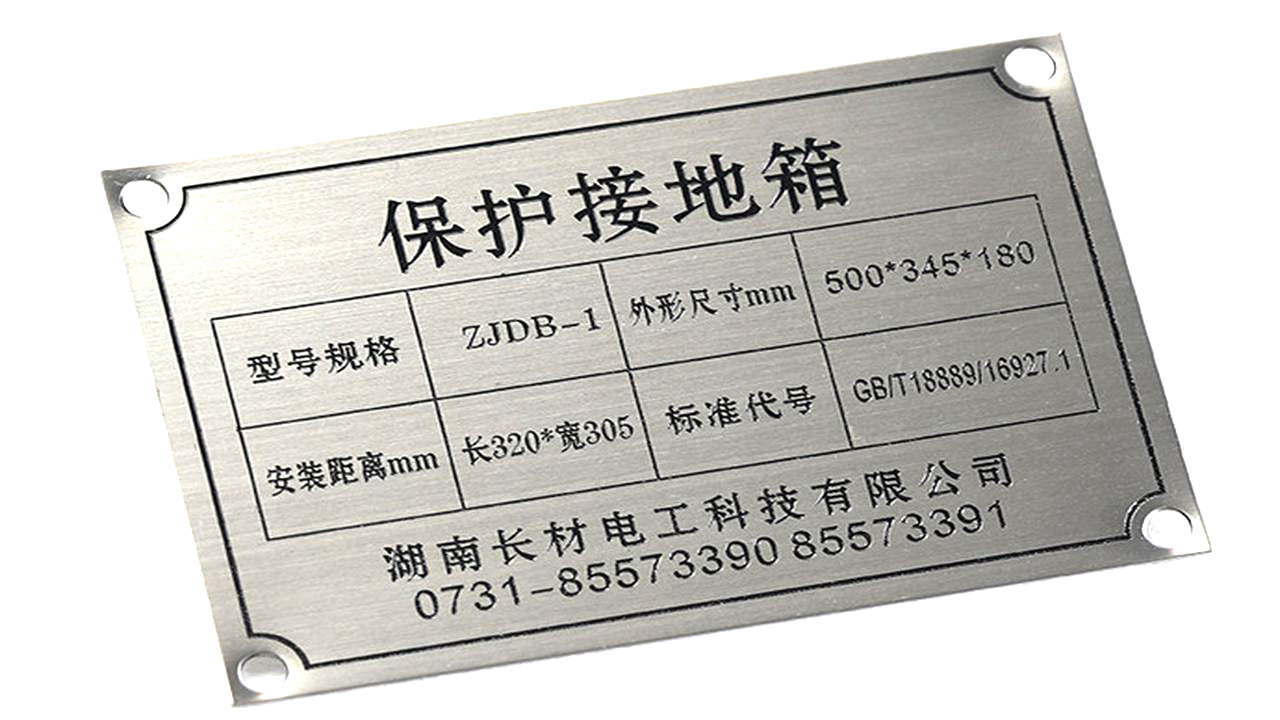

Supplement: S1 Dataset — (ZIP) [file pone.0300792.s001.zip › minimal data set/gt_img_0024_P1.0.jpg]

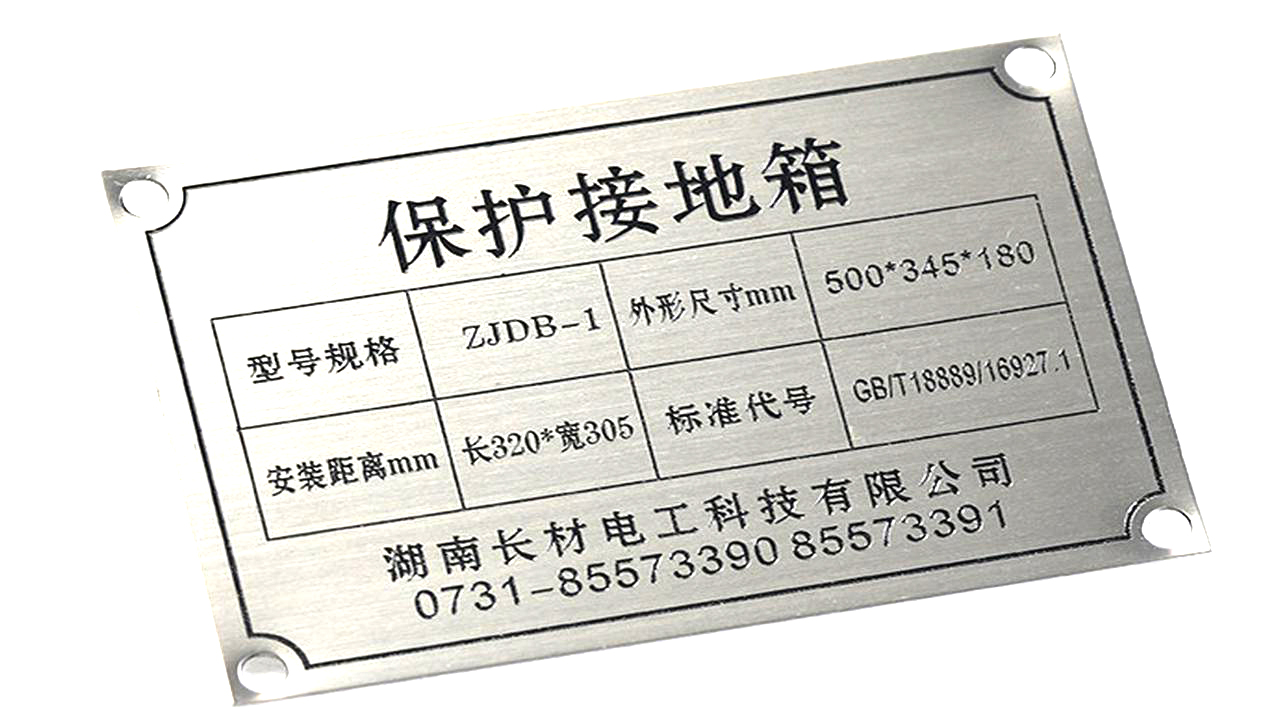

Supplement: S1 Dataset — (ZIP) [file pone.0300792.s001.zip › minimal data set/gt_img_0024_P1.5.jpg]

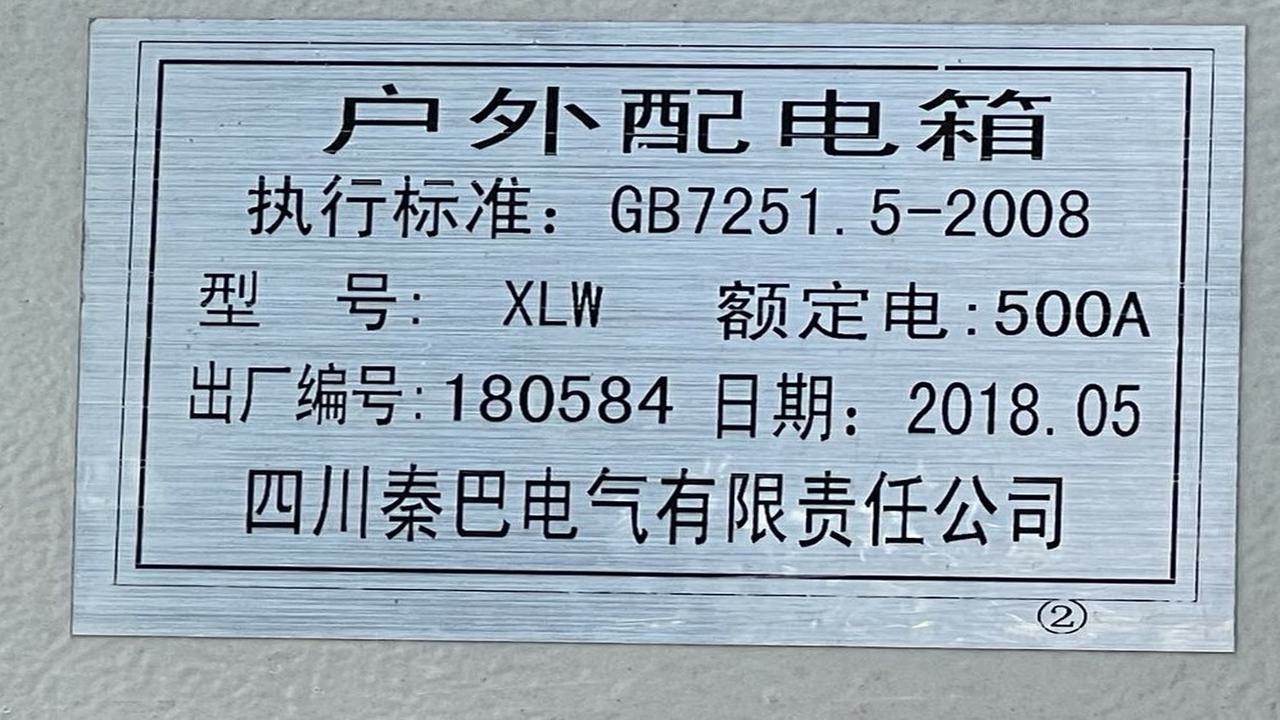

Supplement: S1 Dataset — (ZIP) [file pone.0300792.s001.zip › minimal data set/gt_img_0025_0.jpg]

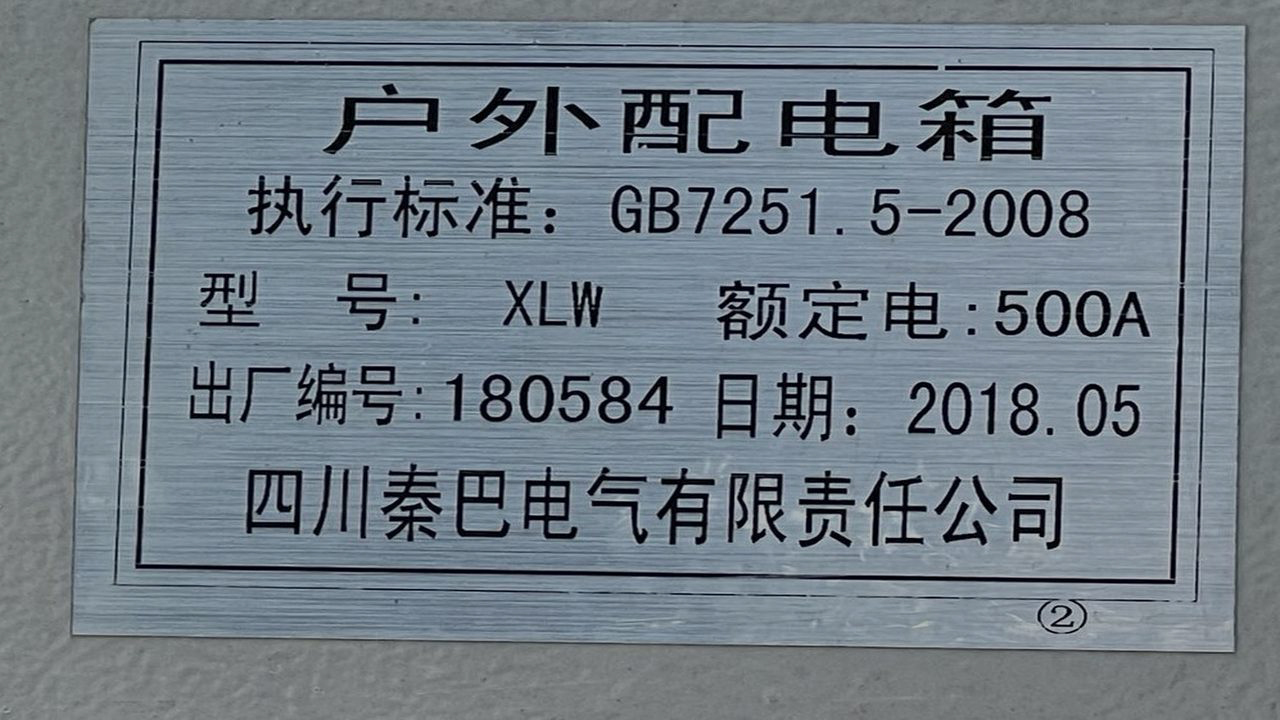

Supplement: S1 Dataset — (ZIP) [file pone.0300792.s001.zip › minimal data set/gt_img_0025_N1.0.jpg]

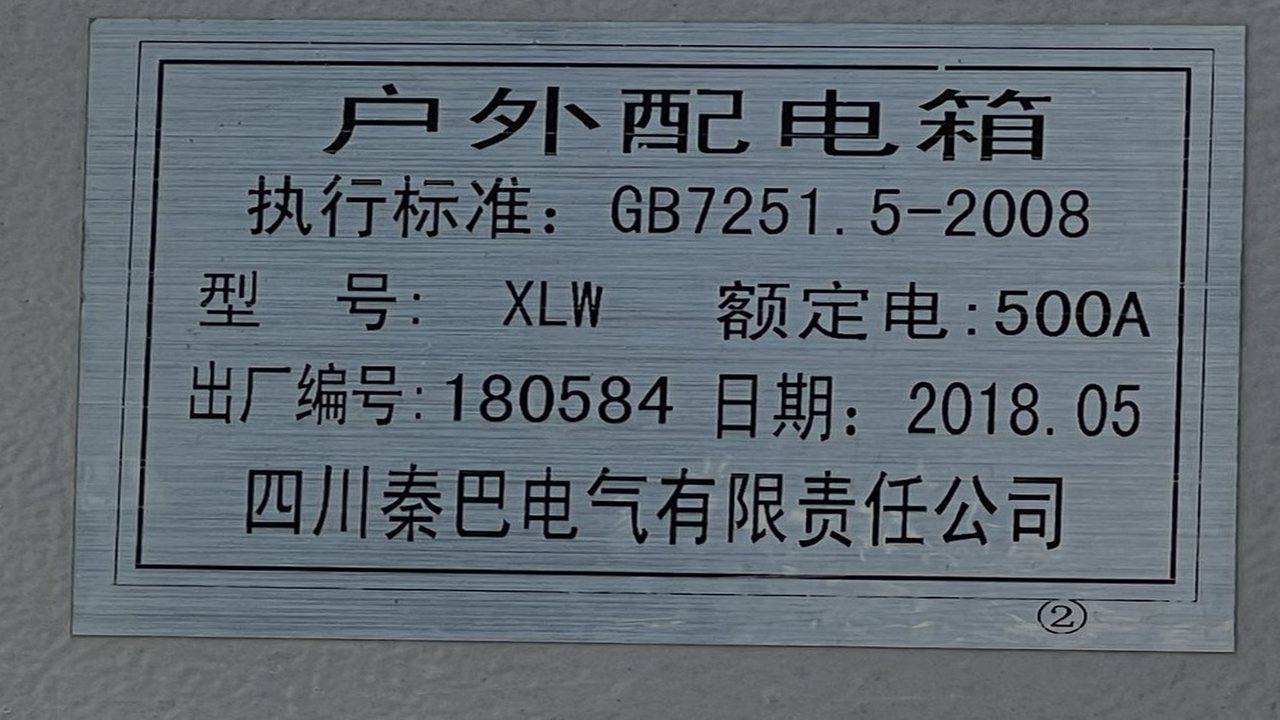

Supplement: S1 Dataset — (ZIP) [file pone.0300792.s001.zip › minimal data set/gt_img_0025_N1.5.jpg]

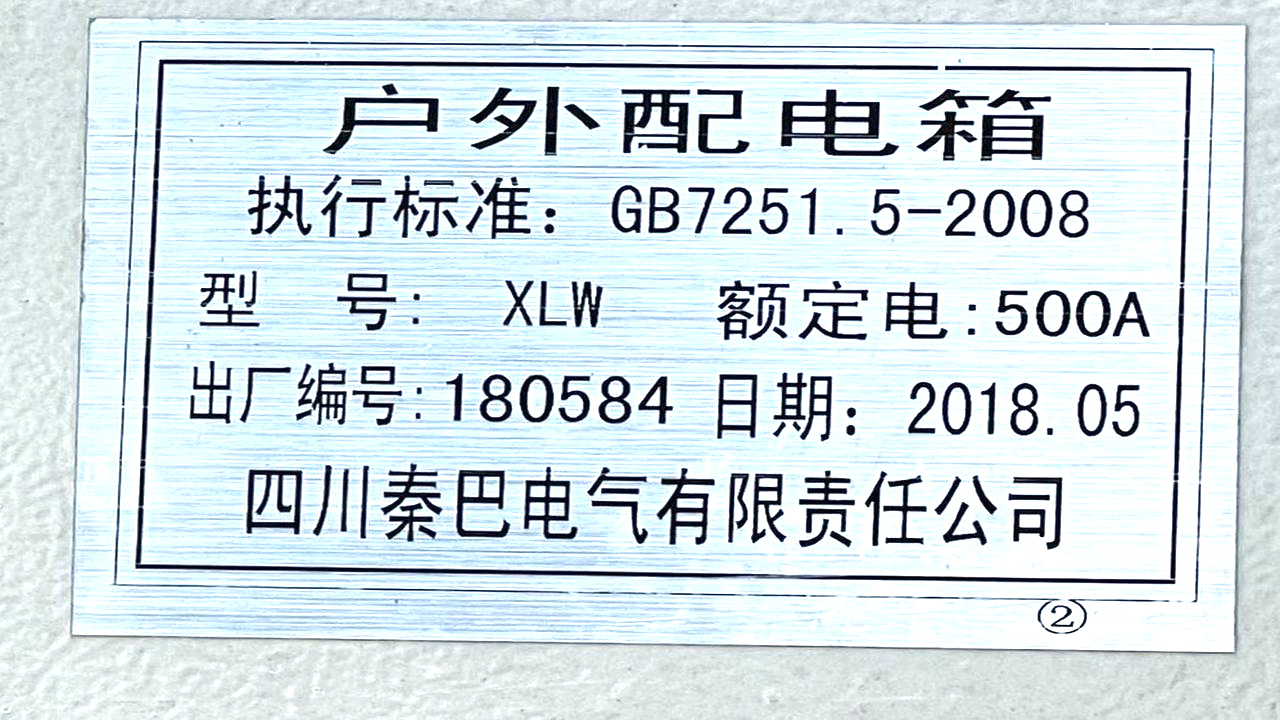

Supplement: S1 Dataset — (ZIP) [file pone.0300792.s001.zip › minimal data set/gt_img_0025_P1.0.jpg]

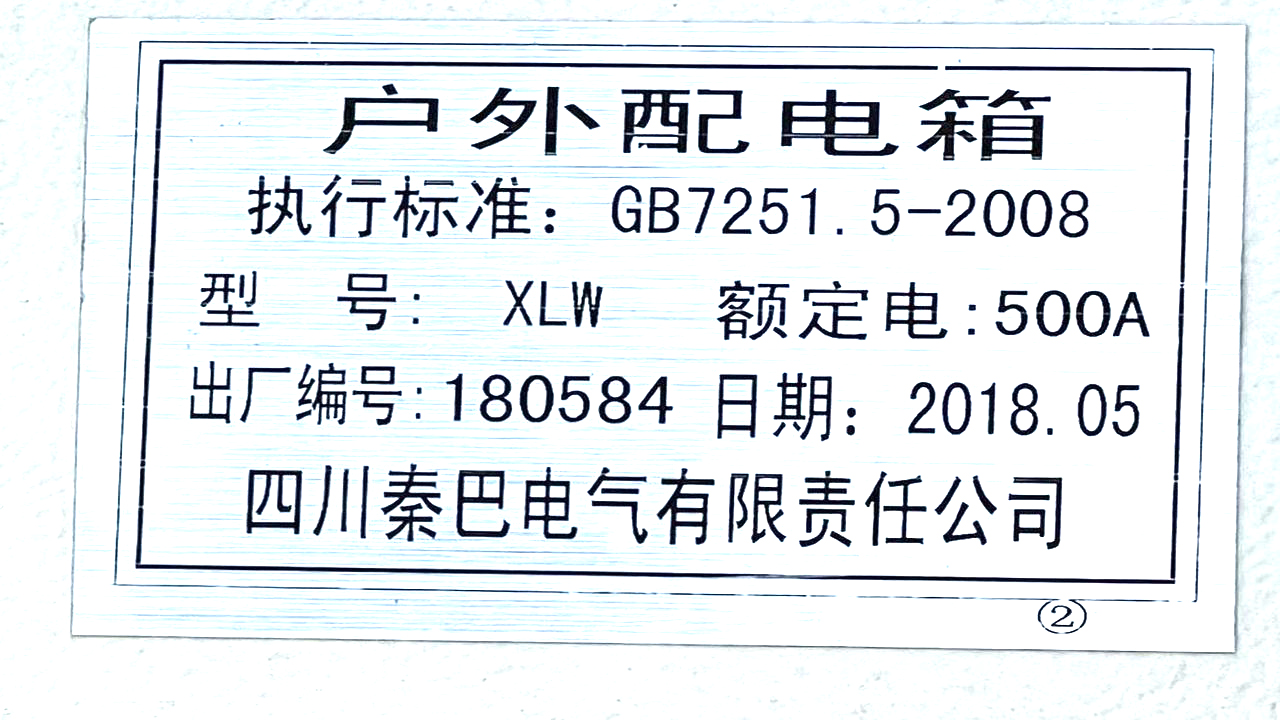

Supplement: S1 Dataset — (ZIP) [file pone.0300792.s001.zip › minimal data set/gt_img_0025_P1.5.jpg]

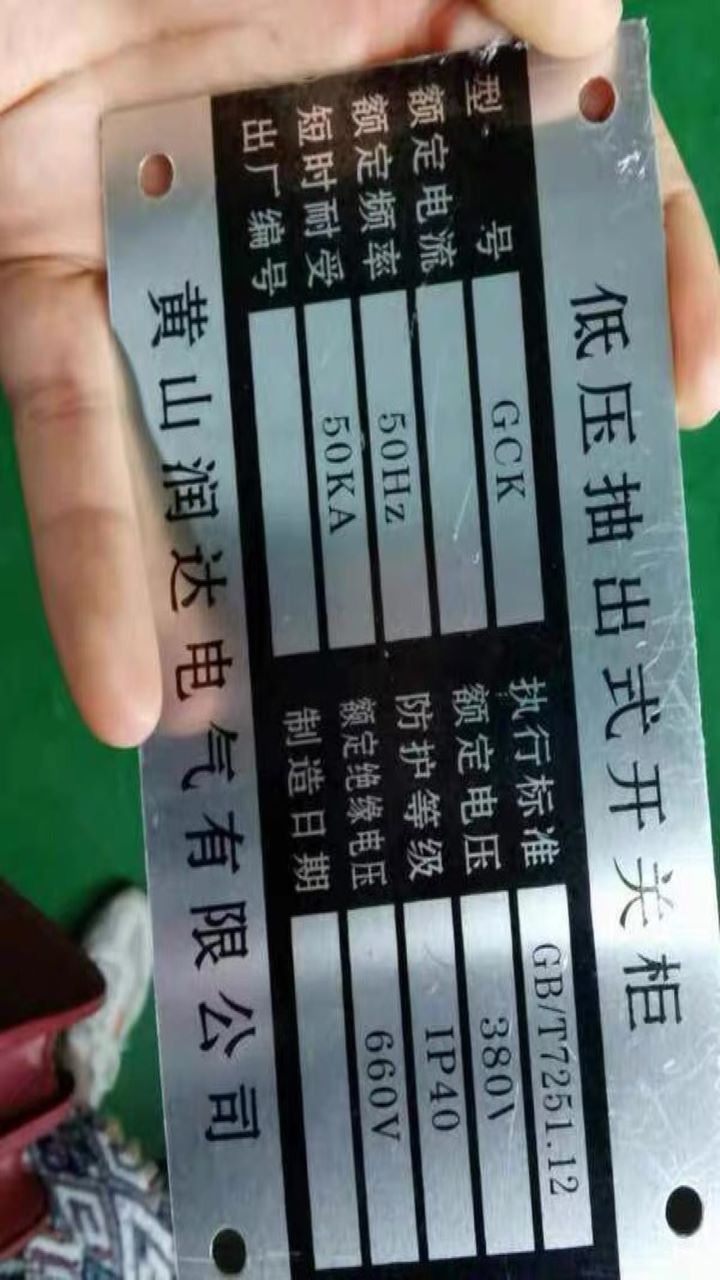

Supplement: S1 Dataset — (ZIP) [file pone.0300792.s001.zip › minimal data set/gt_img_0026_0.jpg]

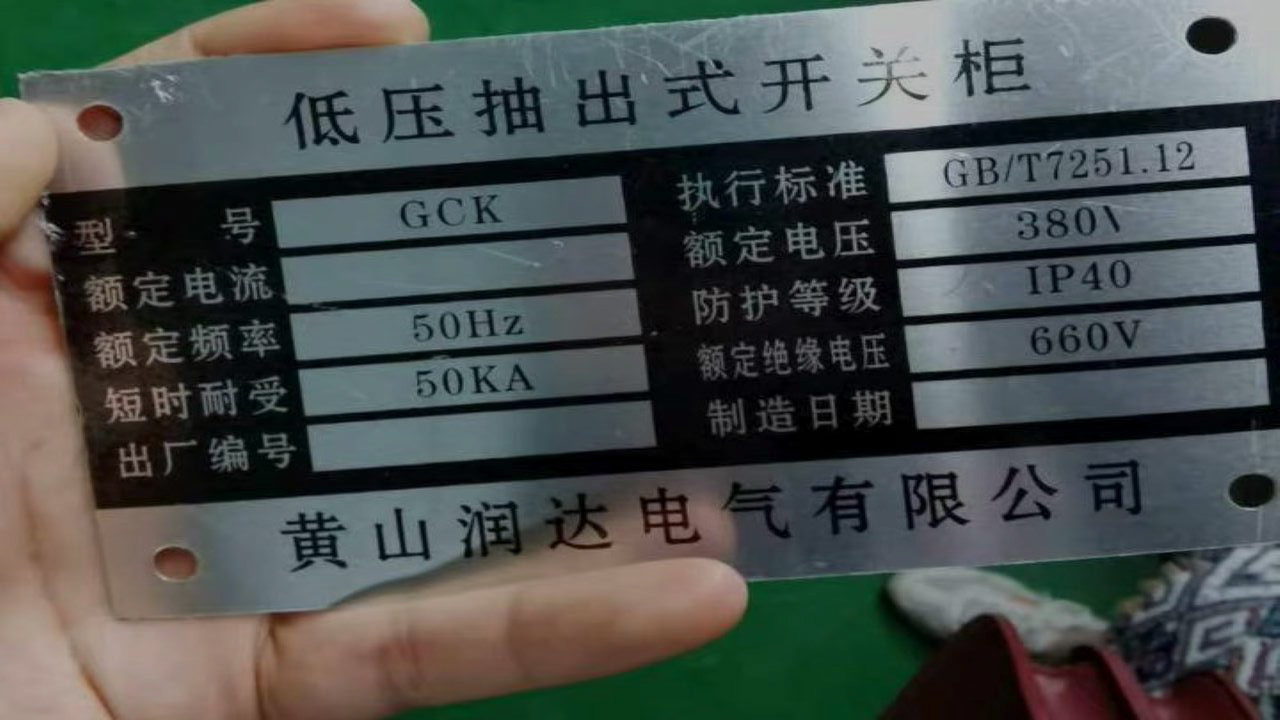

Supplement: S1 Dataset — (ZIP) [file pone.0300792.s001.zip › minimal data set/gt_img_0026_N1.0.jpg]

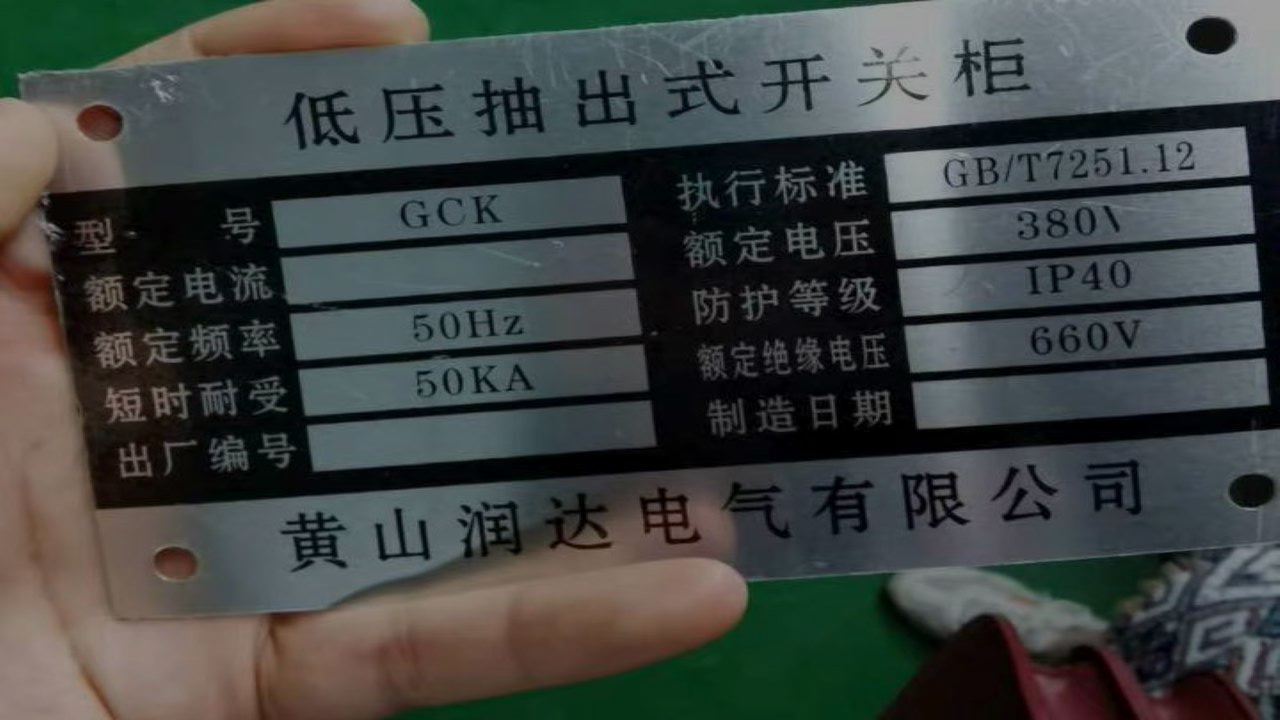

Supplement: S1 Dataset — (ZIP) [file pone.0300792.s001.zip › minimal data set/gt_img_0026_N1.5.jpg]

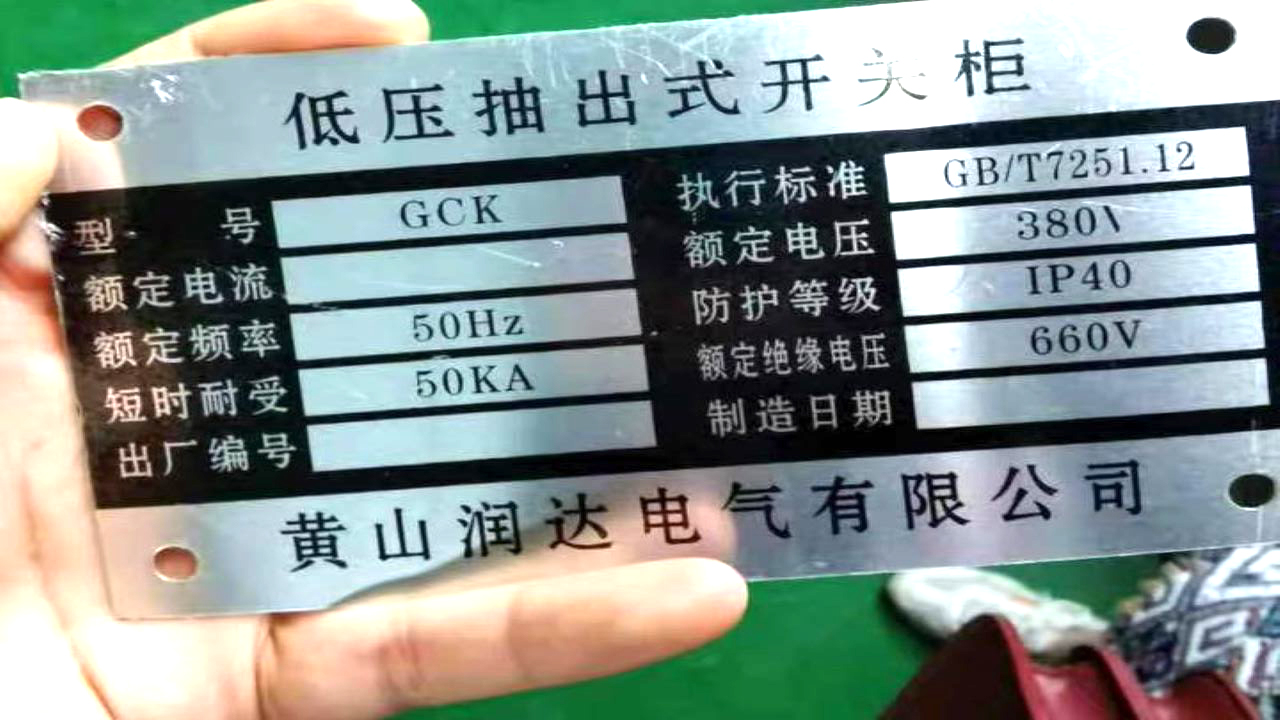

Supplement: S1 Dataset — (ZIP) [file pone.0300792.s001.zip › minimal data set/gt_img_0026_P1.0.jpg]

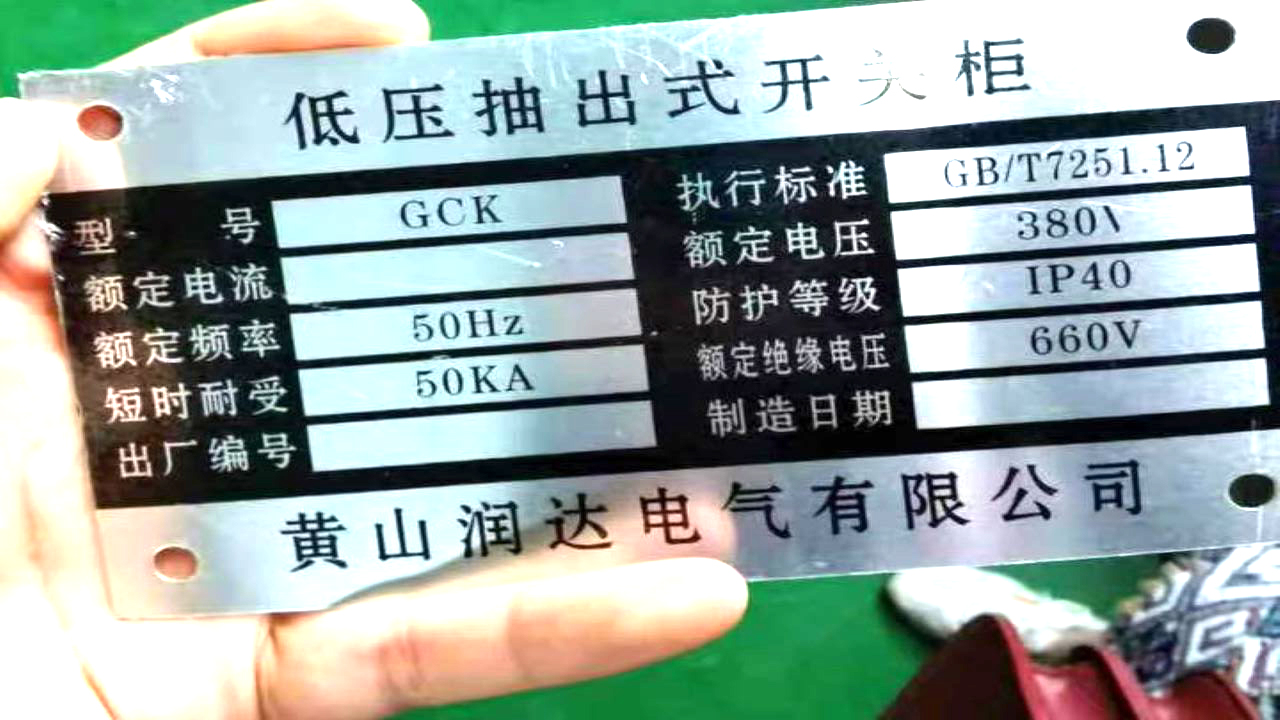

Supplement: S1 Dataset — (ZIP) [file pone.0300792.s001.zip › minimal data set/gt_img_0026_P1.5.jpg]

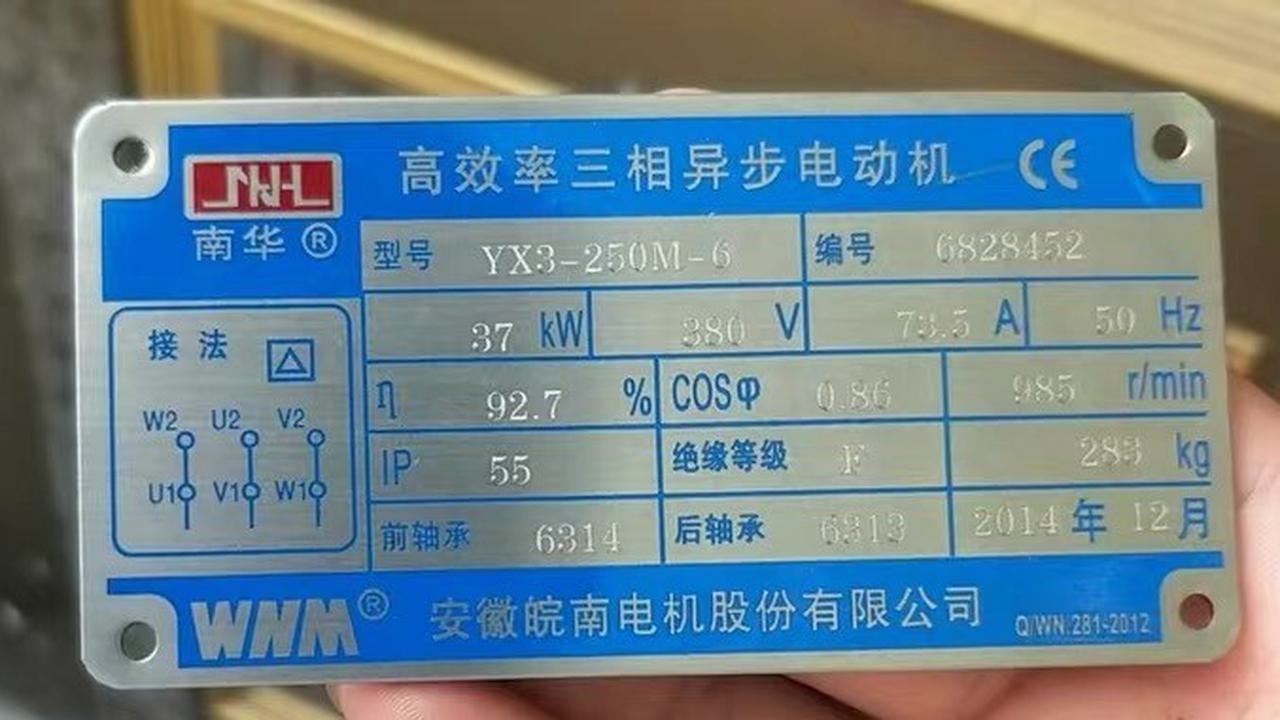

Supplement: S1 Dataset — (ZIP) [file pone.0300792.s001.zip › minimal data set/gt_img_0027_0.jpg]

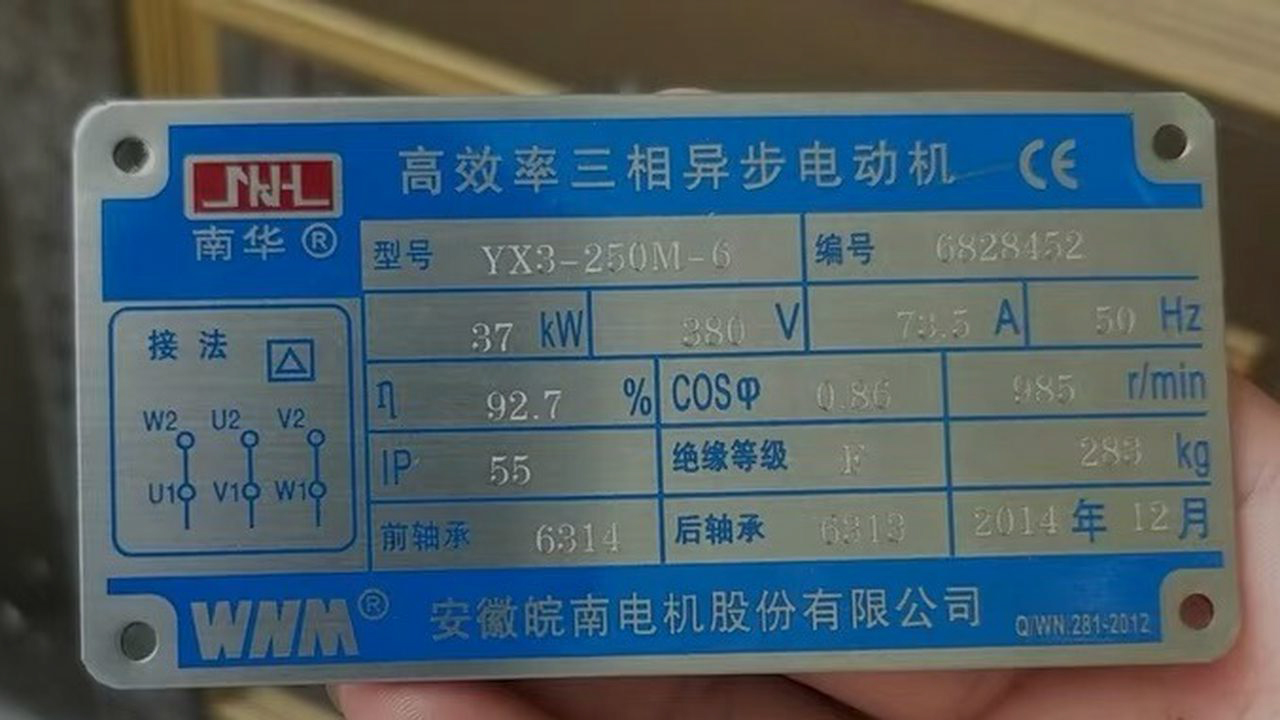

Supplement: S1 Dataset — (ZIP) [file pone.0300792.s001.zip › minimal data set/gt_img_0027_N1.0.jpg]

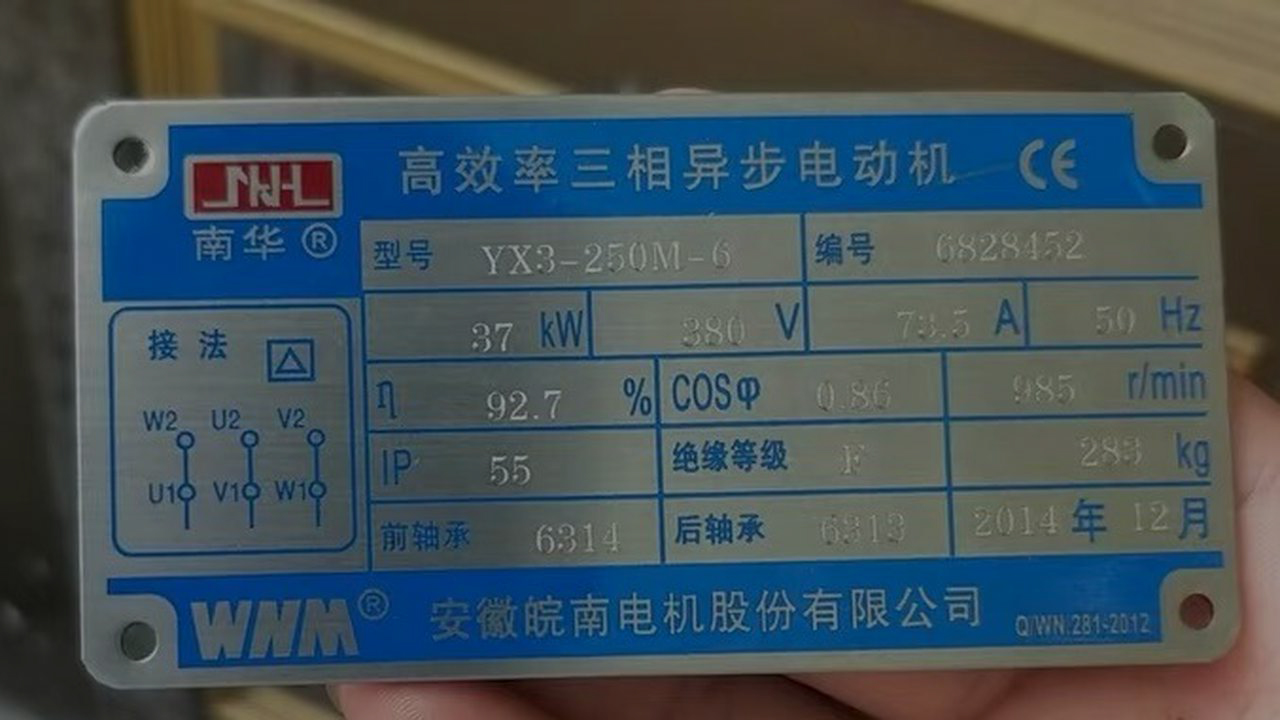

Supplement: S1 Dataset — (ZIP) [file pone.0300792.s001.zip › minimal data set/gt_img_0027_N1.5.jpg]

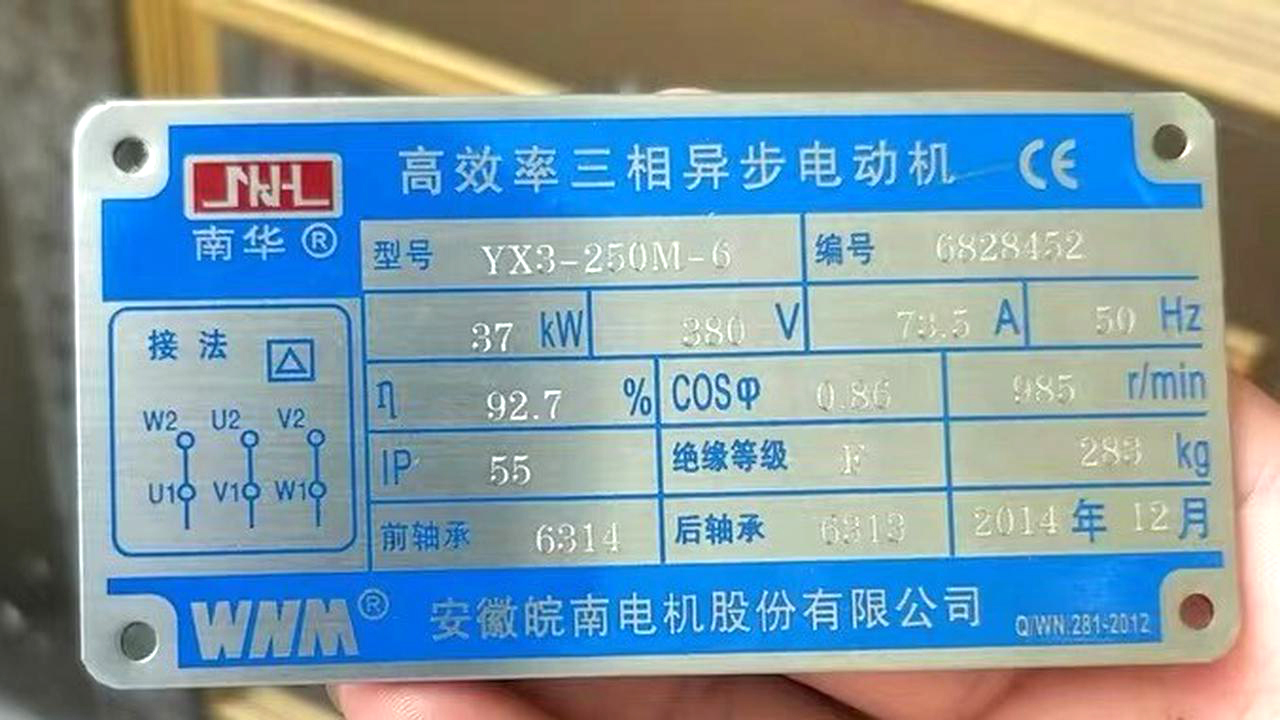

Supplement: S1 Dataset — (ZIP) [file pone.0300792.s001.zip › minimal data set/gt_img_0027_P1.0.jpg]

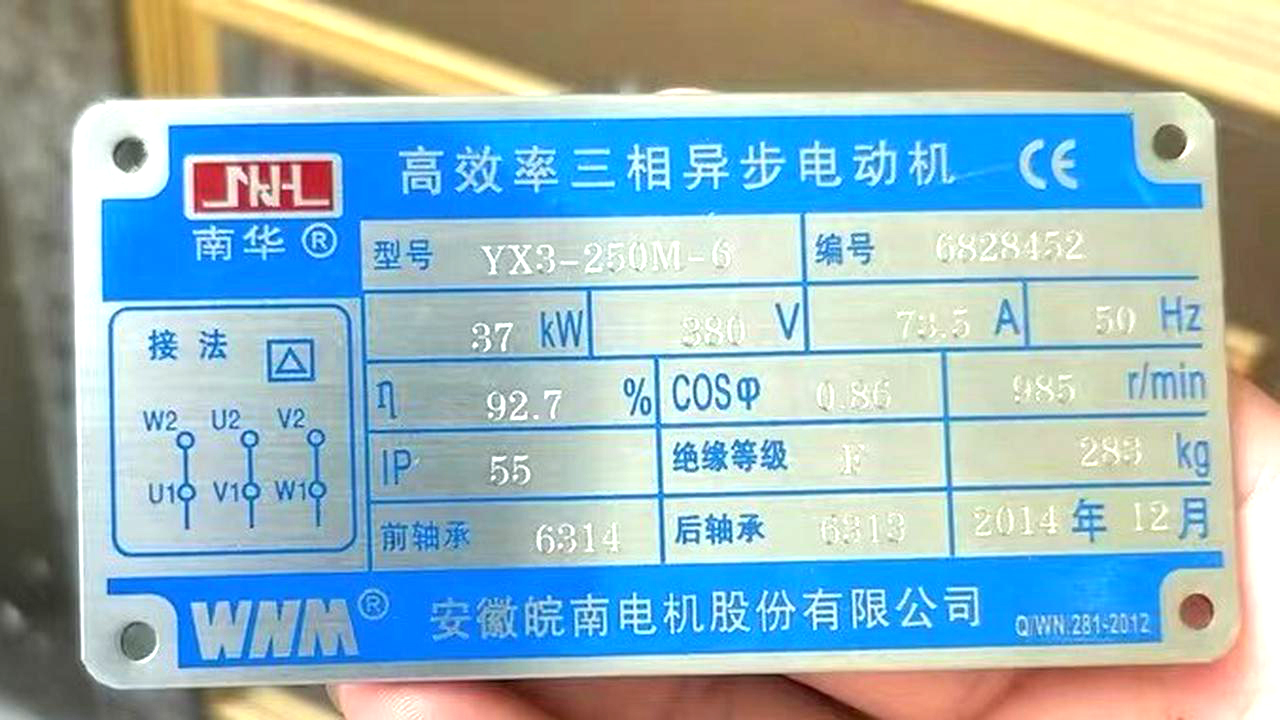

Supplement: S1 Dataset — (ZIP) [file pone.0300792.s001.zip › minimal data set/gt_img_0027_P1.5.jpg]

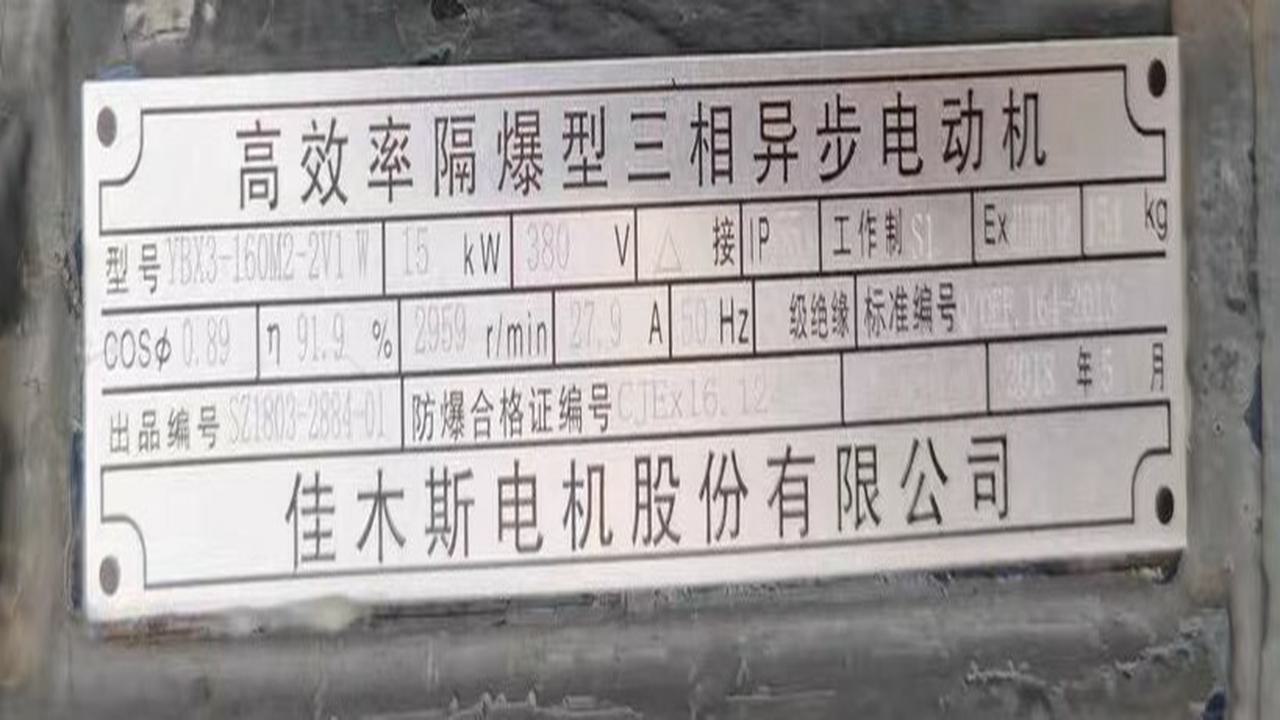

Supplement: S1 Dataset — (ZIP) [file pone.0300792.s001.zip › minimal data set/gt_img_0028_0.jpg]

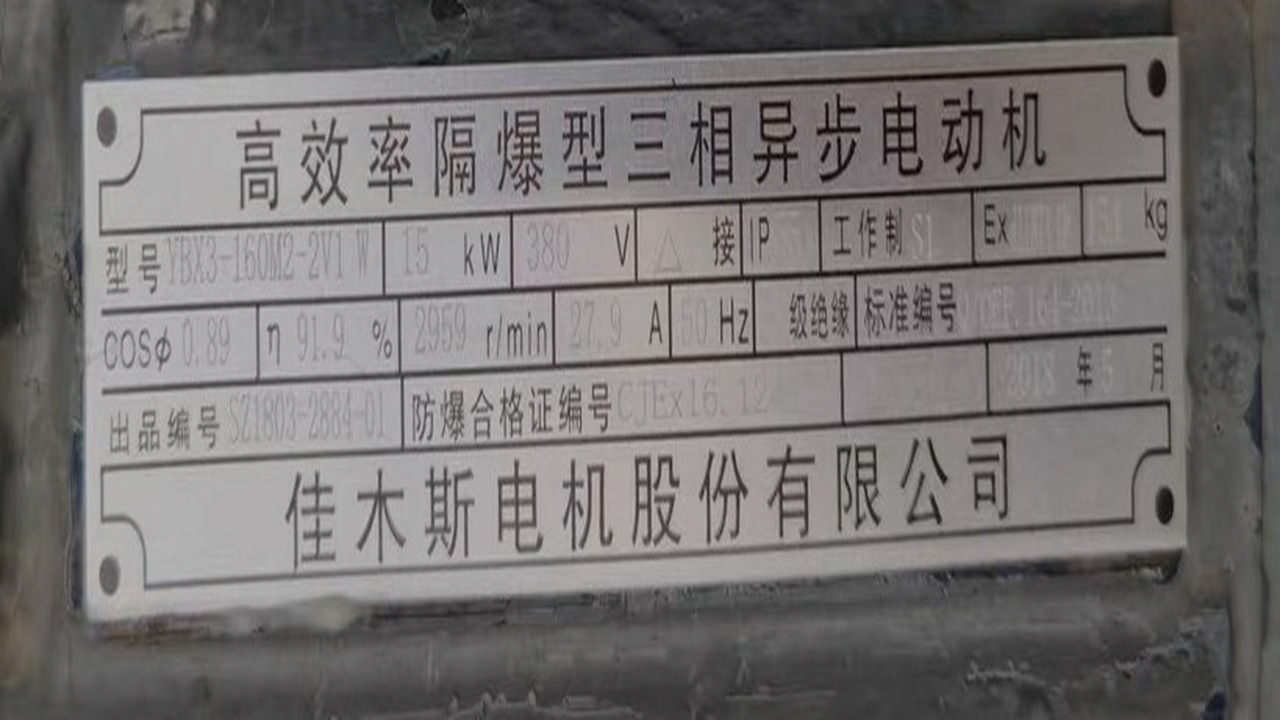

Supplement: S1 Dataset — (ZIP) [file pone.0300792.s001.zip › minimal data set/gt_img_0028_N1.0.jpg]

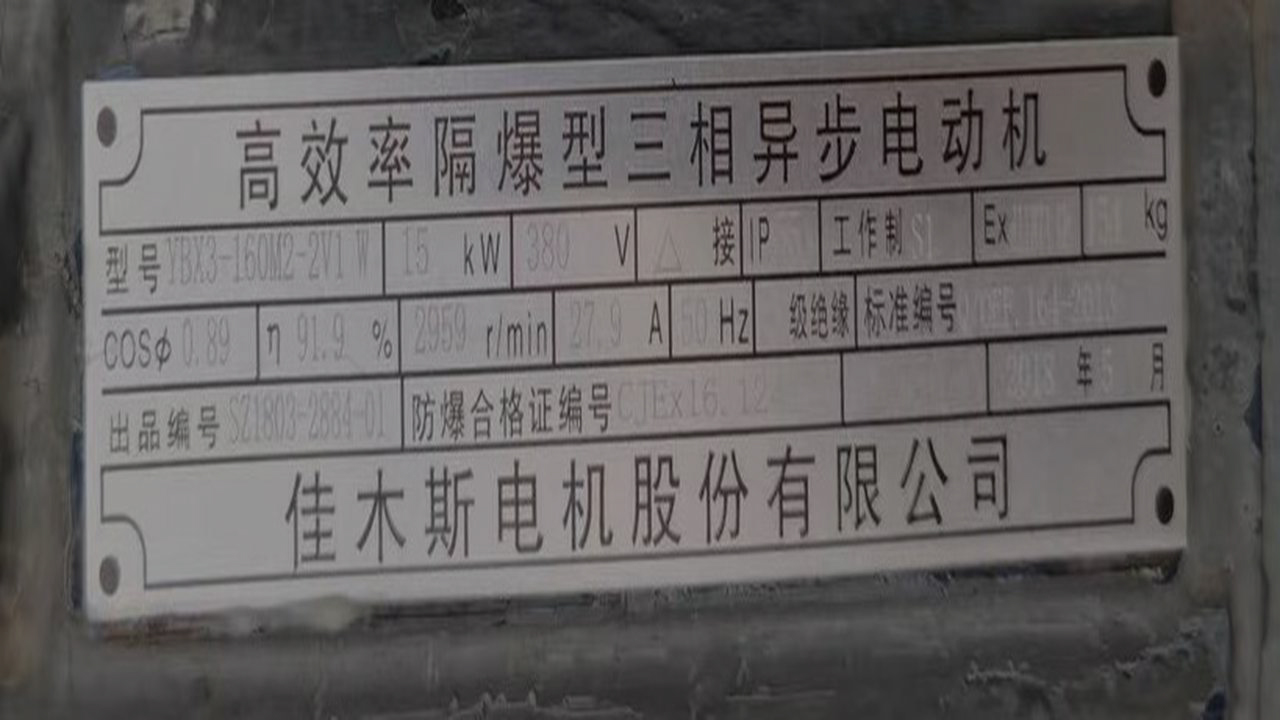

Supplement: S1 Dataset — (ZIP) [file pone.0300792.s001.zip › minimal data set/gt_img_0028_N1.5.jpg]

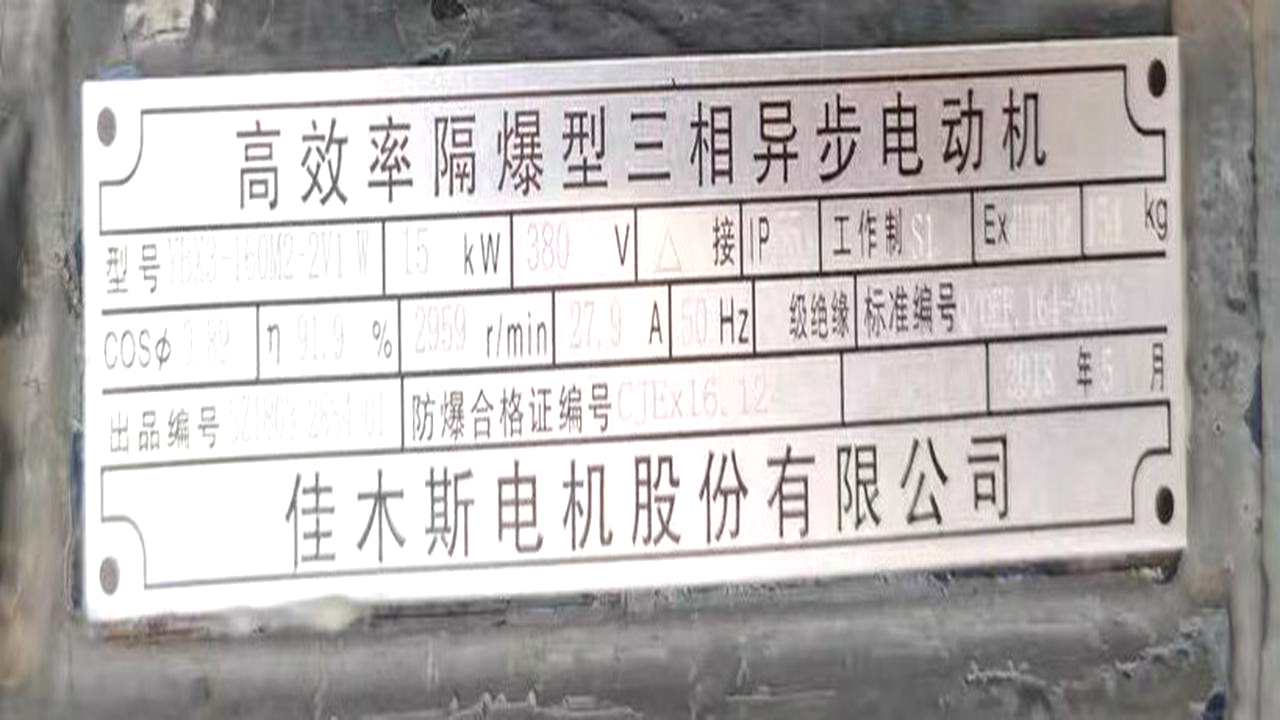

Supplement: S1 Dataset — (ZIP) [file pone.0300792.s001.zip › minimal data set/gt_img_0028_P1.0.jpg]

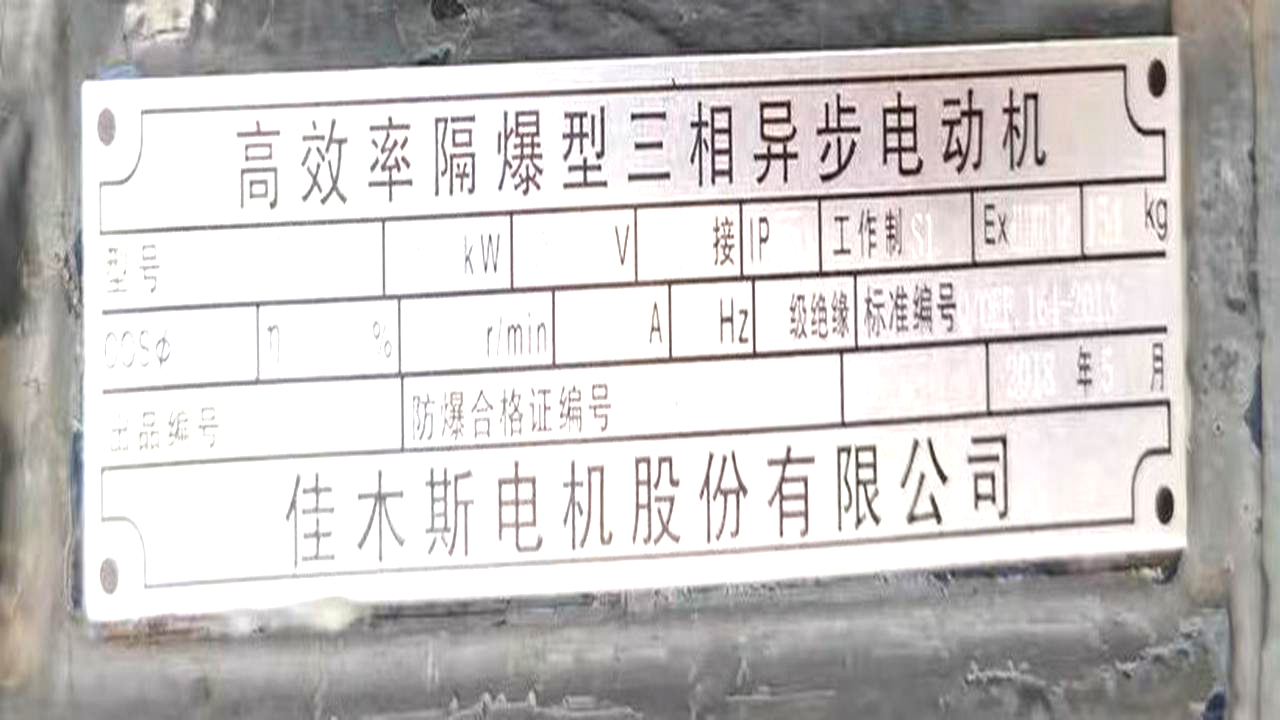

Supplement: S1 Dataset — (ZIP) [file pone.0300792.s001.zip › minimal data set/gt_img_0028_P1.5.jpg]

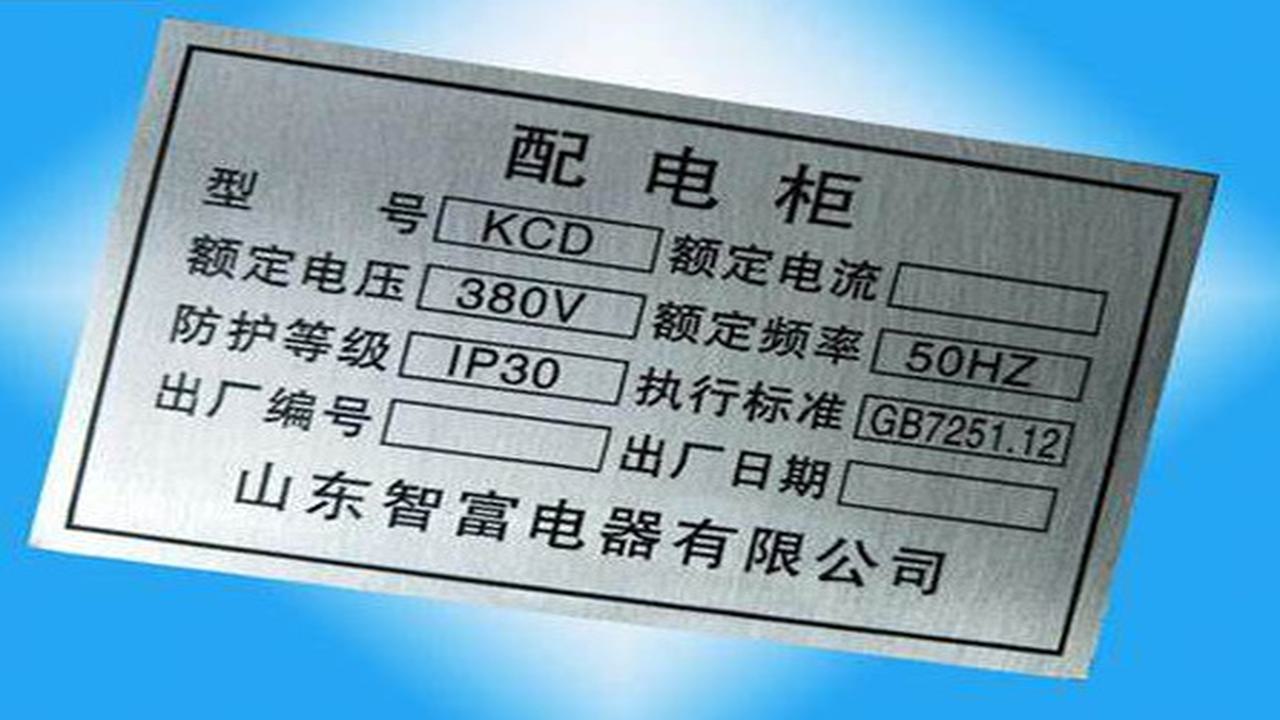

Supplement: S1 Dataset — (ZIP) [file pone.0300792.s001.zip › minimal data set/gt_img_0029_0.jpg]

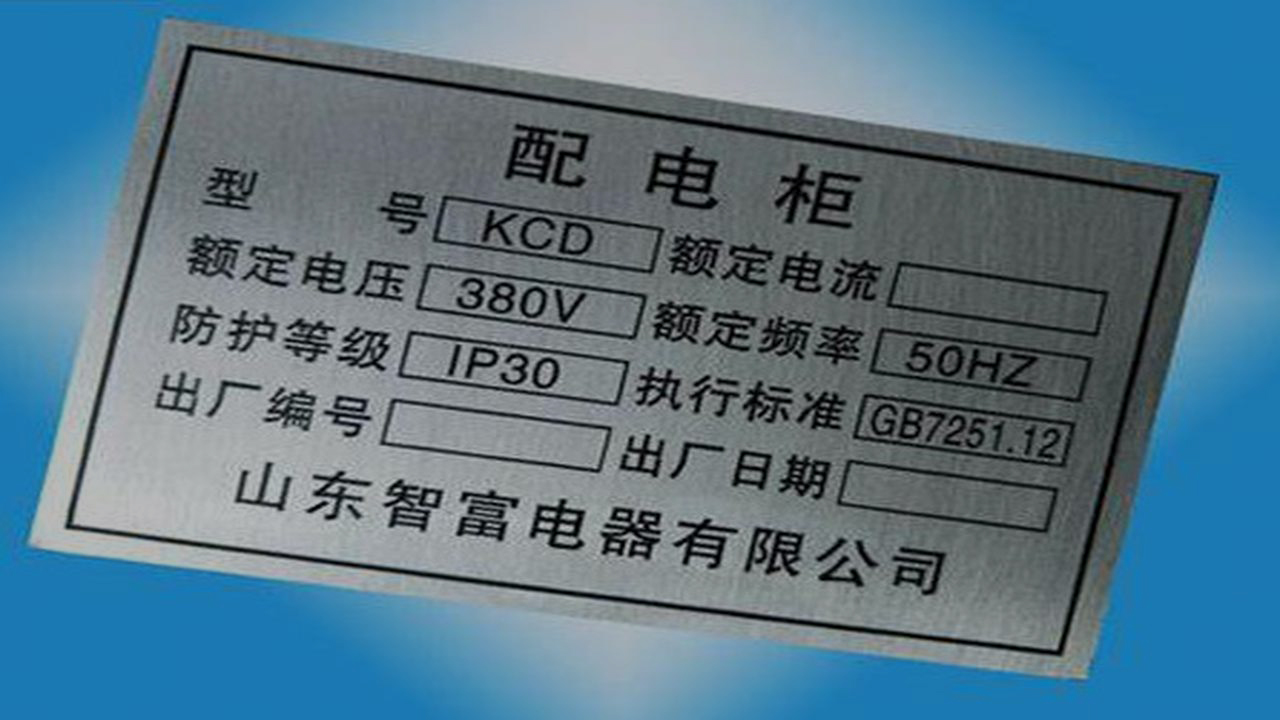

Supplement: S1 Dataset — (ZIP) [file pone.0300792.s001.zip › minimal data set/gt_img_0029_N1.0.jpg]

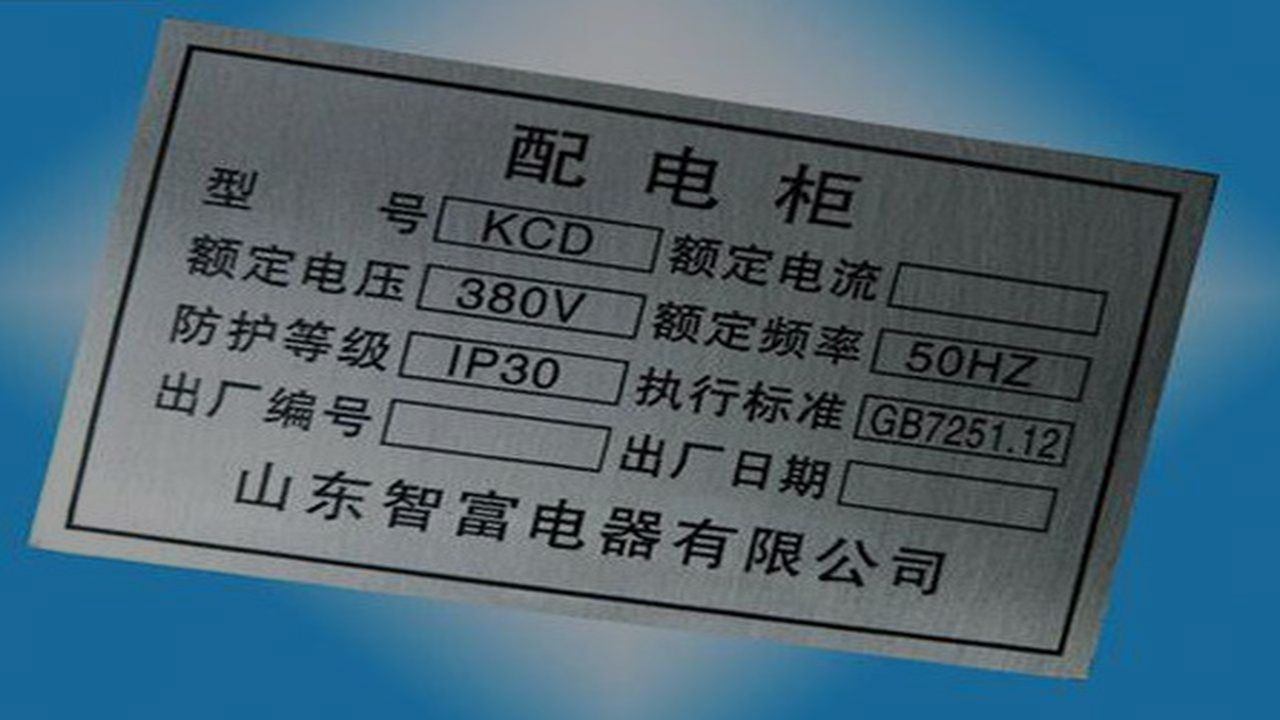

Supplement: S1 Dataset — (ZIP) [file pone.0300792.s001.zip › minimal data set/gt_img_0029_N1.5.jpg]

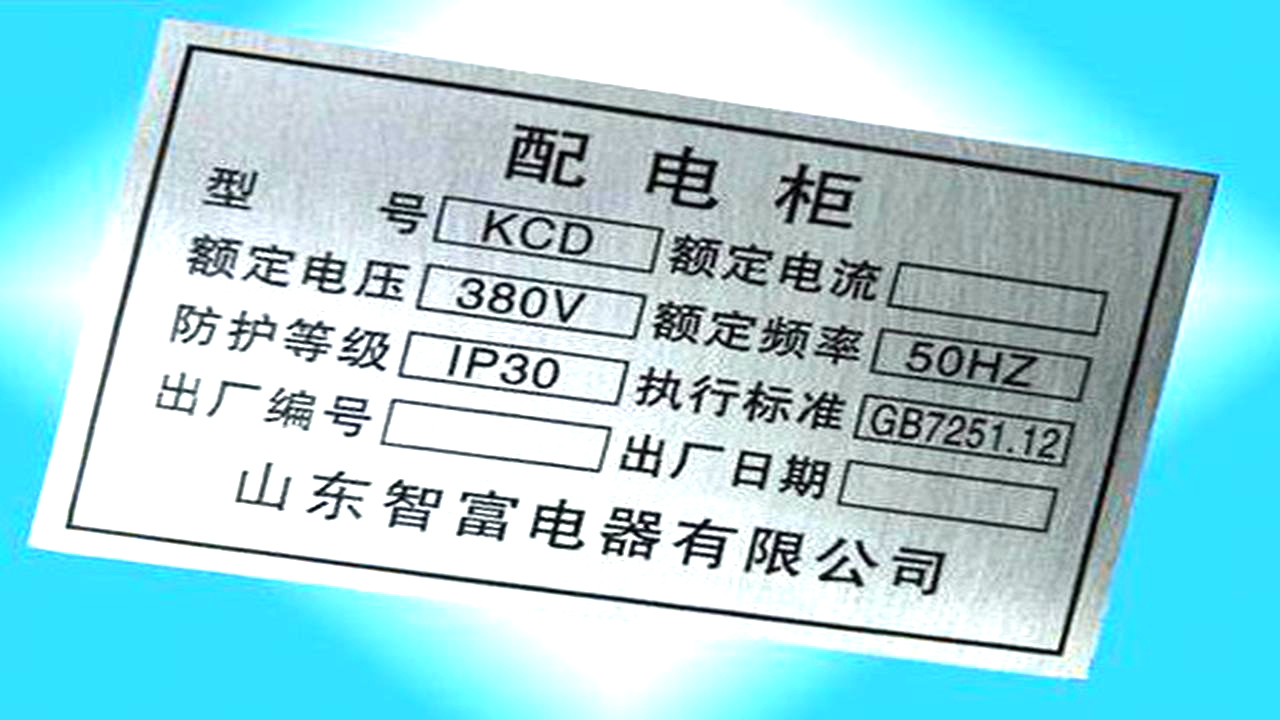

Supplement: S1 Dataset — (ZIP) [file pone.0300792.s001.zip › minimal data set/gt_img_0029_P1.0.jpg]

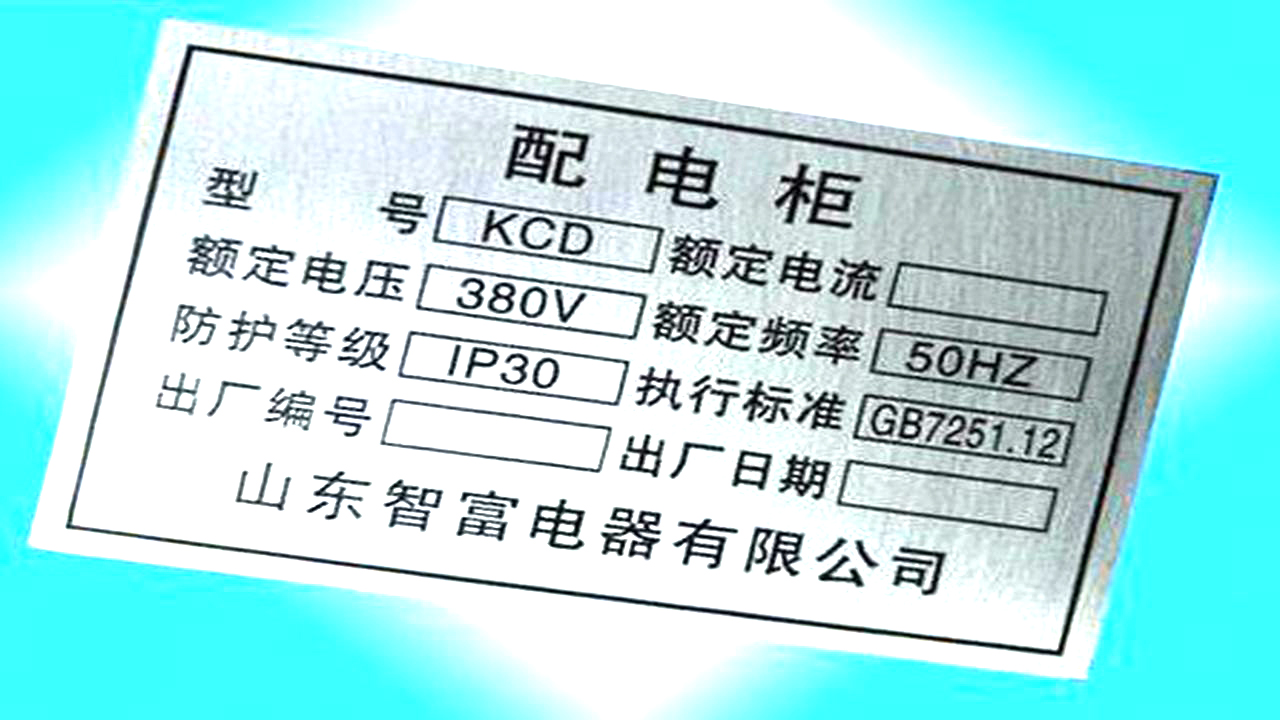

Supplement: S1 Dataset — (ZIP) [file pone.0300792.s001.zip › minimal data set/gt_img_0029_P1.5.jpg]

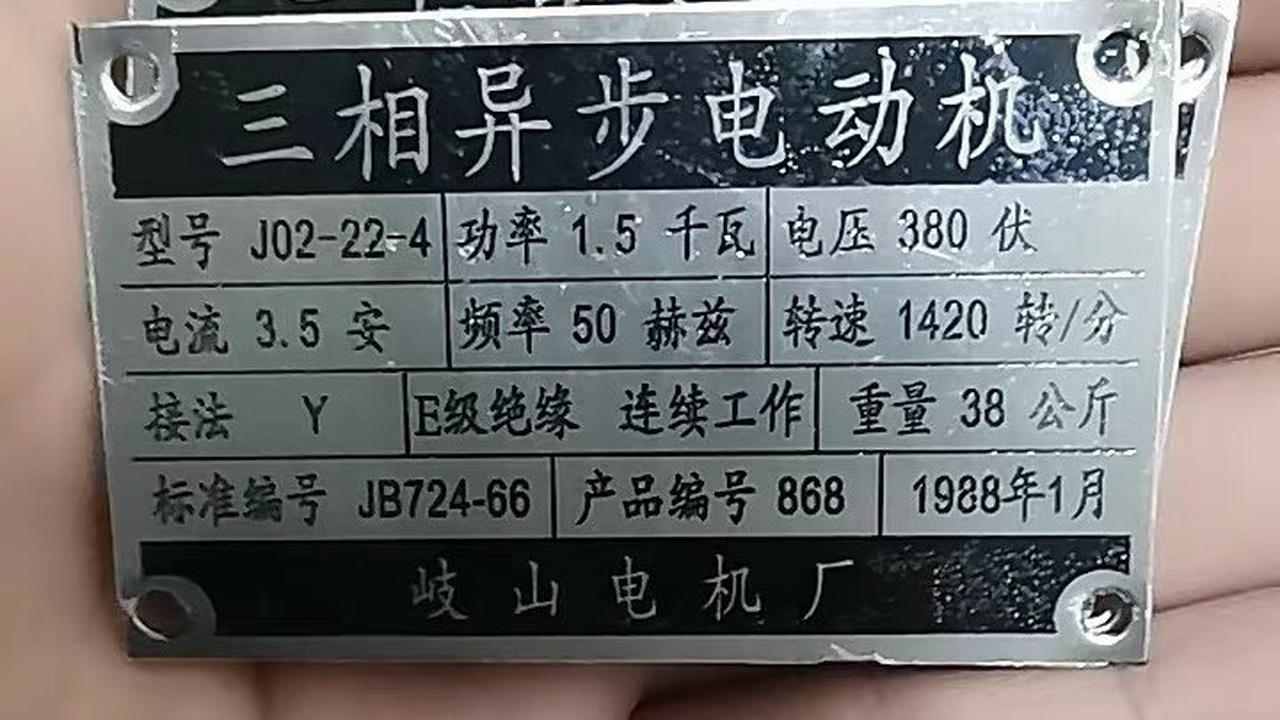

Supplement: S1 Dataset — (ZIP) [file pone.0300792.s001.zip › minimal data set/gt_img_0030_0.jpg]

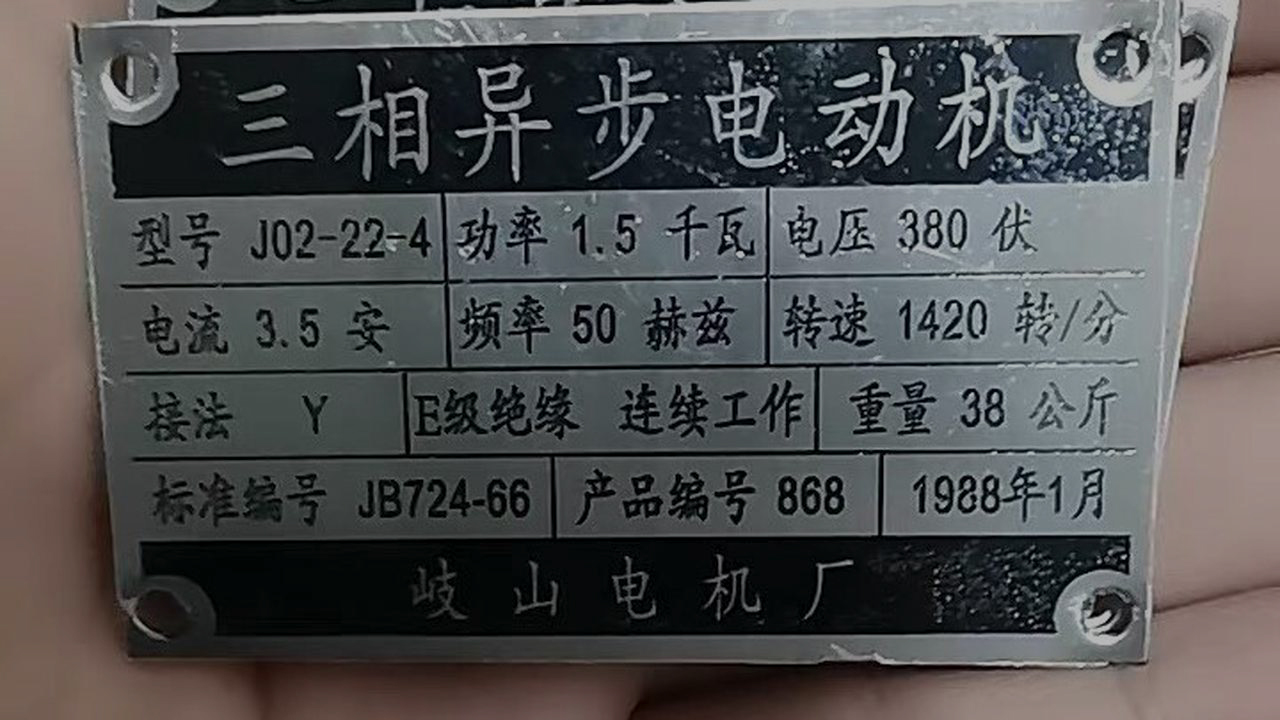

Supplement: S1 Dataset — (ZIP) [file pone.0300792.s001.zip › minimal data set/gt_img_0030_N1.0.jpg]

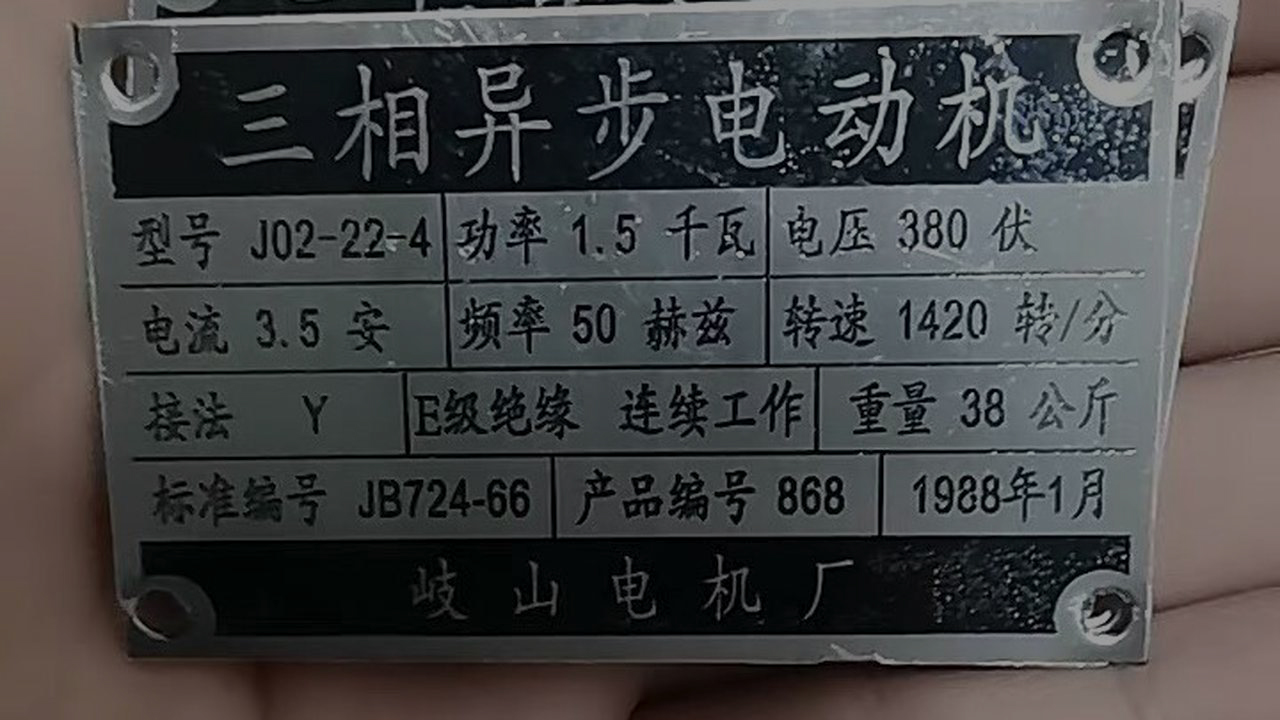

Supplement: S1 Dataset — (ZIP) [file pone.0300792.s001.zip › minimal data set/gt_img_0030_N1.5.jpg]

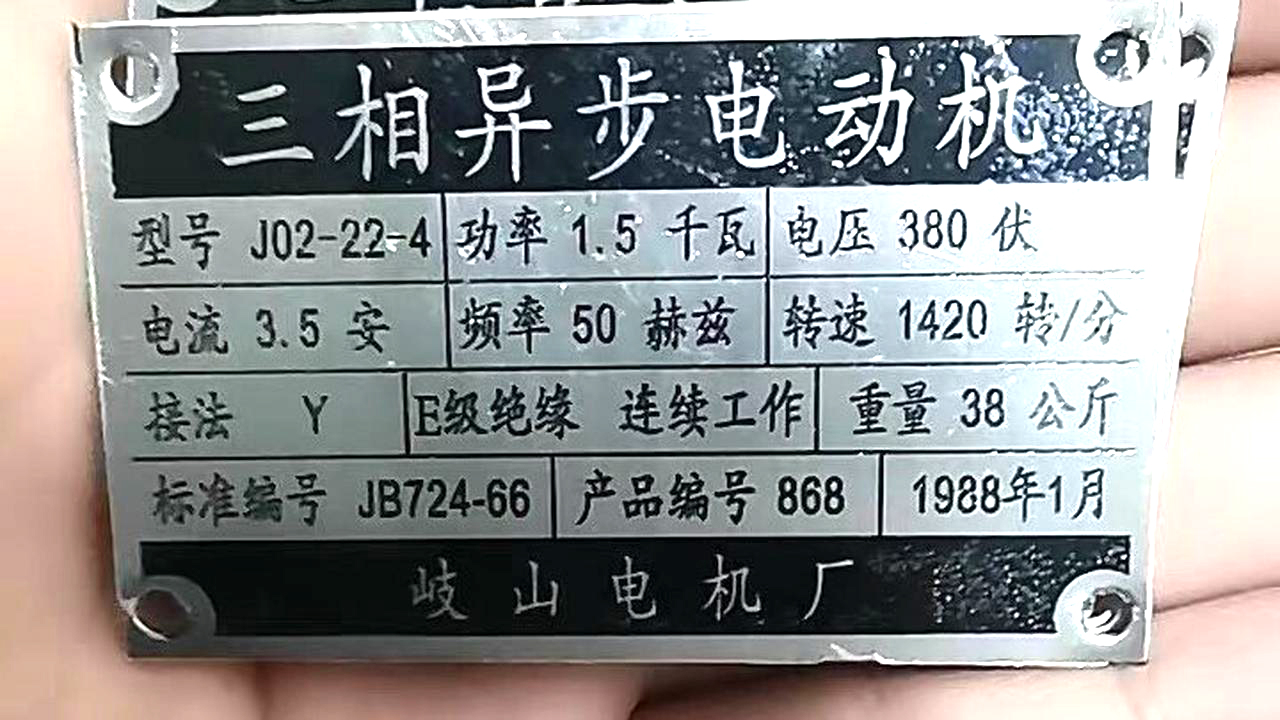

Supplement: S1 Dataset — (ZIP) [file pone.0300792.s001.zip › minimal data set/gt_img_0030_P1.0.jpg]

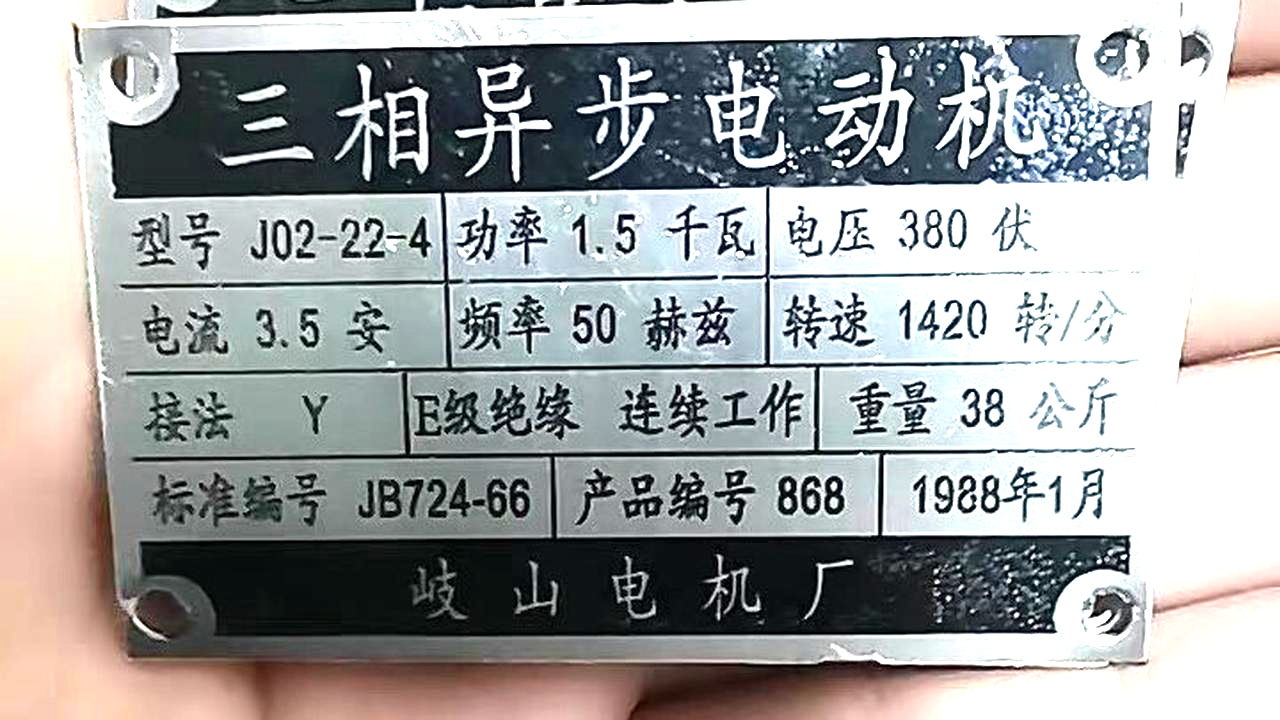

Supplement: S1 Dataset — (ZIP) [file pone.0300792.s001.zip › minimal data set/gt_img_0030_P1.5.jpg]

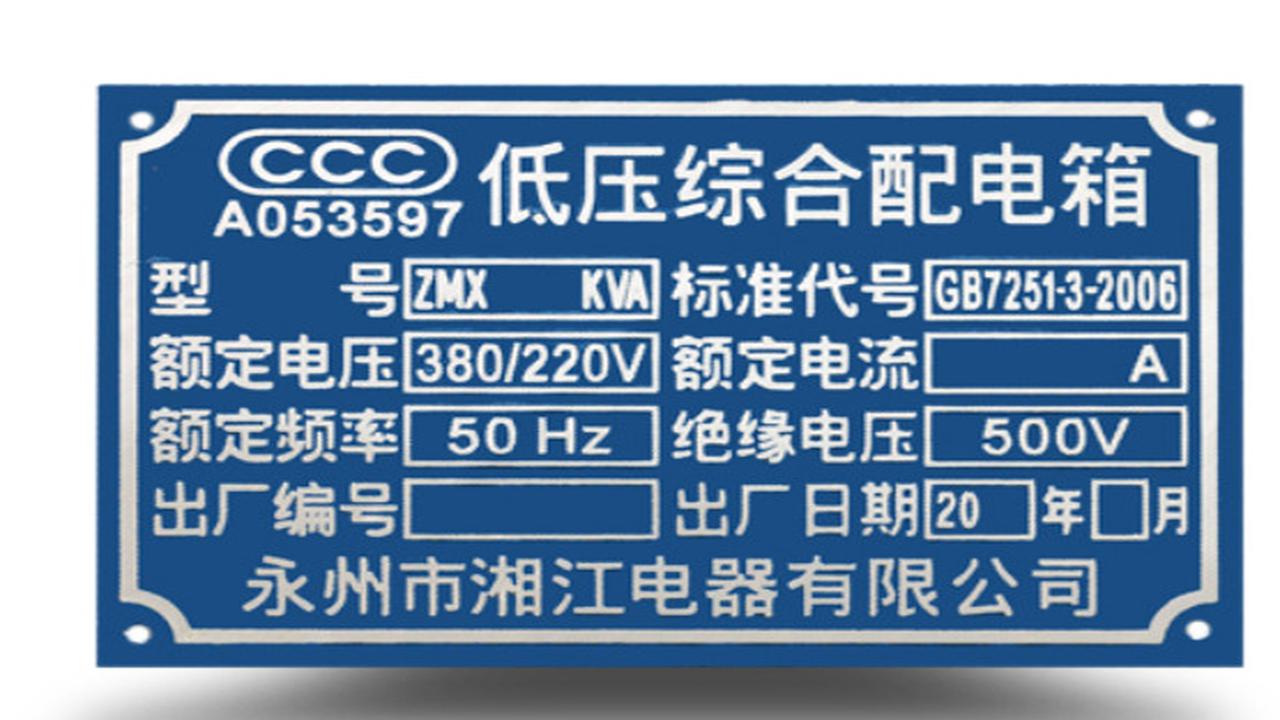

Supplement: S1 Dataset — (ZIP) [file pone.0300792.s001.zip › minimal data set/gt_img_0031_0.jpg]

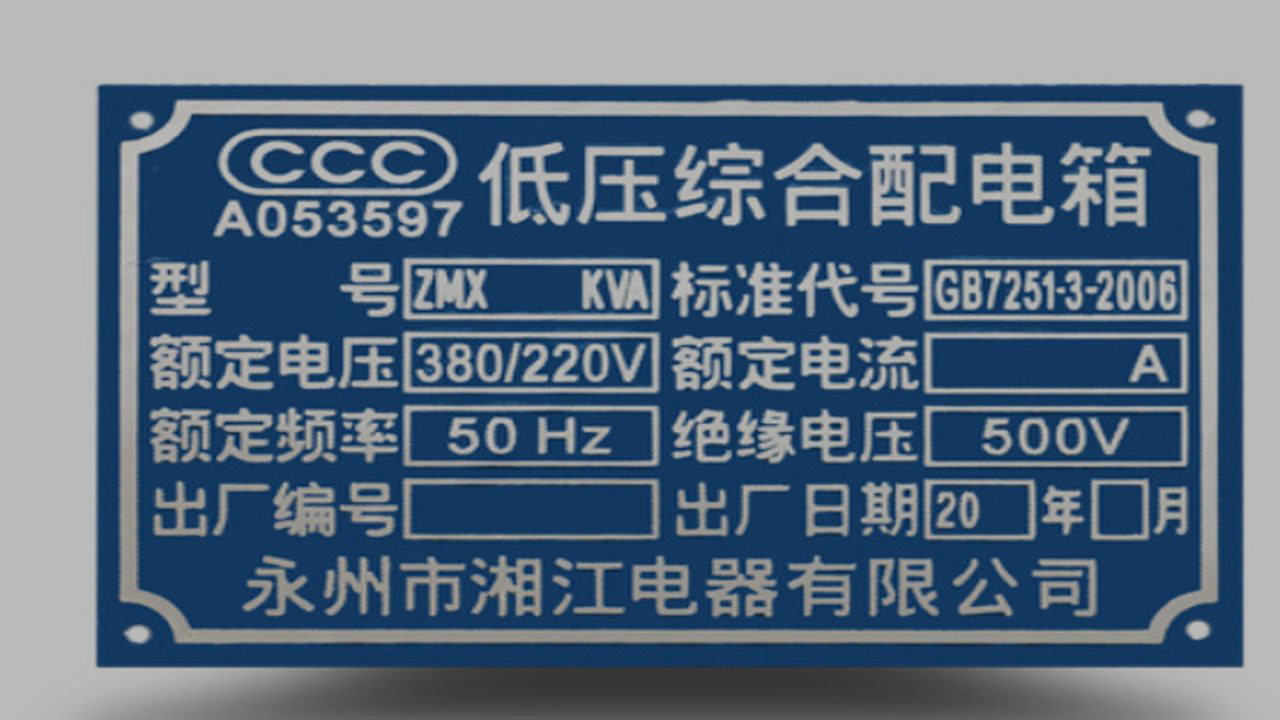

Supplement: S1 Dataset — (ZIP) [file pone.0300792.s001.zip › minimal data set/gt_img_0031_N1.0.jpg]

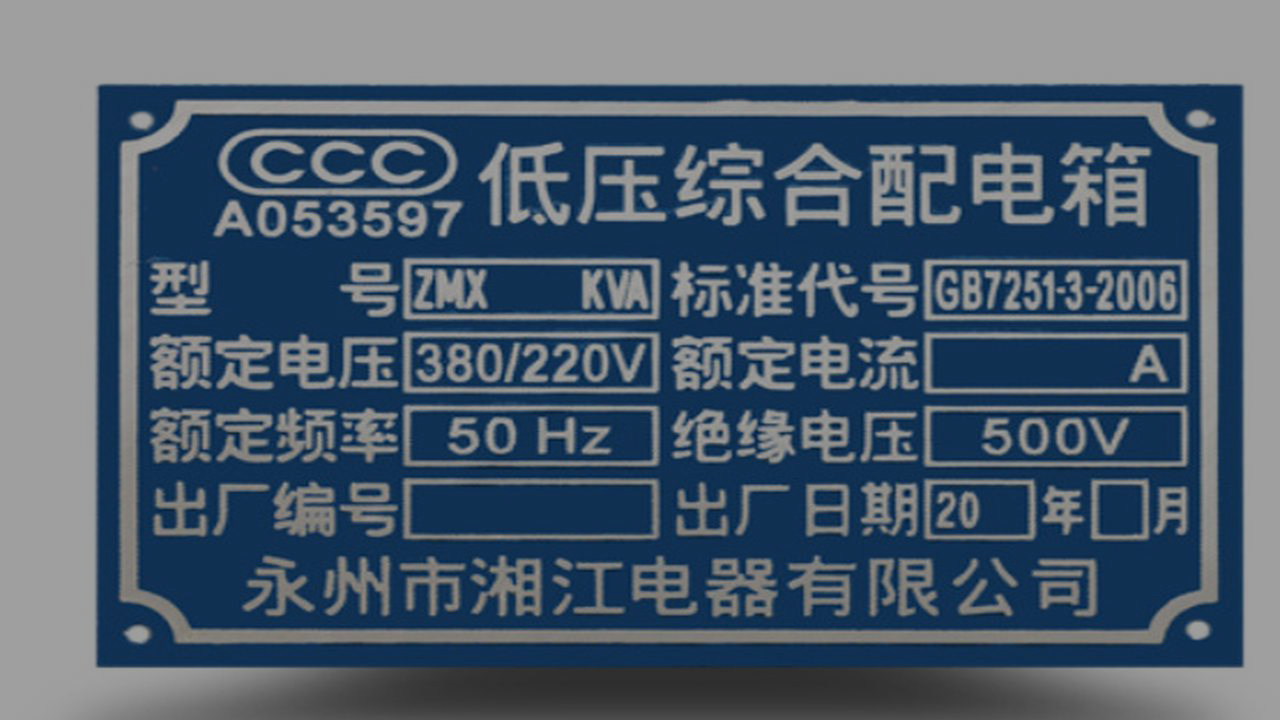

Supplement: S1 Dataset — (ZIP) [file pone.0300792.s001.zip › minimal data set/gt_img_0031_N1.5.jpg]

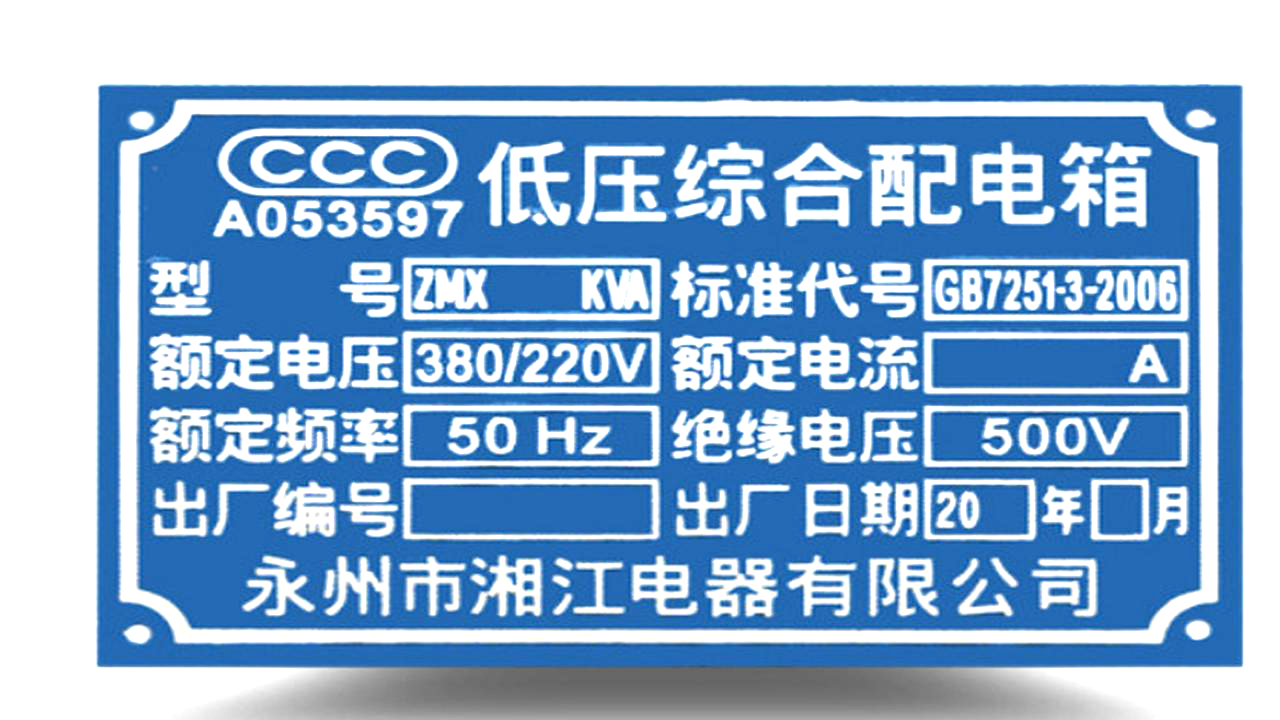

Supplement: S1 Dataset — (ZIP) [file pone.0300792.s001.zip › minimal data set/gt_img_0031_P1.0.jpg]

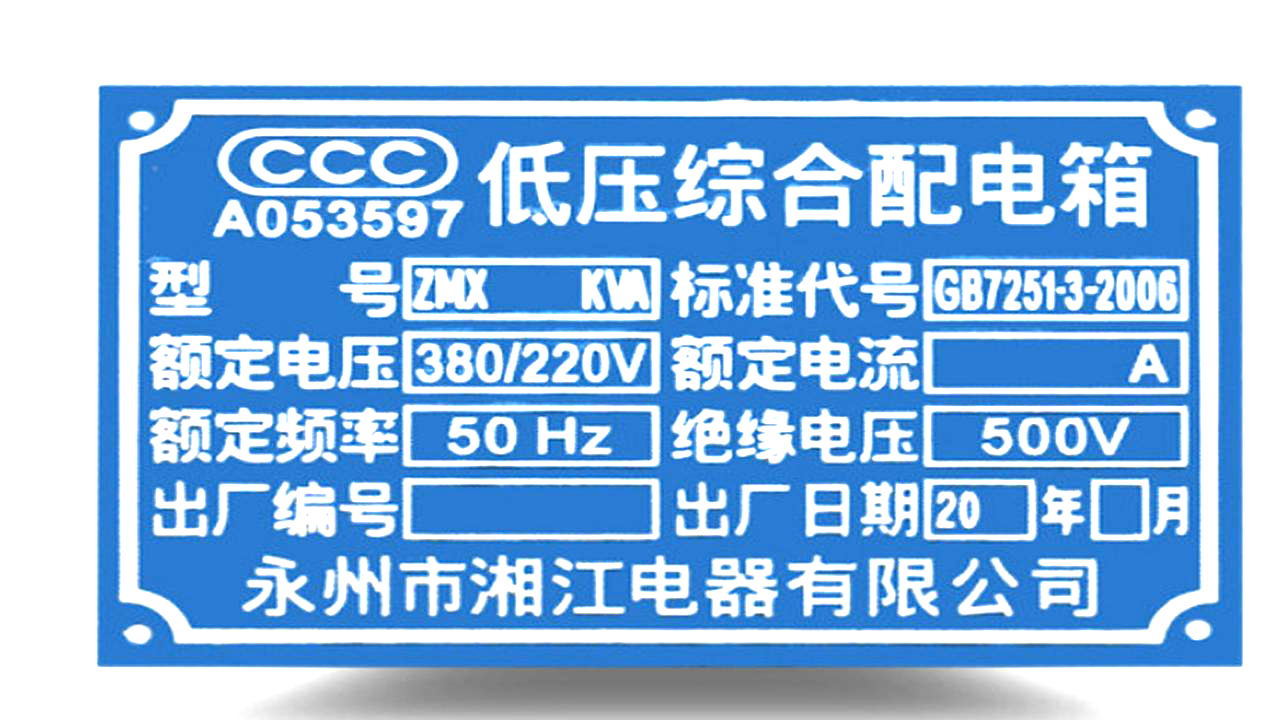

Supplement: S1 Dataset — (ZIP) [file pone.0300792.s001.zip › minimal data set/gt_img_0031_P1.5.jpg]

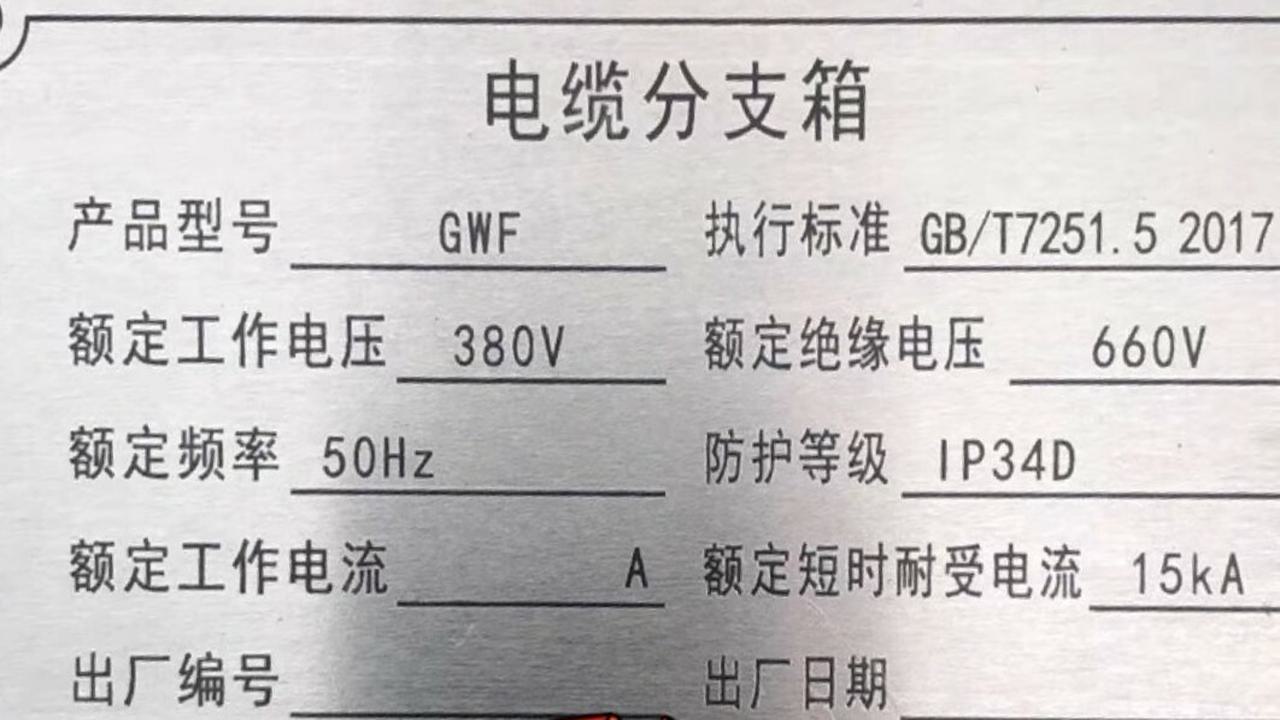

Supplement: S1 Dataset — (ZIP) [file pone.0300792.s001.zip › minimal data set/gt_img_0032_0.jpg]

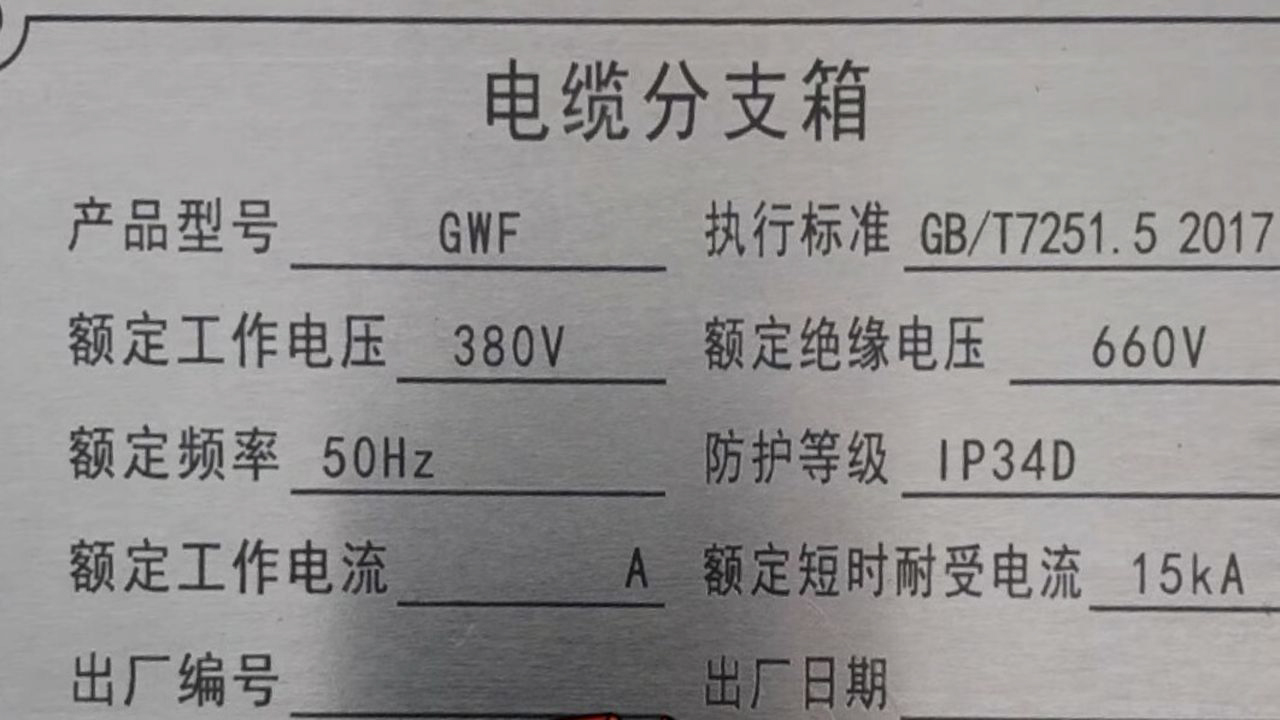

Supplement: S1 Dataset — (ZIP) [file pone.0300792.s001.zip › minimal data set/gt_img_0032_N1.0.jpg]

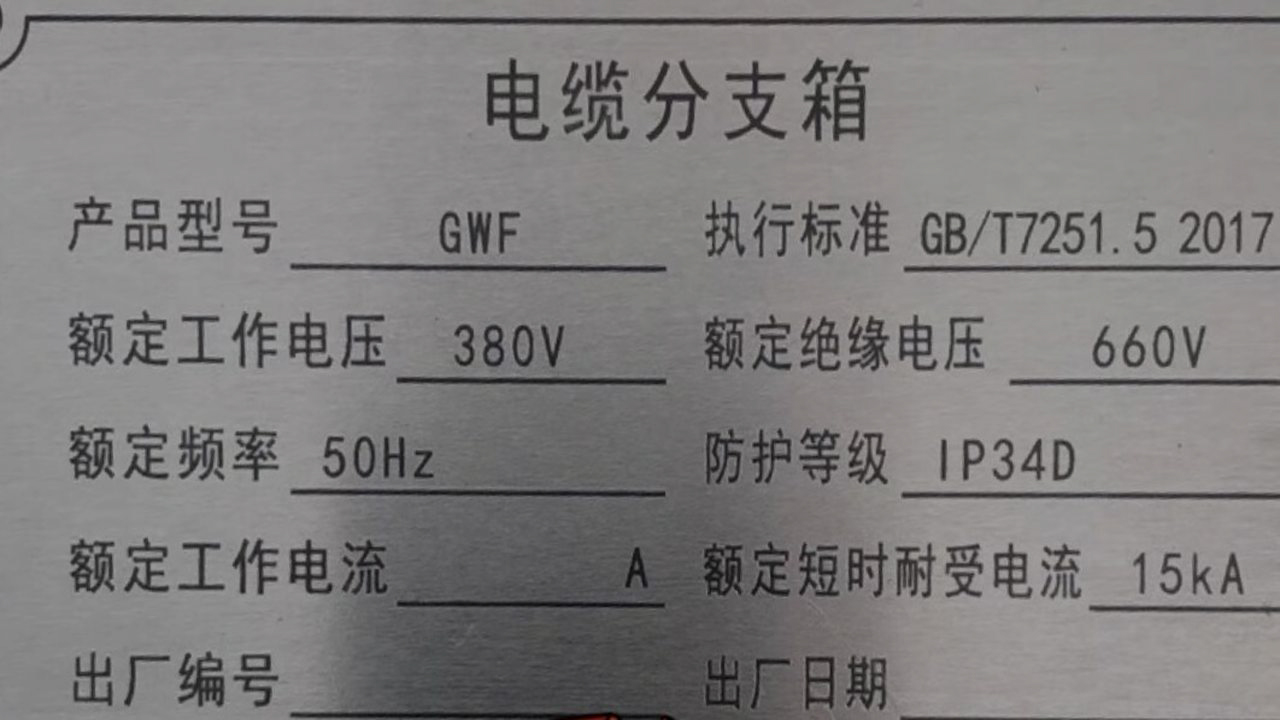

Supplement: S1 Dataset — (ZIP) [file pone.0300792.s001.zip › minimal data set/gt_img_0032_N1.5.jpg]

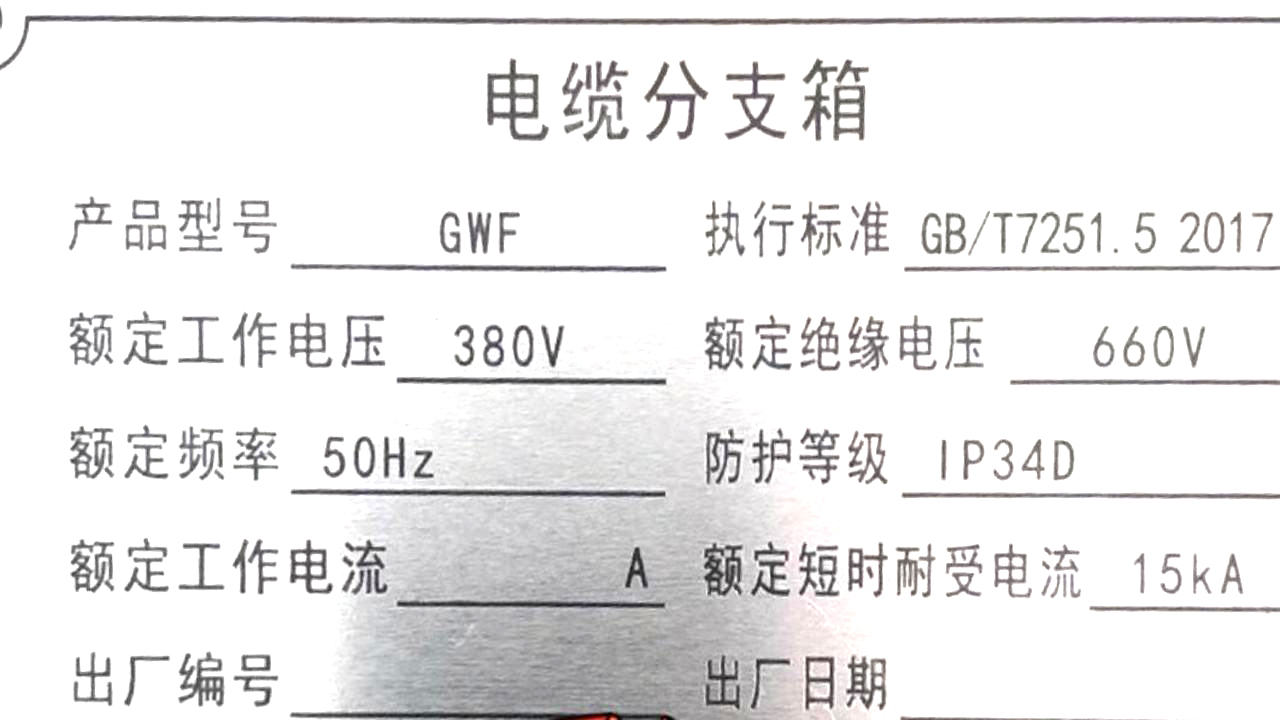

Supplement: S1 Dataset — (ZIP) [file pone.0300792.s001.zip › minimal data set/gt_img_0032_P1.0.jpg]

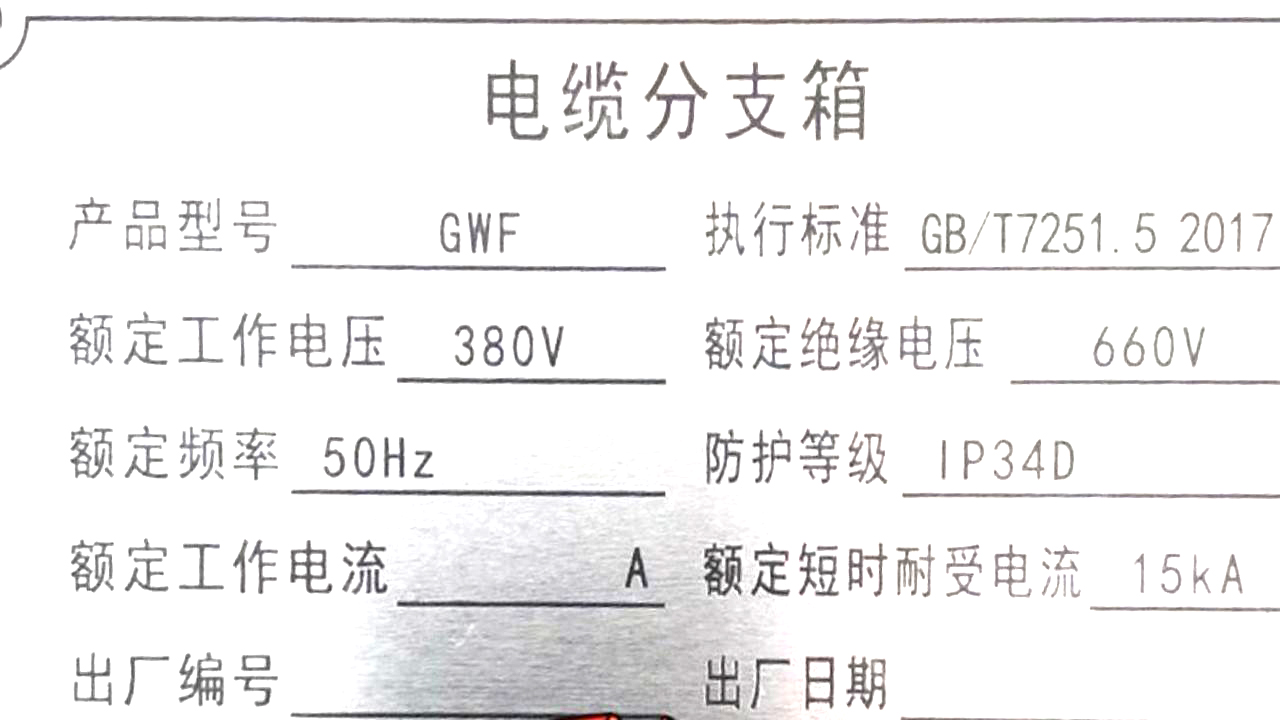

Supplement: S1 Dataset — (ZIP) [file pone.0300792.s001.zip › minimal data set/gt_img_0032_P1.5.jpg]

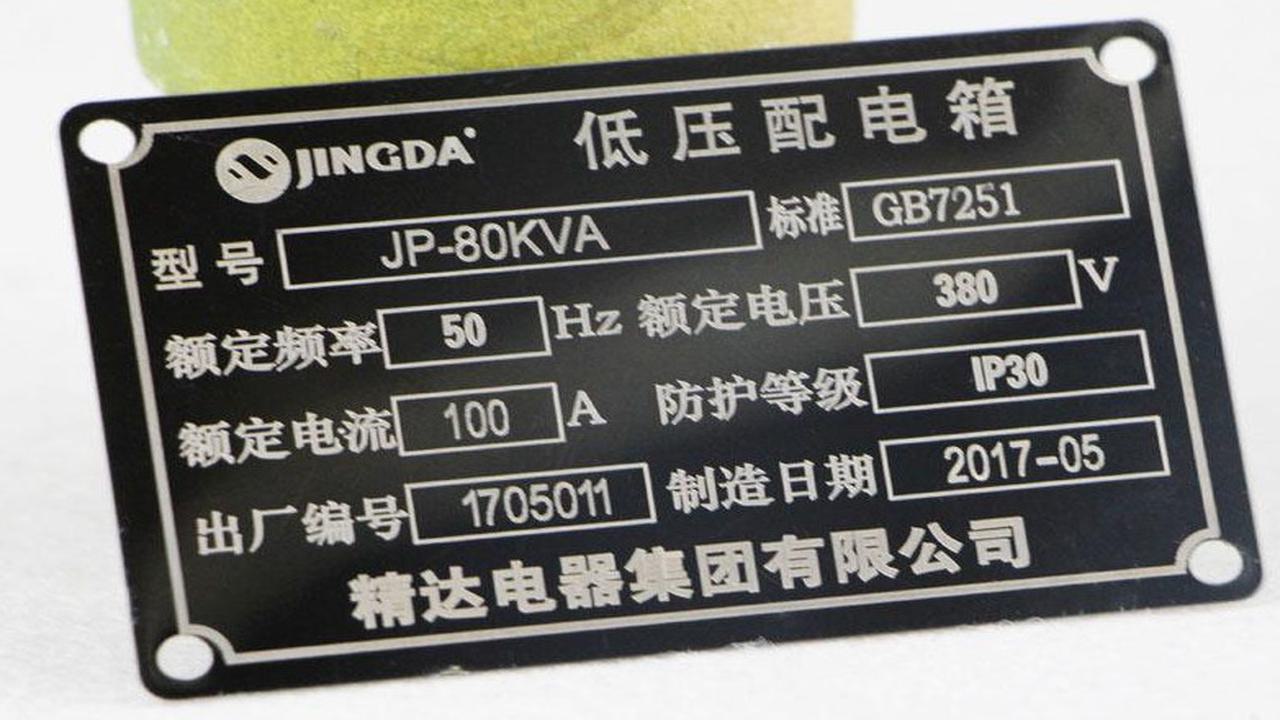

Supplement: S1 Dataset — (ZIP) [file pone.0300792.s001.zip › minimal data set/gt_img_0033_0.jpg]

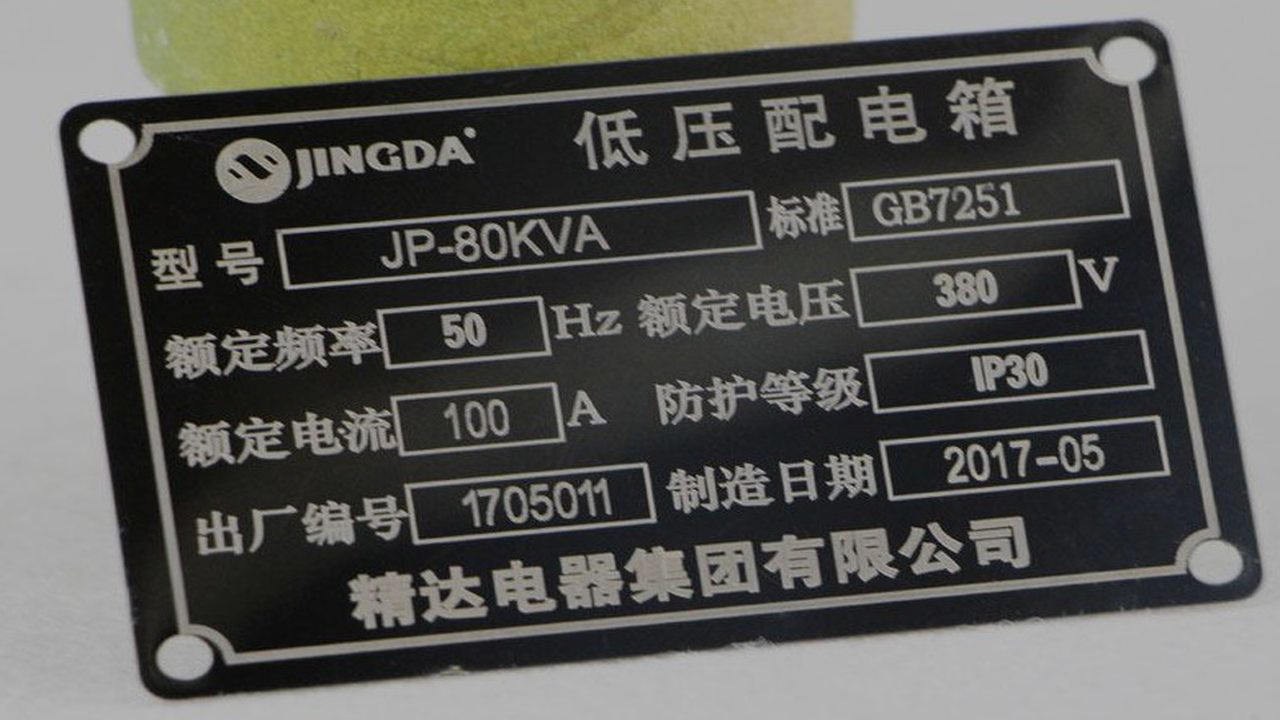

Supplement: S1 Dataset — (ZIP) [file pone.0300792.s001.zip › minimal data set/gt_img_0033_N1.0.jpg]

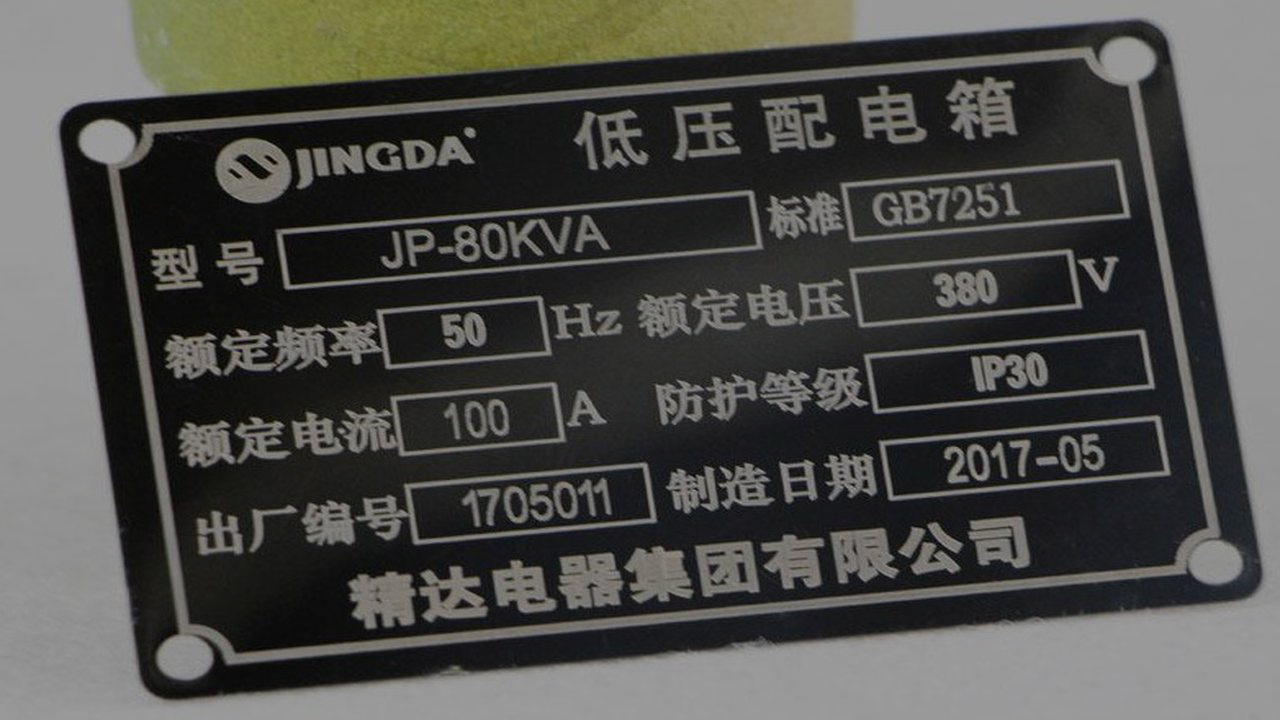

Supplement: S1 Dataset — (ZIP) [file pone.0300792.s001.zip › minimal data set/gt_img_0033_N1.5.jpg]

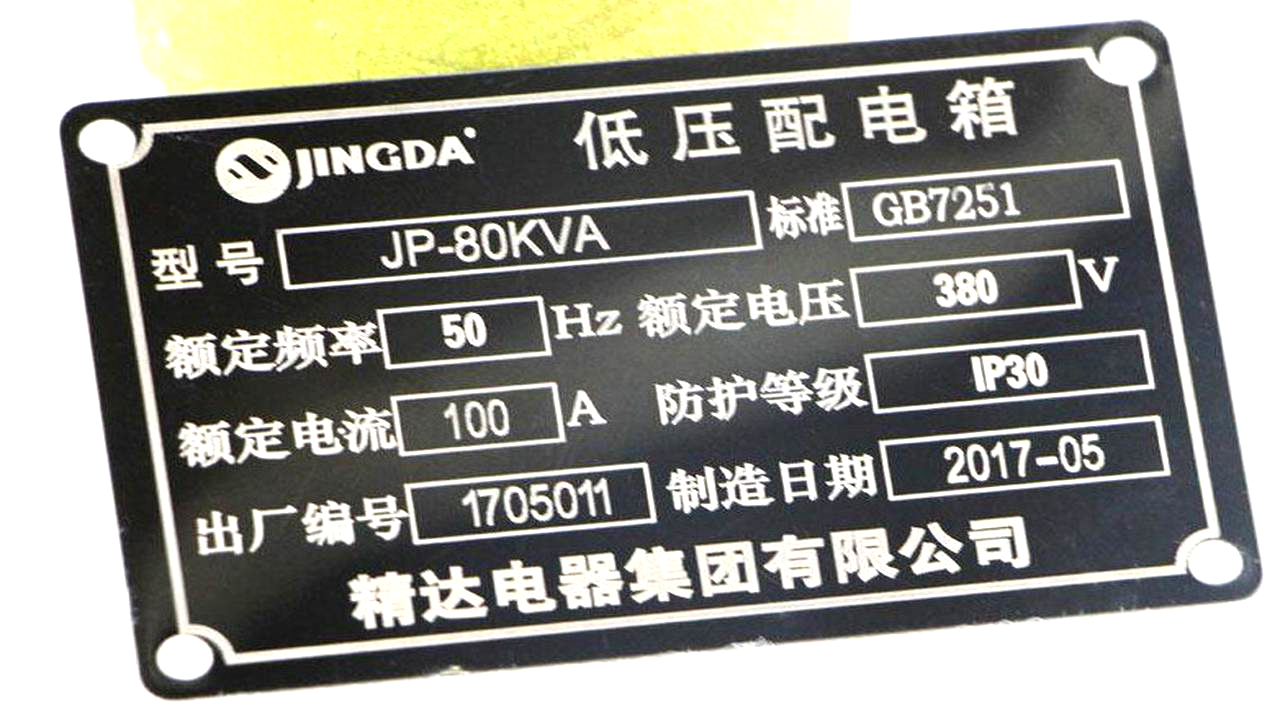

Supplement: S1 Dataset — (ZIP) [file pone.0300792.s001.zip › minimal data set/gt_img_0033_P1.0.jpg]

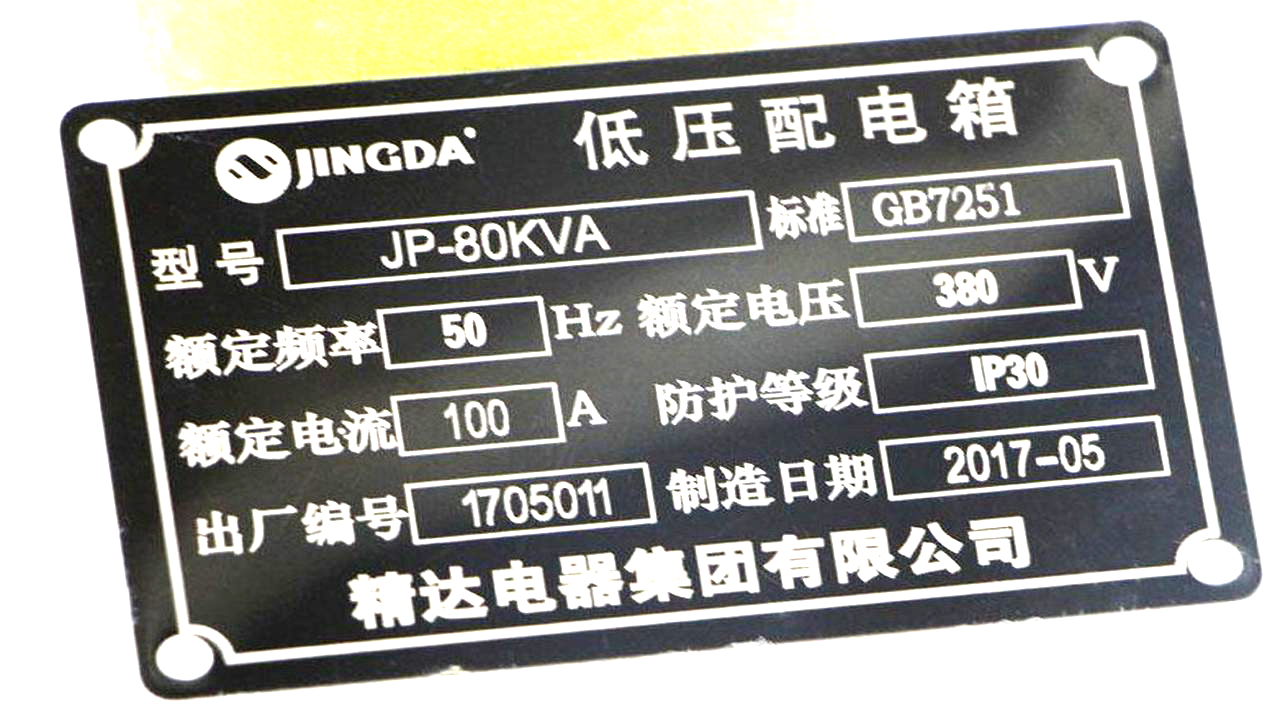

Supplement: S1 Dataset — (ZIP) [file pone.0300792.s001.zip › minimal data set/gt_img_0033_P1.5.jpg]

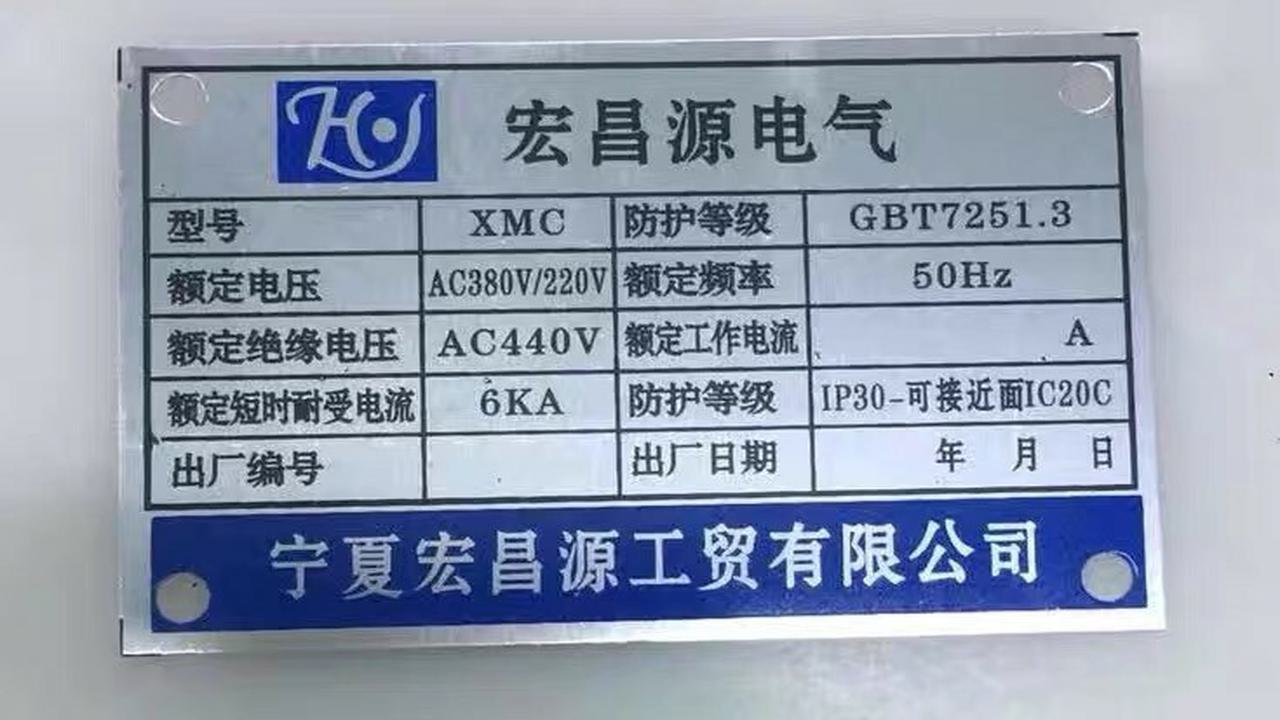

Supplement: S1 Dataset — (ZIP) [file pone.0300792.s001.zip › minimal data set/gt_img_0034_0.jpg]

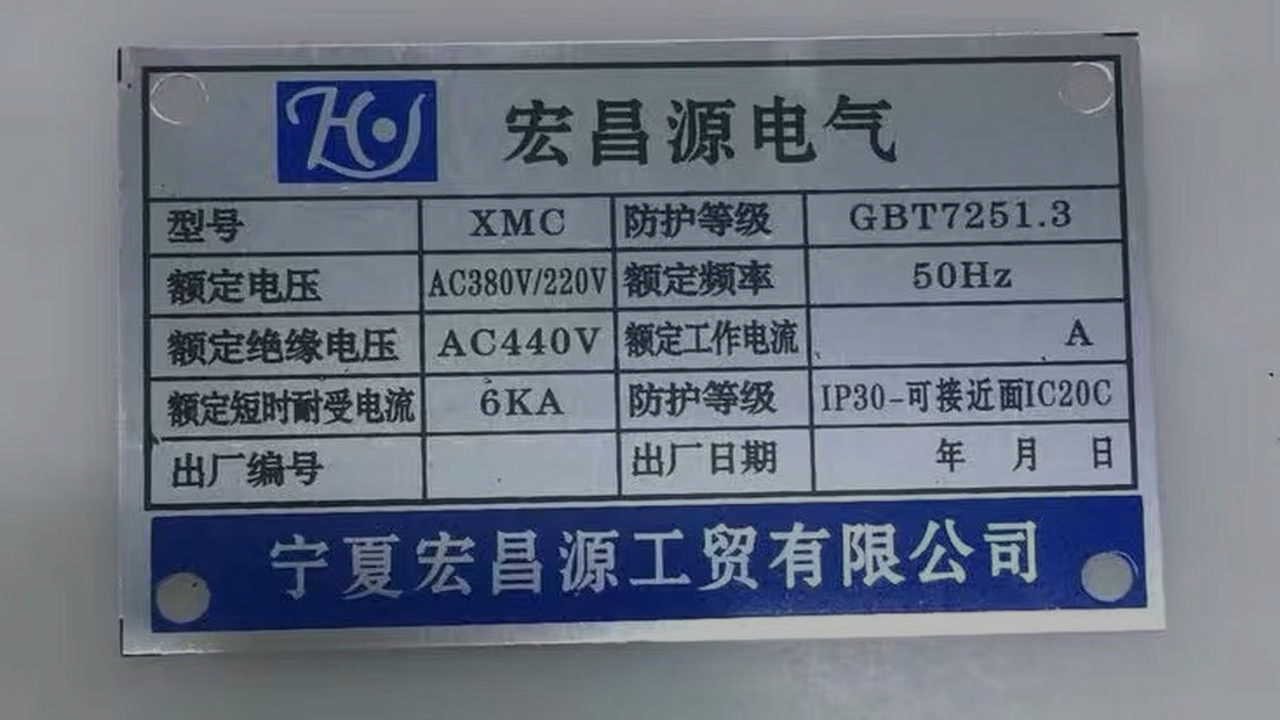

Supplement: S1 Dataset — (ZIP) [file pone.0300792.s001.zip › minimal data set/gt_img_0034_N1.0.jpg]

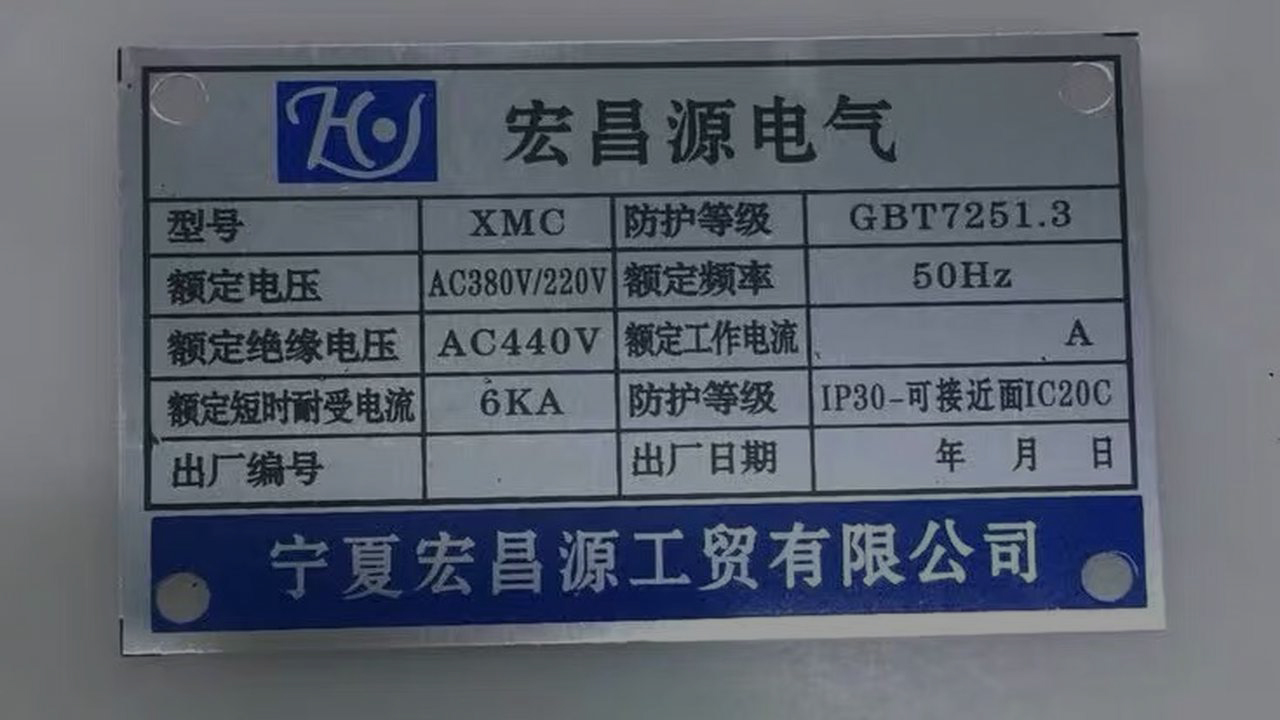

Supplement: S1 Dataset — (ZIP) [file pone.0300792.s001.zip › minimal data set/gt_img_0034_N1.5.jpg]

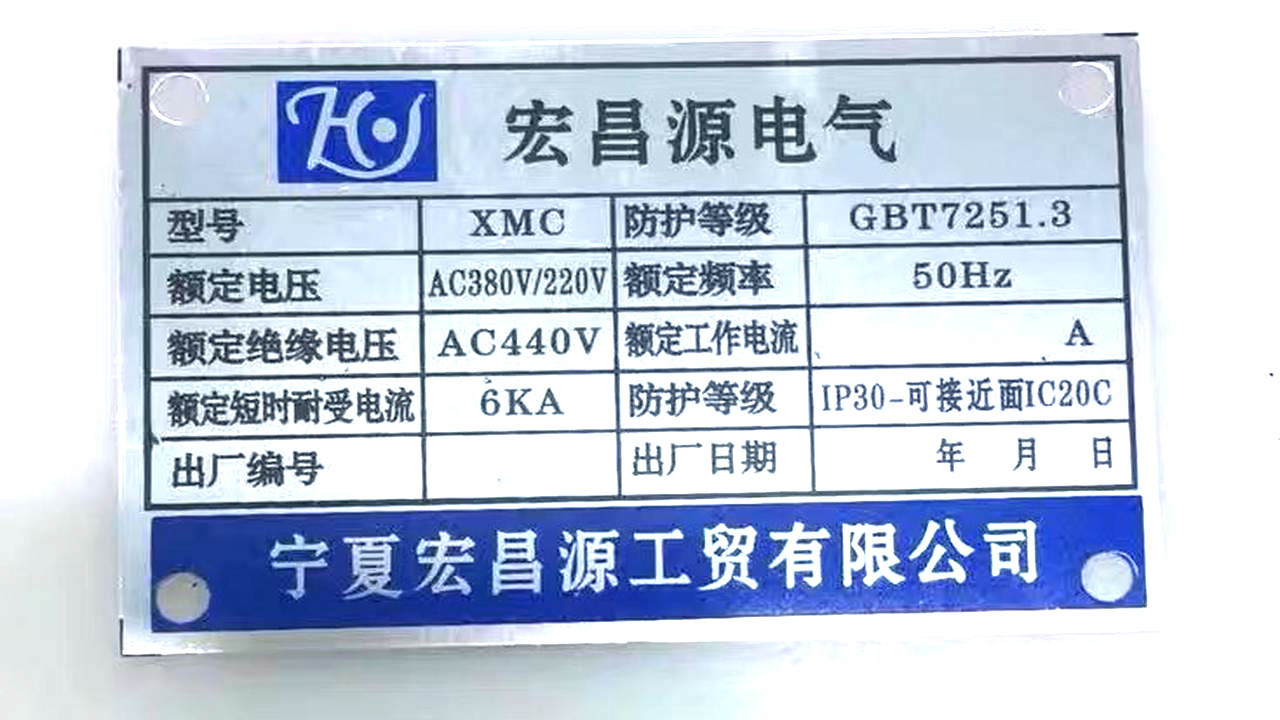

Supplement: S1 Dataset — (ZIP) [file pone.0300792.s001.zip › minimal data set/gt_img_0034_P1.0.jpg]

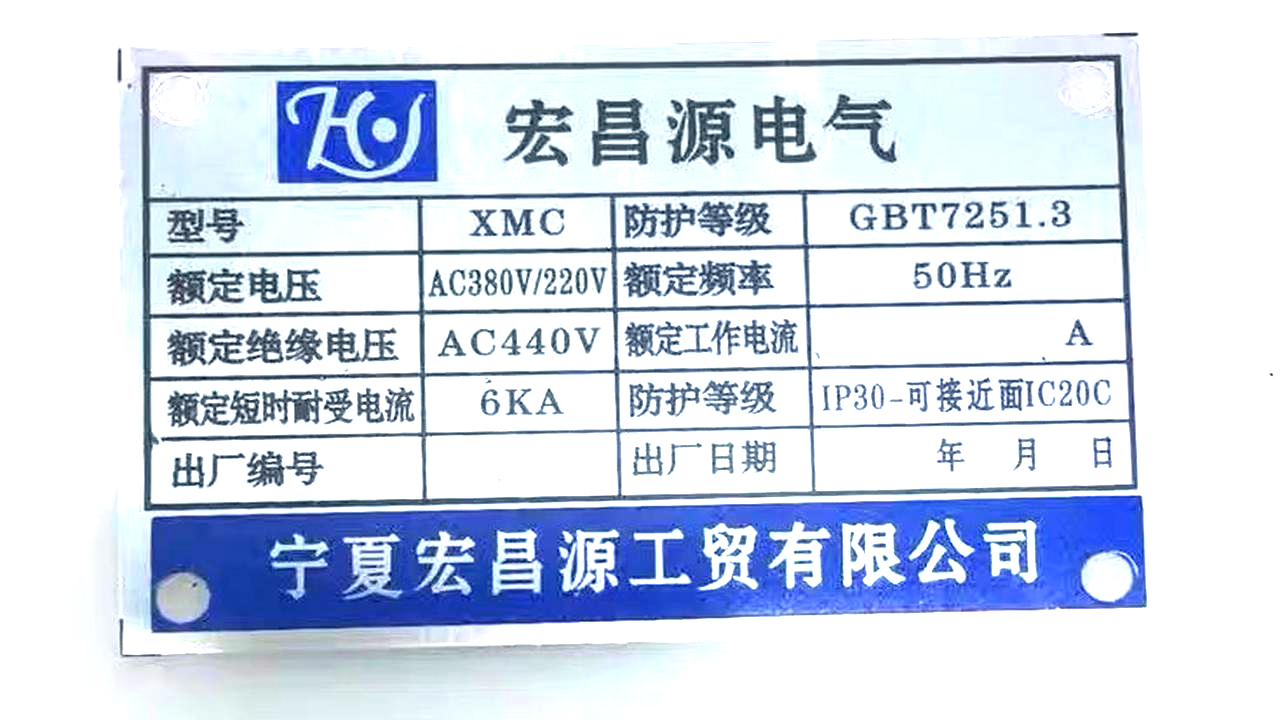

Supplement: S1 Dataset — (ZIP) [file pone.0300792.s001.zip › minimal data set/gt_img_0034_P1.5.jpg]

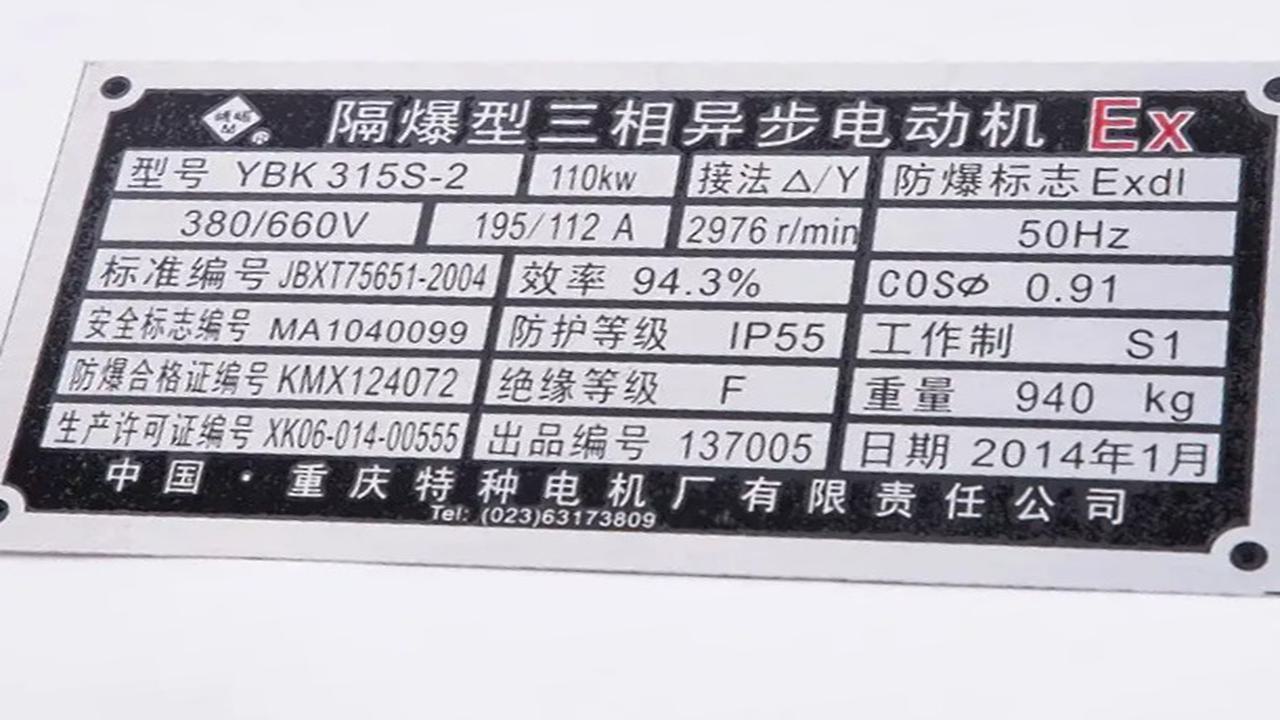

Supplement: S1 Dataset — (ZIP) [file pone.0300792.s001.zip › minimal data set/gt_img_0035_0.jpg]

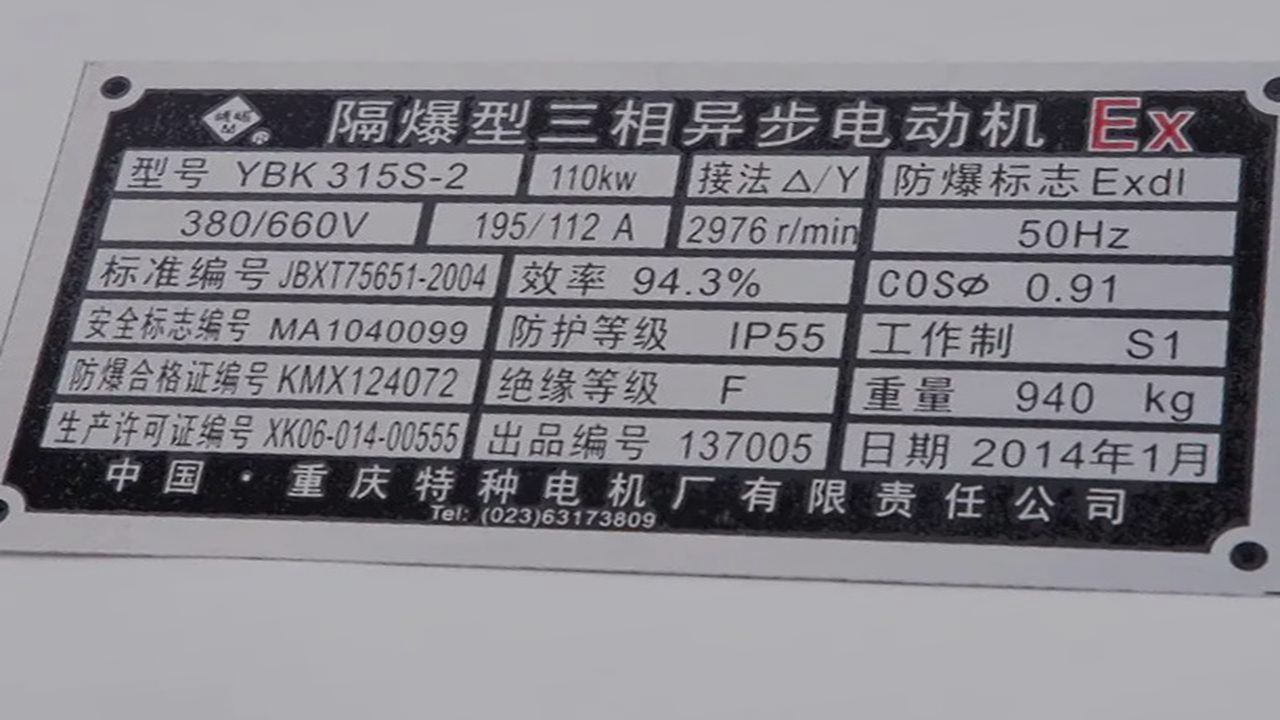

Supplement: S1 Dataset — (ZIP) [file pone.0300792.s001.zip › minimal data set/gt_img_0035_N1.0.jpg]

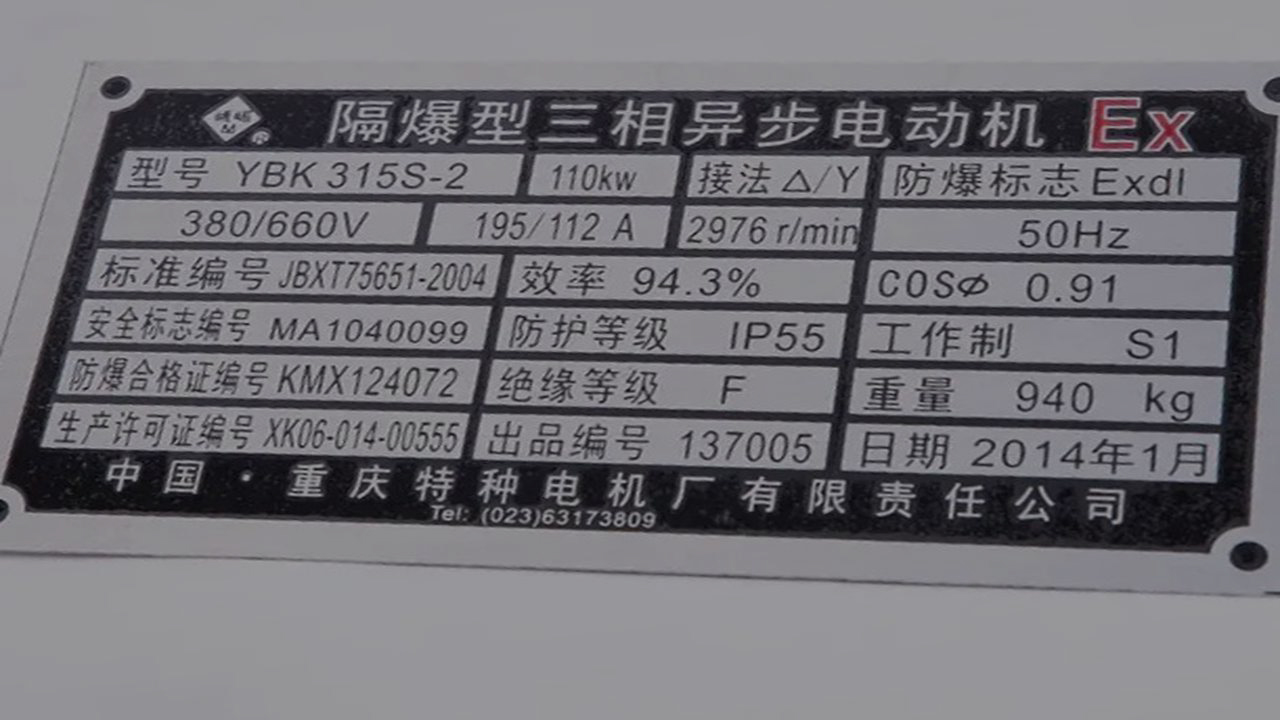

Supplement: S1 Dataset — (ZIP) [file pone.0300792.s001.zip › minimal data set/gt_img_0035_N1.5.jpg]

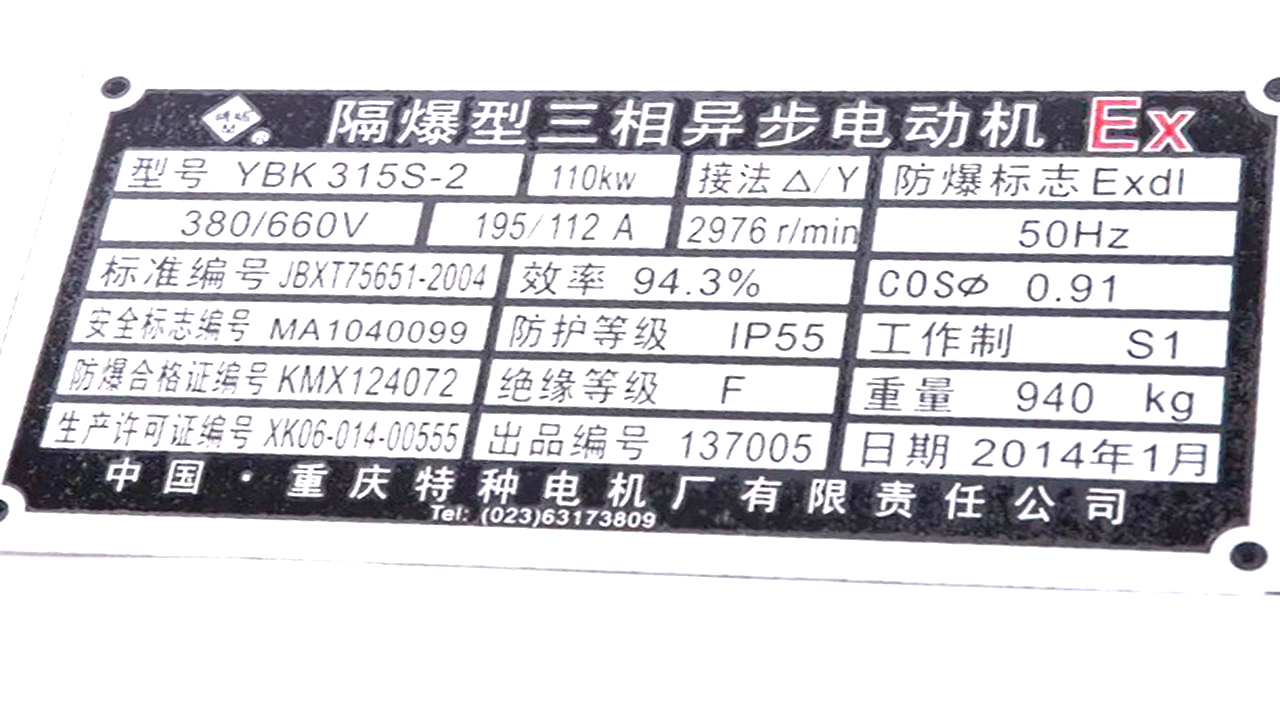

Supplement: S1 Dataset — (ZIP) [file pone.0300792.s001.zip › minimal data set/gt_img_0035_P1.0.jpg]

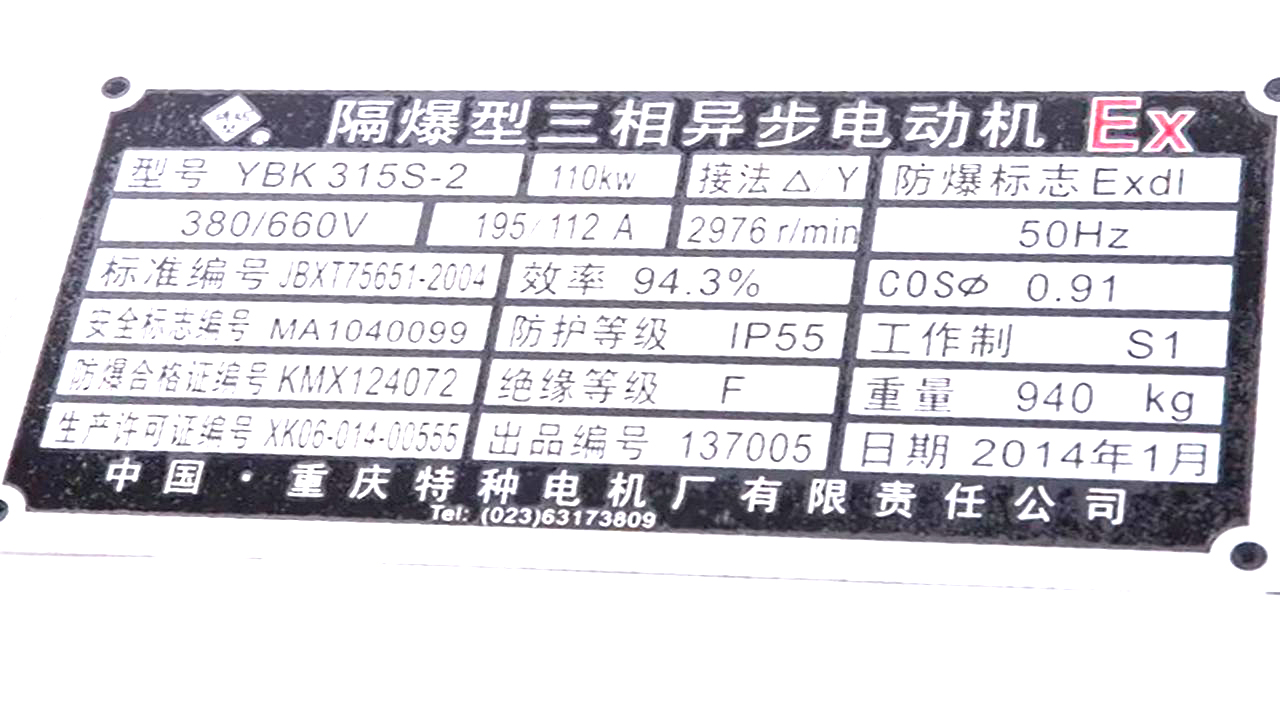

Supplement: S1 Dataset — (ZIP) [file pone.0300792.s001.zip › minimal data set/gt_img_0035_P1.5.jpg]

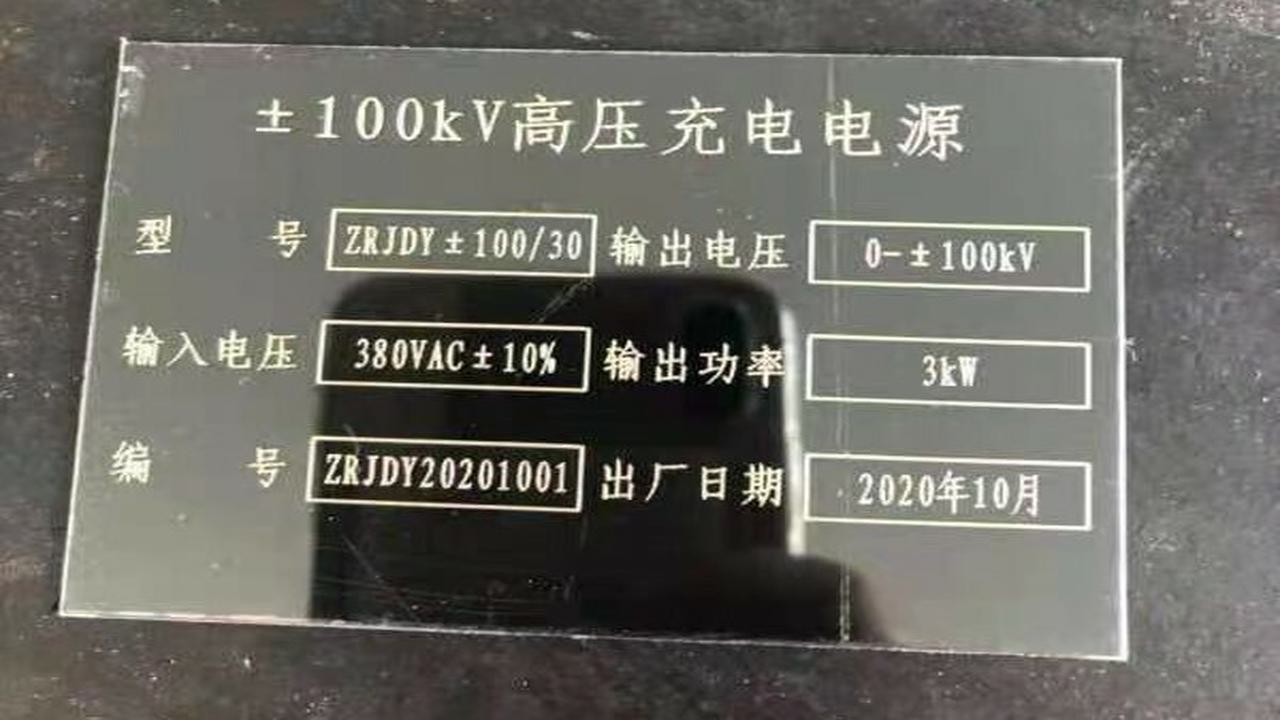

Supplement: S1 Dataset — (ZIP) [file pone.0300792.s001.zip › minimal data set/gt_img_0036_0.jpg]

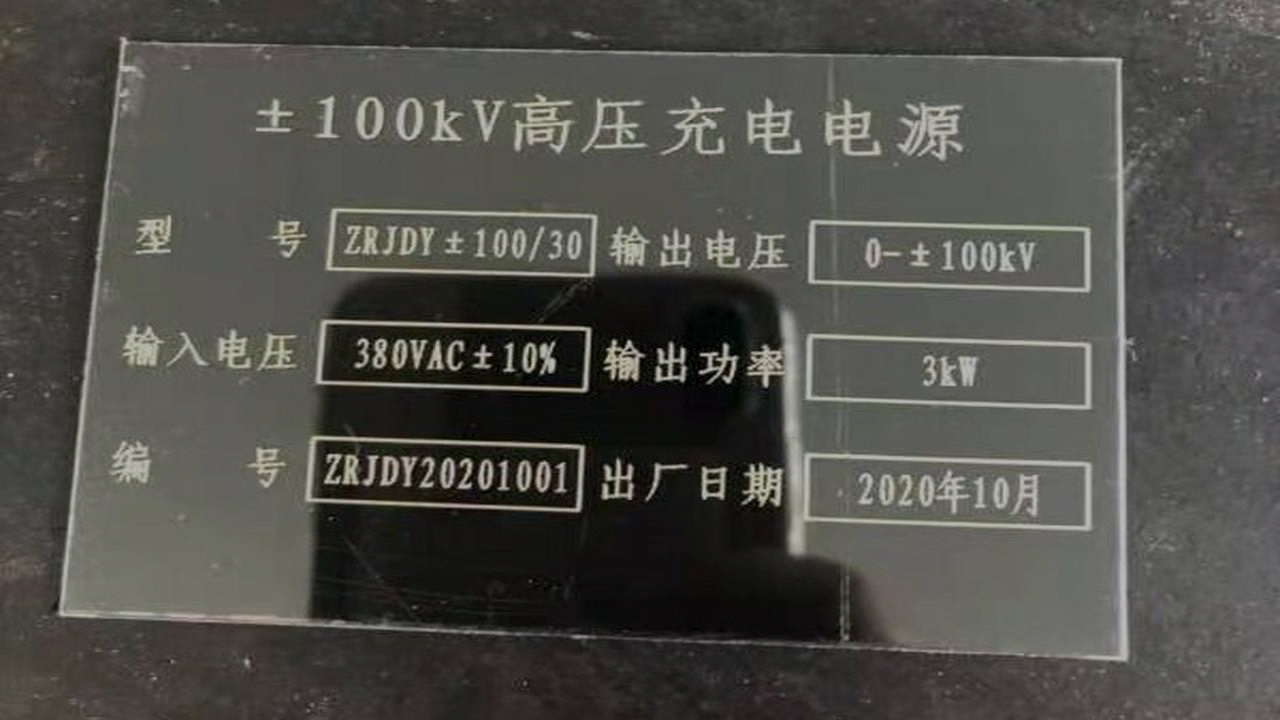

Supplement: S1 Dataset — (ZIP) [file pone.0300792.s001.zip › minimal data set/gt_img_0036_N1.0.jpg]

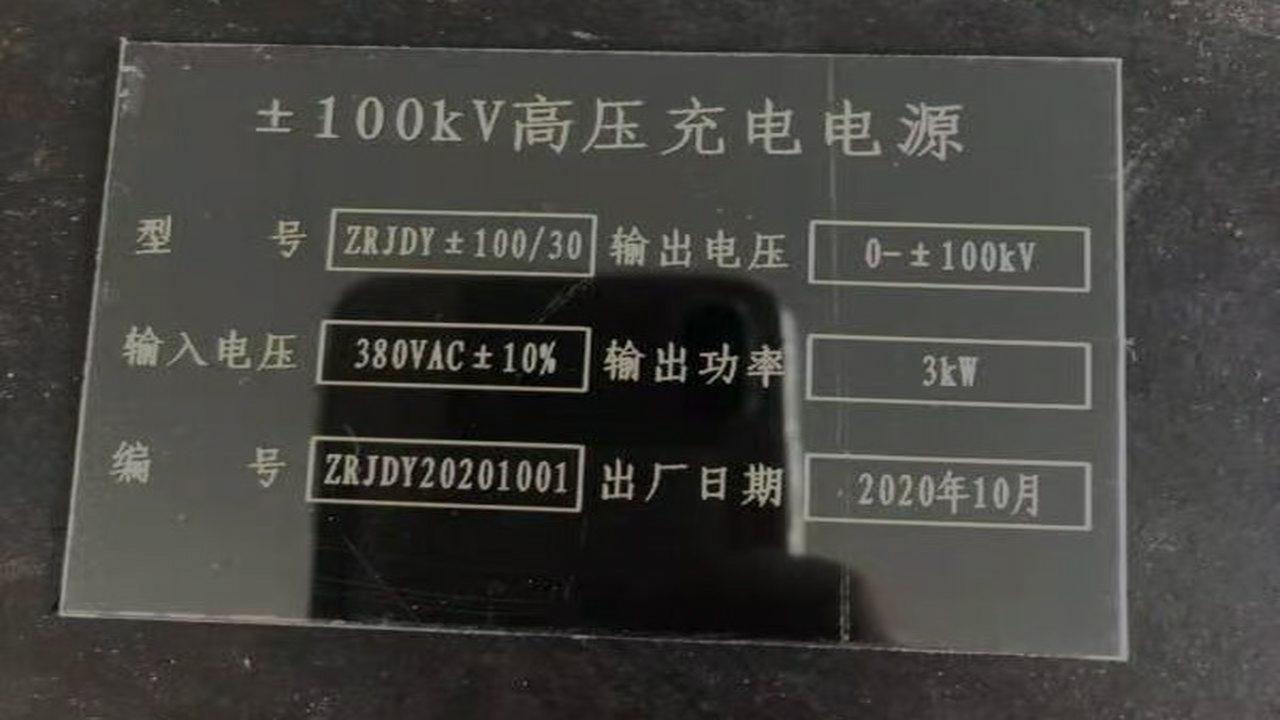

Supplement: S1 Dataset — (ZIP) [file pone.0300792.s001.zip › minimal data set/gt_img_0036_N1.5.jpg]

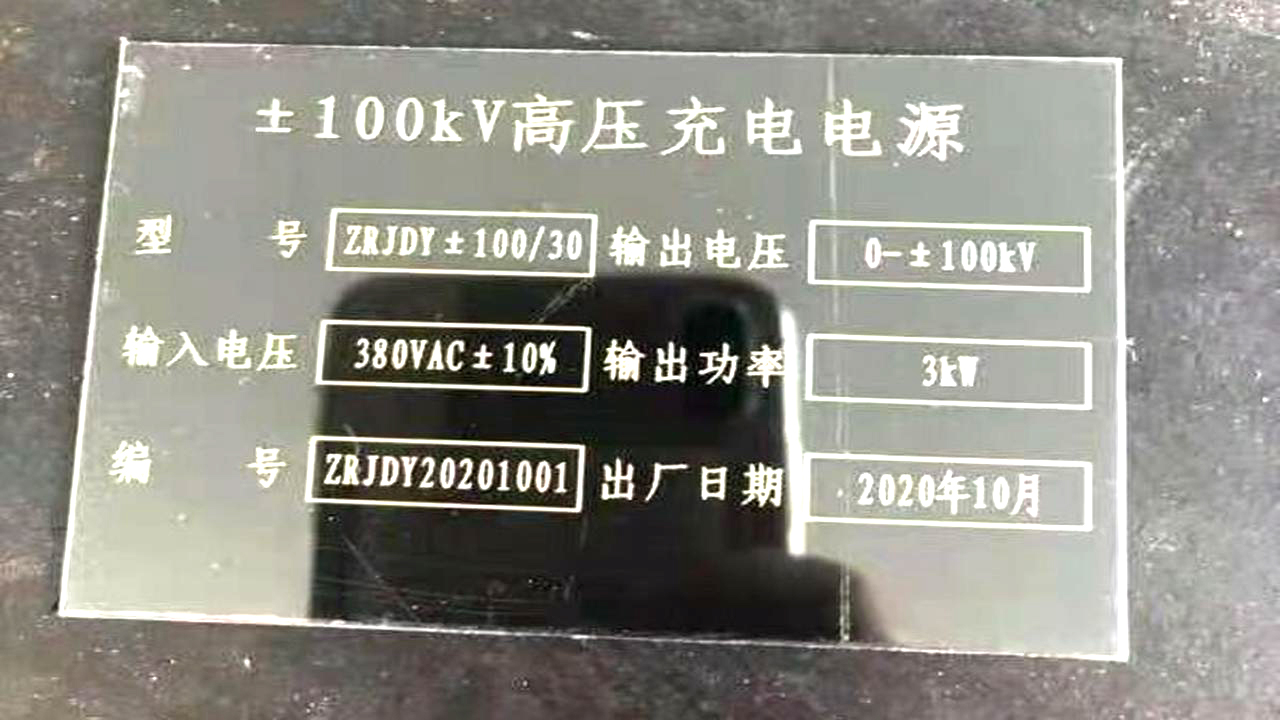

Supplement: S1 Dataset — (ZIP) [file pone.0300792.s001.zip › minimal data set/gt_img_0036_P1.0.jpg]

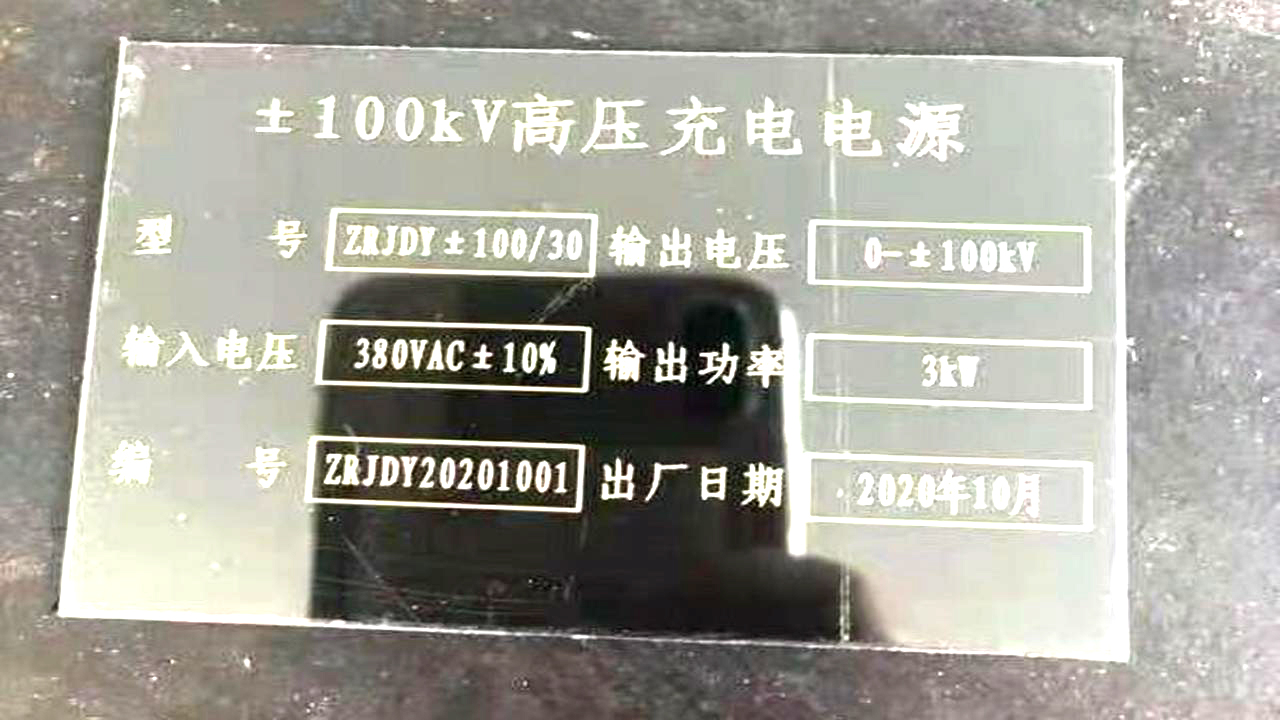

Supplement: S1 Dataset — (ZIP) [file pone.0300792.s001.zip › minimal data set/gt_img_0036_P1.5.jpg]

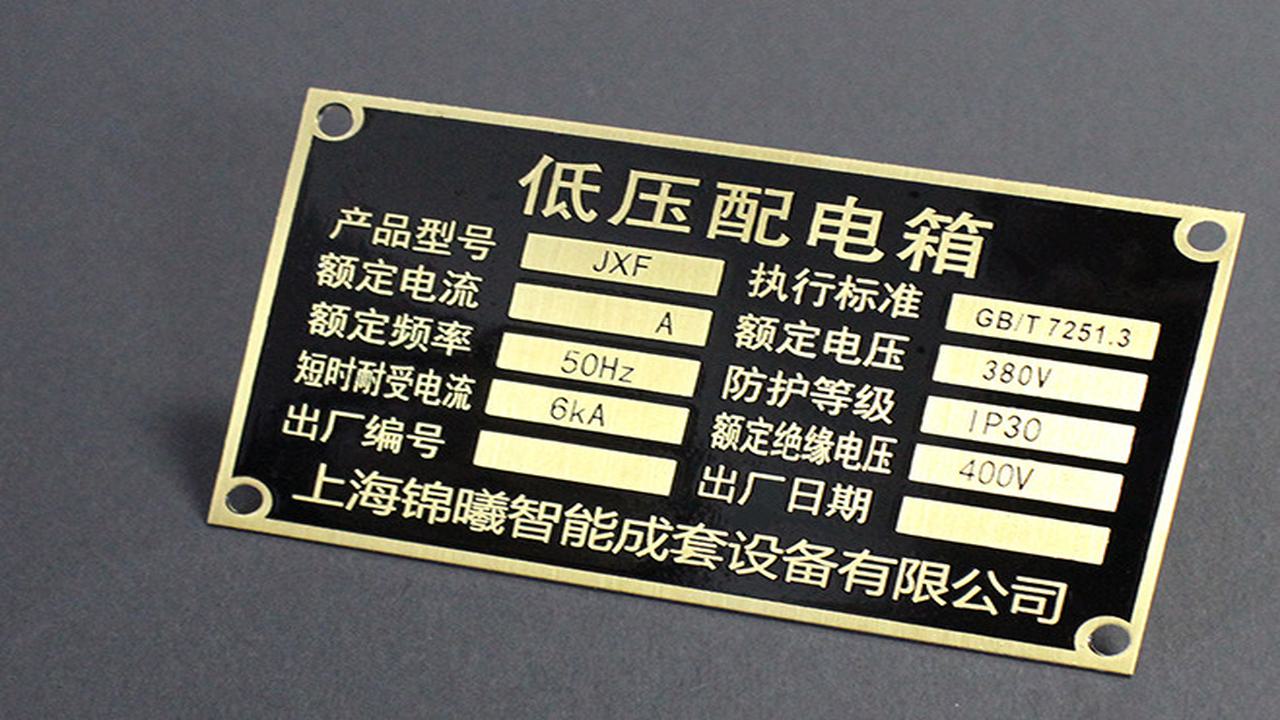

Supplement: S1 Dataset — (ZIP) [file pone.0300792.s001.zip › minimal data set/gt_img_0037_0.jpg]

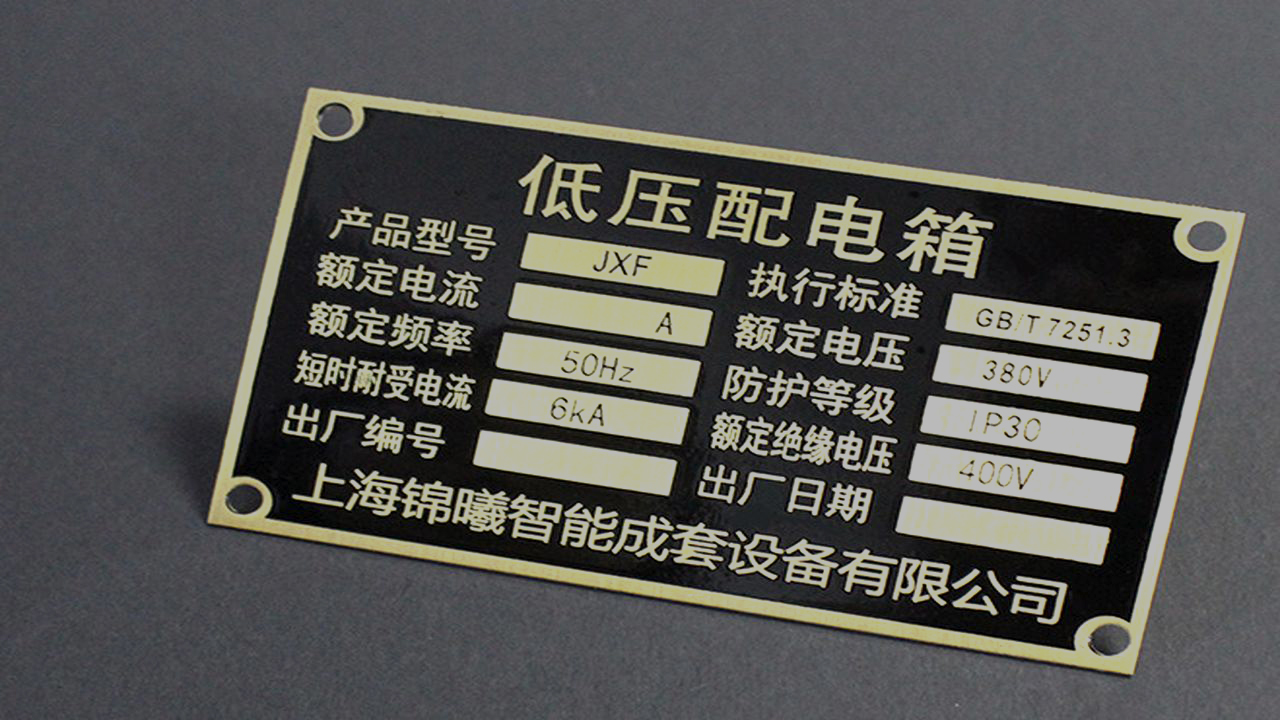

Supplement: S1 Dataset — (ZIP) [file pone.0300792.s001.zip › minimal data set/gt_img_0037_N1.0.jpg]

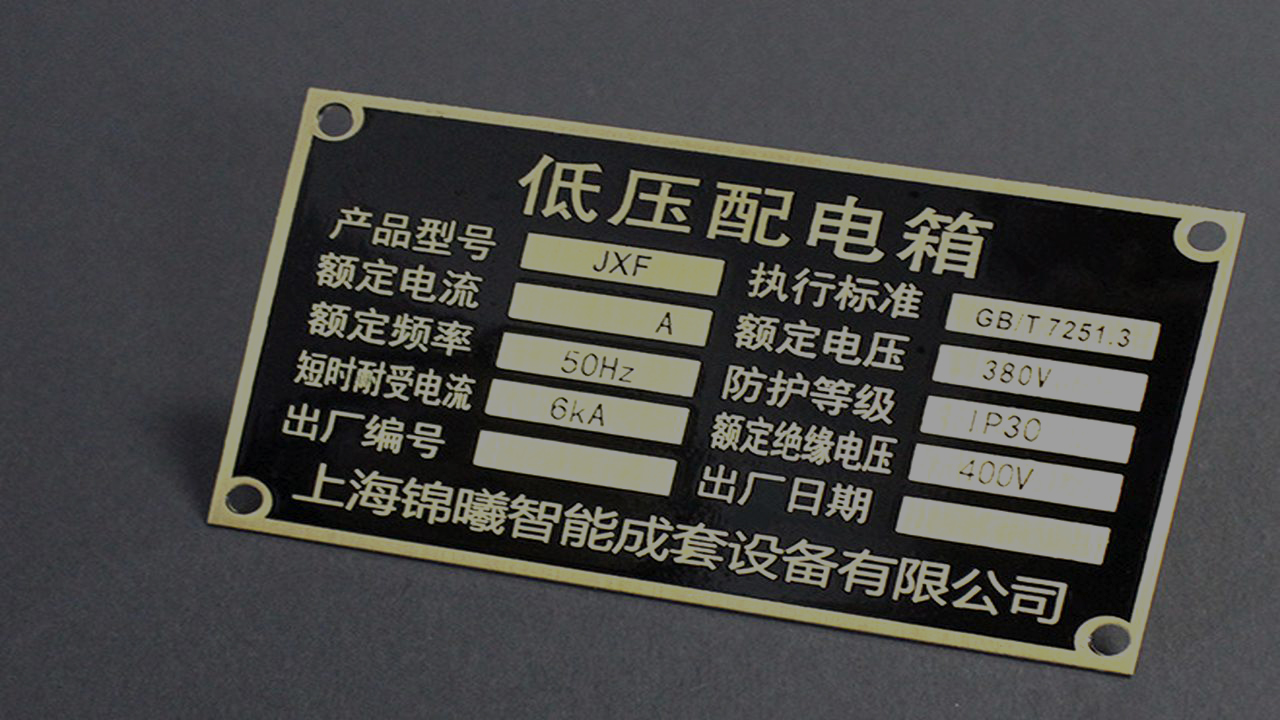

Supplement: S1 Dataset — (ZIP) [file pone.0300792.s001.zip › minimal data set/gt_img_0037_N1.5.jpg]

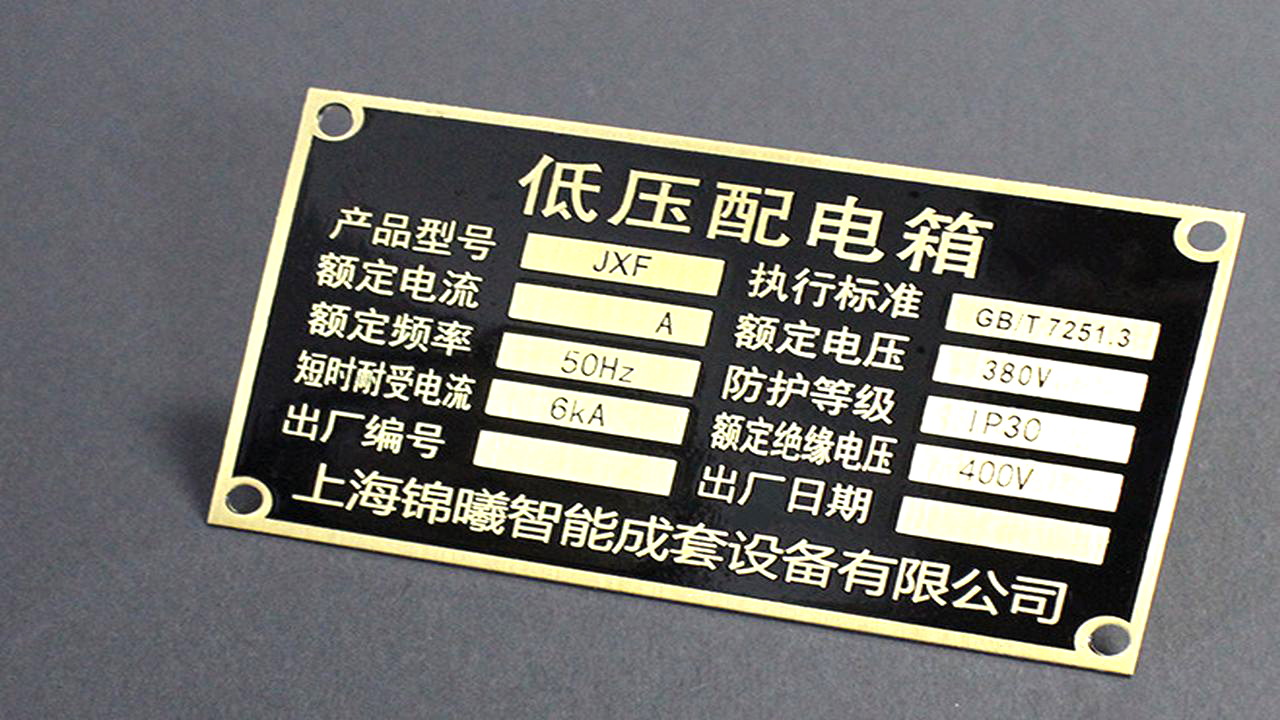

Supplement: S1 Dataset — (ZIP) [file pone.0300792.s001.zip › minimal data set/gt_img_0037_P1.0.jpg]

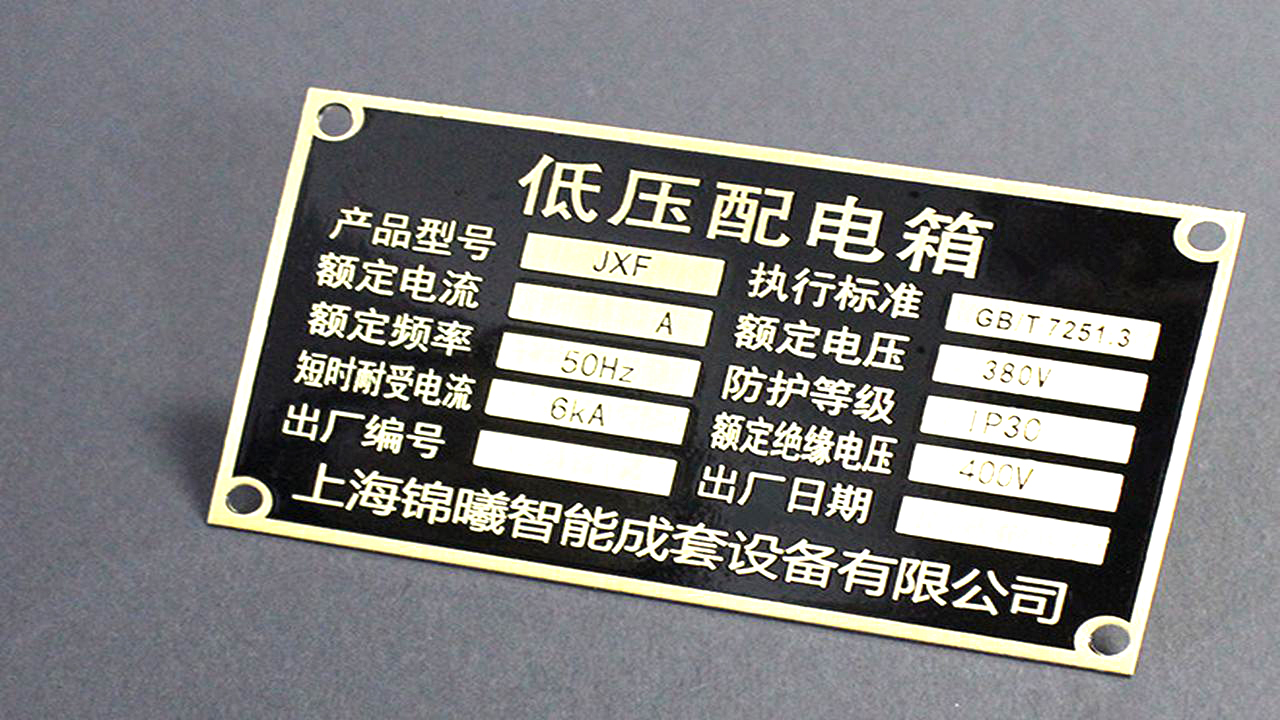

Supplement: S1 Dataset — (ZIP) [file pone.0300792.s001.zip › minimal data set/gt_img_0037_P1.5.jpg]

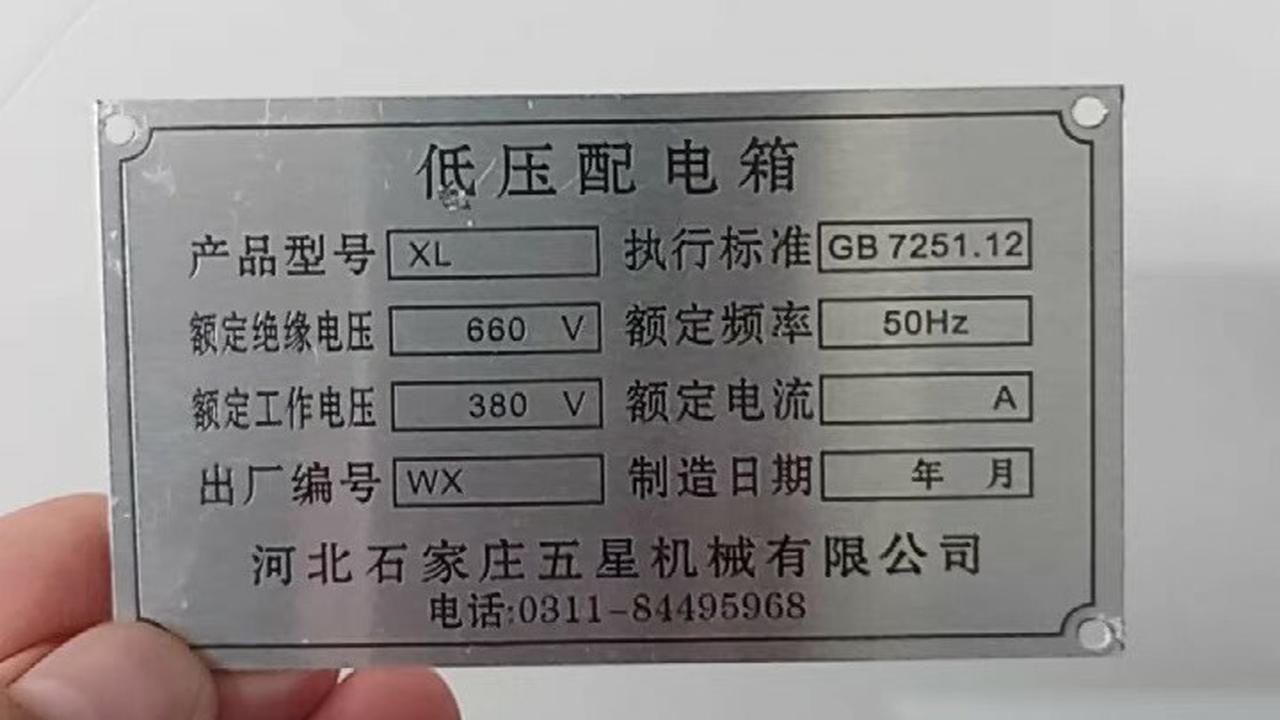

Supplement: S1 Dataset — (ZIP) [file pone.0300792.s001.zip › minimal data set/gt_img_0038_0.jpg]

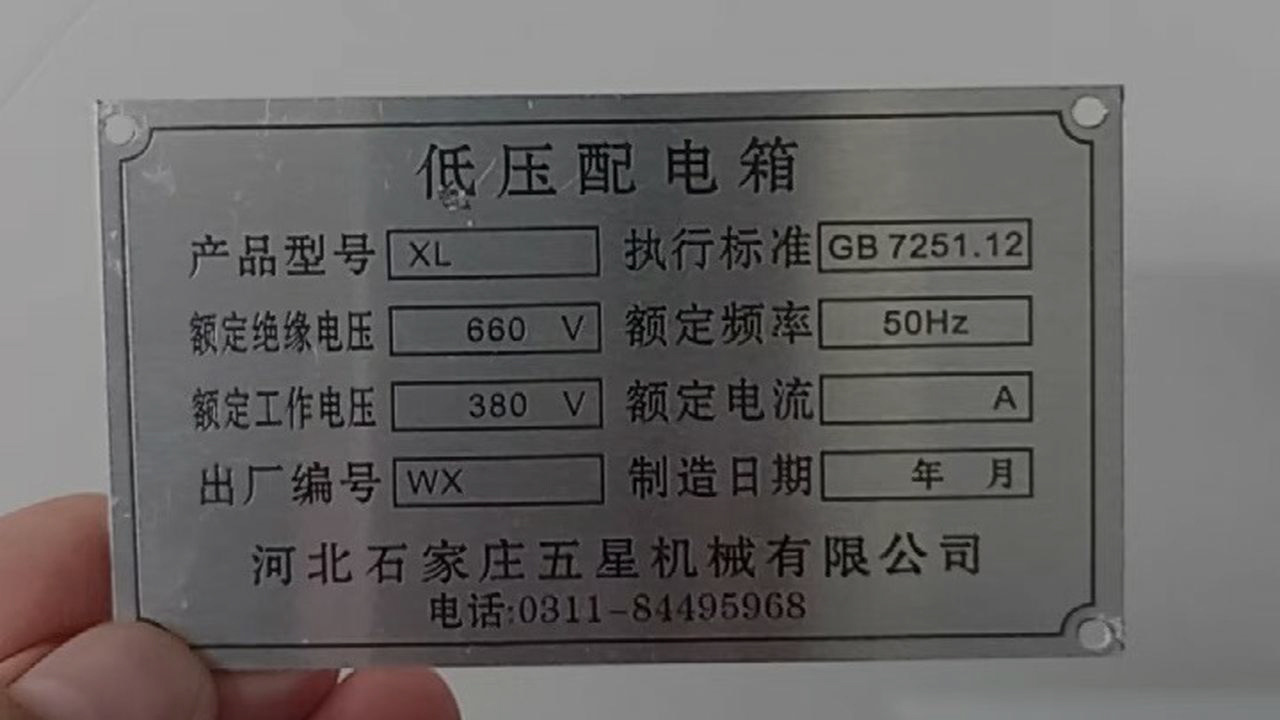

Supplement: S1 Dataset — (ZIP) [file pone.0300792.s001.zip › minimal data set/gt_img_0038_N1.0.jpg]

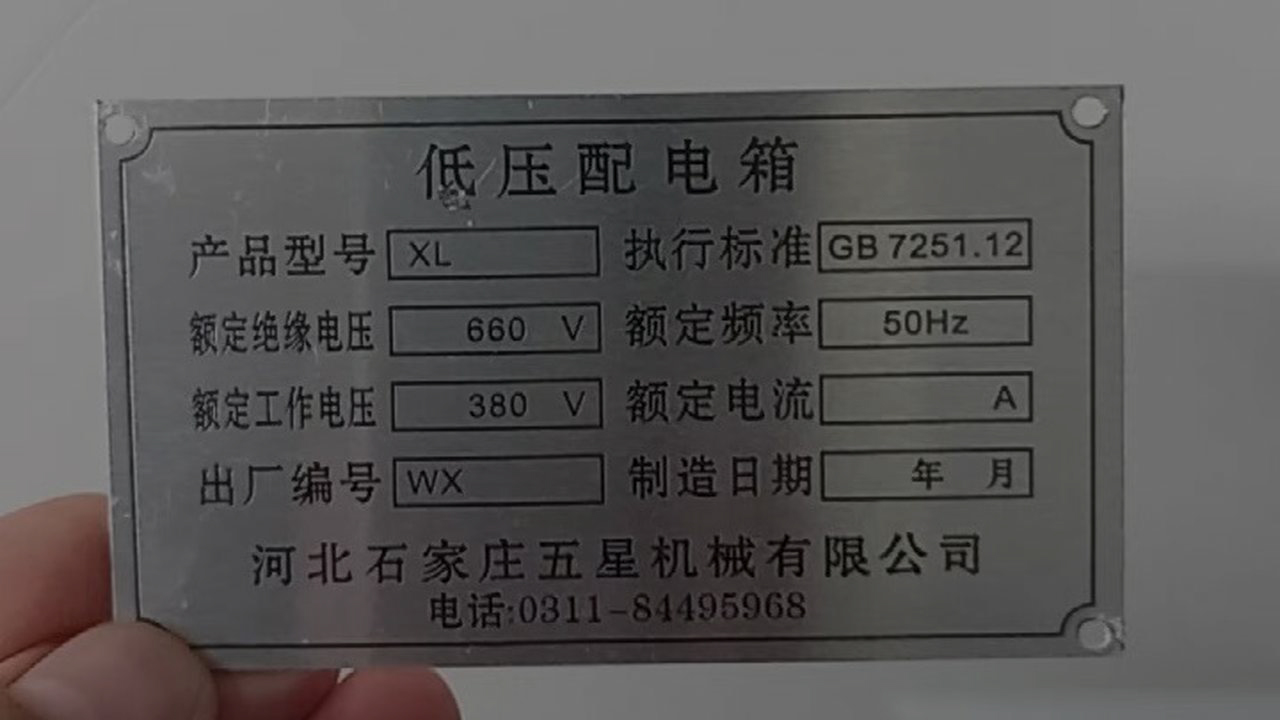

Supplement: S1 Dataset — (ZIP) [file pone.0300792.s001.zip › minimal data set/gt_img_0038_N1.5.jpg]

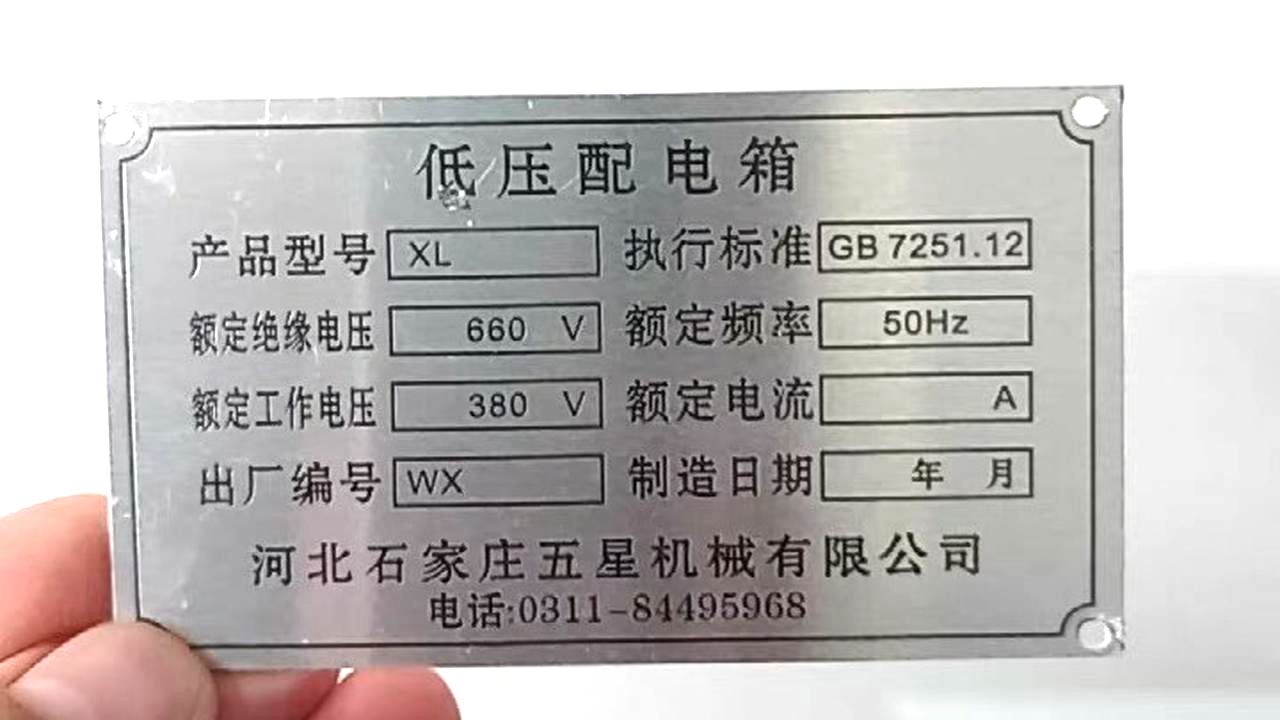

Supplement: S1 Dataset — (ZIP) [file pone.0300792.s001.zip › minimal data set/gt_img_0038_P1.0.jpg]

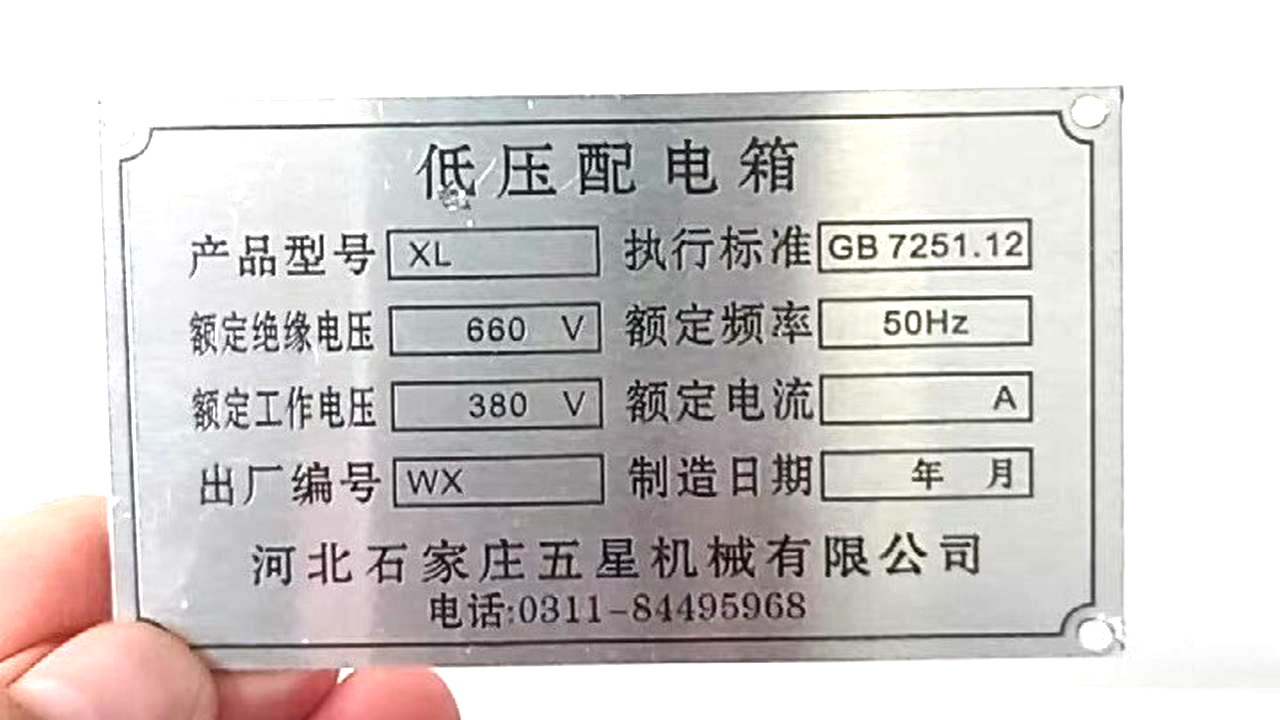

Supplement: S1 Dataset — (ZIP) [file pone.0300792.s001.zip › minimal data set/gt_img_0038_P1.5.jpg]

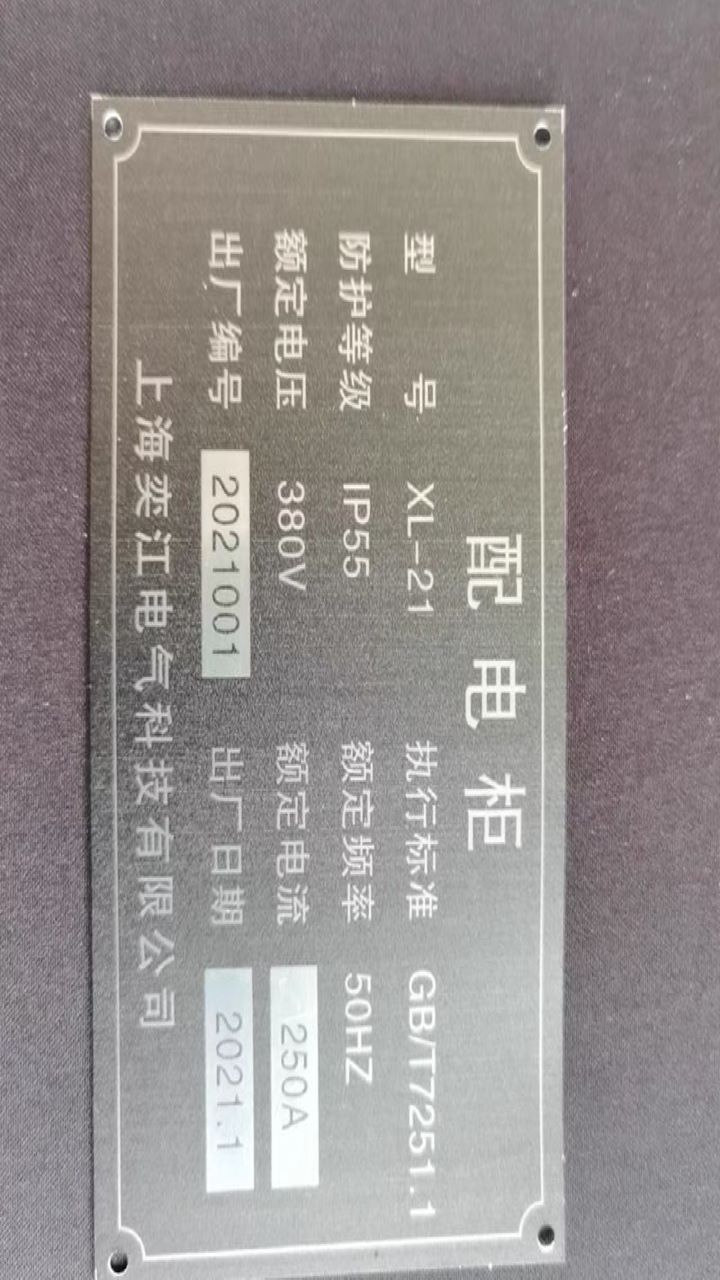

Supplement: S1 Dataset — (ZIP) [file pone.0300792.s001.zip › minimal data set/gt_img_0039_0.jpg]

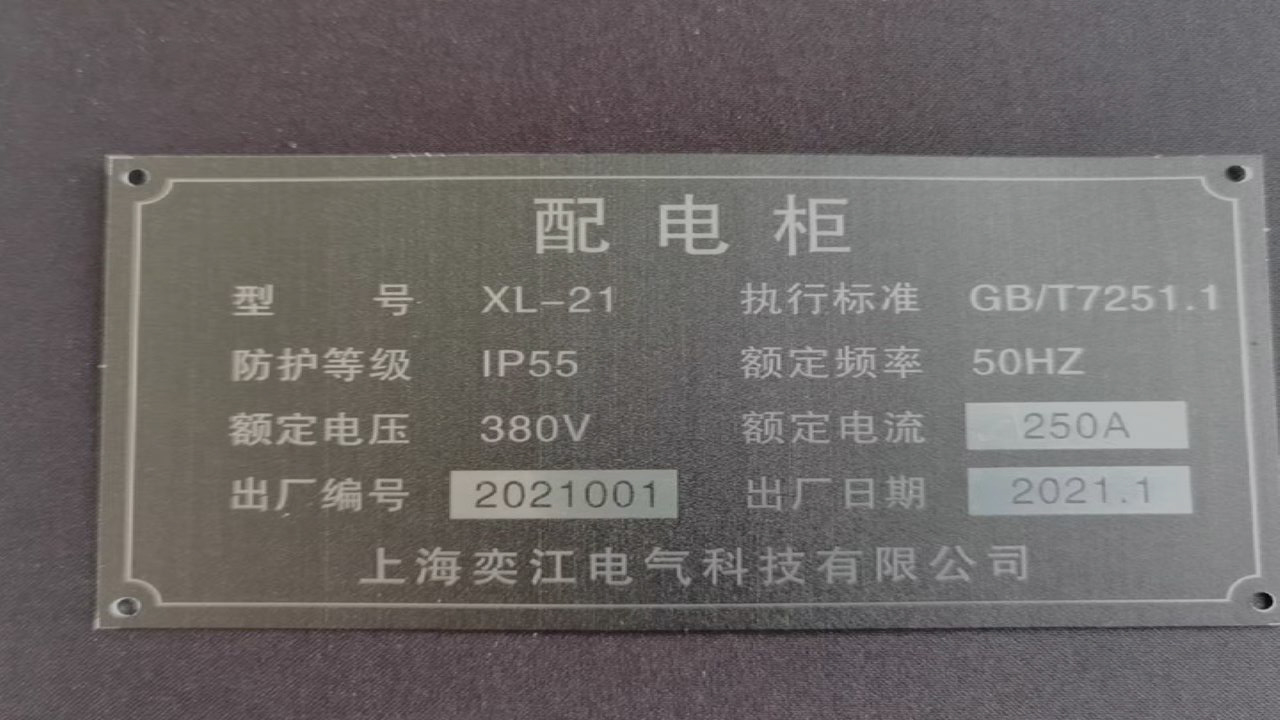

Supplement: S1 Dataset — (ZIP) [file pone.0300792.s001.zip › minimal data set/gt_img_0039_N1.0.jpg]

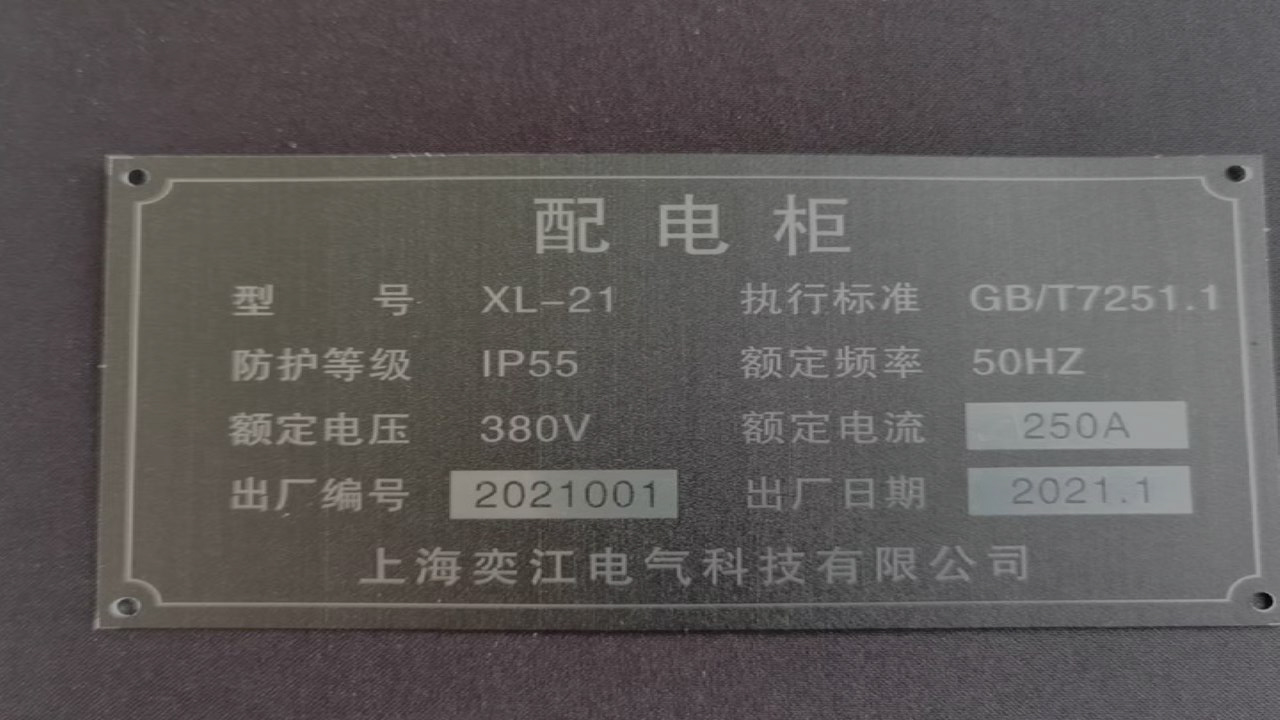

Supplement: S1 Dataset — (ZIP) [file pone.0300792.s001.zip › minimal data set/gt_img_0039_N1.5.jpg]

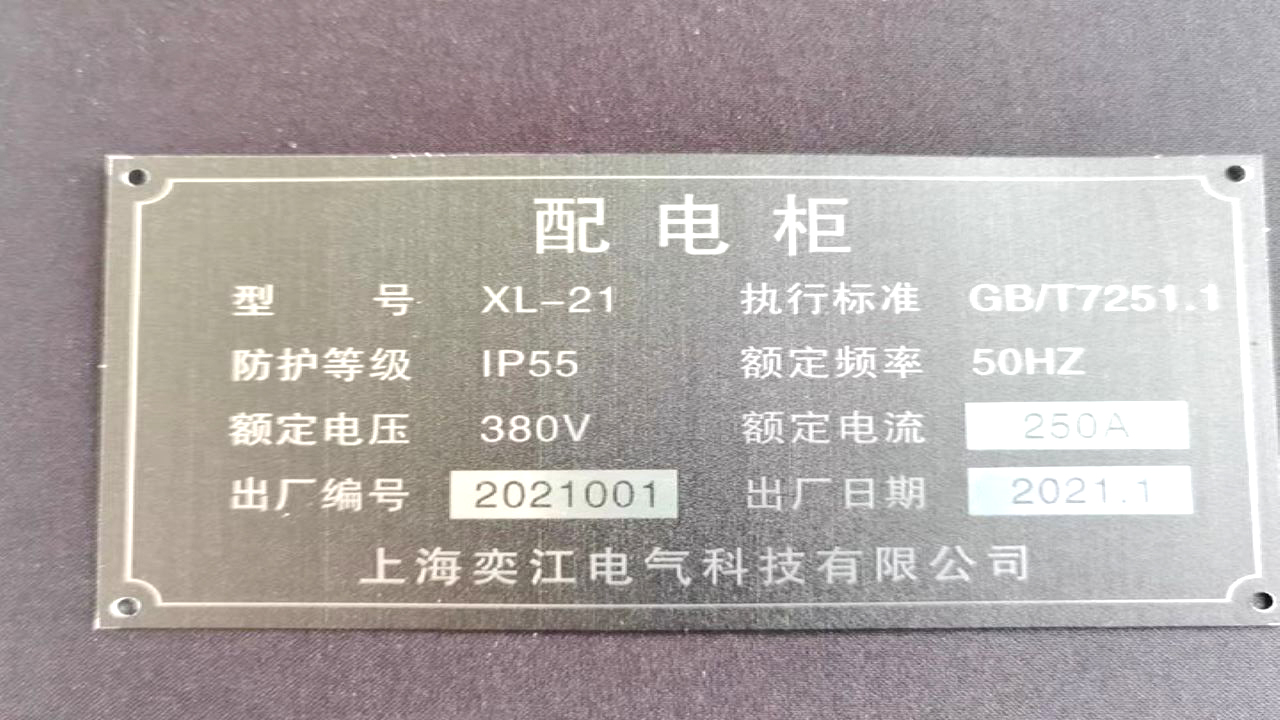

Supplement: S1 Dataset — (ZIP) [file pone.0300792.s001.zip › minimal data set/gt_img_0039_P1.0.jpg]

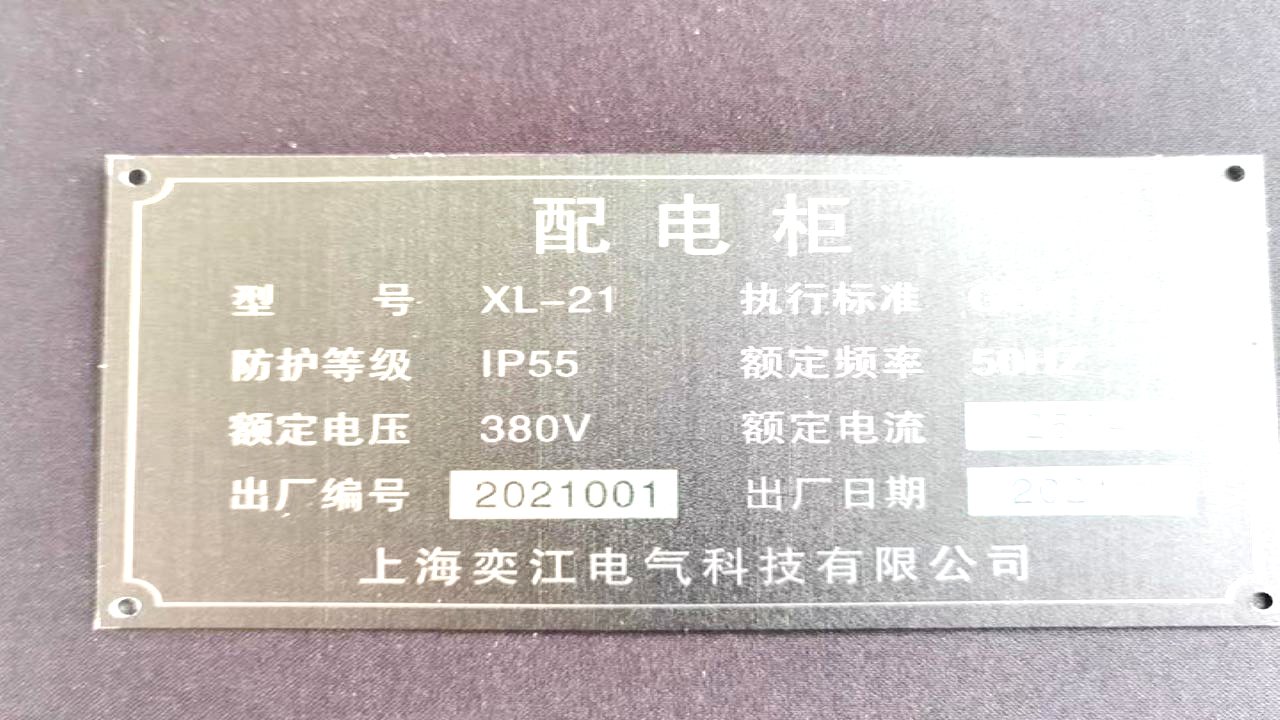

Supplement: S1 Dataset — (ZIP) [file pone.0300792.s001.zip › minimal data set/gt_img_0039_P1.5.jpg]

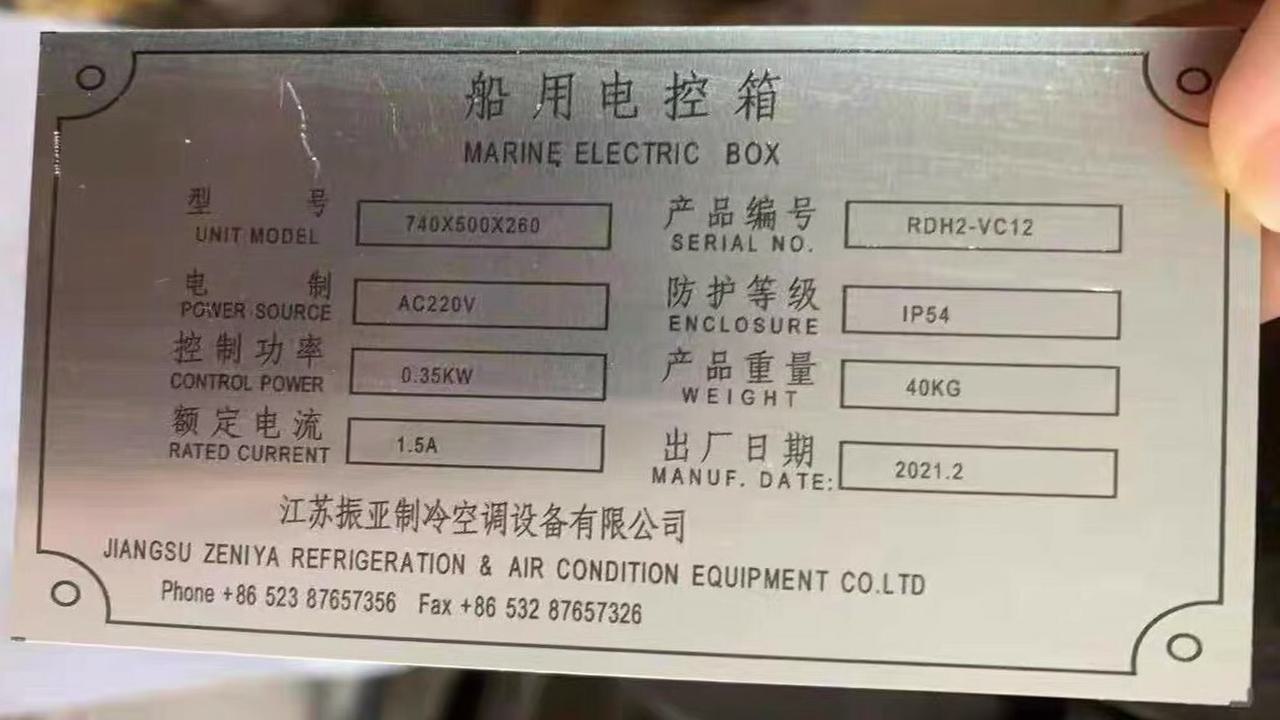

Supplement: S1 Dataset — (ZIP) [file pone.0300792.s001.zip › minimal data set/gt_img_0040_0.jpg]

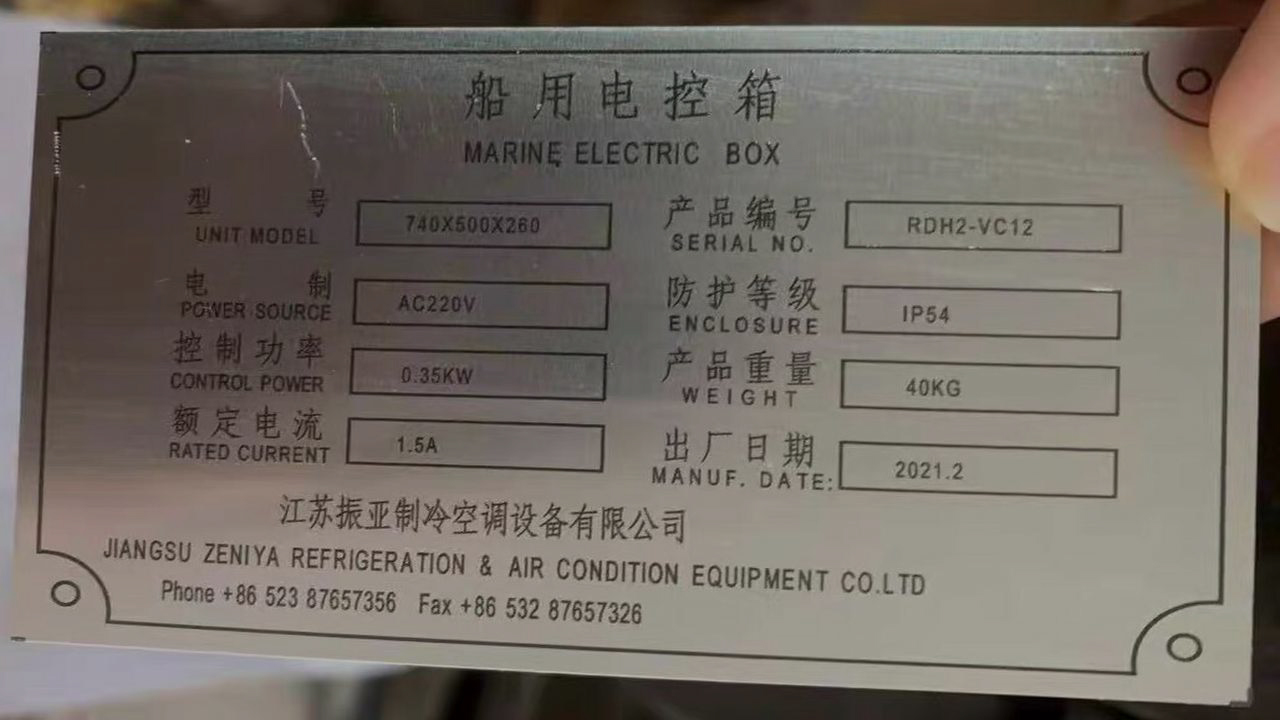

Supplement: S1 Dataset — (ZIP) [file pone.0300792.s001.zip › minimal data set/gt_img_0040_N1.0.jpg]

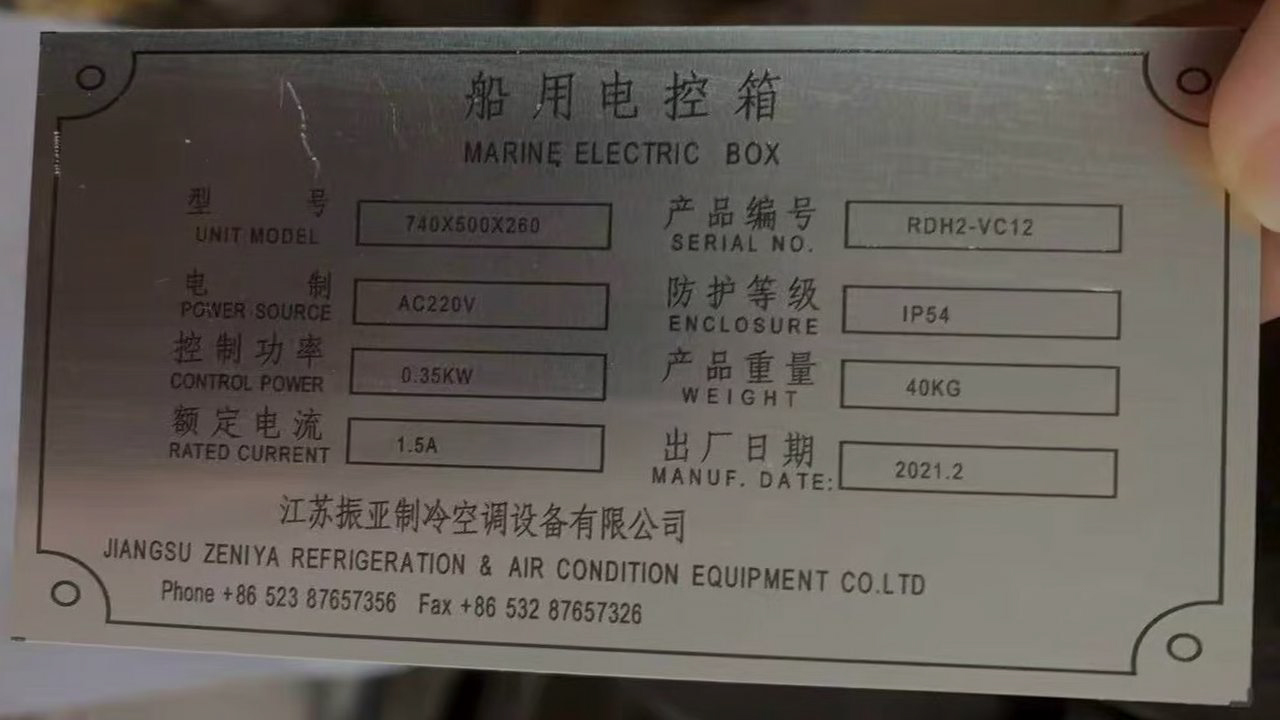

Supplement: S1 Dataset — (ZIP) [file pone.0300792.s001.zip › minimal data set/gt_img_0040_N1.5.jpg]

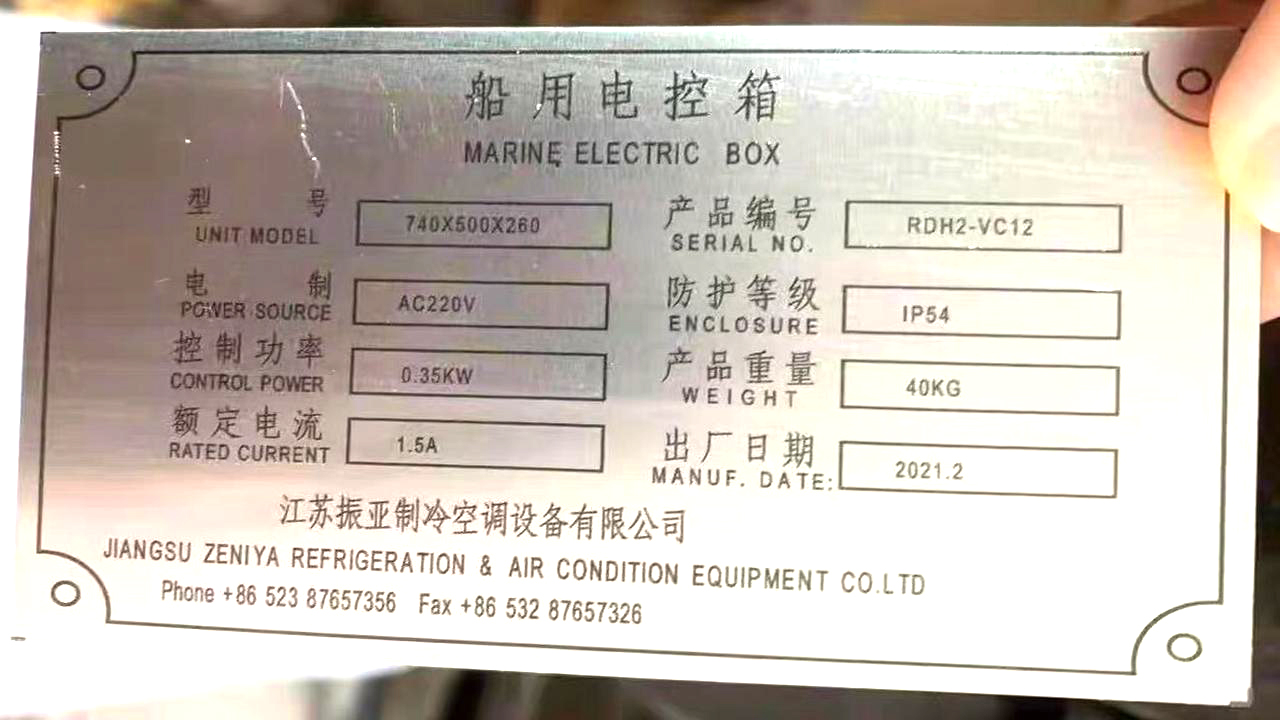

Supplement: S1 Dataset — (ZIP) [file pone.0300792.s001.zip › minimal data set/gt_img_0040_P1.0.jpg]

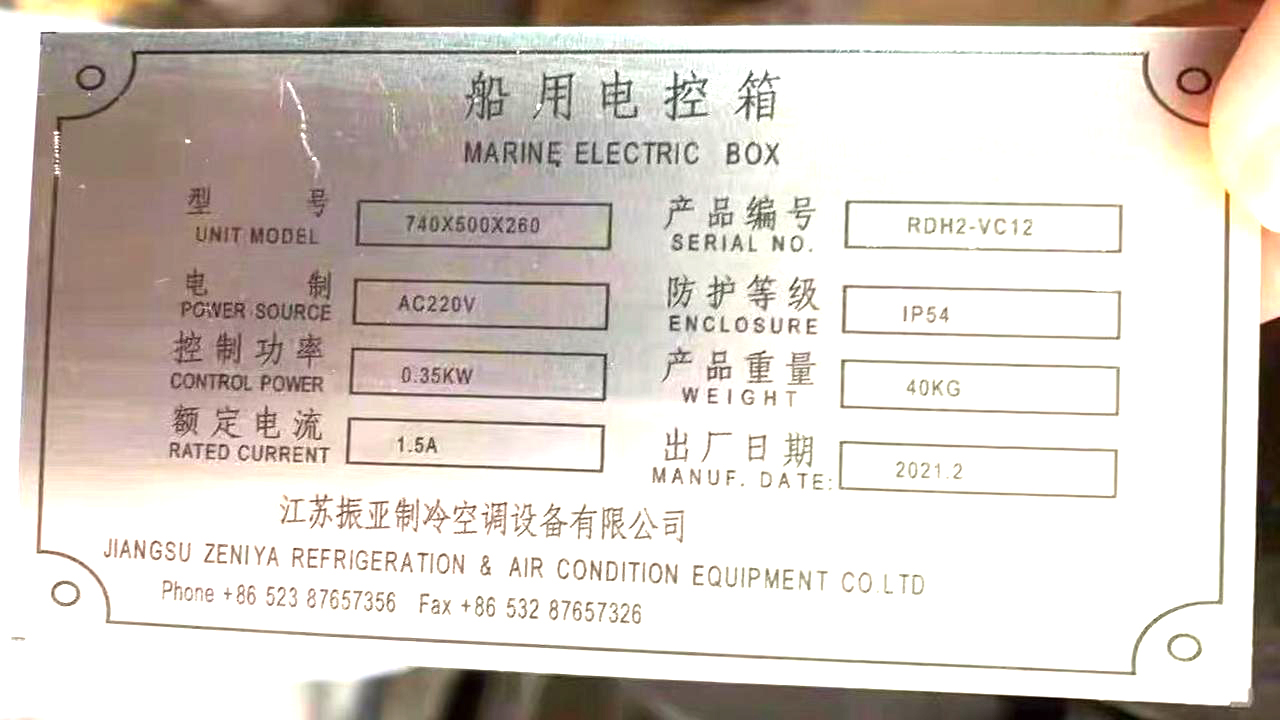

Supplement: S1 Dataset — (ZIP) [file pone.0300792.s001.zip › minimal data set/gt_img_0040_P1.5.jpg]
