# Supplementary figures and images for: Exposure image correction of electrical equipment nameplate based on the LMPEC algorithm (part 1 of 2)
Source: PLoS One. 2024 Jun 27;19(6):e0300792. doi: 10.1371/journal.pone.0300792 (PMC11210806; doi:10.1371/journal.pone.0300792)

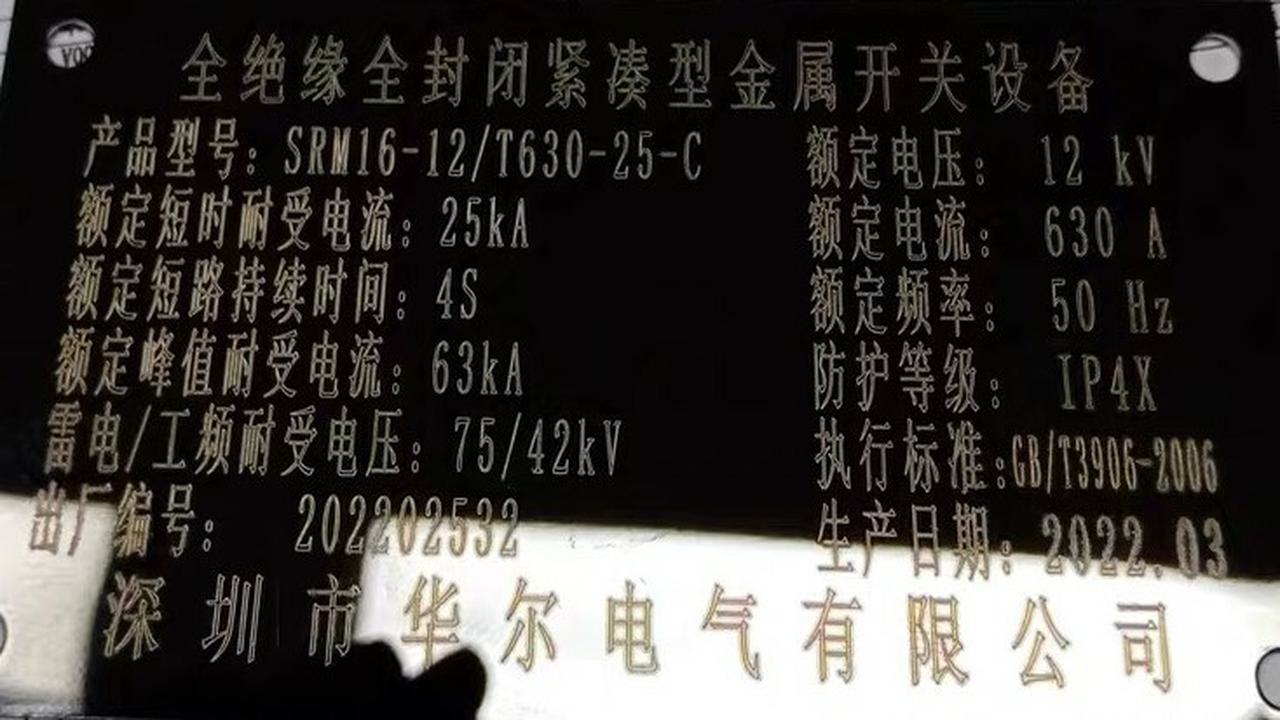

Supplement: S1 Dataset — (ZIP) [file pone.0300792.s001.zip › minimal data set/gt_img_0001_0.jpg]

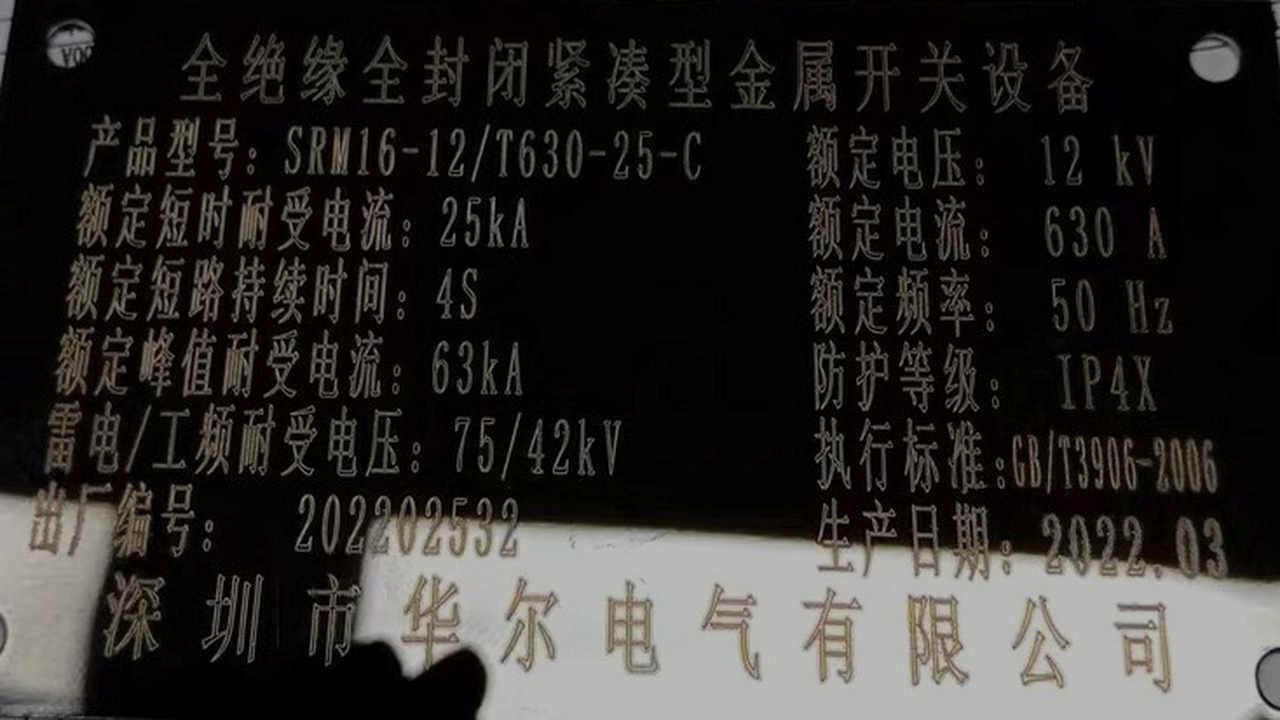

Supplement: S1 Dataset — (ZIP) [file pone.0300792.s001.zip › minimal data set/gt_img_0001_N1.0.jpg]

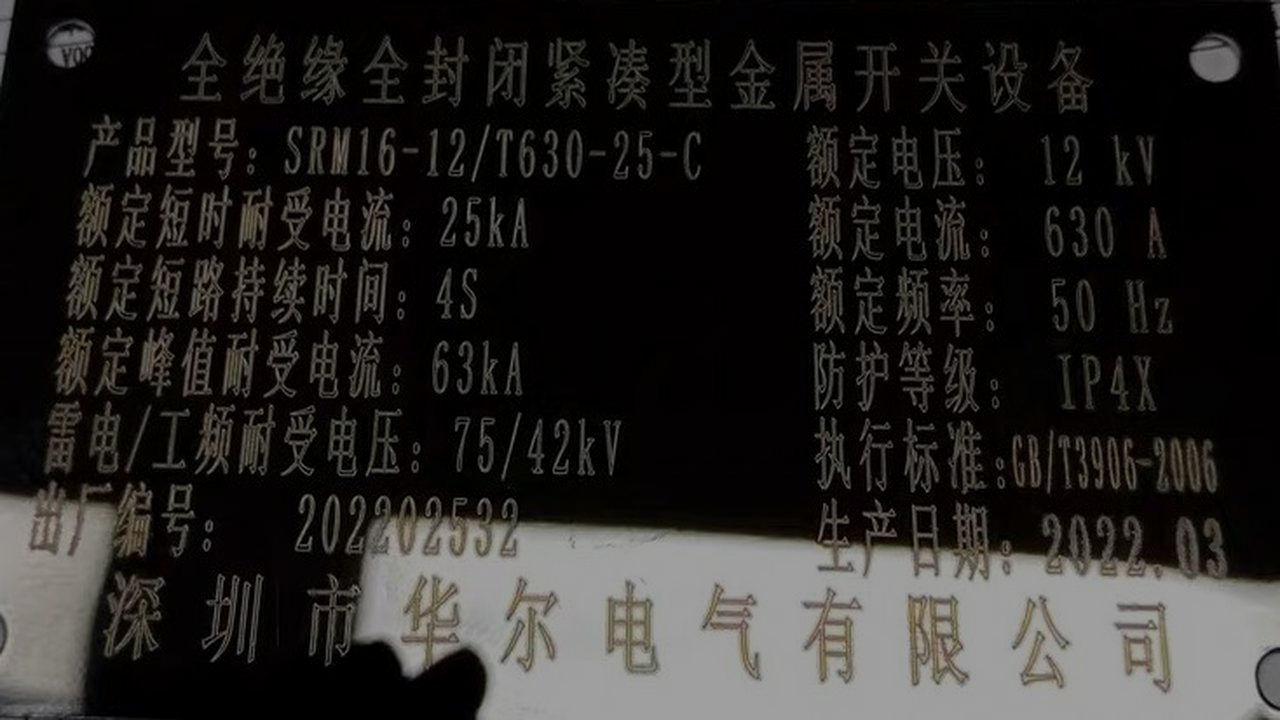

Supplement: S1 Dataset — (ZIP) [file pone.0300792.s001.zip › minimal data set/gt_img_0001_N1.5.jpg]

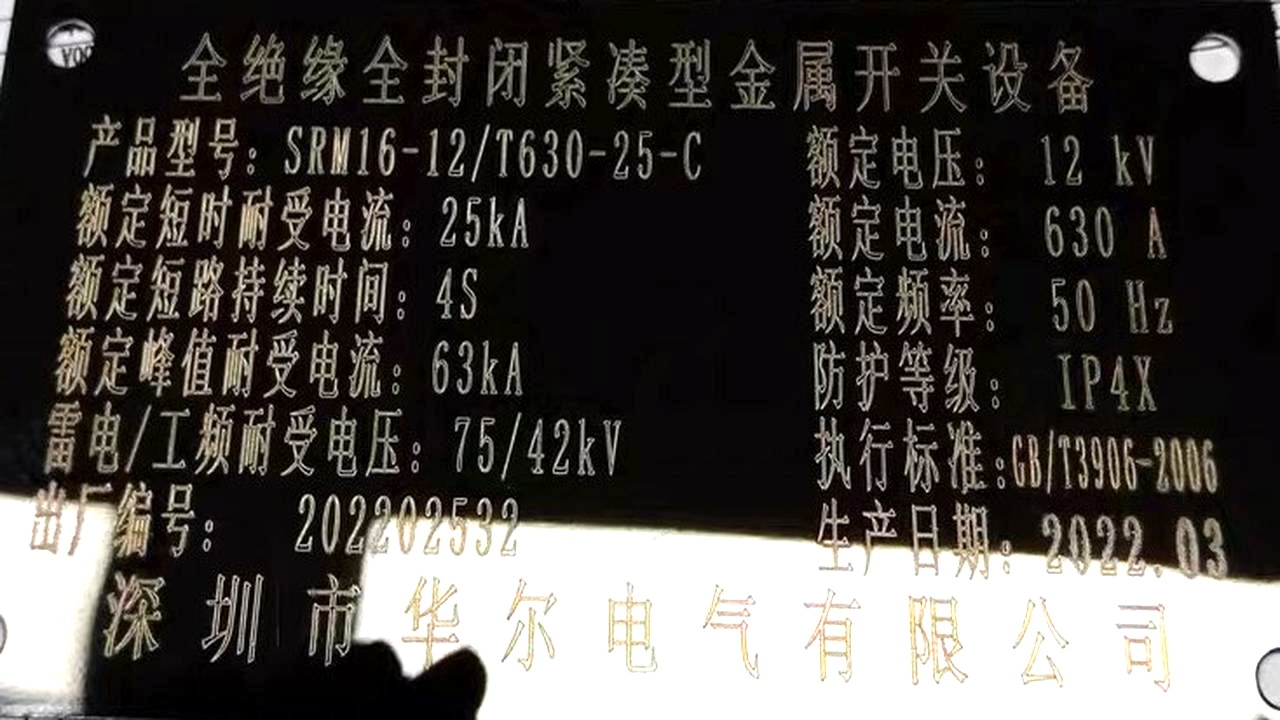

Supplement: S1 Dataset — (ZIP) [file pone.0300792.s001.zip › minimal data set/gt_img_0001_P1.0.jpg]

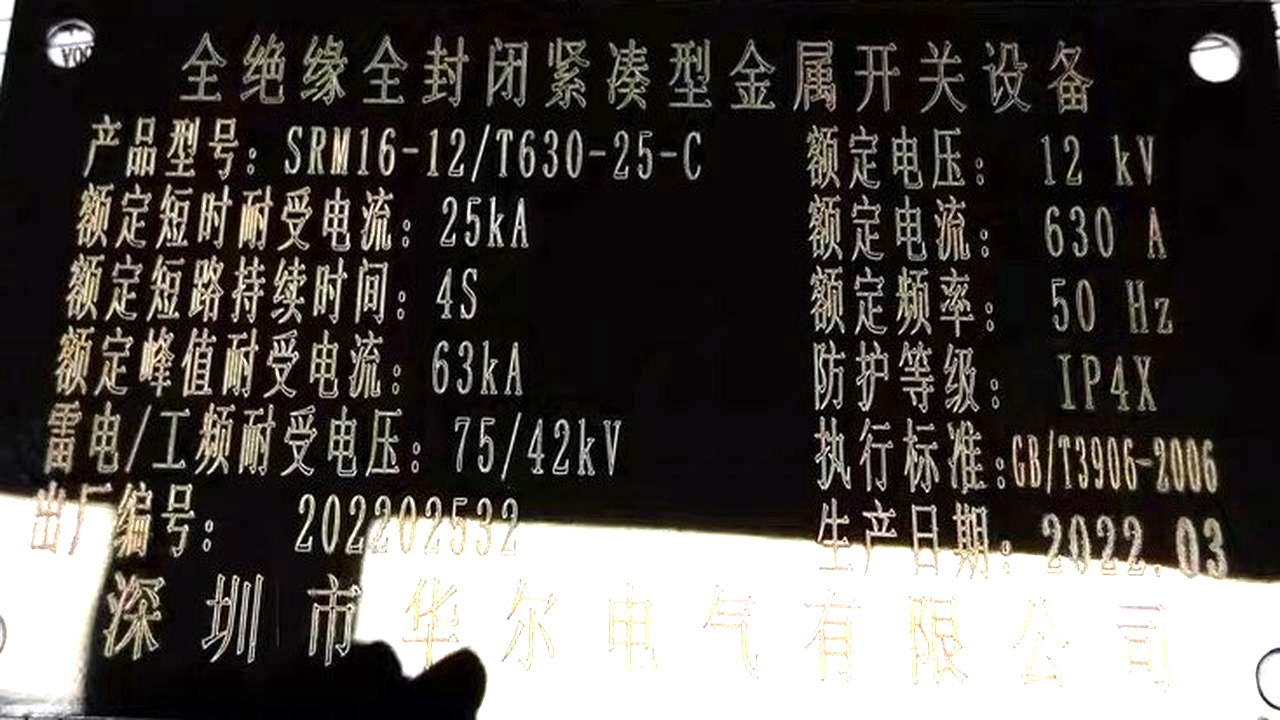

Supplement: S1 Dataset — (ZIP) [file pone.0300792.s001.zip › minimal data set/gt_img_0001_P1.5.jpg]

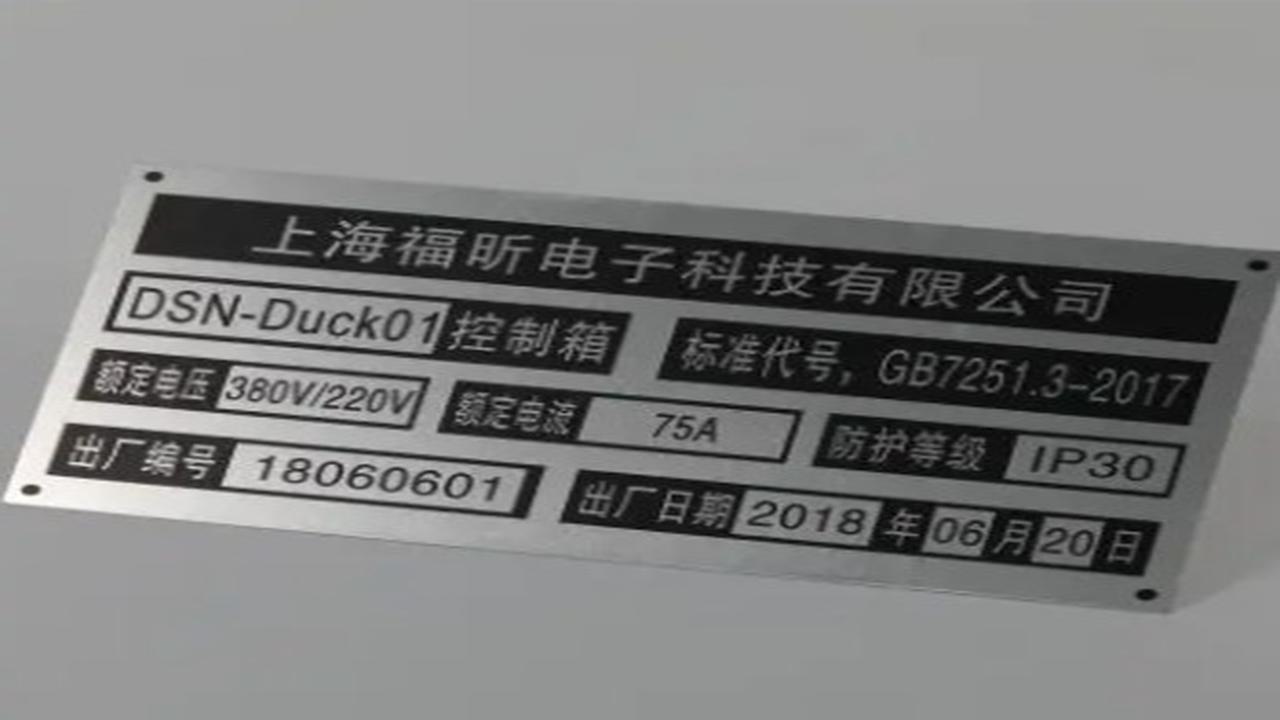

Supplement: S1 Dataset — (ZIP) [file pone.0300792.s001.zip › minimal data set/gt_img_0002_0.jpg]

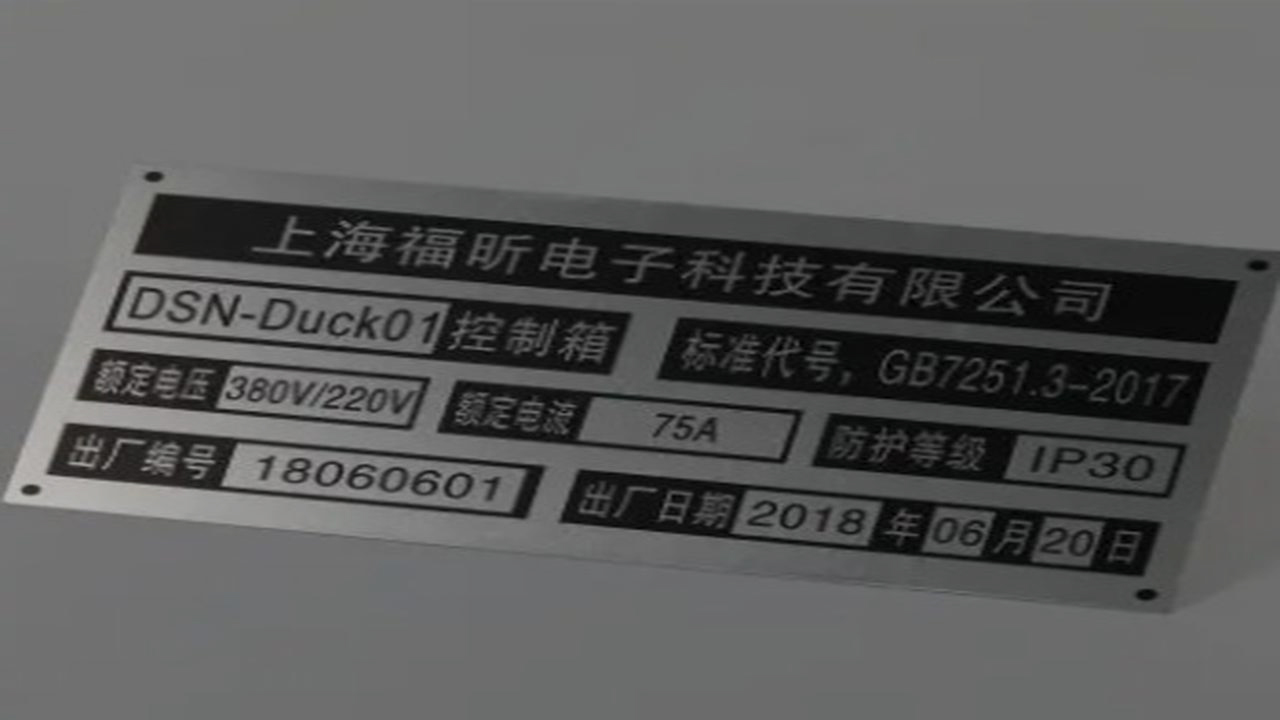

Supplement: S1 Dataset — (ZIP) [file pone.0300792.s001.zip › minimal data set/gt_img_0002_N1.0.jpg]

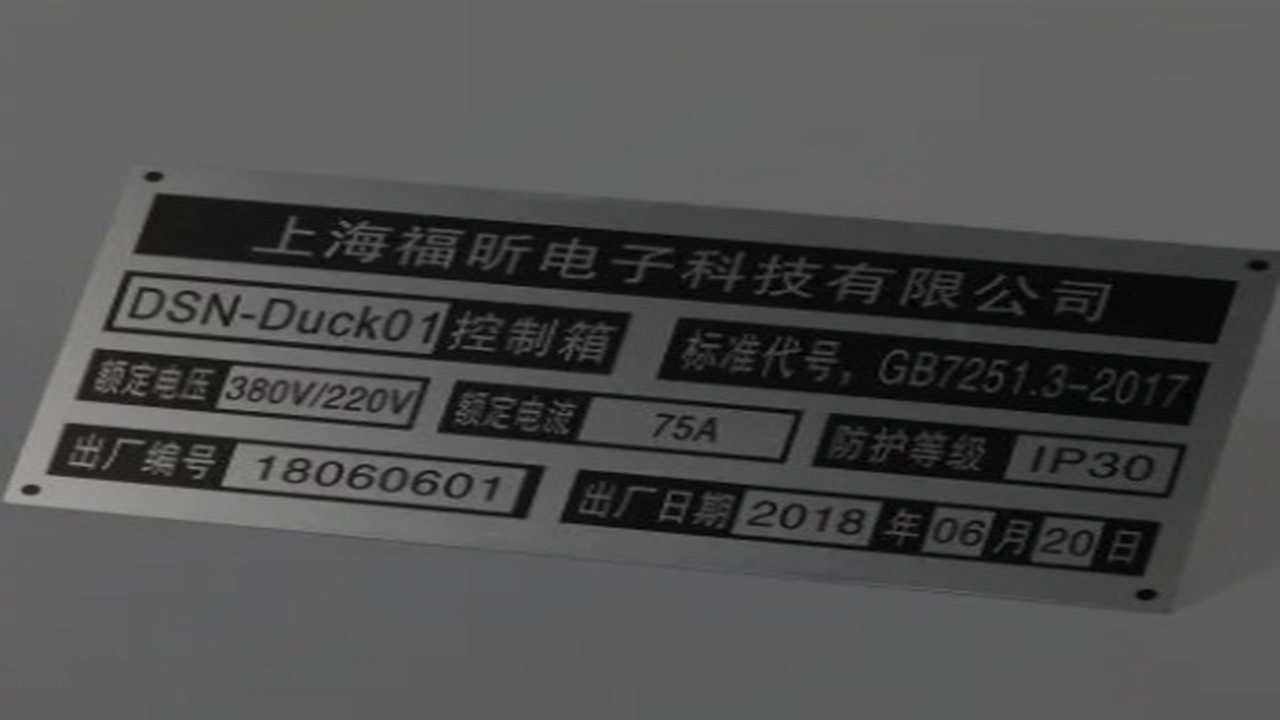

Supplement: S1 Dataset — (ZIP) [file pone.0300792.s001.zip › minimal data set/gt_img_0002_N1.5.jpg]

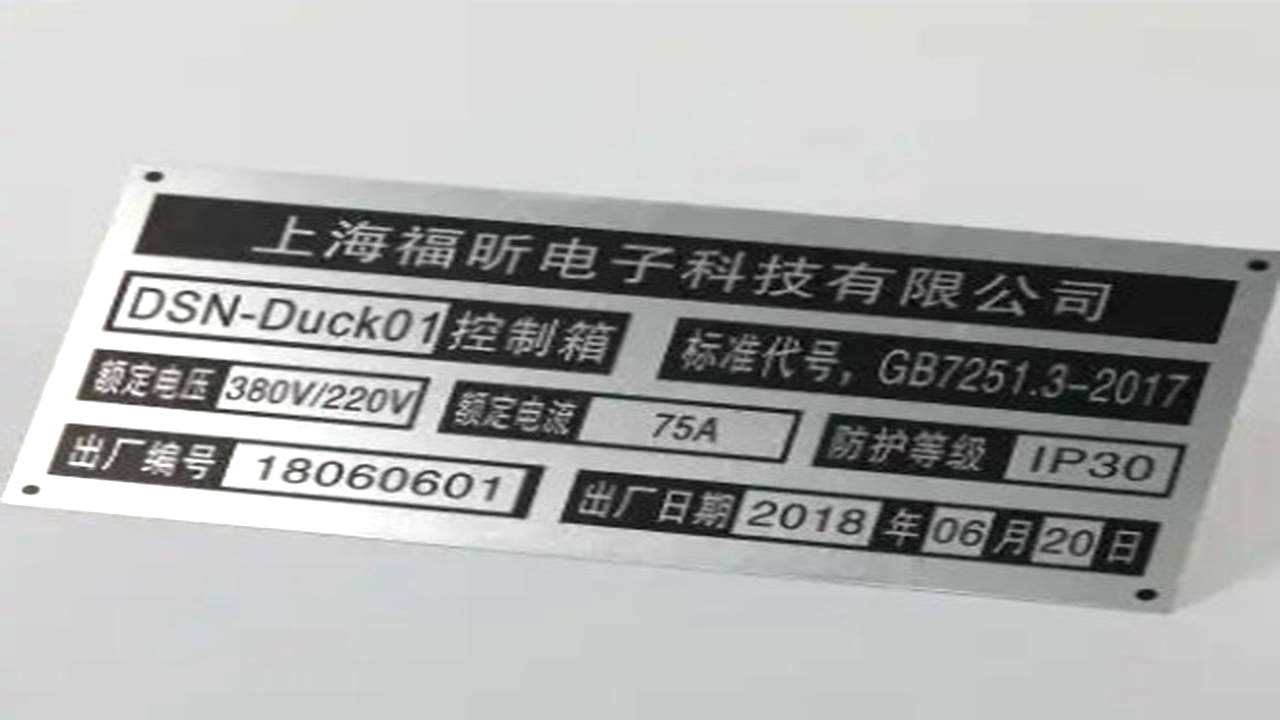

Supplement: S1 Dataset — (ZIP) [file pone.0300792.s001.zip › minimal data set/gt_img_0002_P1.0.jpg]

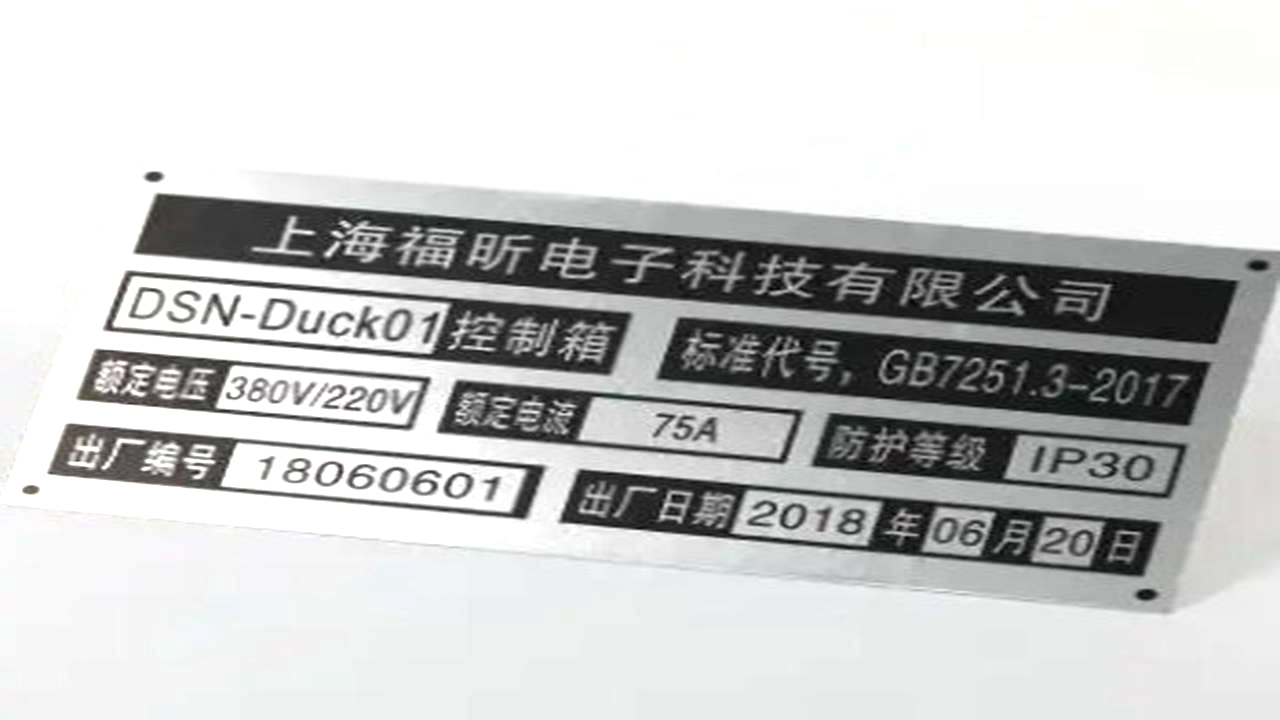

Supplement: S1 Dataset — (ZIP) [file pone.0300792.s001.zip › minimal data set/gt_img_0002_P1.5.jpg]

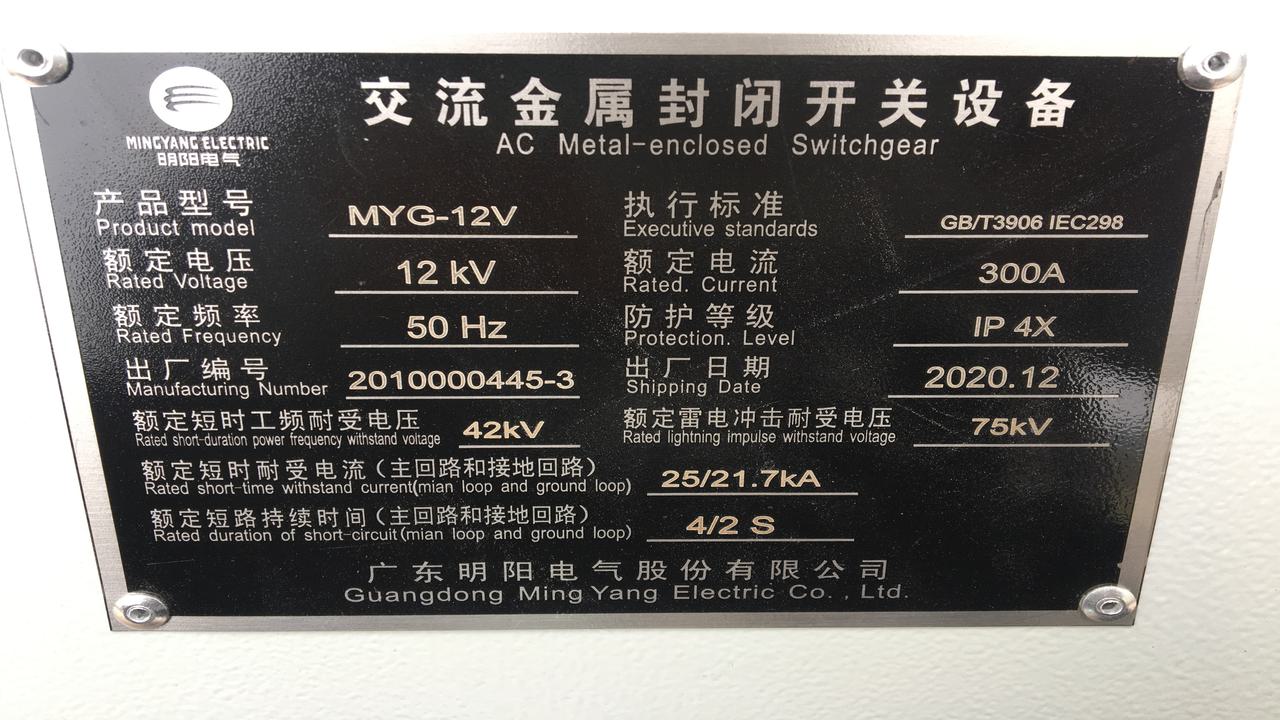

Supplement: S1 Dataset — (ZIP) [file pone.0300792.s001.zip › minimal data set/gt_img_0003_0.jpg]

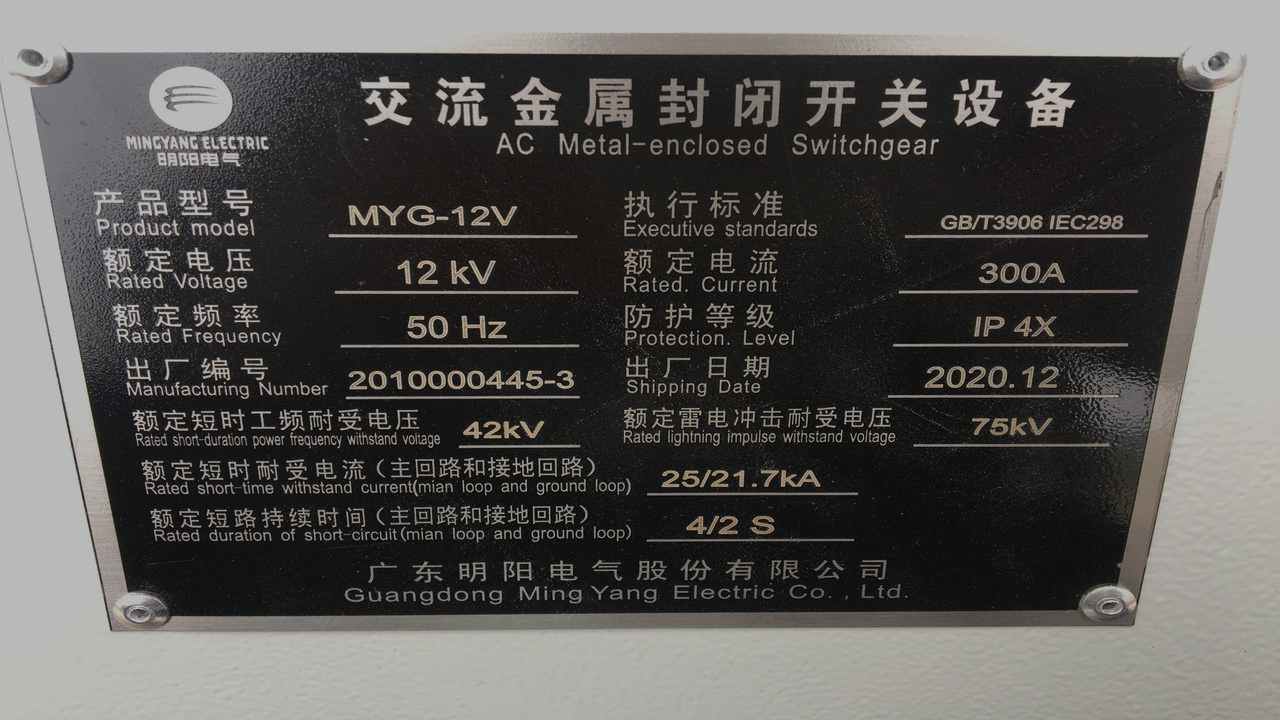

Supplement: S1 Dataset — (ZIP) [file pone.0300792.s001.zip › minimal data set/gt_img_0003_N1.0.jpg]

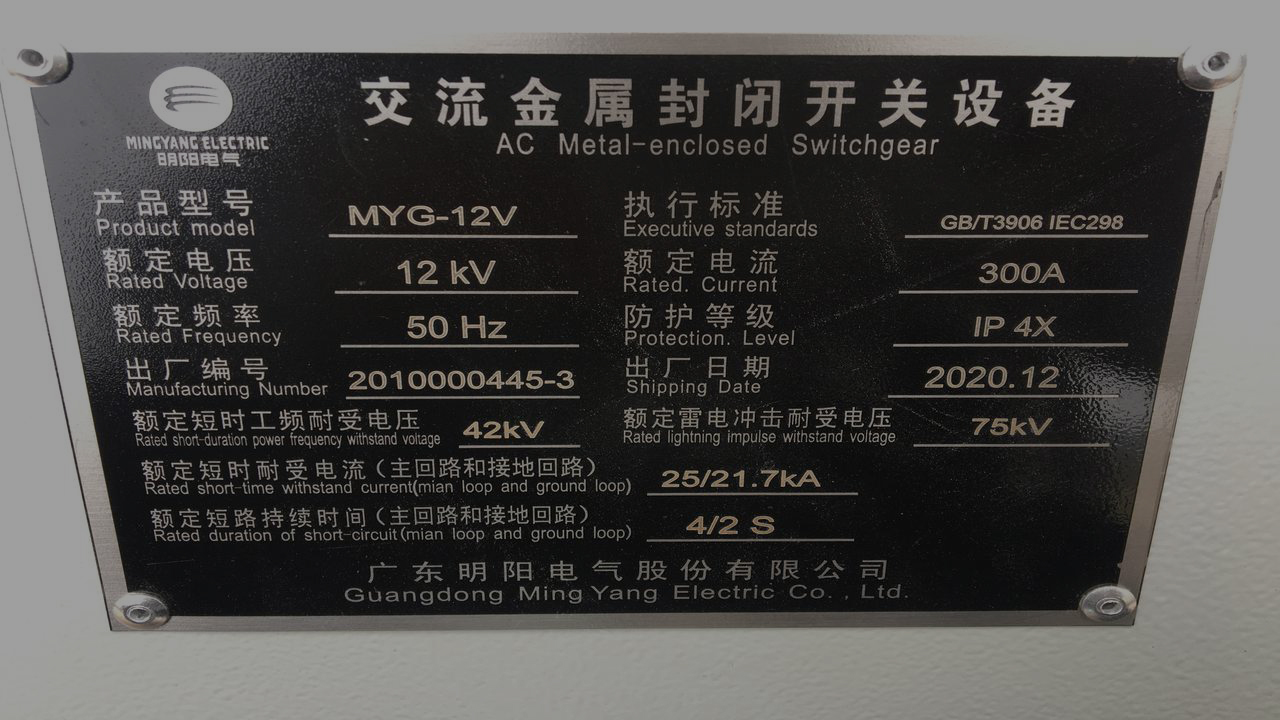

Supplement: S1 Dataset — (ZIP) [file pone.0300792.s001.zip › minimal data set/gt_img_0003_N1.5.jpg]

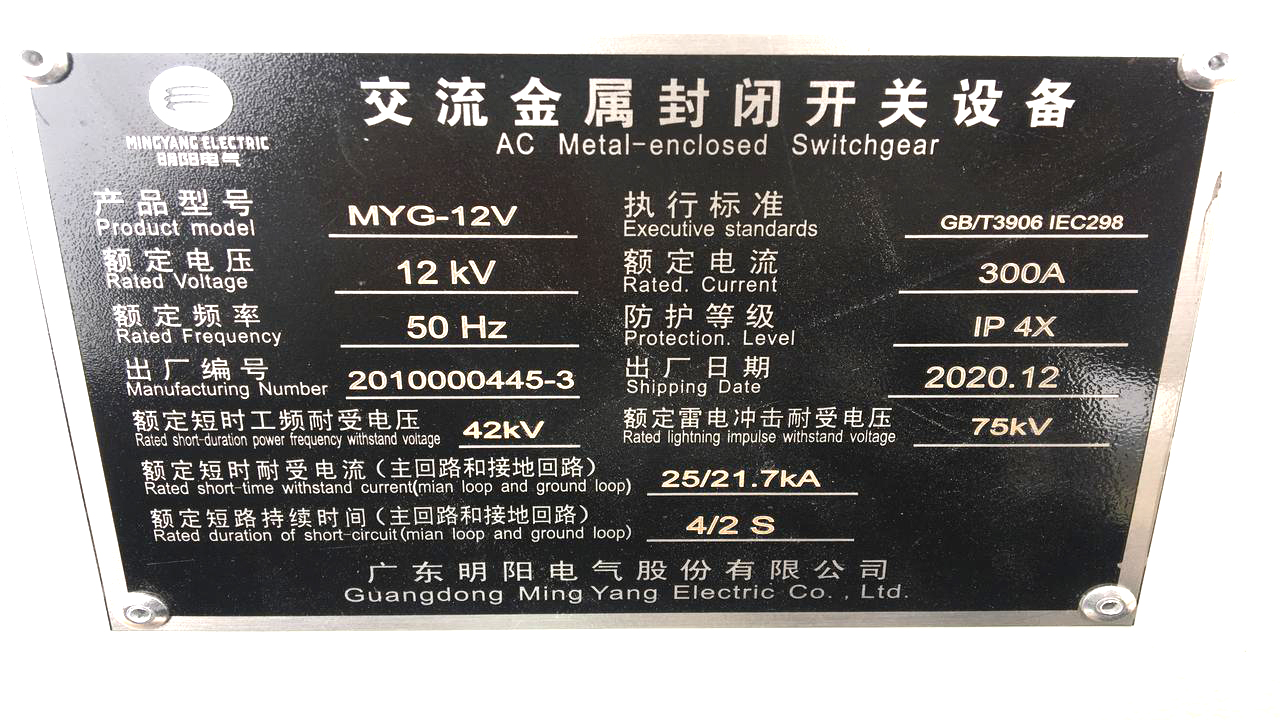

Supplement: S1 Dataset — (ZIP) [file pone.0300792.s001.zip › minimal data set/gt_img_0003_P1.0.jpg]

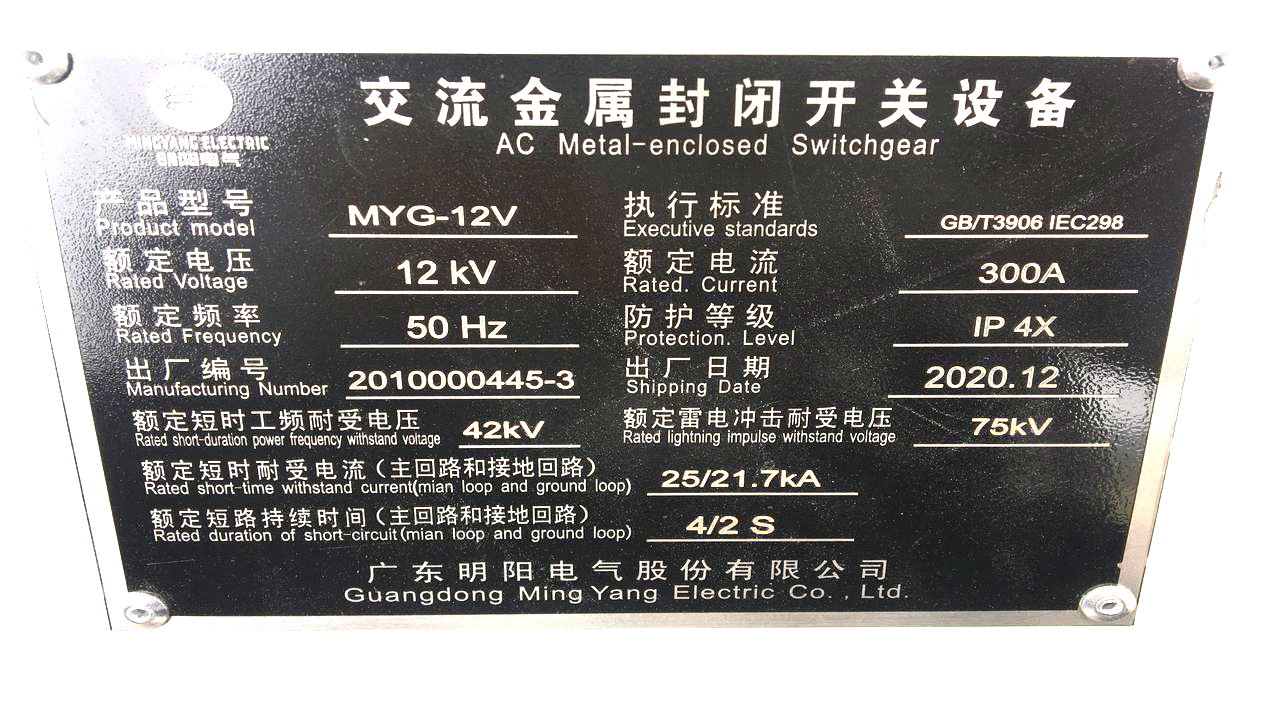

Supplement: S1 Dataset — (ZIP) [file pone.0300792.s001.zip › minimal data set/gt_img_0003_P1.5.jpg]

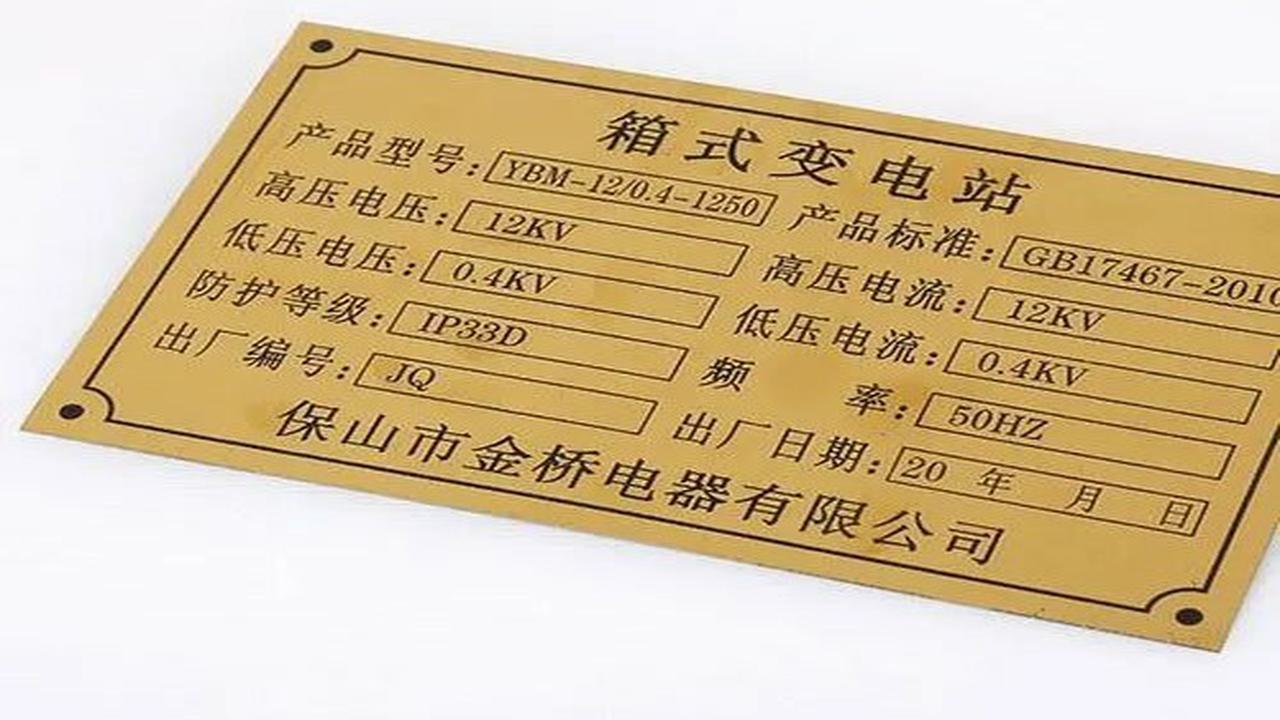

Supplement: S1 Dataset — (ZIP) [file pone.0300792.s001.zip › minimal data set/gt_img_0004_0.jpg]

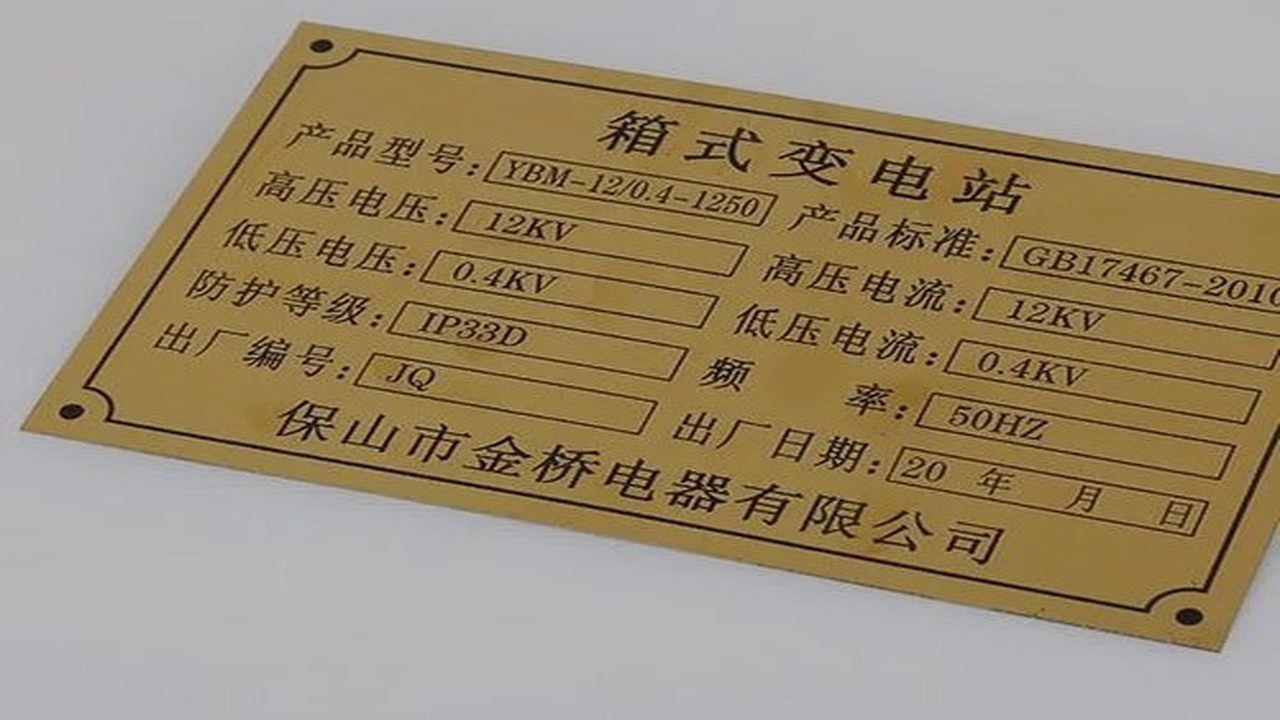

Supplement: S1 Dataset — (ZIP) [file pone.0300792.s001.zip › minimal data set/gt_img_0004_N1.0.jpg]

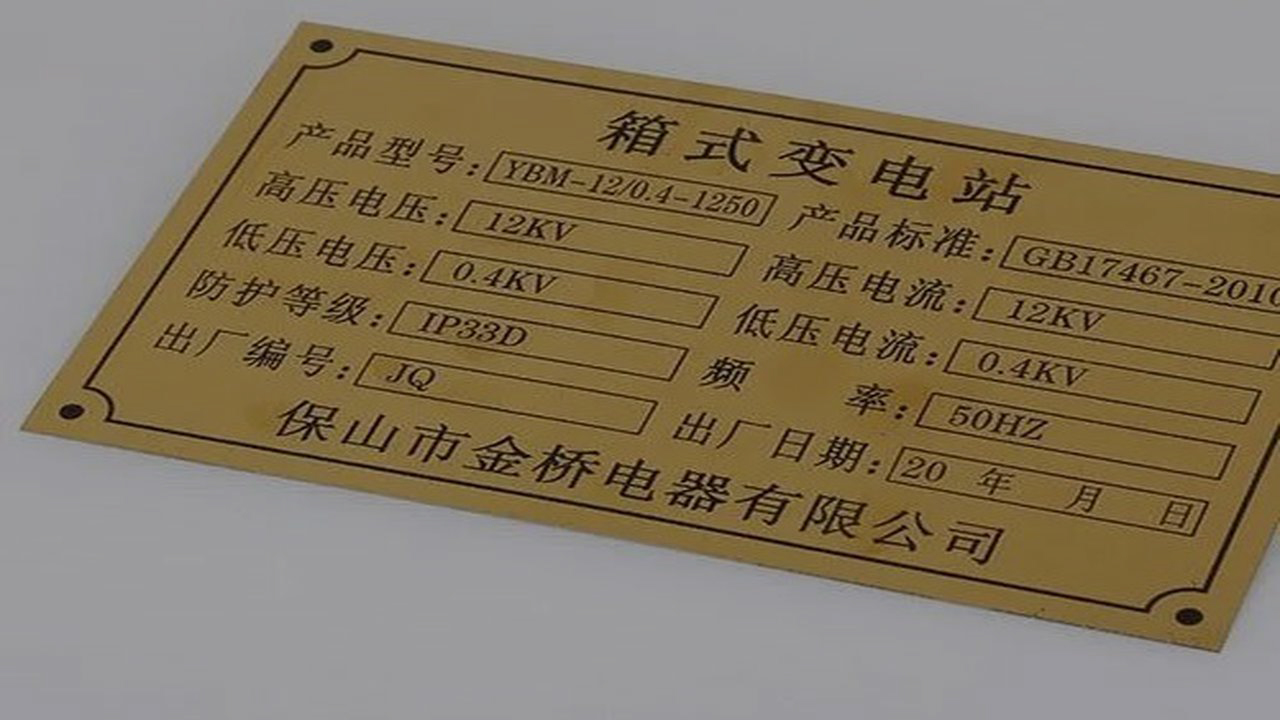

Supplement: S1 Dataset — (ZIP) [file pone.0300792.s001.zip › minimal data set/gt_img_0004_N1.5.jpg]

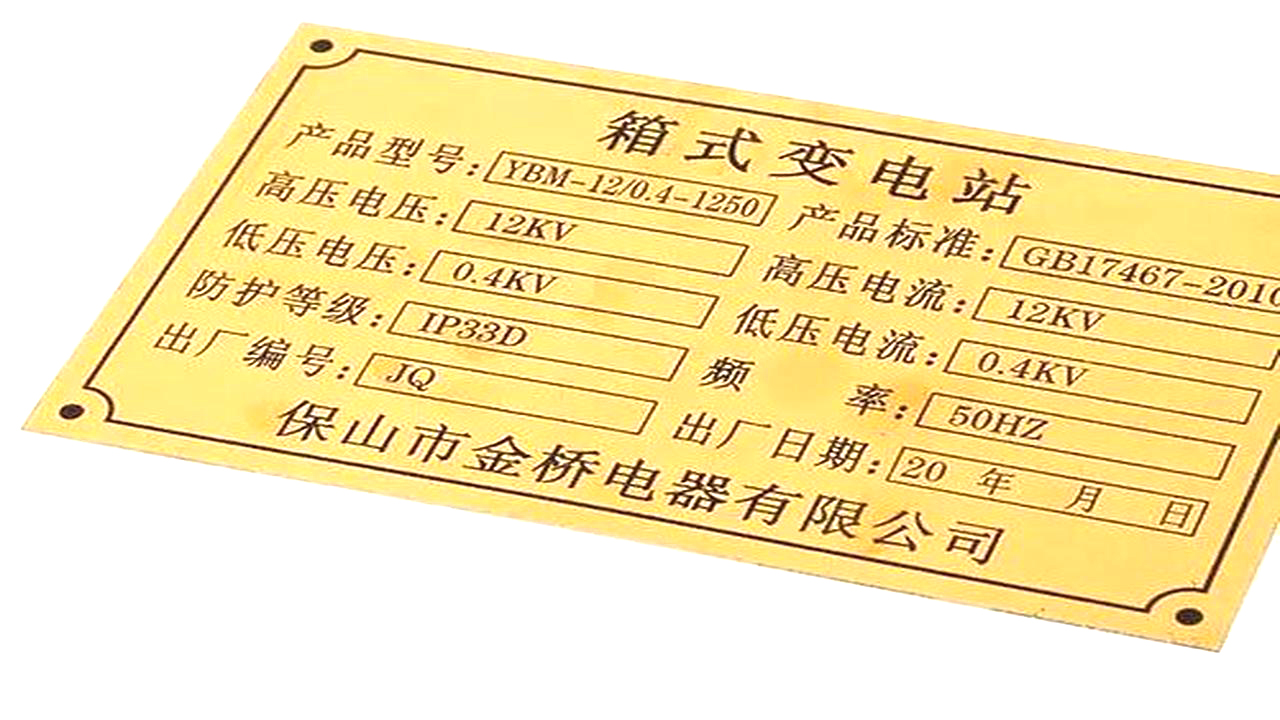

Supplement: S1 Dataset — (ZIP) [file pone.0300792.s001.zip › minimal data set/gt_img_0004_P1.0.jpg]

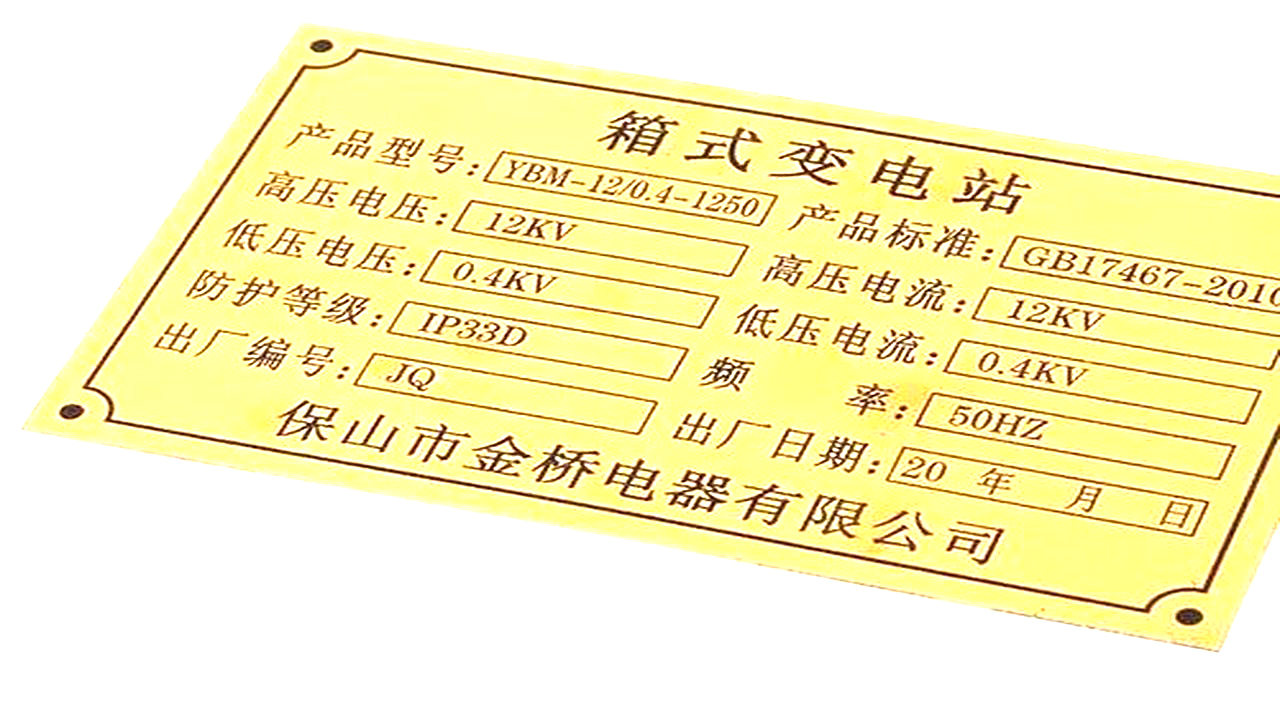

Supplement: S1 Dataset — (ZIP) [file pone.0300792.s001.zip › minimal data set/gt_img_0004_P1.5.jpg]

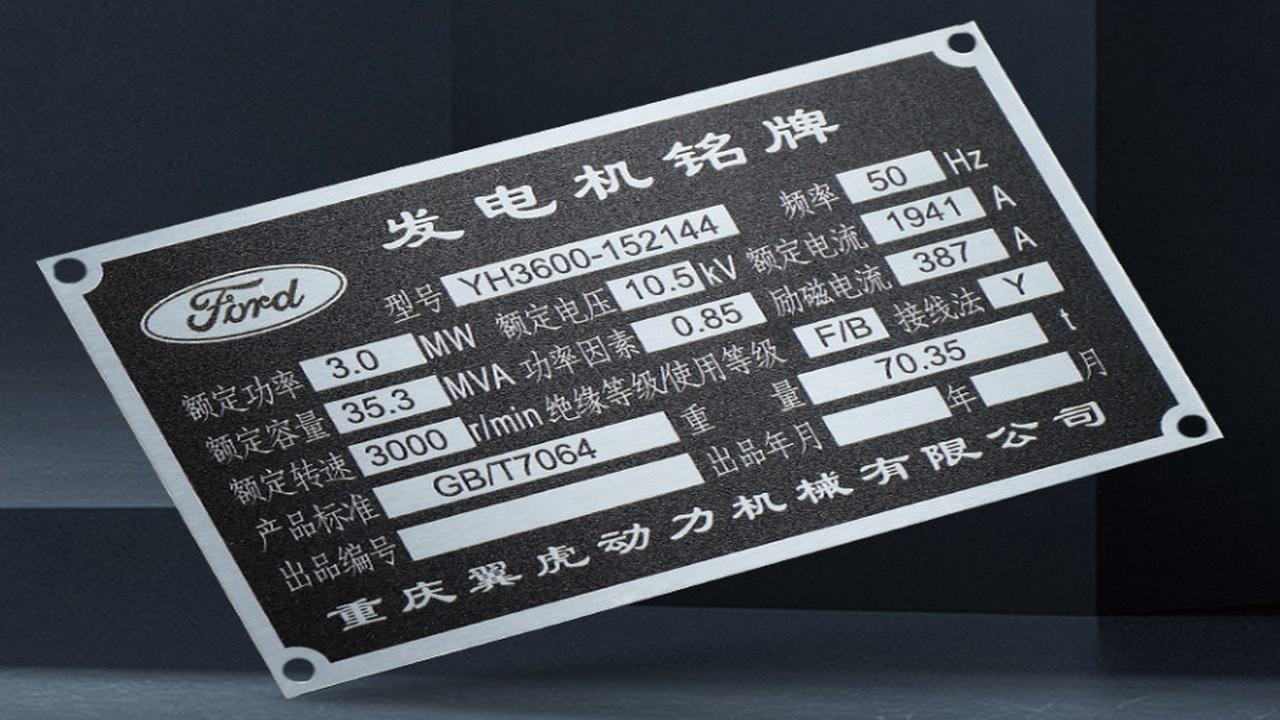

Supplement: S1 Dataset — (ZIP) [file pone.0300792.s001.zip › minimal data set/gt_img_0005_0.jpg]

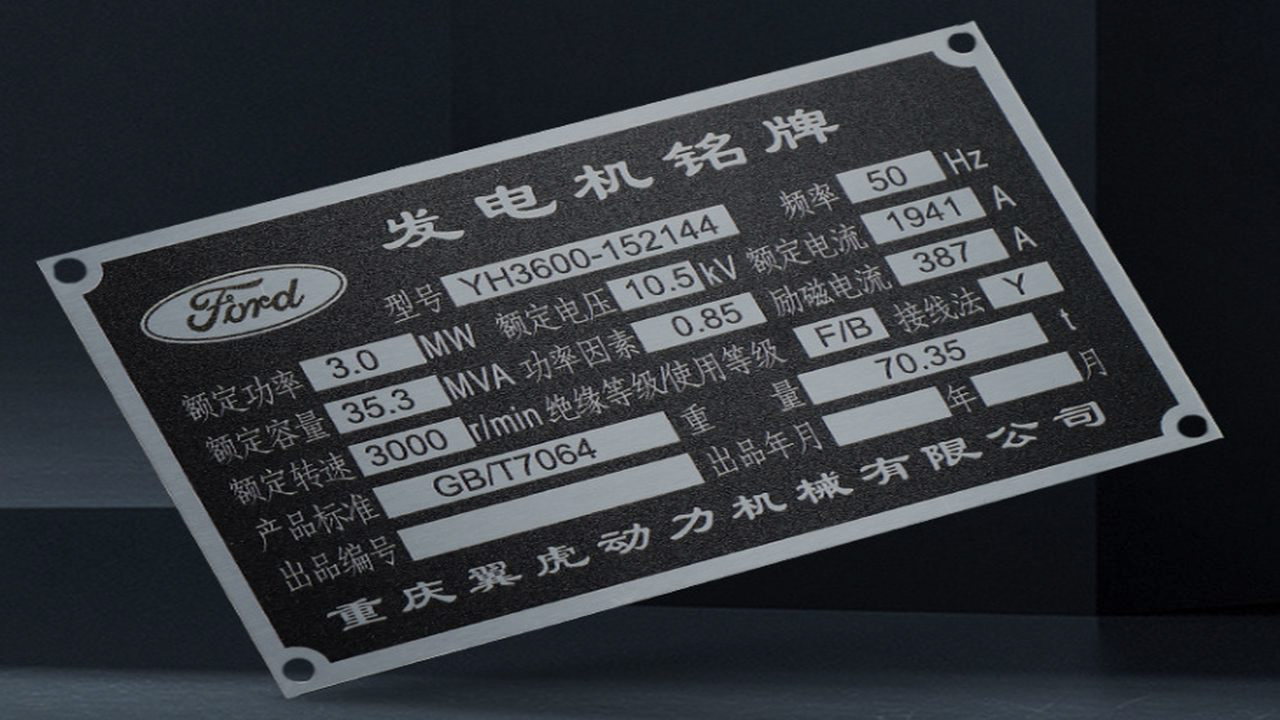

Supplement: S1 Dataset — (ZIP) [file pone.0300792.s001.zip › minimal data set/gt_img_0005_N1.0.jpg]

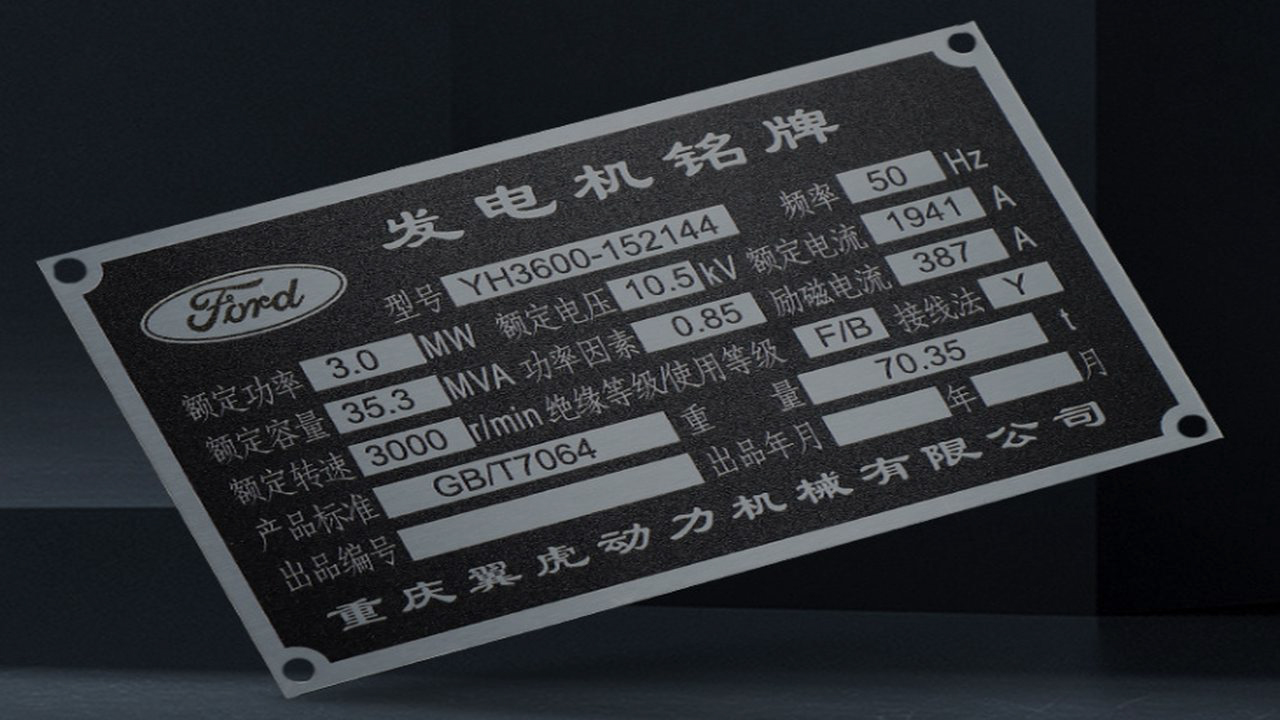

Supplement: S1 Dataset — (ZIP) [file pone.0300792.s001.zip › minimal data set/gt_img_0005_N1.5.jpg]

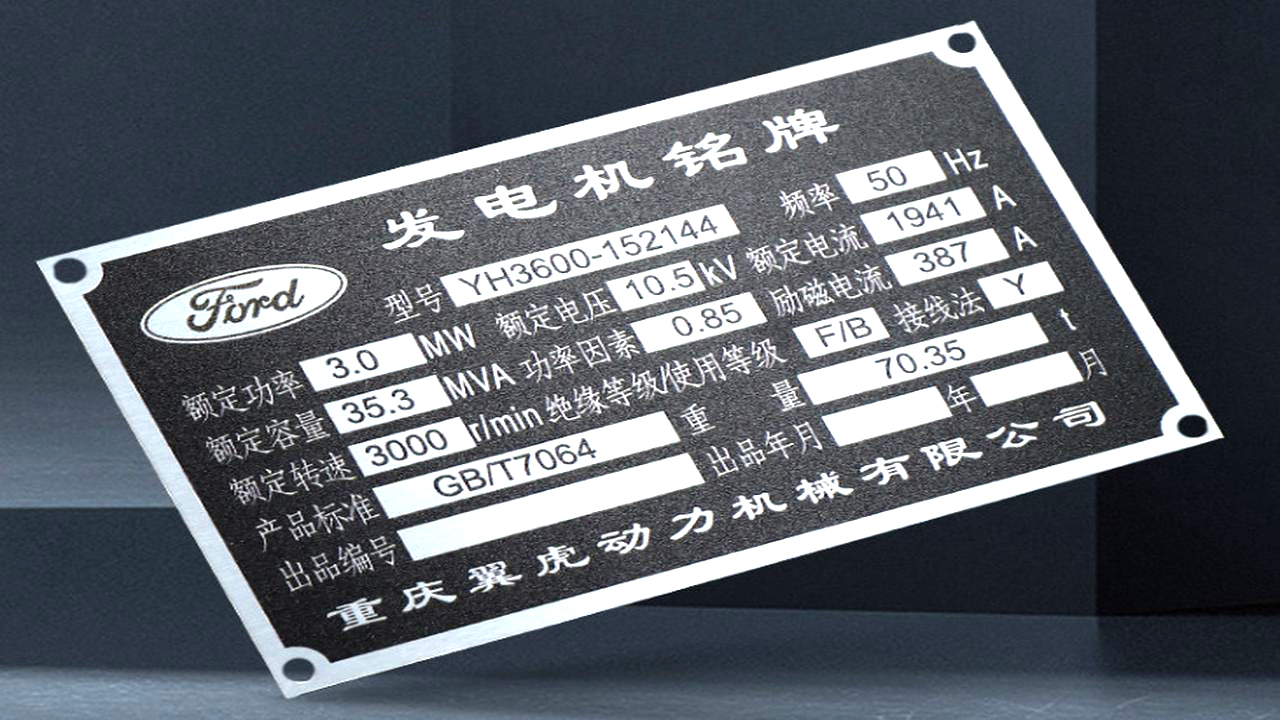

Supplement: S1 Dataset — (ZIP) [file pone.0300792.s001.zip › minimal data set/gt_img_0005_P1.0.jpg]

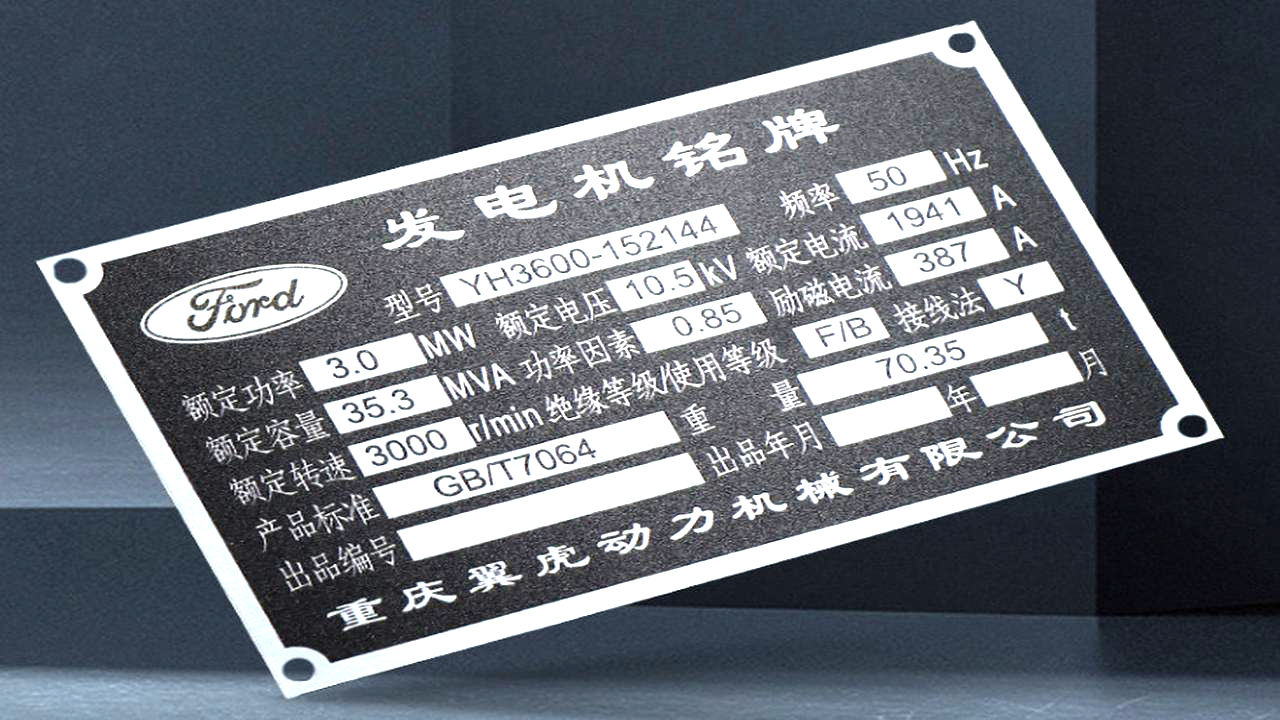

Supplement: S1 Dataset — (ZIP) [file pone.0300792.s001.zip › minimal data set/gt_img_0005_P1.5.jpg]

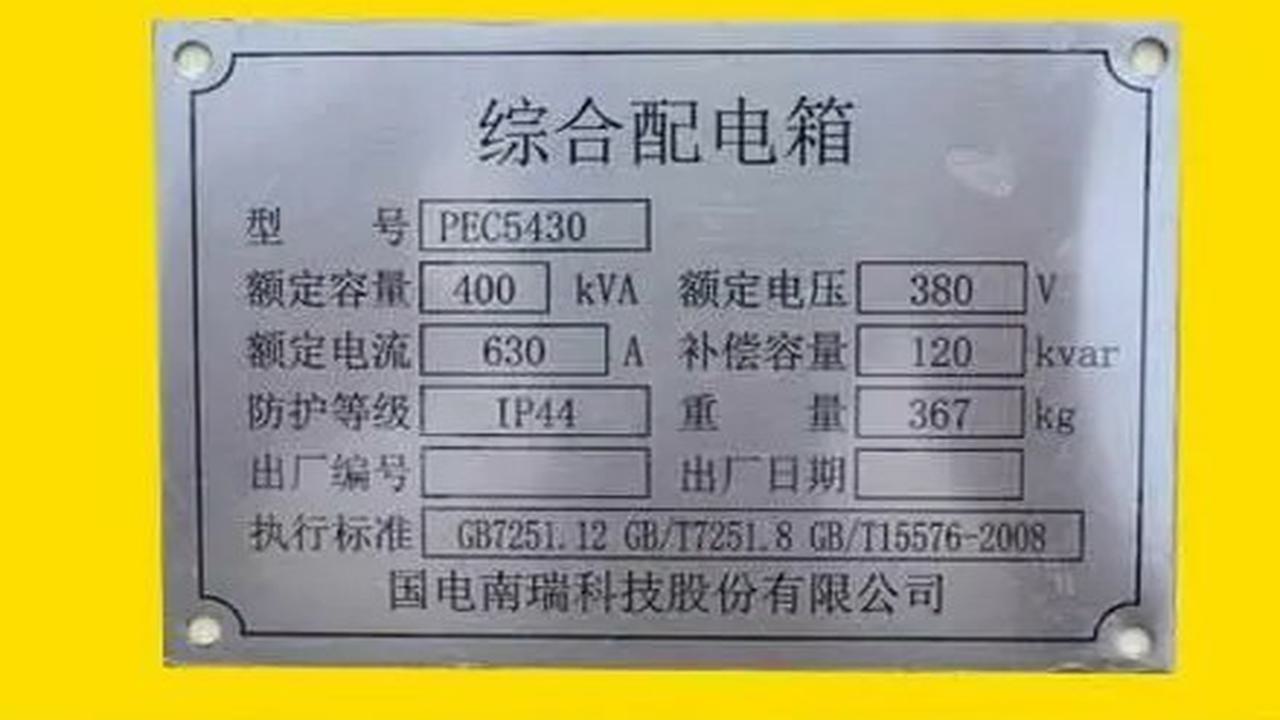

Supplement: S1 Dataset — (ZIP) [file pone.0300792.s001.zip › minimal data set/gt_img_0006_0.jpg]

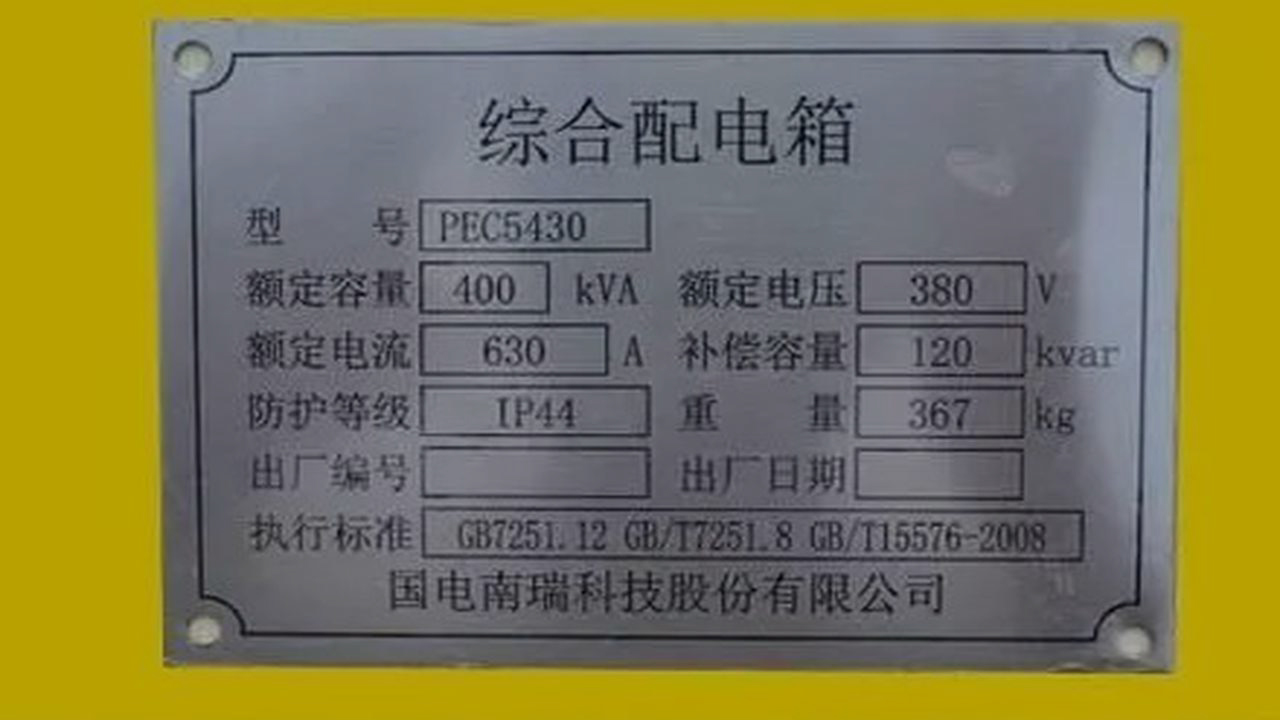

Supplement: S1 Dataset — (ZIP) [file pone.0300792.s001.zip › minimal data set/gt_img_0006_N1.0.jpg]

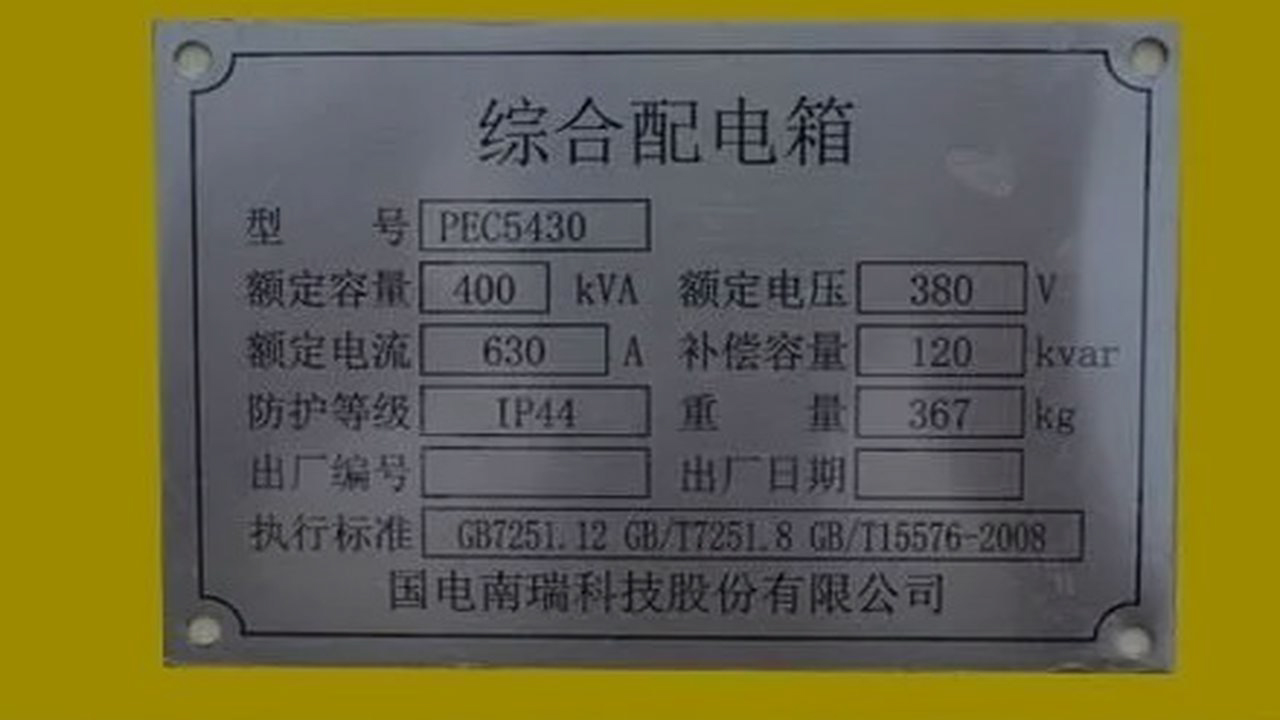

Supplement: S1 Dataset — (ZIP) [file pone.0300792.s001.zip › minimal data set/gt_img_0006_N1.5.jpg]

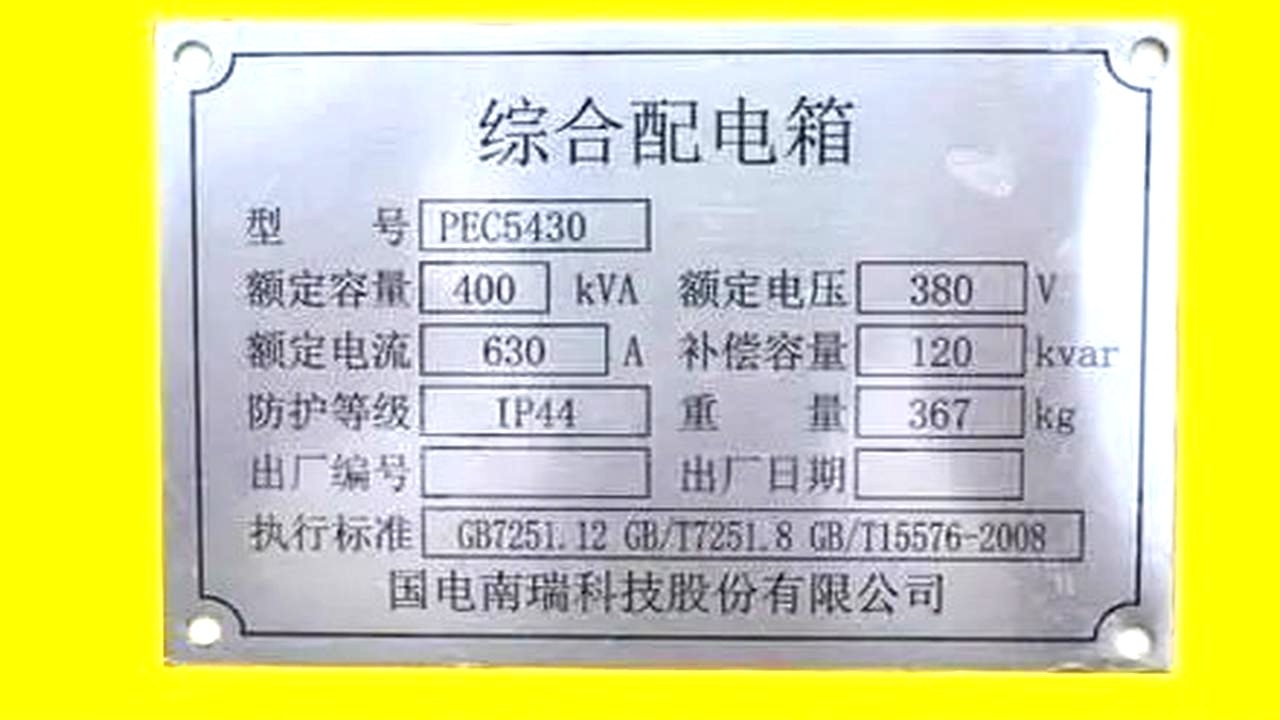

Supplement: S1 Dataset — (ZIP) [file pone.0300792.s001.zip › minimal data set/gt_img_0006_P1.0.jpg]

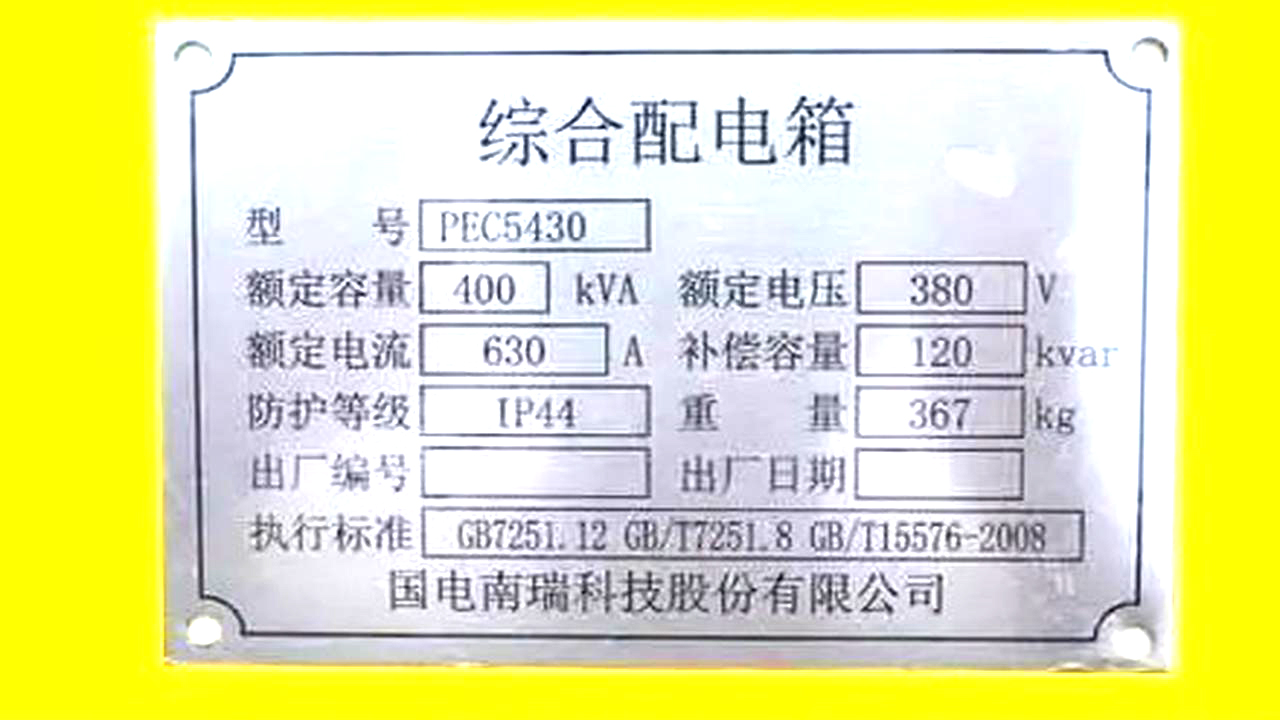

Supplement: S1 Dataset — (ZIP) [file pone.0300792.s001.zip › minimal data set/gt_img_0006_P1.5.jpg]

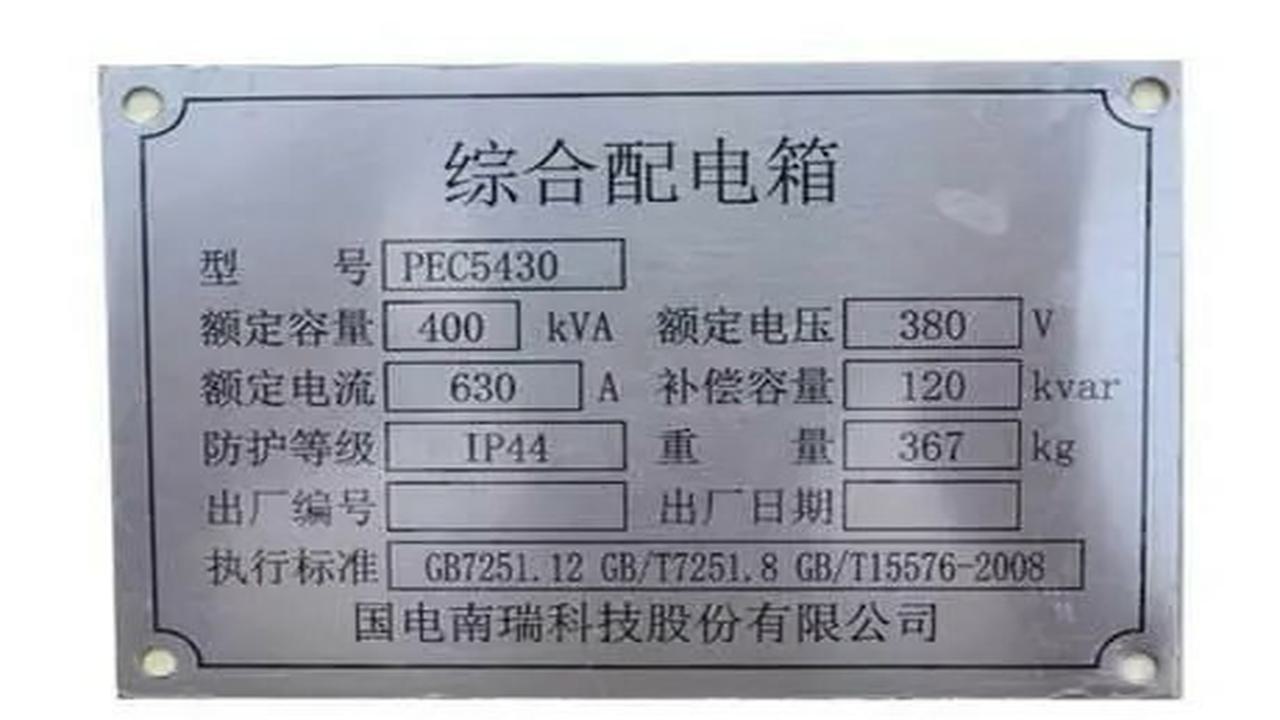

Supplement: S1 Dataset — (ZIP) [file pone.0300792.s001.zip › minimal data set/gt_img_0007_0.jpg]

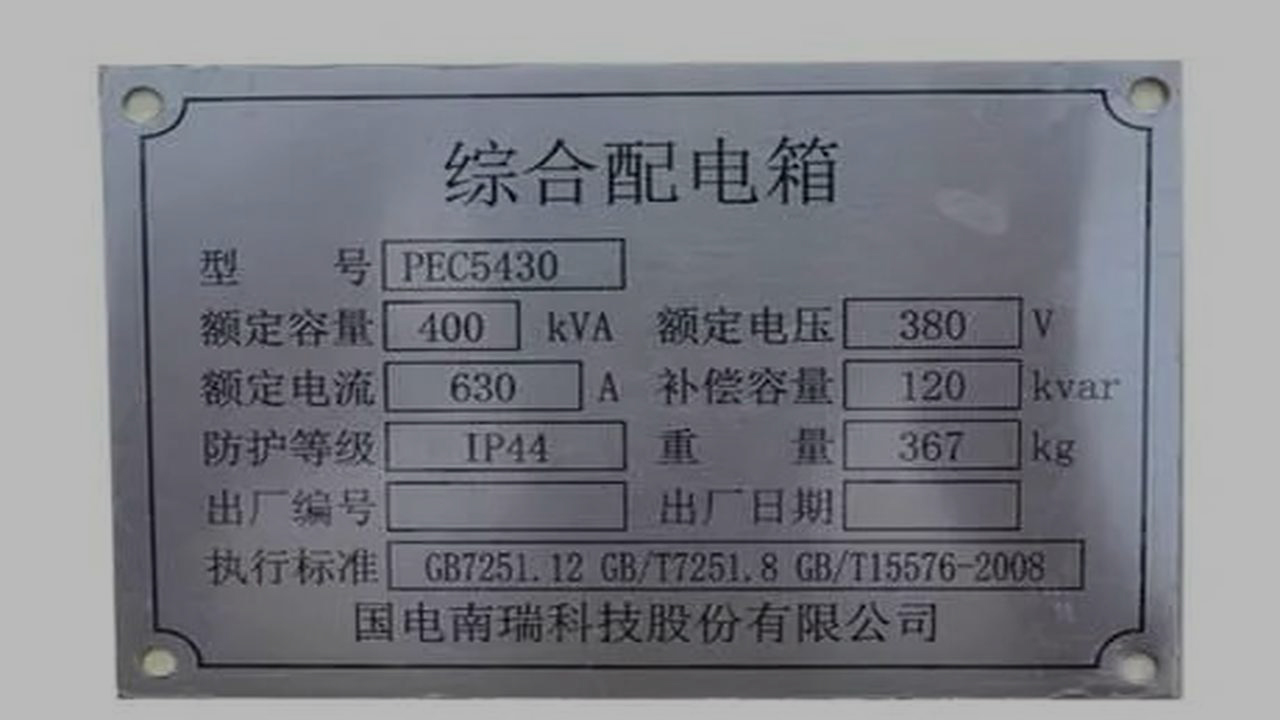

Supplement: S1 Dataset — (ZIP) [file pone.0300792.s001.zip › minimal data set/gt_img_0007_N1.0.jpg]

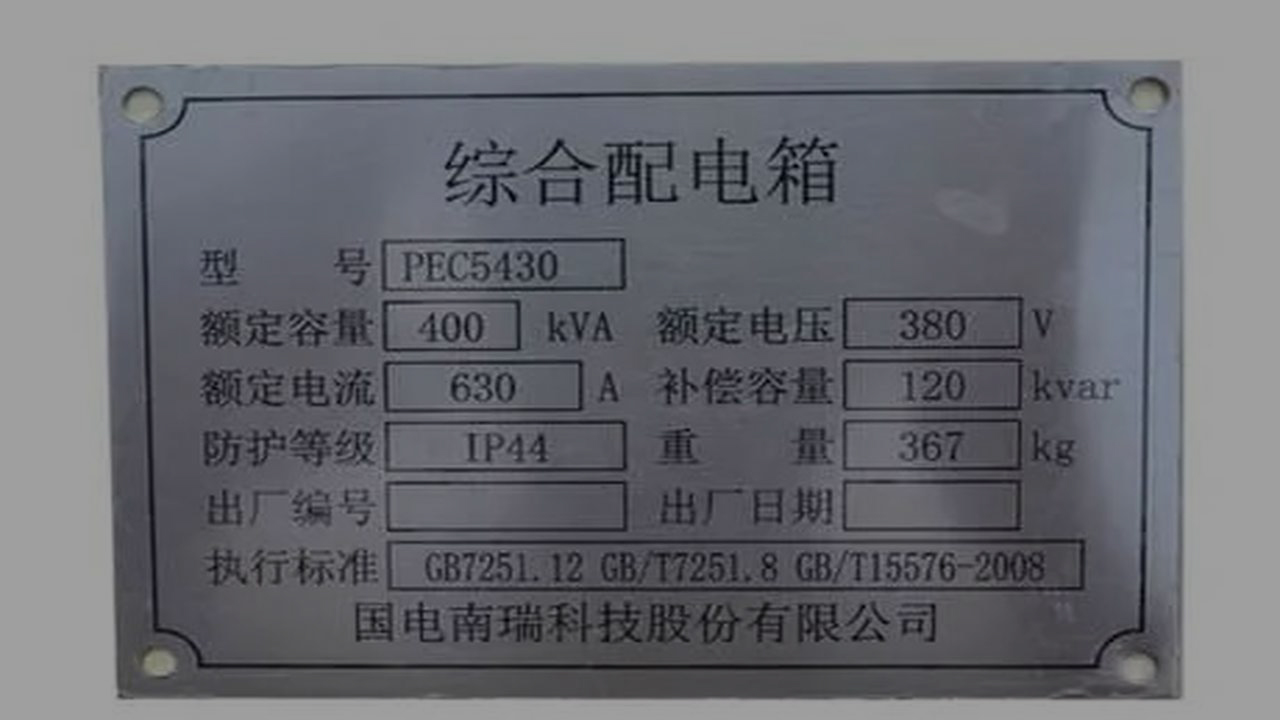

Supplement: S1 Dataset — (ZIP) [file pone.0300792.s001.zip › minimal data set/gt_img_0007_N1.5.jpg]

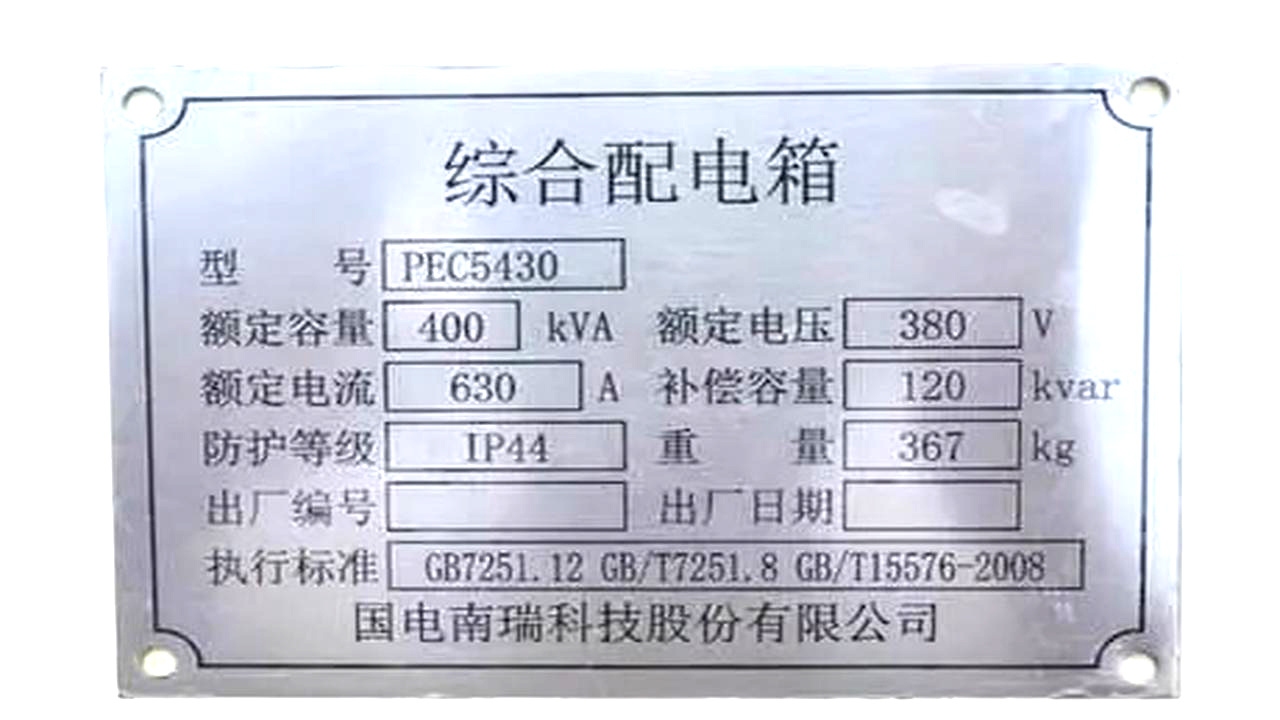

Supplement: S1 Dataset — (ZIP) [file pone.0300792.s001.zip › minimal data set/gt_img_0007_P1.0.jpg]

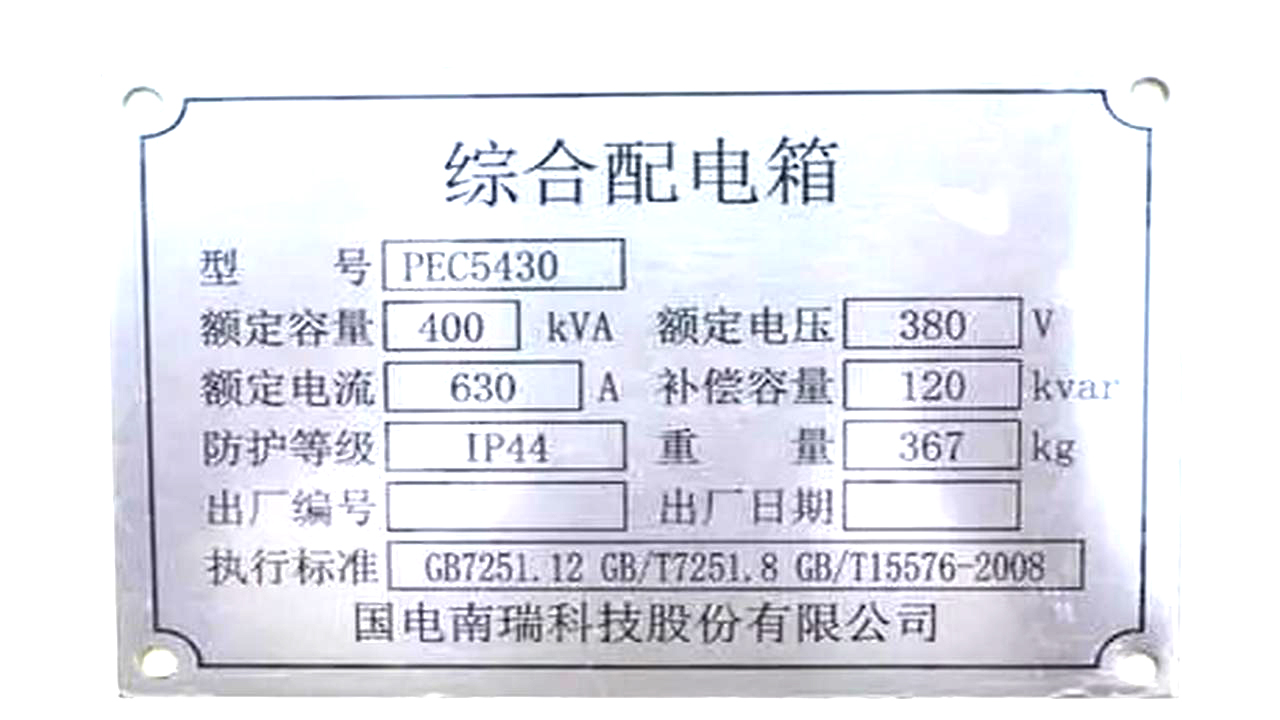

Supplement: S1 Dataset — (ZIP) [file pone.0300792.s001.zip › minimal data set/gt_img_0007_P1.5.jpg]

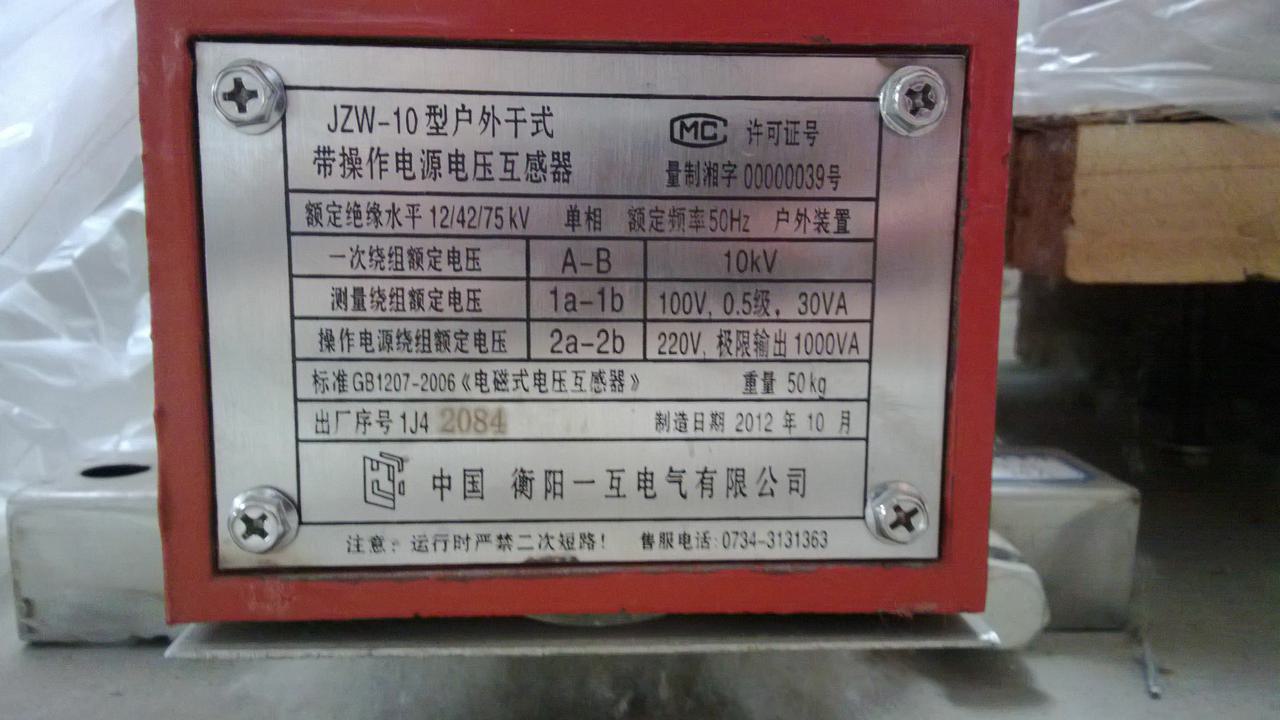

Supplement: S1 Dataset — (ZIP) [file pone.0300792.s001.zip › minimal data set/gt_img_0008_0.jpg]

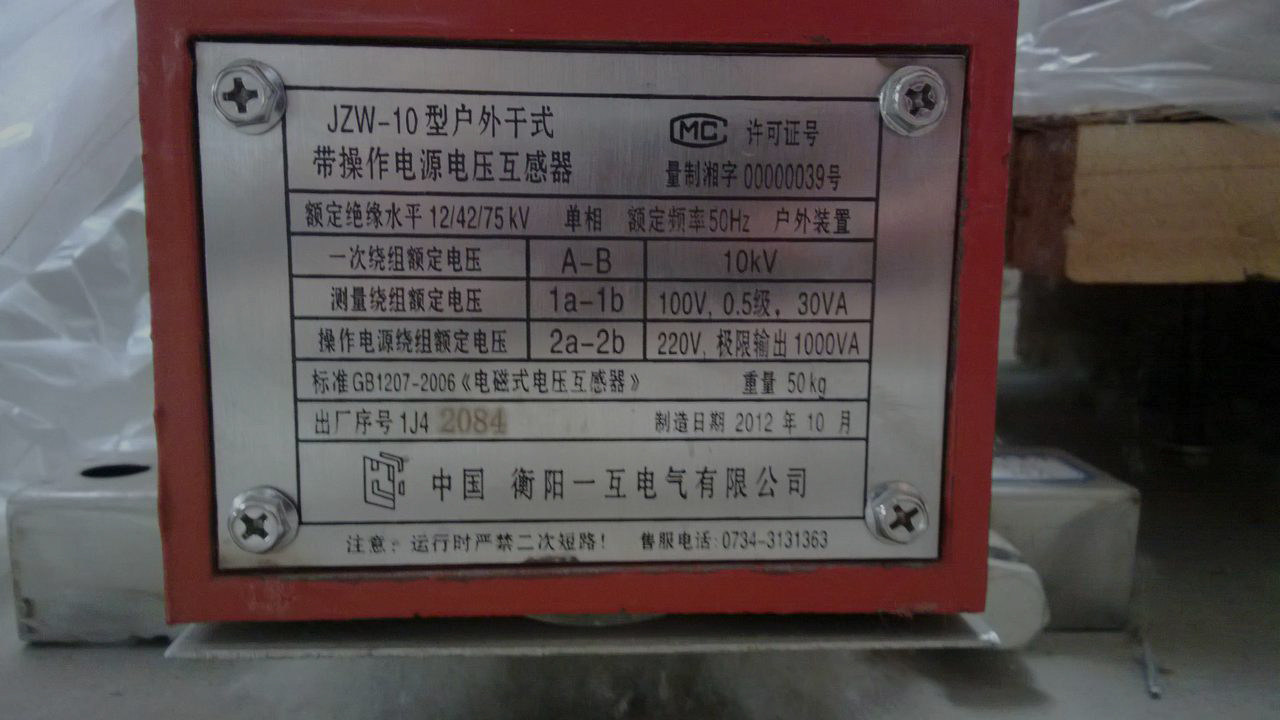

Supplement: S1 Dataset — (ZIP) [file pone.0300792.s001.zip › minimal data set/gt_img_0008_N1.0.jpg]

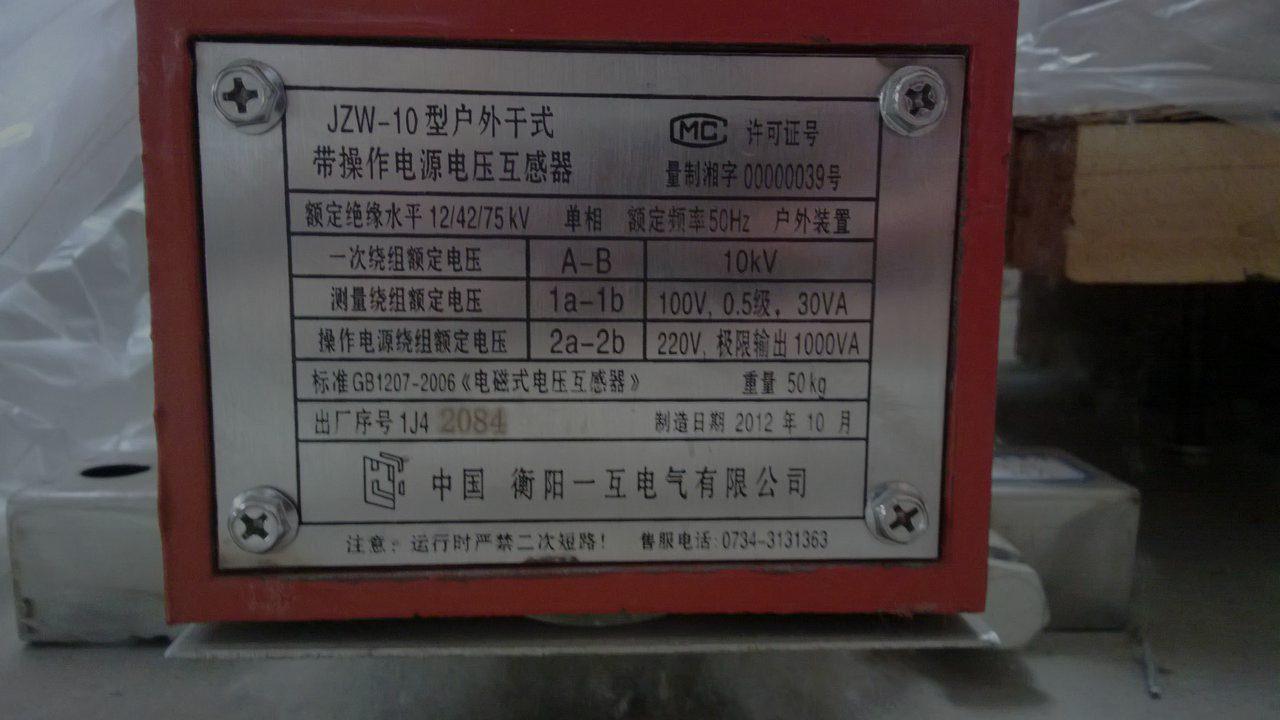

Supplement: S1 Dataset — (ZIP) [file pone.0300792.s001.zip › minimal data set/gt_img_0008_N1.5.jpg]

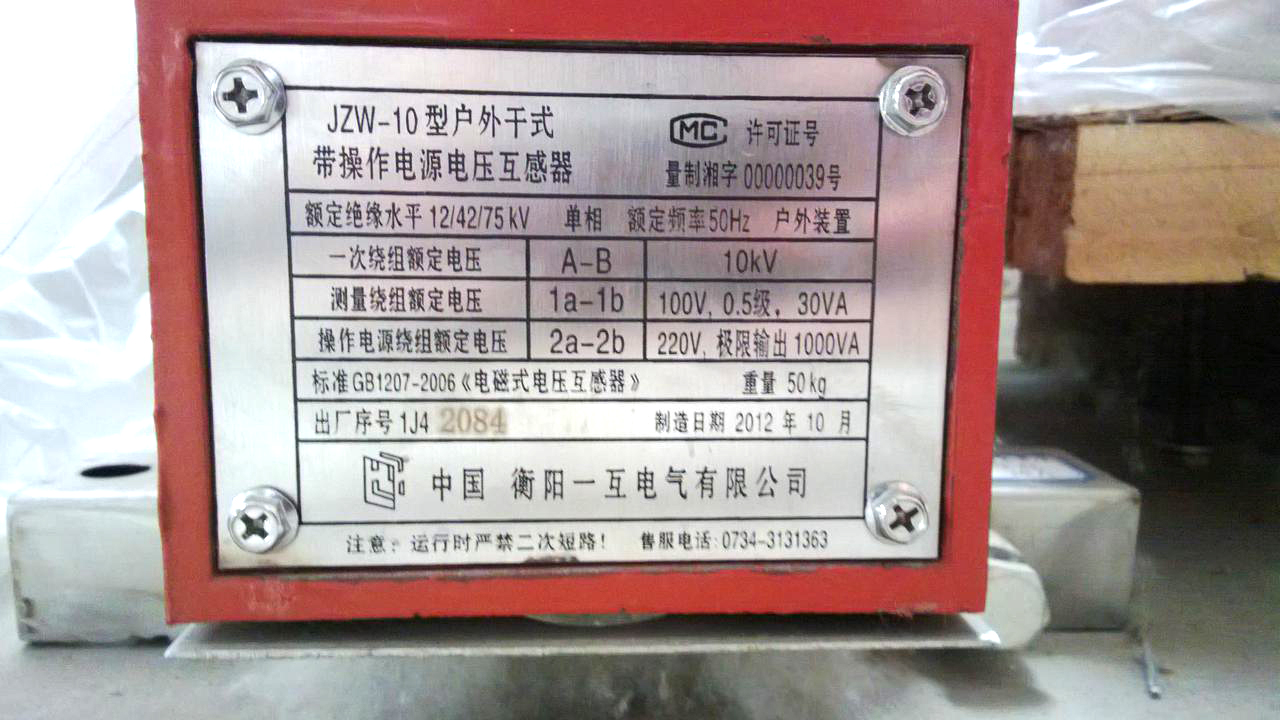

Supplement: S1 Dataset — (ZIP) [file pone.0300792.s001.zip › minimal data set/gt_img_0008_P1.0.jpg]

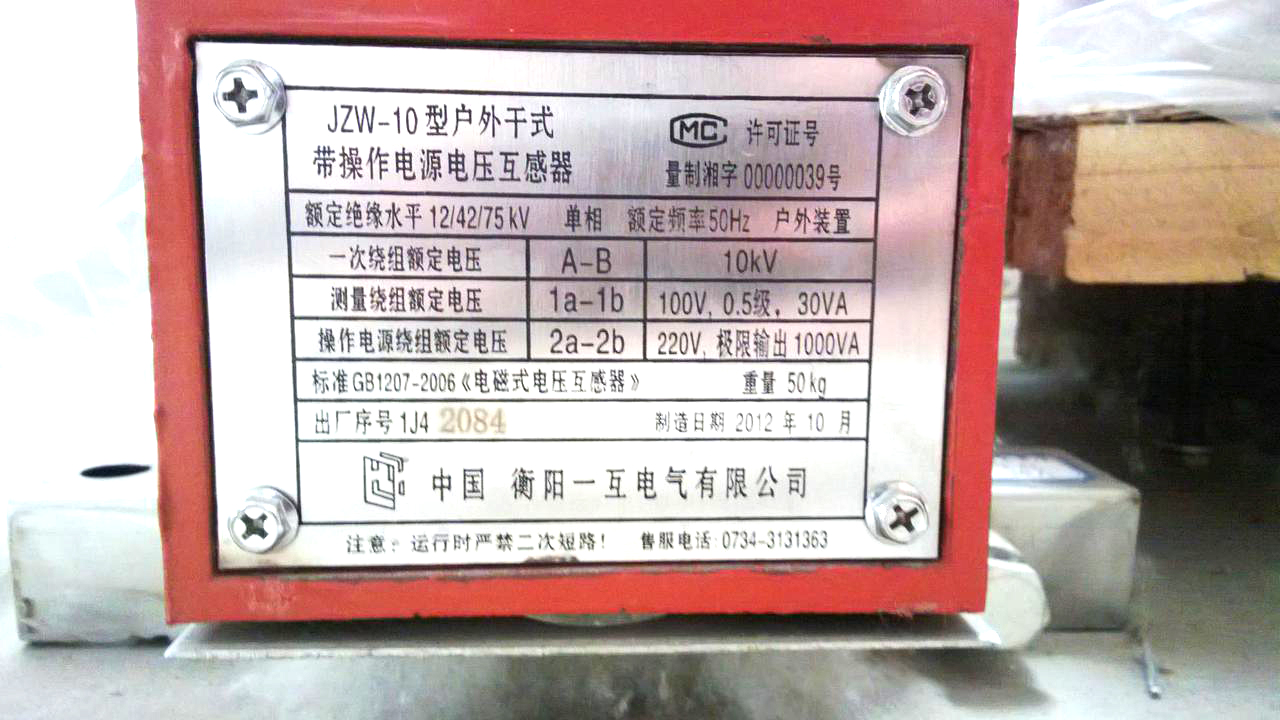

Supplement: S1 Dataset — (ZIP) [file pone.0300792.s001.zip › minimal data set/gt_img_0008_P1.5.jpg]

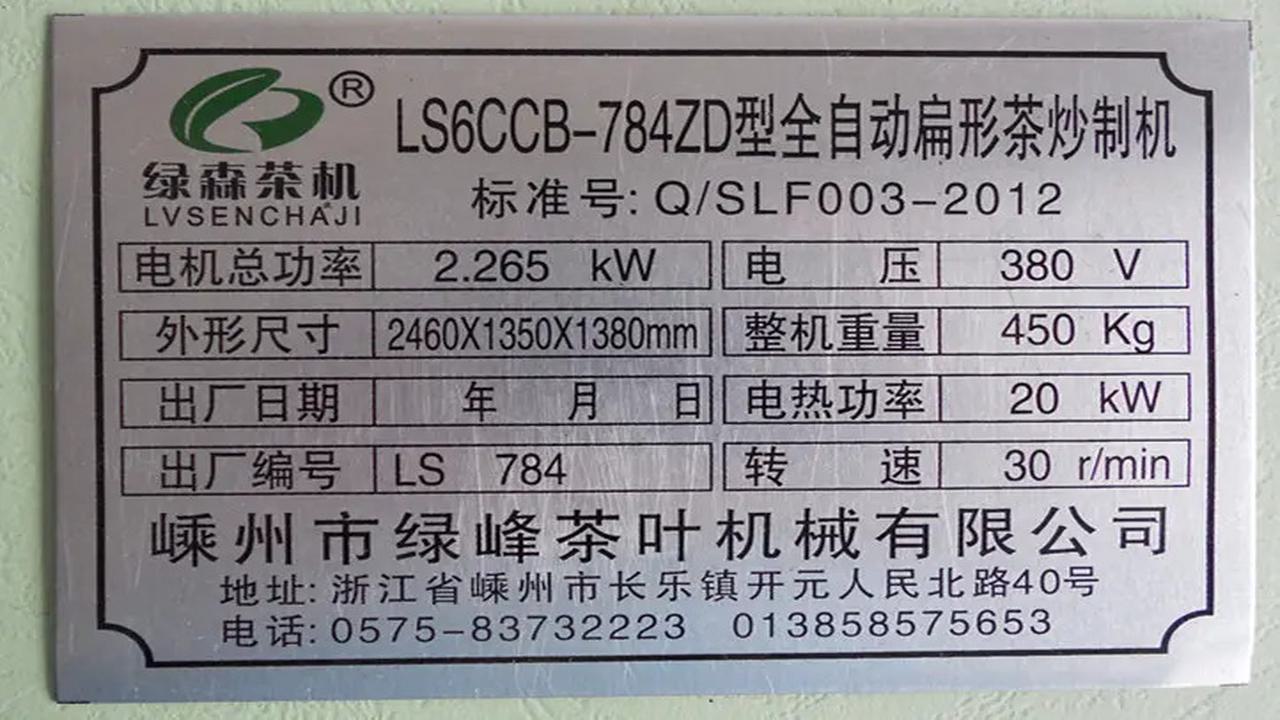

Supplement: S1 Dataset — (ZIP) [file pone.0300792.s001.zip › minimal data set/gt_img_0009_0.jpg]

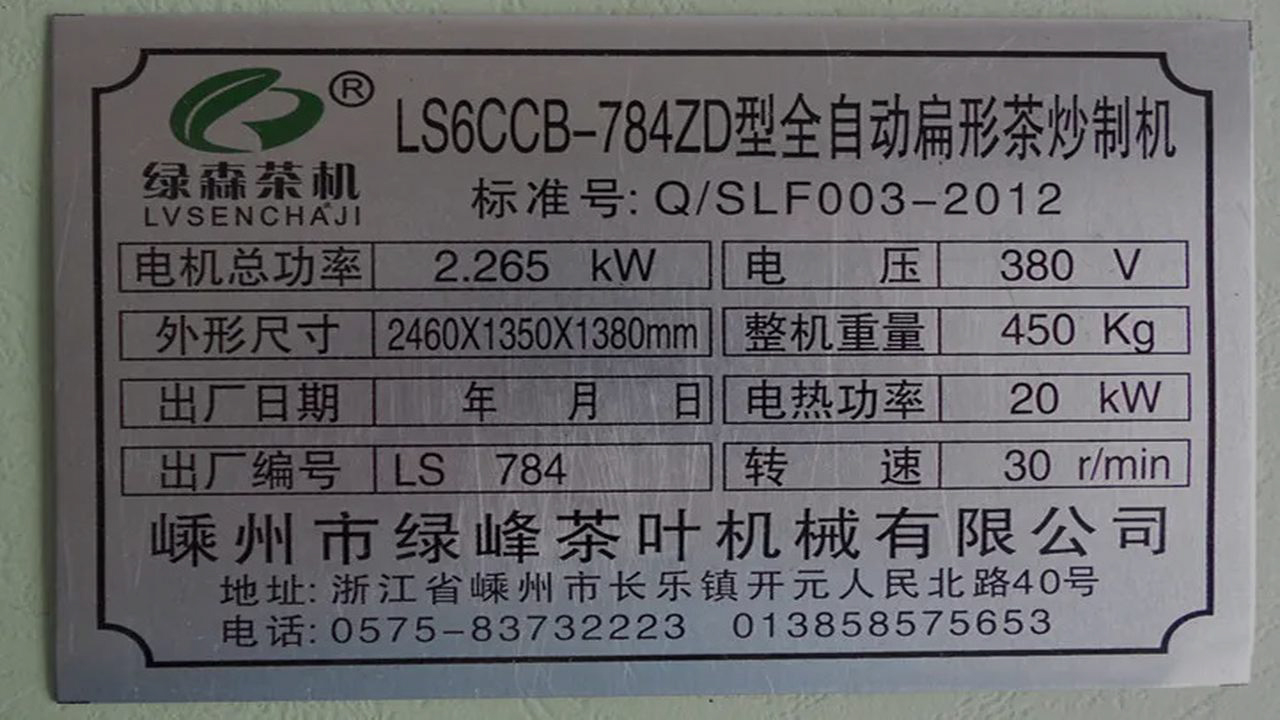

Supplement: S1 Dataset — (ZIP) [file pone.0300792.s001.zip › minimal data set/gt_img_0009_N1.0.jpg]

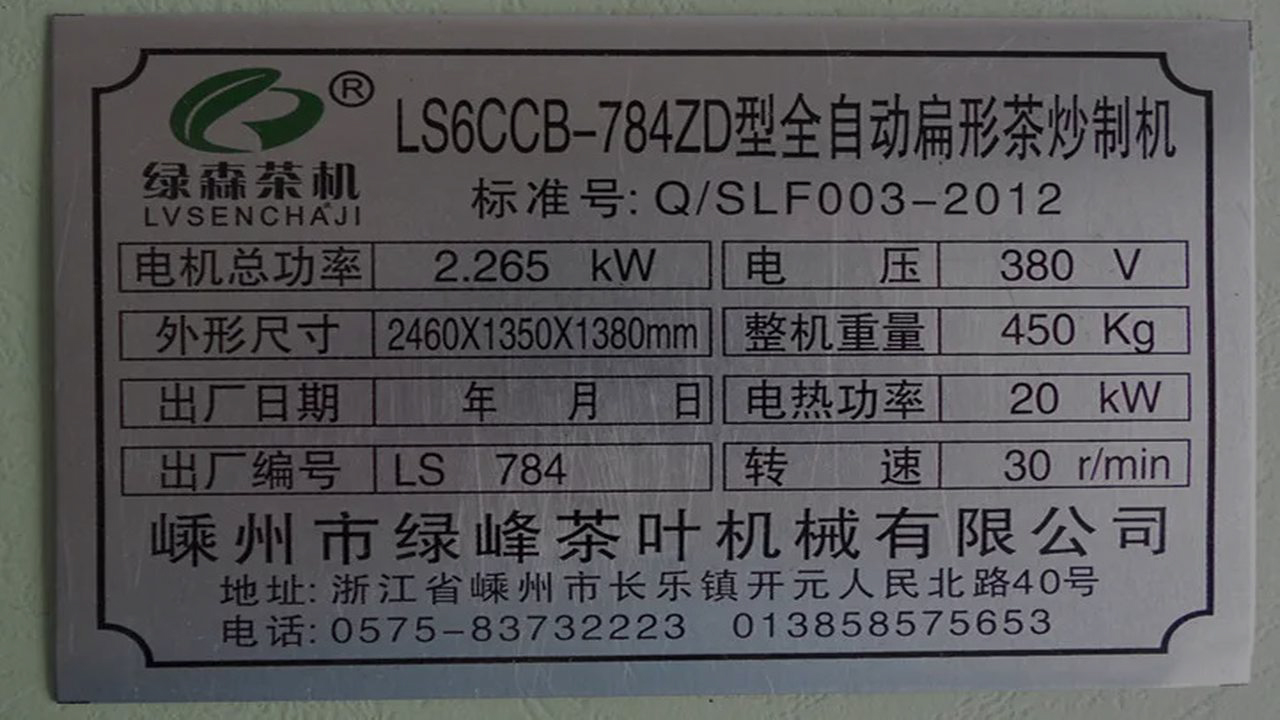

Supplement: S1 Dataset — (ZIP) [file pone.0300792.s001.zip › minimal data set/gt_img_0009_N1.5.jpg]

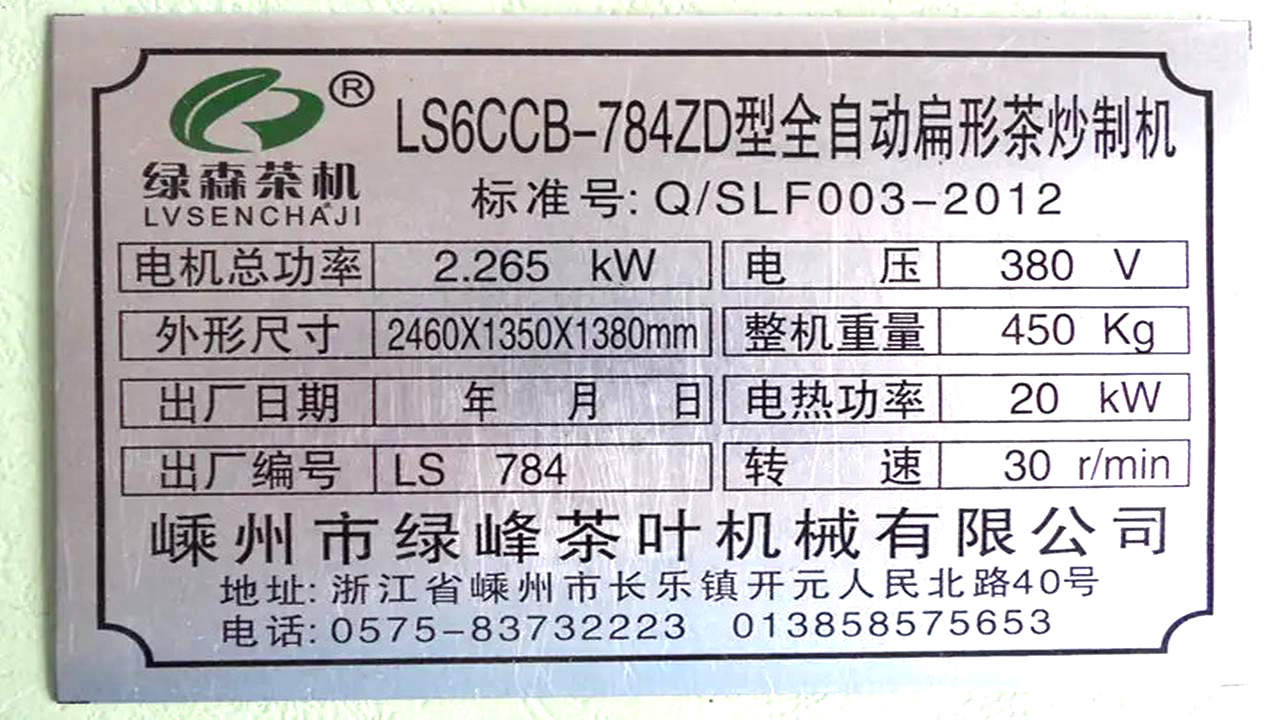

Supplement: S1 Dataset — (ZIP) [file pone.0300792.s001.zip › minimal data set/gt_img_0009_P1.0.jpg]

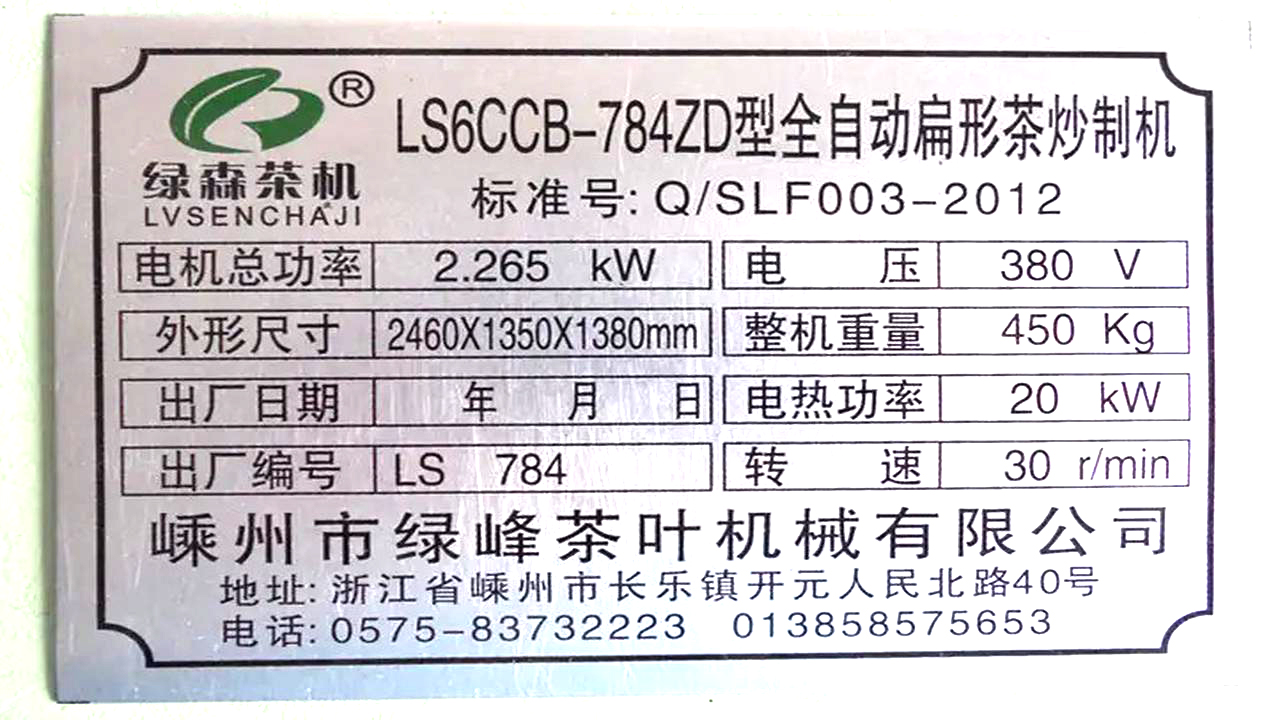

Supplement: S1 Dataset — (ZIP) [file pone.0300792.s001.zip › minimal data set/gt_img_0009_P1.5.jpg]

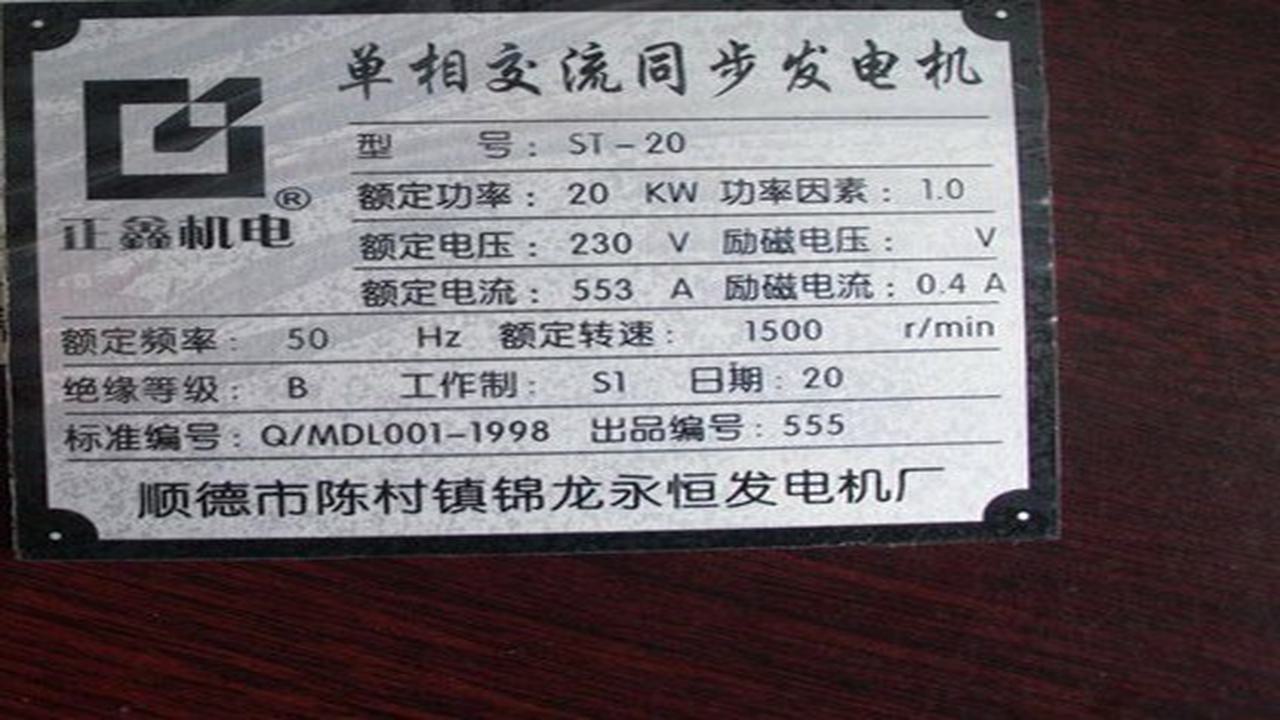

Supplement: S1 Dataset — (ZIP) [file pone.0300792.s001.zip › minimal data set/gt_img_0010_0.jpg]

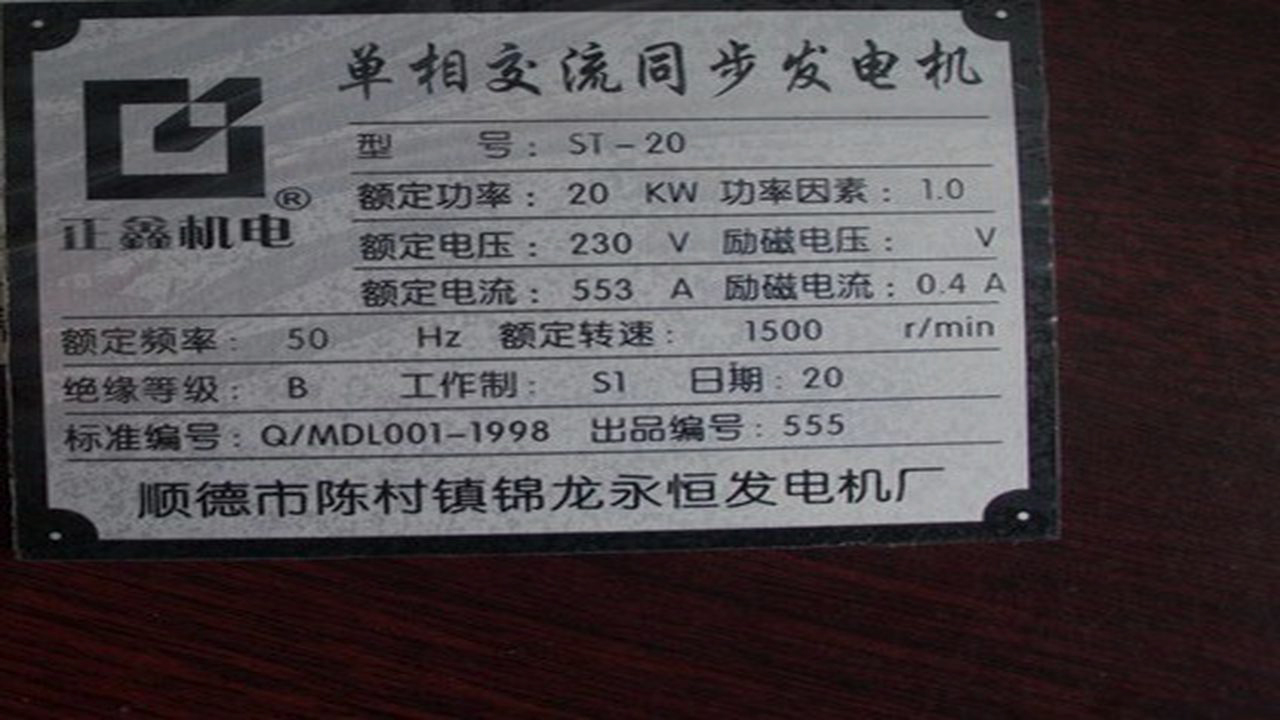

Supplement: S1 Dataset — (ZIP) [file pone.0300792.s001.zip › minimal data set/gt_img_0010_N1.0.jpg]

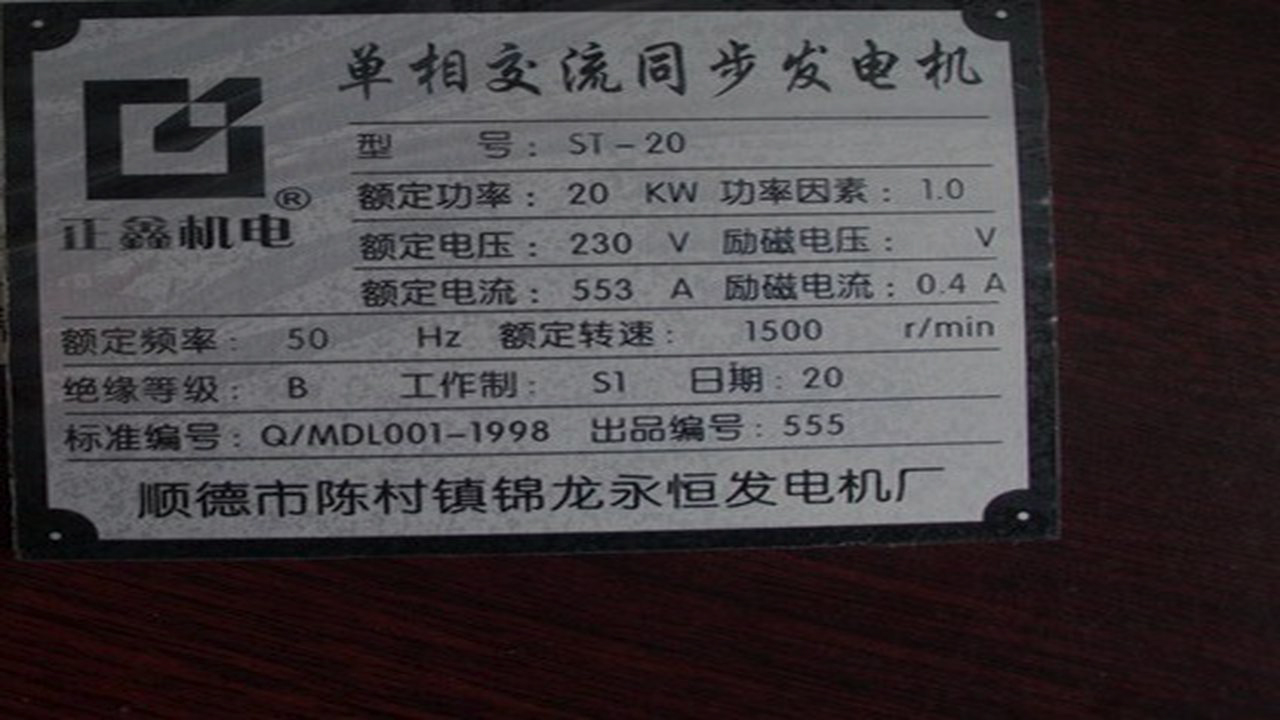

Supplement: S1 Dataset — (ZIP) [file pone.0300792.s001.zip › minimal data set/gt_img_0010_N1.5.jpg]

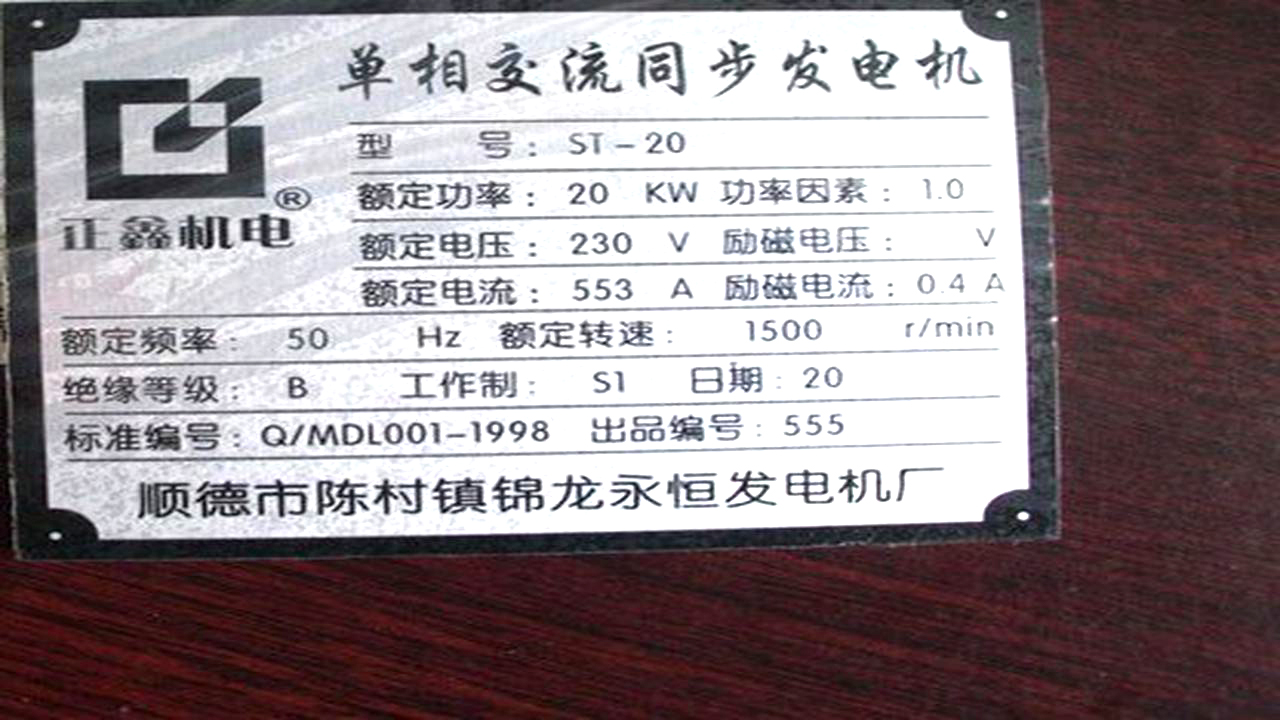

Supplement: S1 Dataset — (ZIP) [file pone.0300792.s001.zip › minimal data set/gt_img_0010_P1.0.jpg]

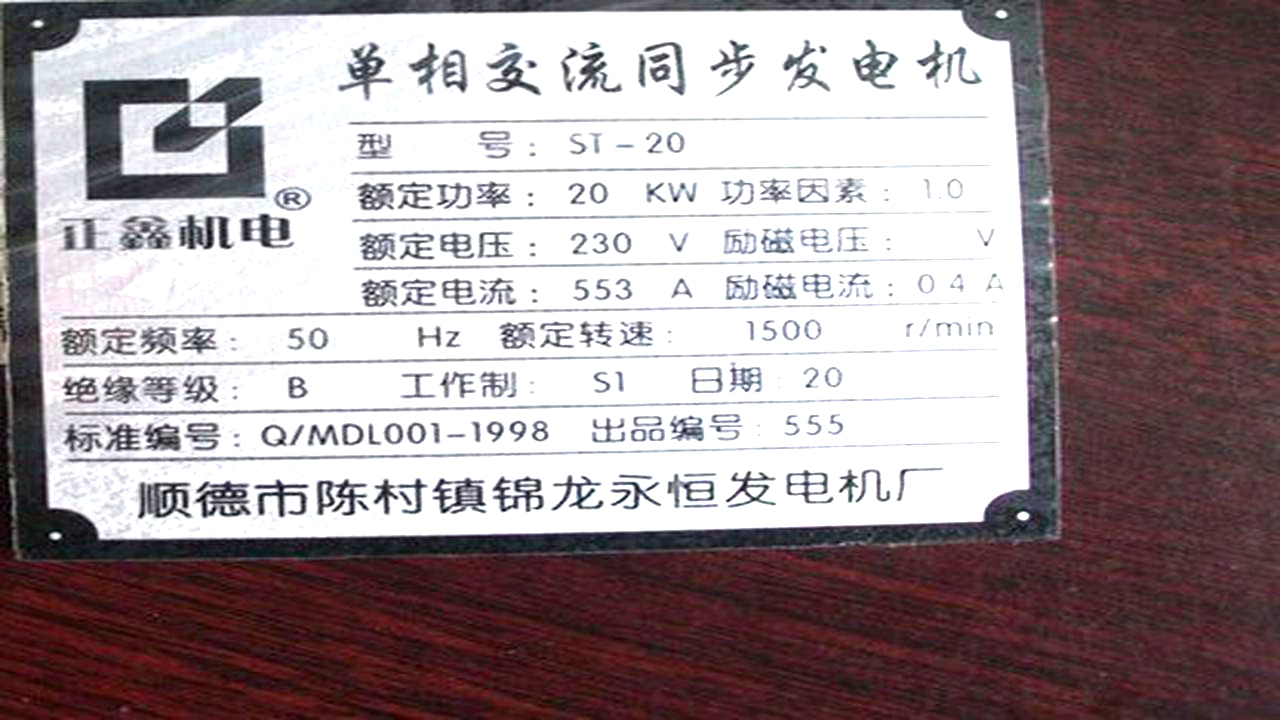

Supplement: S1 Dataset — (ZIP) [file pone.0300792.s001.zip › minimal data set/gt_img_0010_P1.5.jpg]

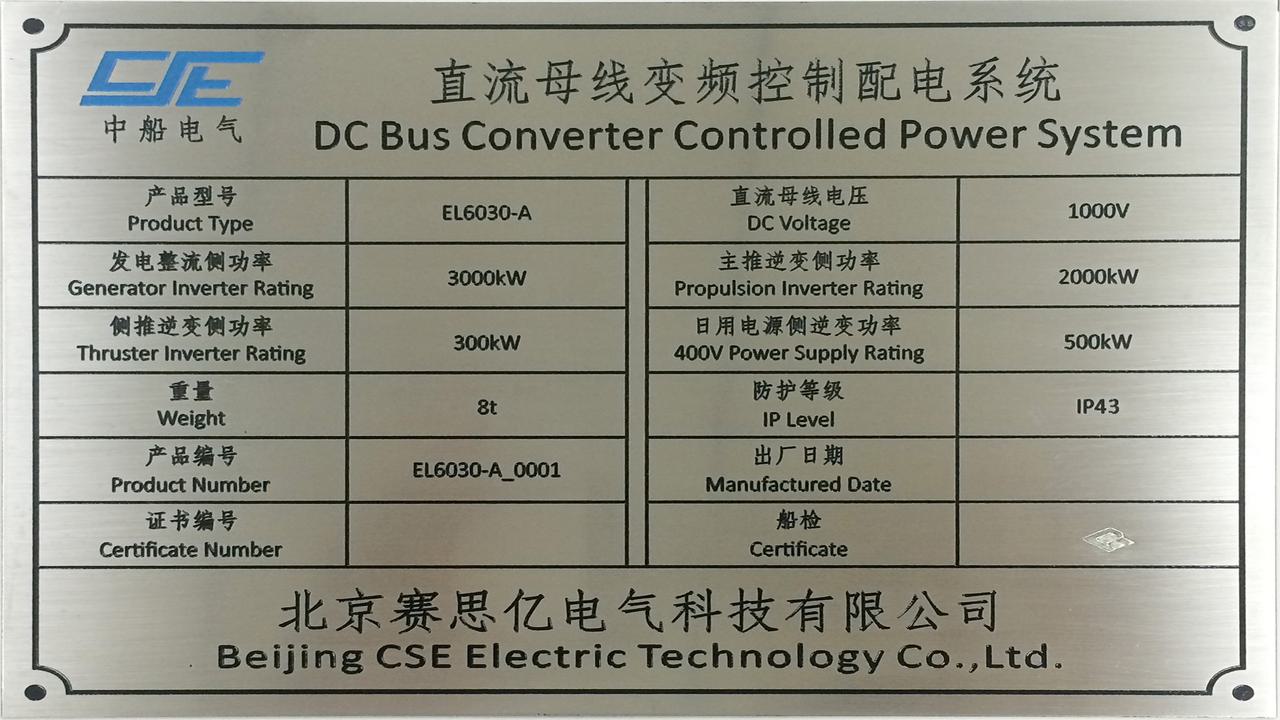

Supplement: S1 Dataset — (ZIP) [file pone.0300792.s001.zip › minimal data set/gt_img_0011_0.jpg]

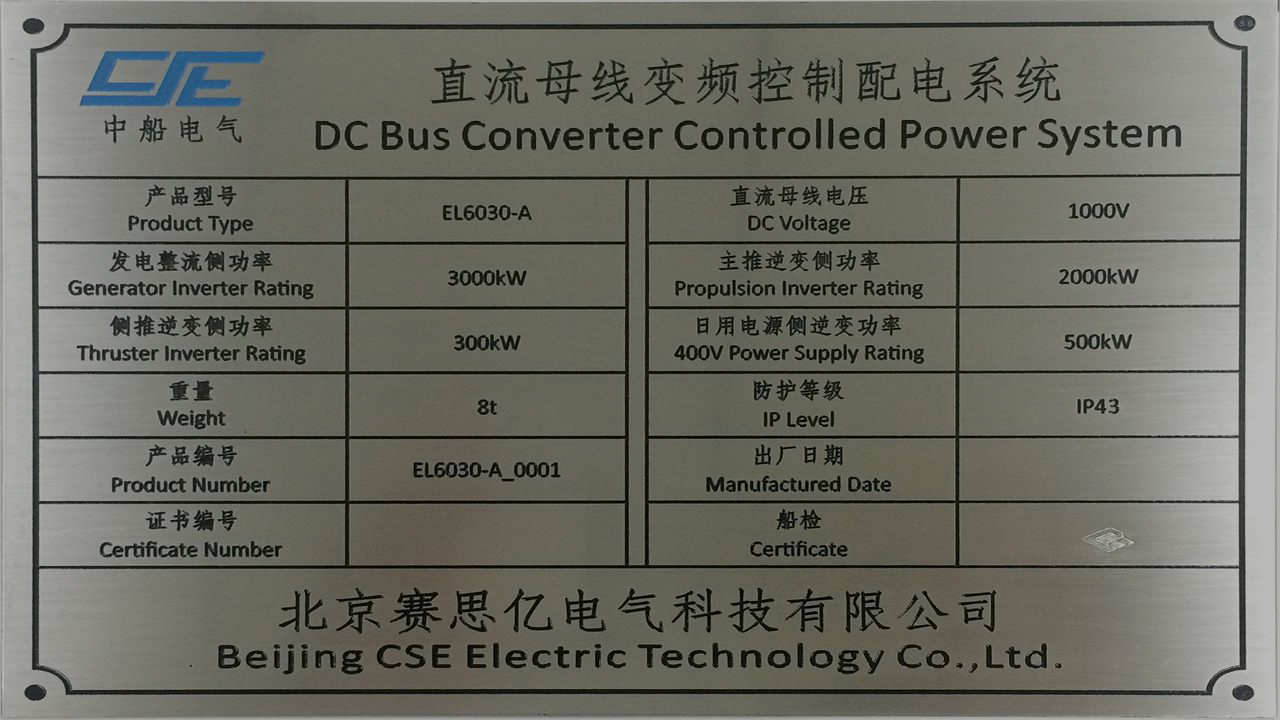

Supplement: S1 Dataset — (ZIP) [file pone.0300792.s001.zip › minimal data set/gt_img_0011_N1.0.jpg]

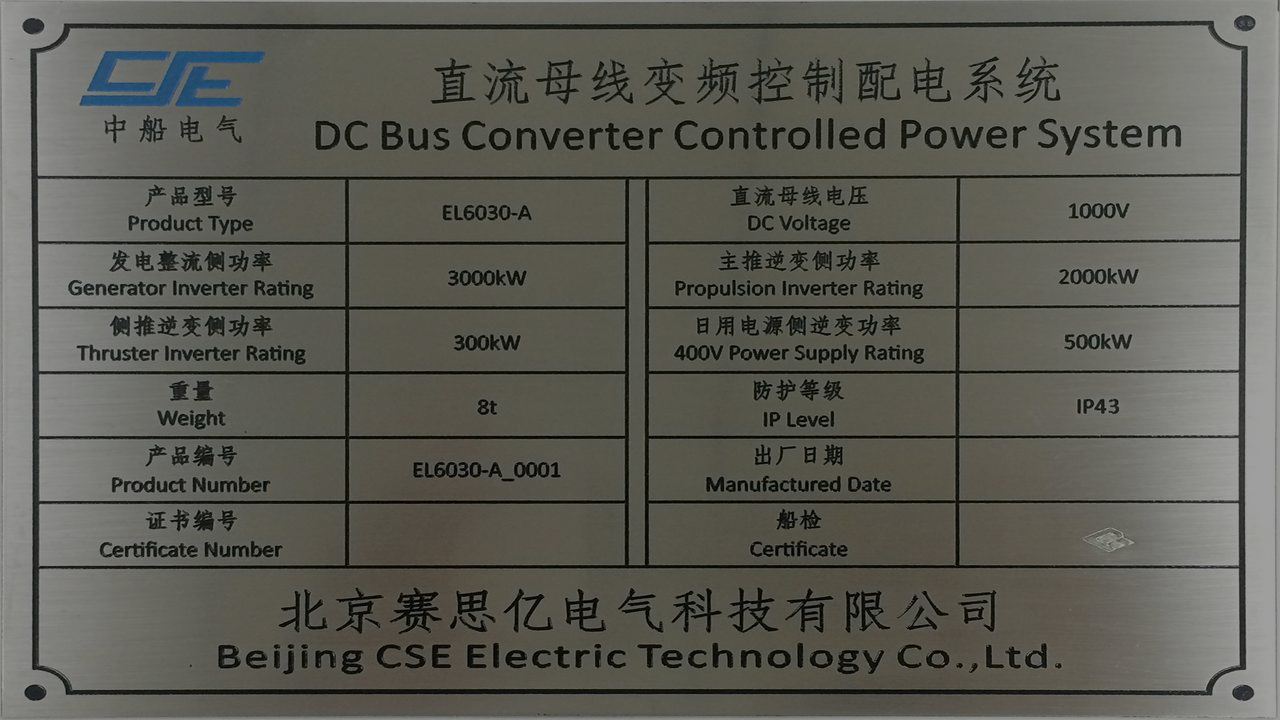

Supplement: S1 Dataset — (ZIP) [file pone.0300792.s001.zip › minimal data set/gt_img_0011_N1.5.jpg]

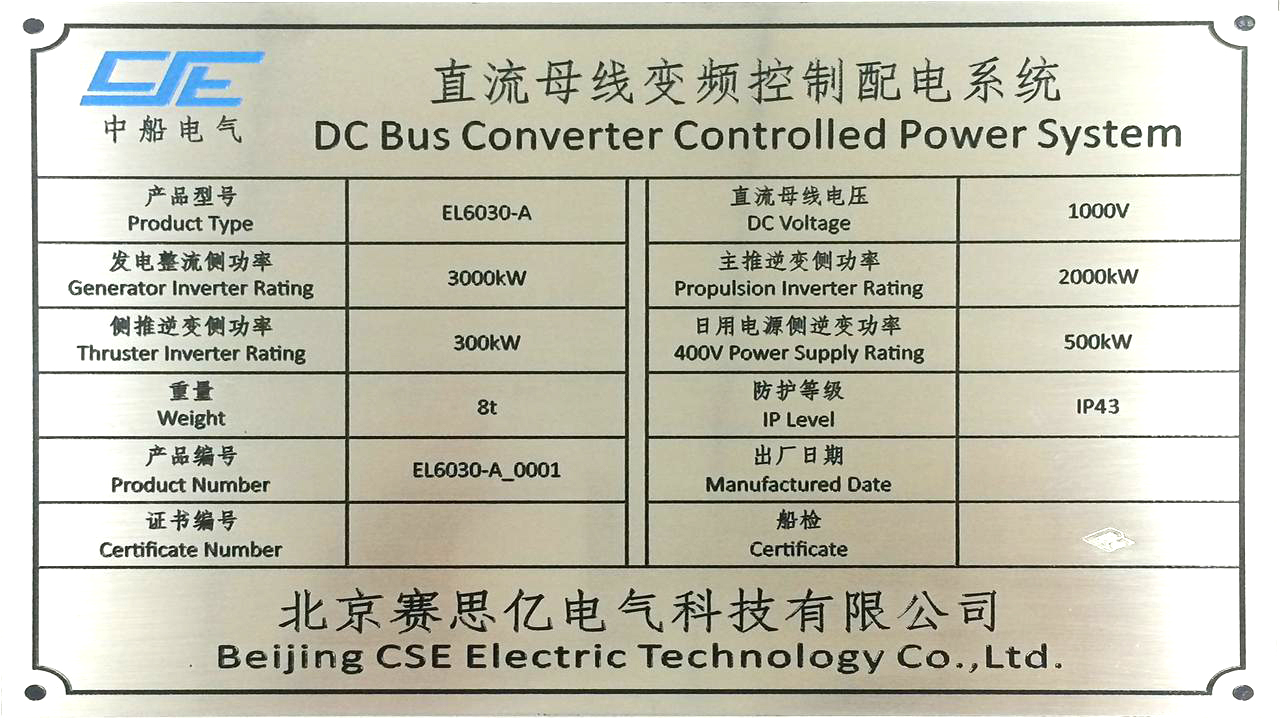

Supplement: S1 Dataset — (ZIP) [file pone.0300792.s001.zip › minimal data set/gt_img_0011_P1.0.jpg]

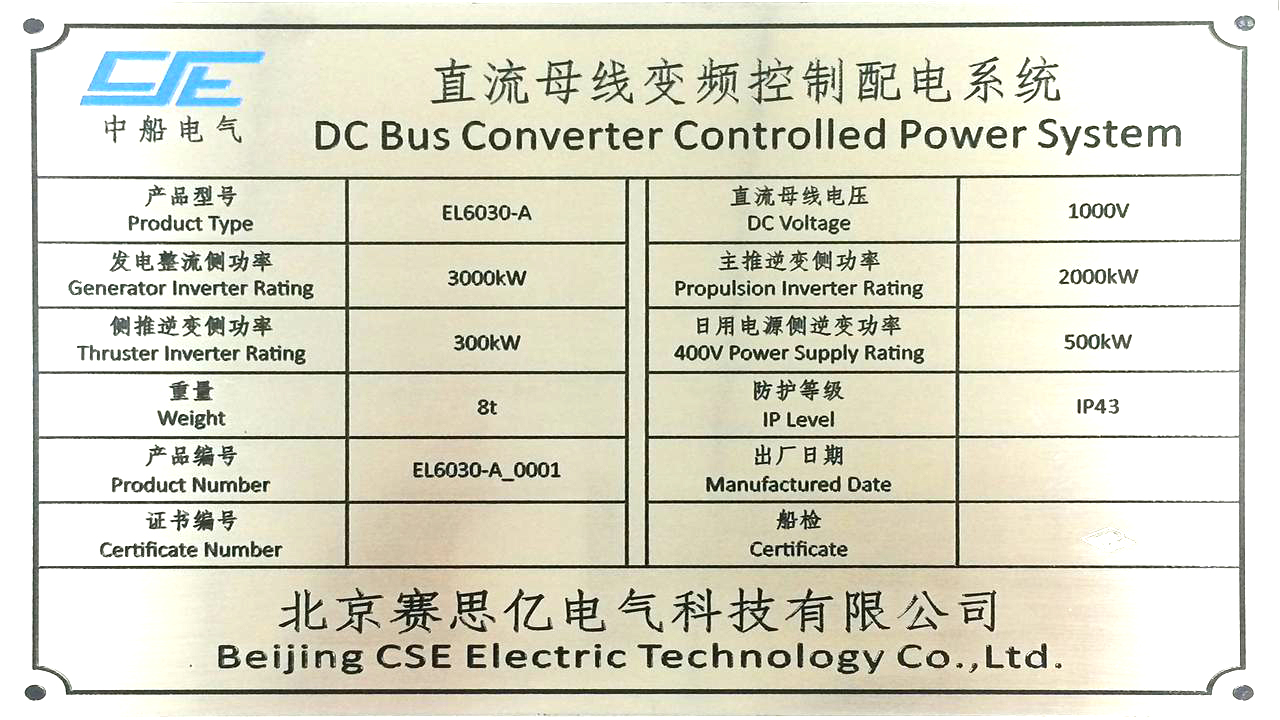

Supplement: S1 Dataset — (ZIP) [file pone.0300792.s001.zip › minimal data set/gt_img_0011_P1.5.jpg]

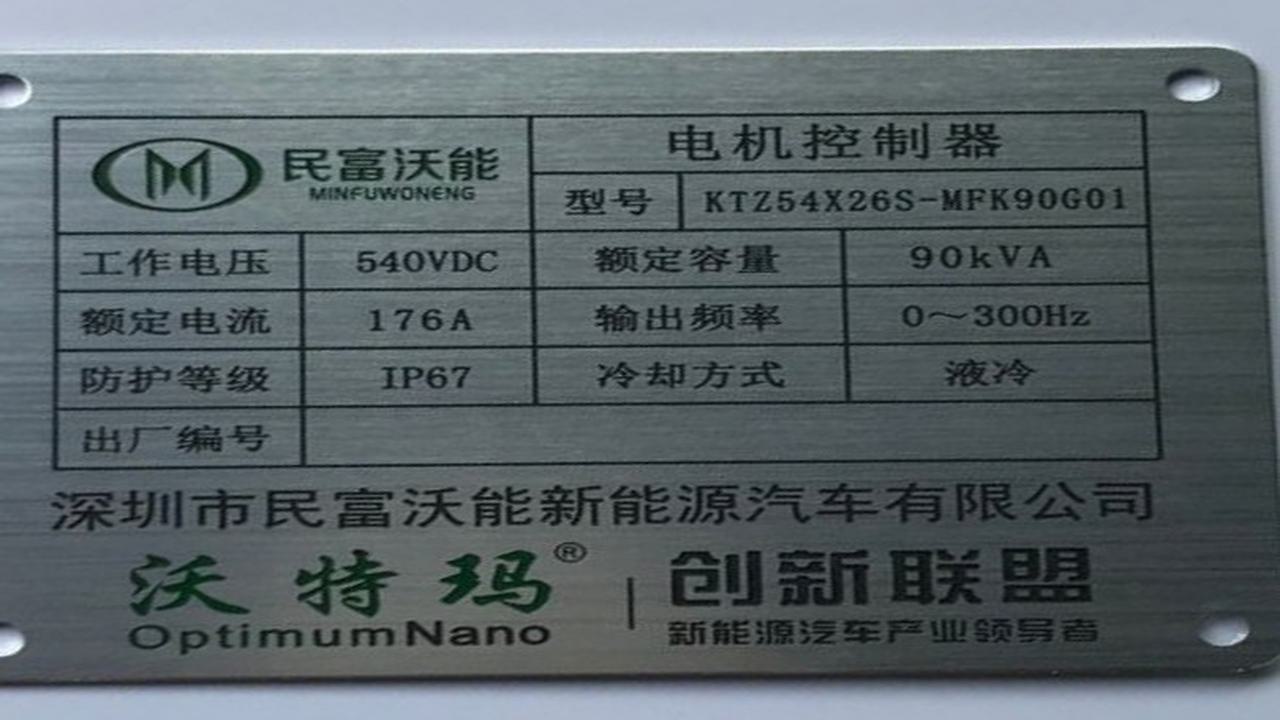

Supplement: S1 Dataset — (ZIP) [file pone.0300792.s001.zip › minimal data set/gt_img_0012_0.jpg]

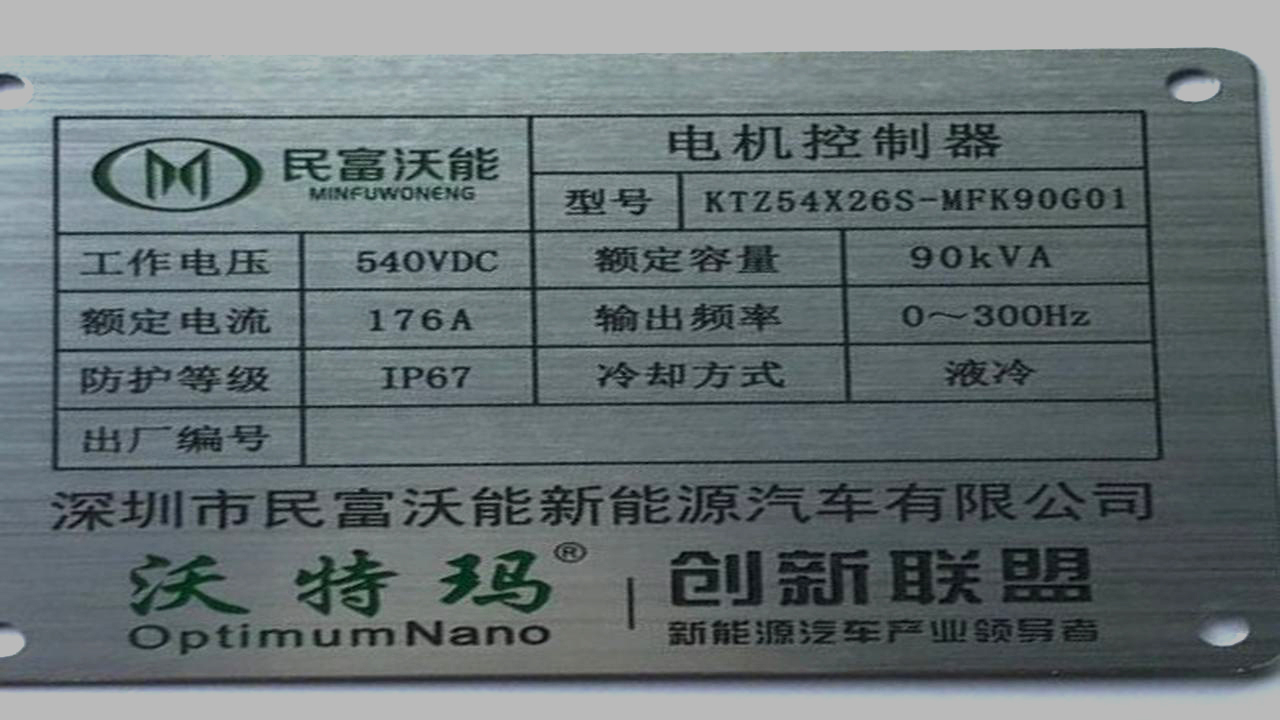

Supplement: S1 Dataset — (ZIP) [file pone.0300792.s001.zip › minimal data set/gt_img_0012_N1.0.jpg]

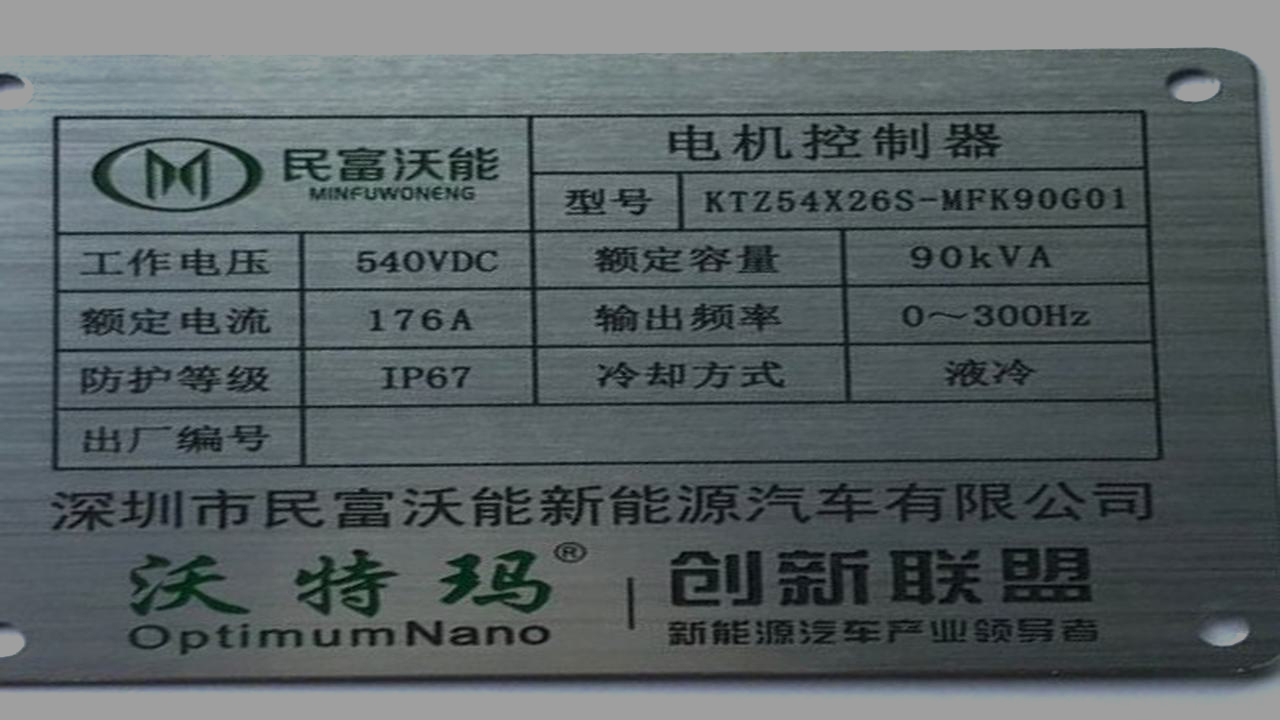

Supplement: S1 Dataset — (ZIP) [file pone.0300792.s001.zip › minimal data set/gt_img_0012_N1.5.jpg]

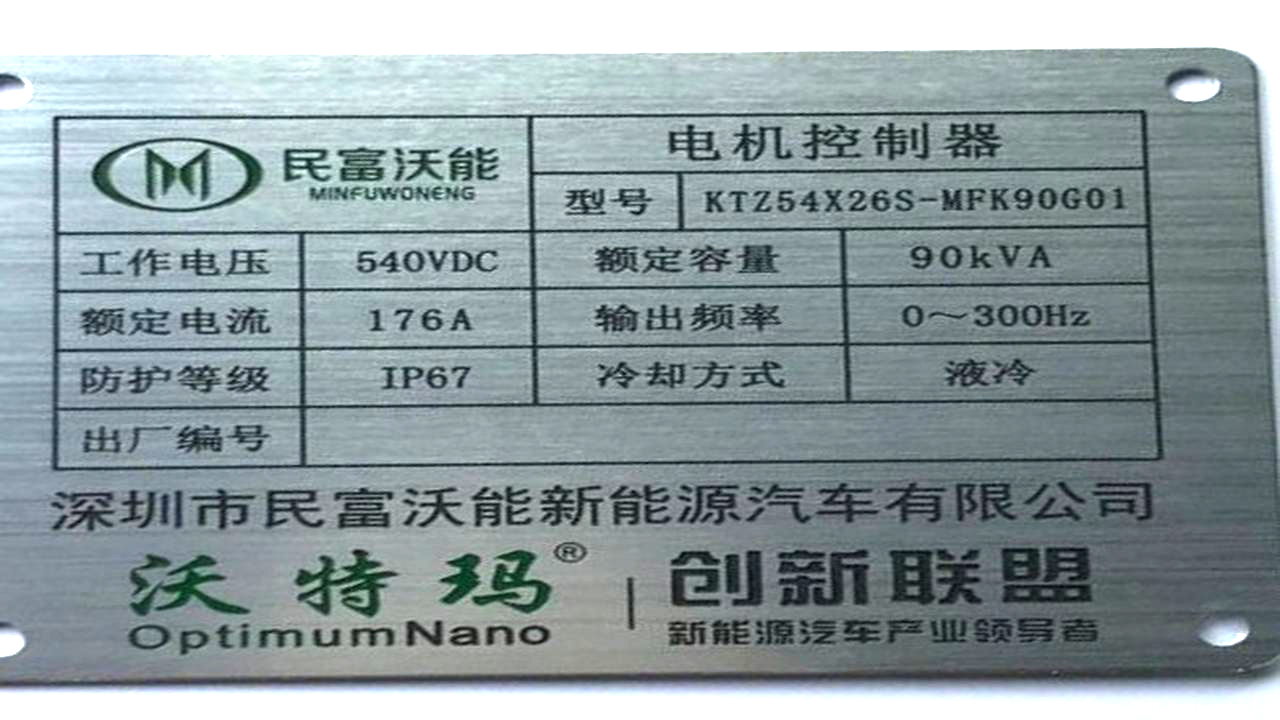

Supplement: S1 Dataset — (ZIP) [file pone.0300792.s001.zip › minimal data set/gt_img_0012_P1.0.jpg]

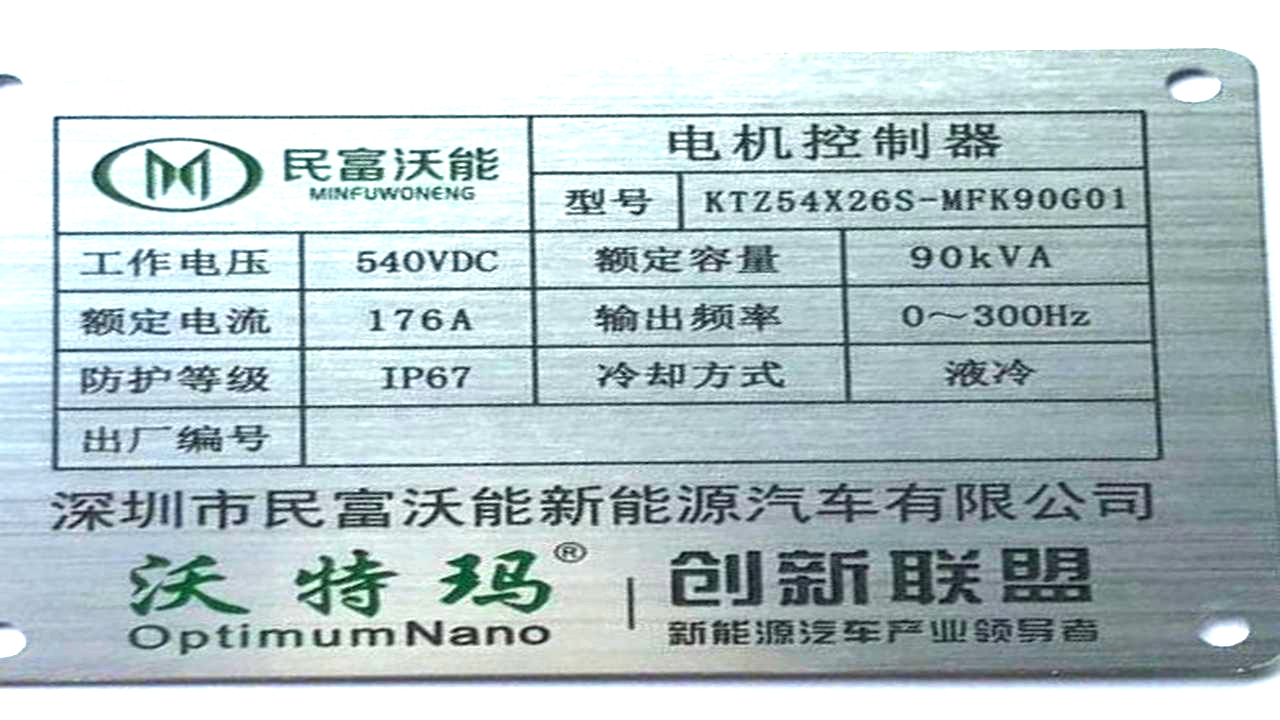

Supplement: S1 Dataset — (ZIP) [file pone.0300792.s001.zip › minimal data set/gt_img_0012_P1.5.jpg]

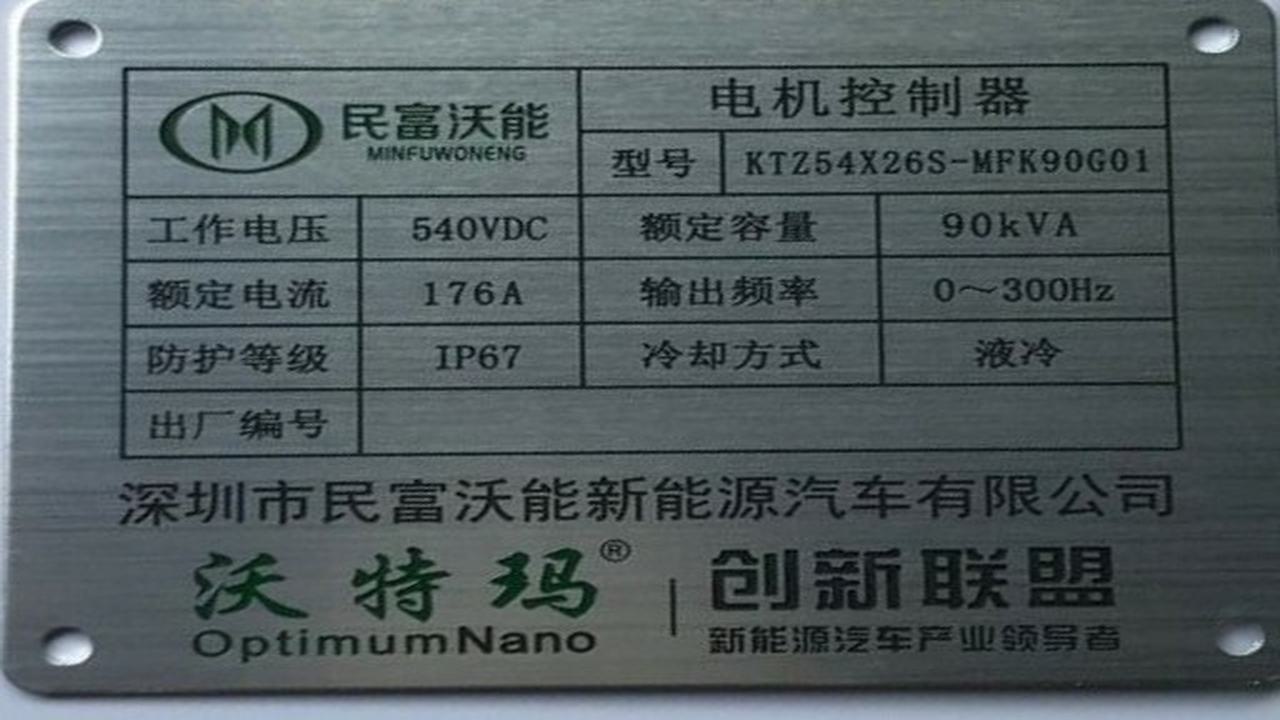

Supplement: S1 Dataset — (ZIP) [file pone.0300792.s001.zip › minimal data set/gt_img_0013_0.jpg]

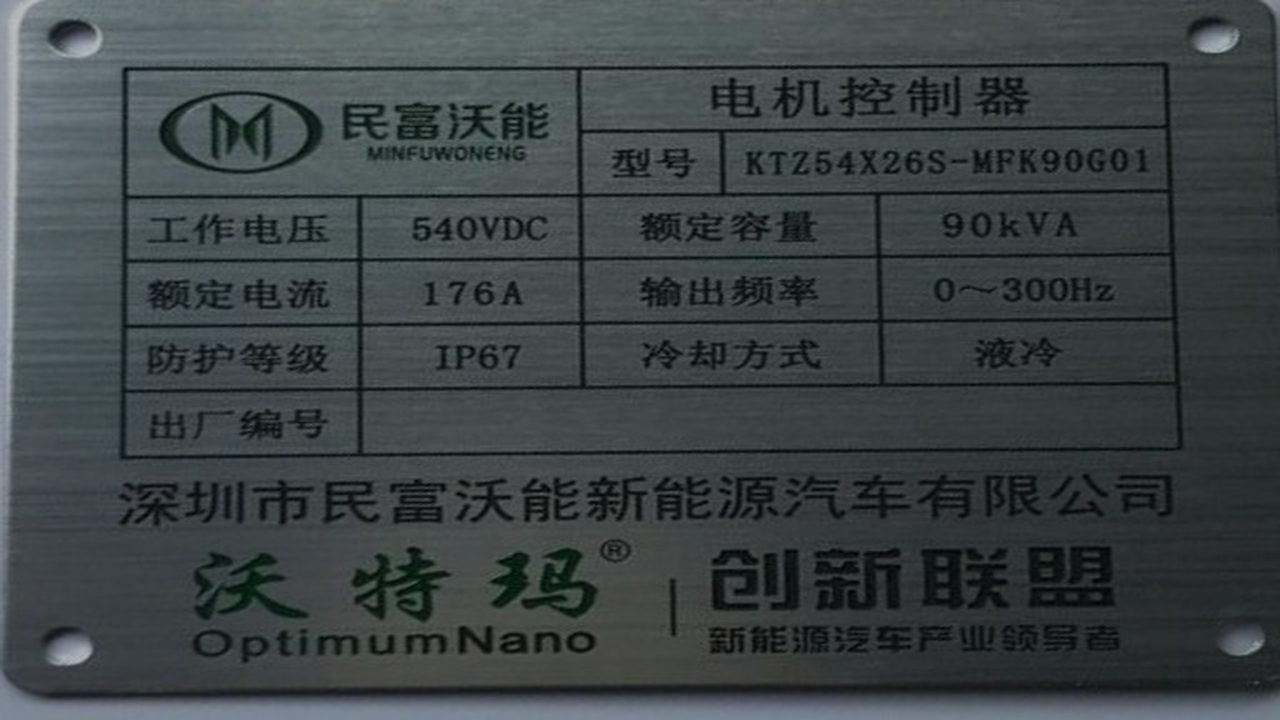

Supplement: S1 Dataset — (ZIP) [file pone.0300792.s001.zip › minimal data set/gt_img_0013_N1.0.jpg]

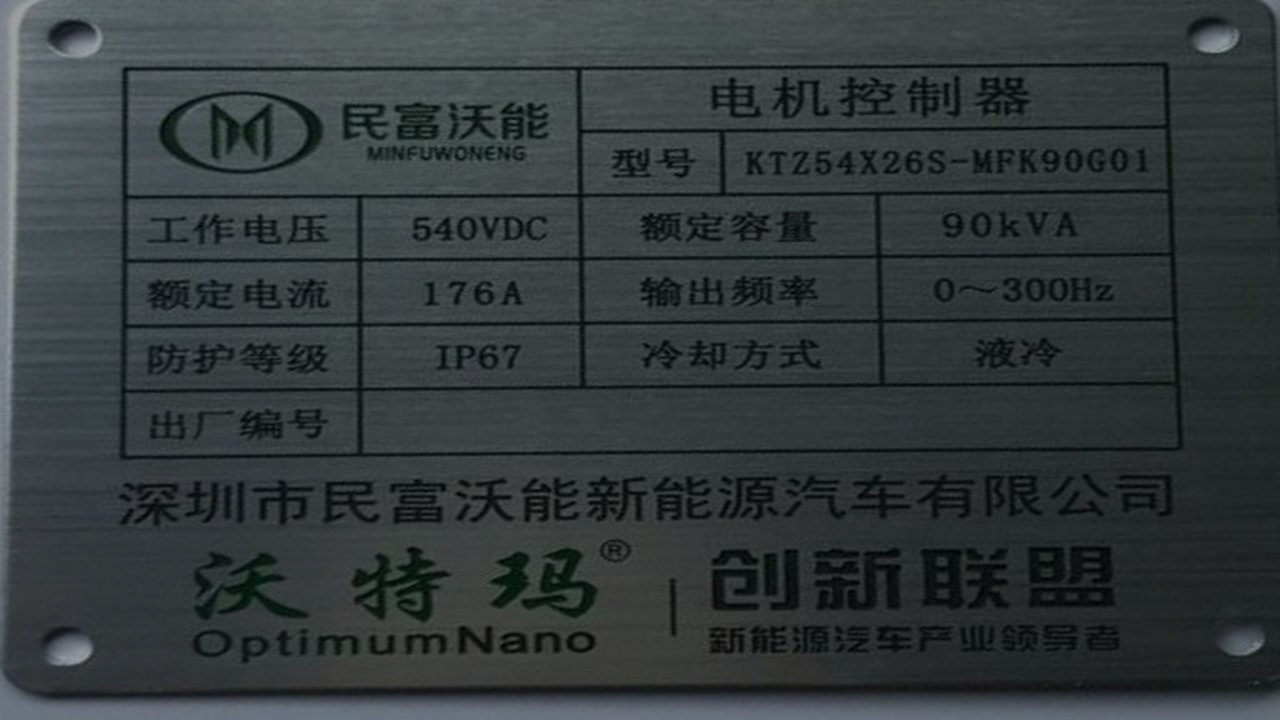

Supplement: S1 Dataset — (ZIP) [file pone.0300792.s001.zip › minimal data set/gt_img_0013_N1.5.jpg]

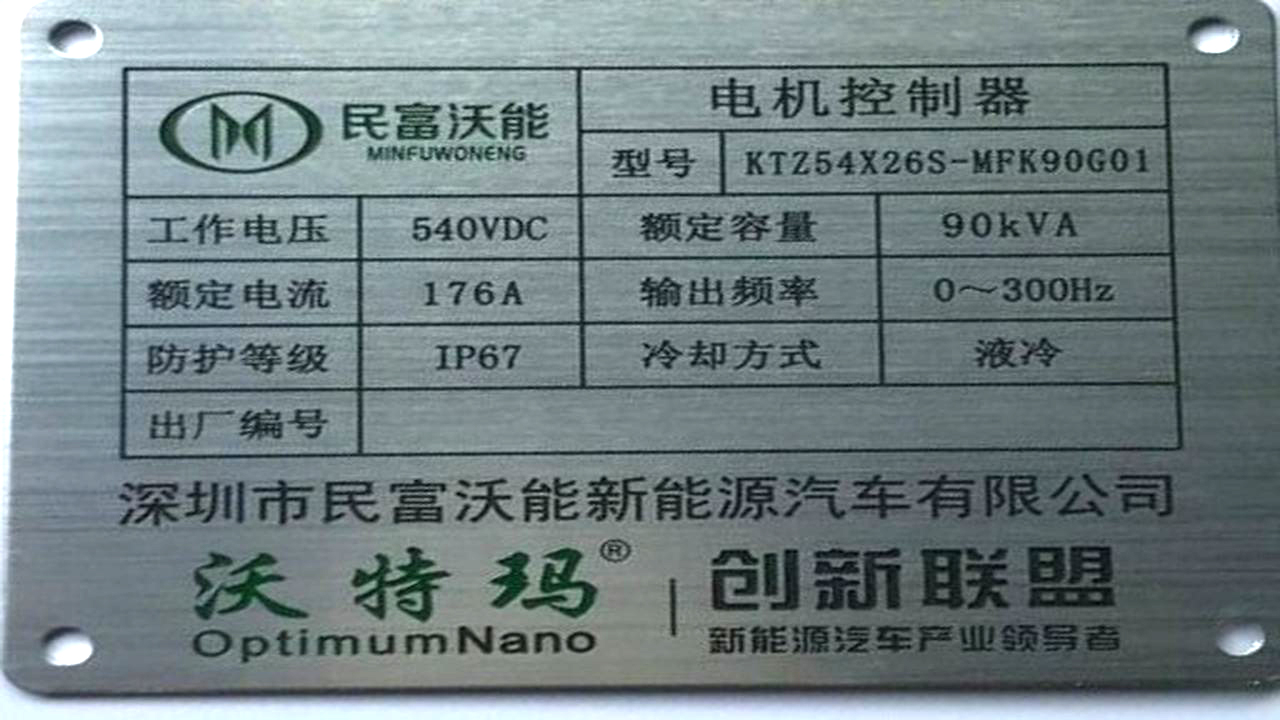

Supplement: S1 Dataset — (ZIP) [file pone.0300792.s001.zip › minimal data set/gt_img_0013_P1.0.jpg]

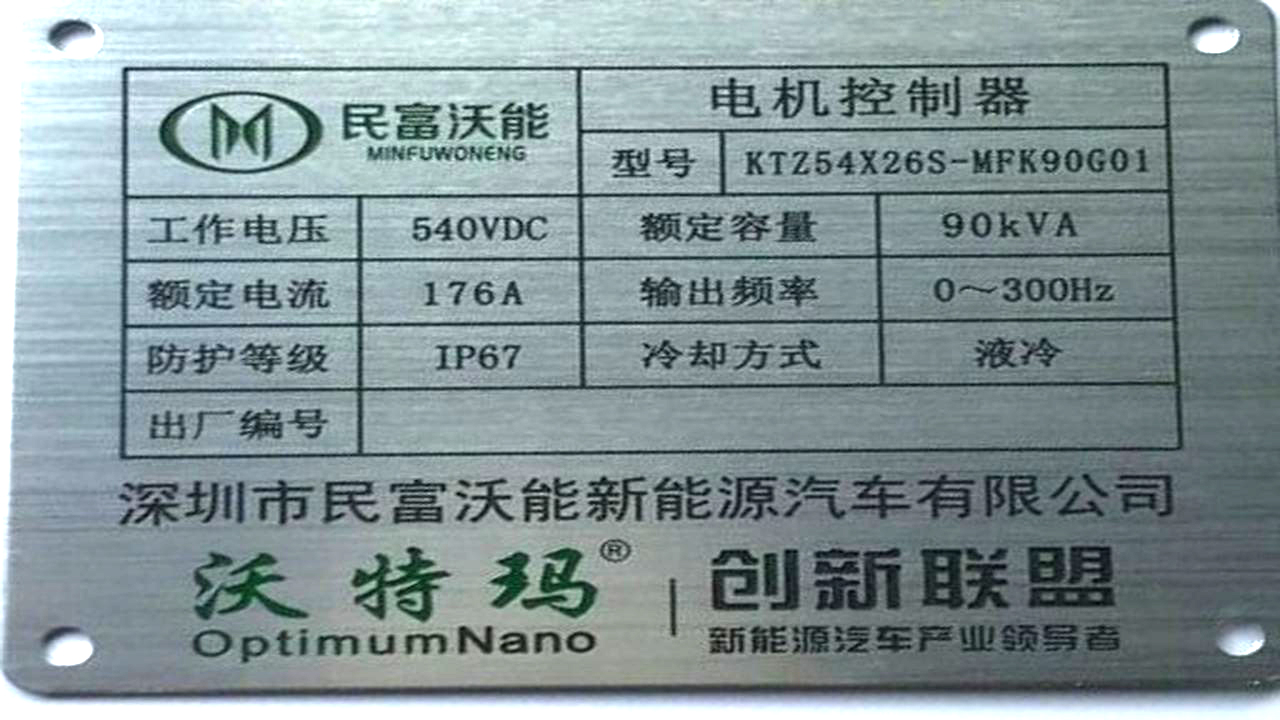

Supplement: S1 Dataset — (ZIP) [file pone.0300792.s001.zip › minimal data set/gt_img_0013_P1.5.jpg]

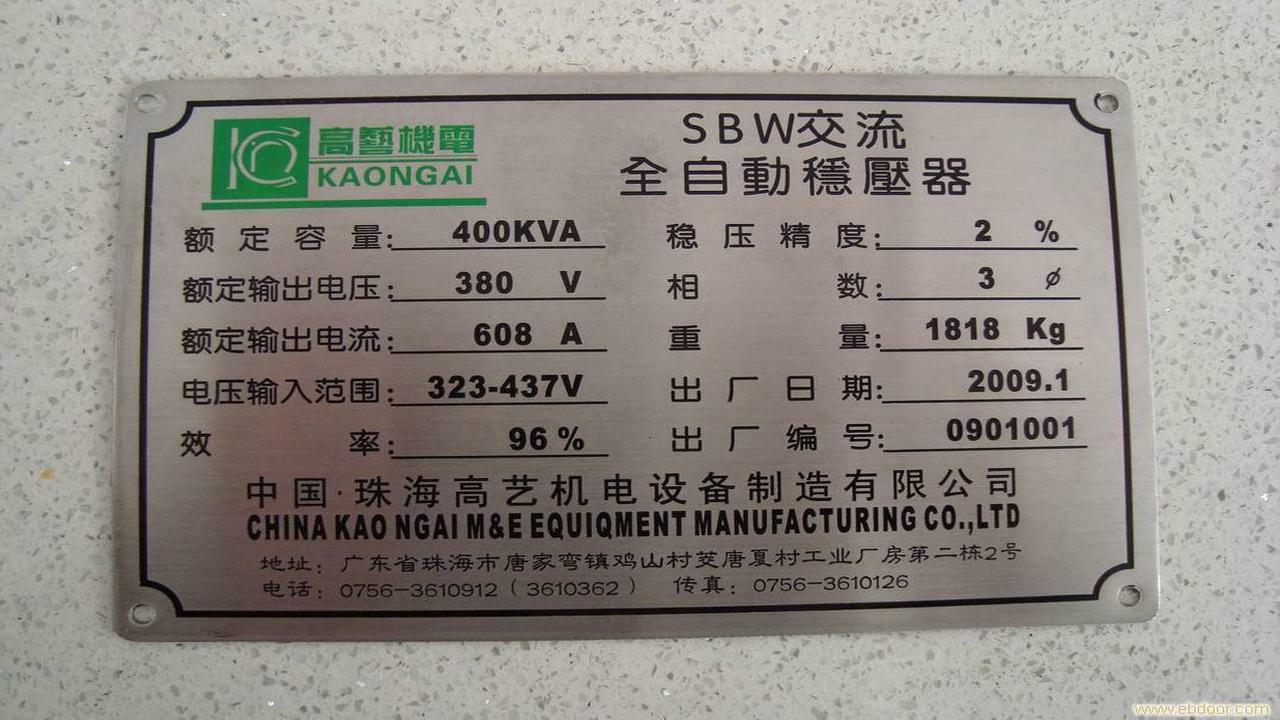

Supplement: S1 Dataset — (ZIP) [file pone.0300792.s001.zip › minimal data set/gt_img_0014_0.jpg]

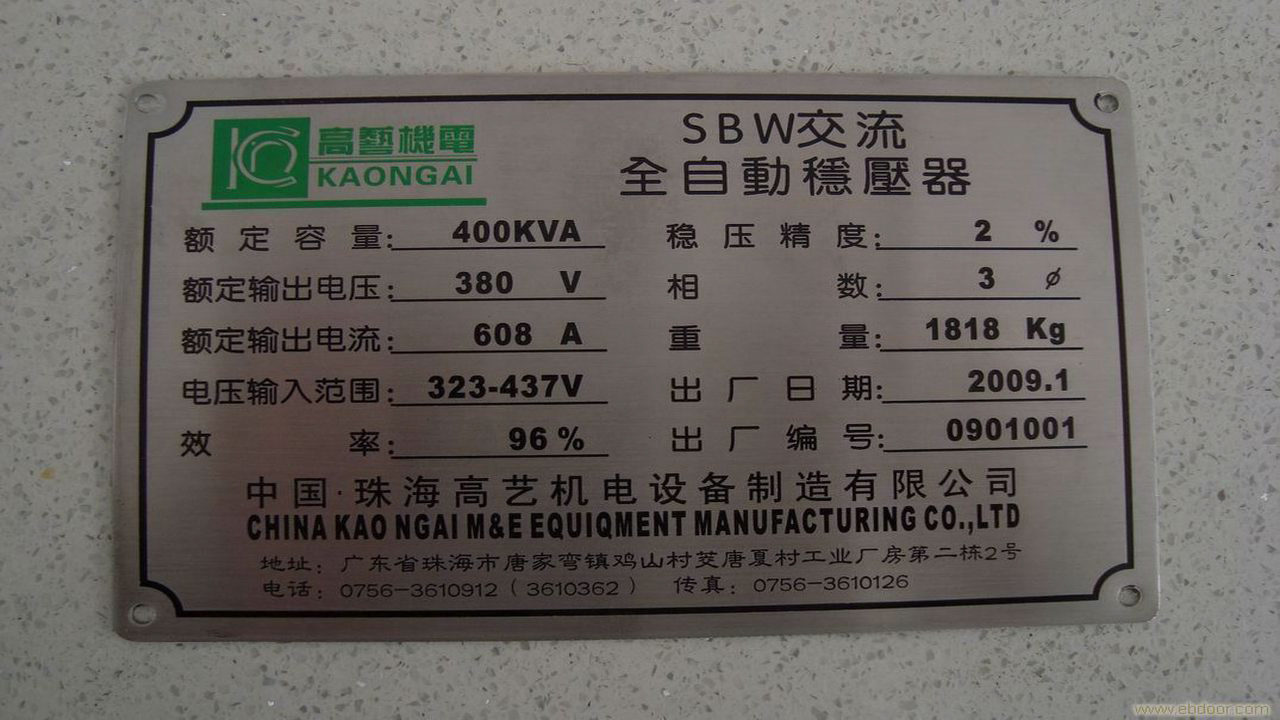

Supplement: S1 Dataset — (ZIP) [file pone.0300792.s001.zip › minimal data set/gt_img_0014_N1.0.jpg]

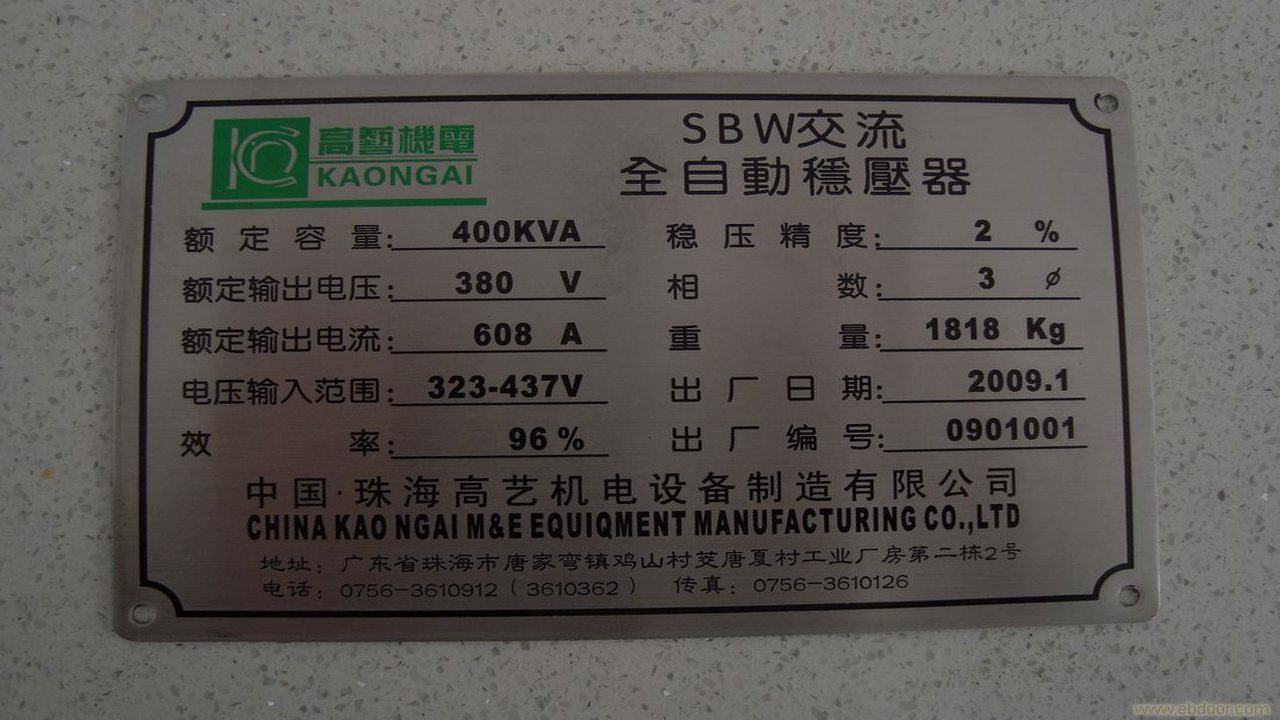

Supplement: S1 Dataset — (ZIP) [file pone.0300792.s001.zip › minimal data set/gt_img_0014_N1.5.jpg]

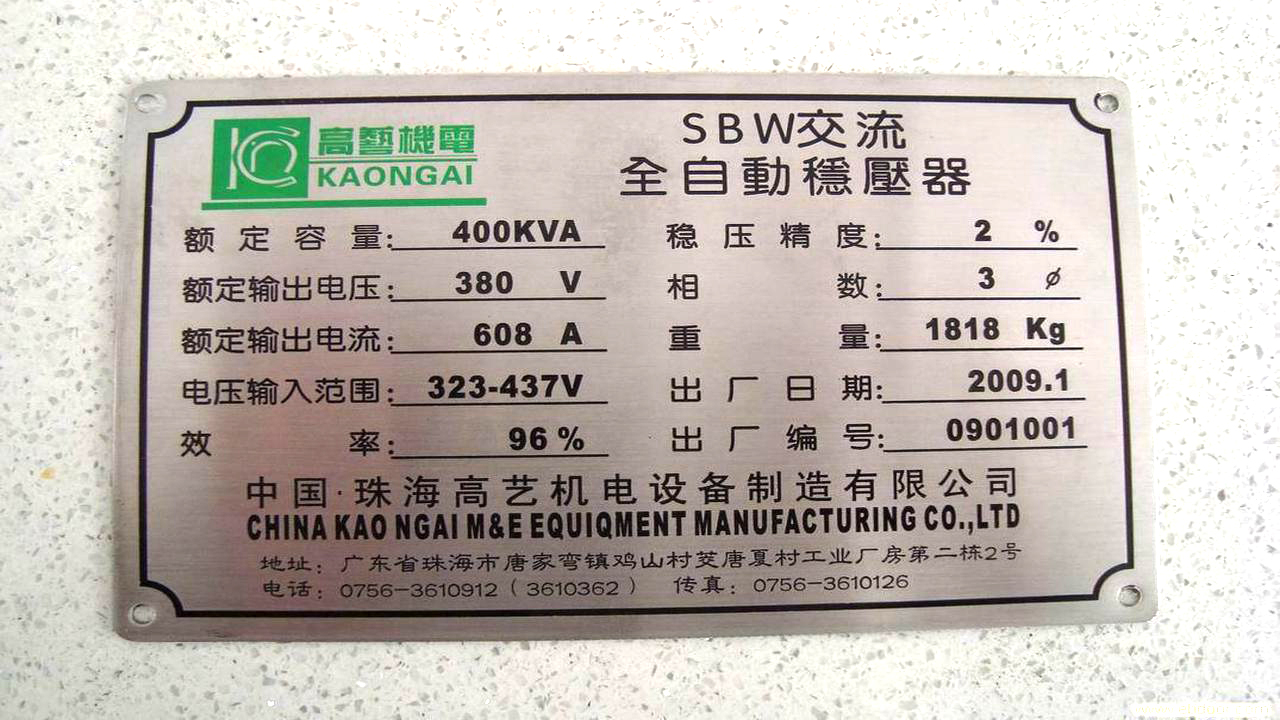

Supplement: S1 Dataset — (ZIP) [file pone.0300792.s001.zip › minimal data set/gt_img_0014_P1.0.jpg]

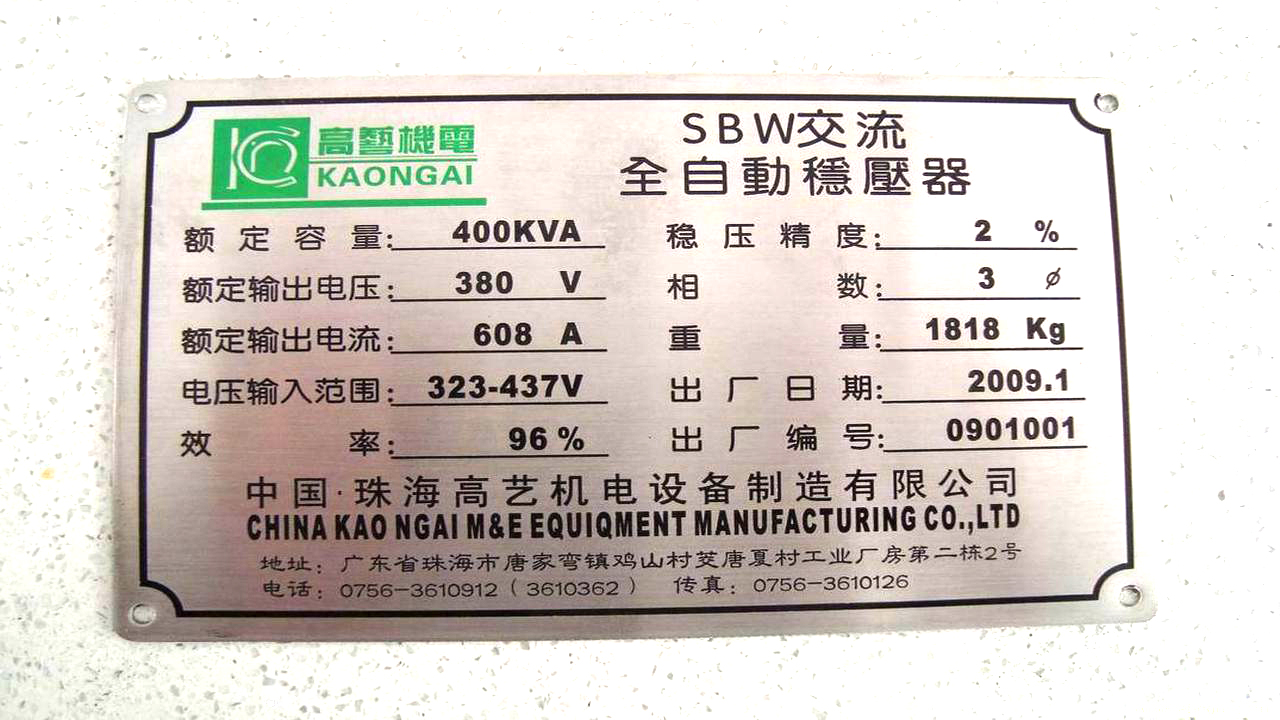

Supplement: S1 Dataset — (ZIP) [file pone.0300792.s001.zip › minimal data set/gt_img_0014_P1.5.jpg]

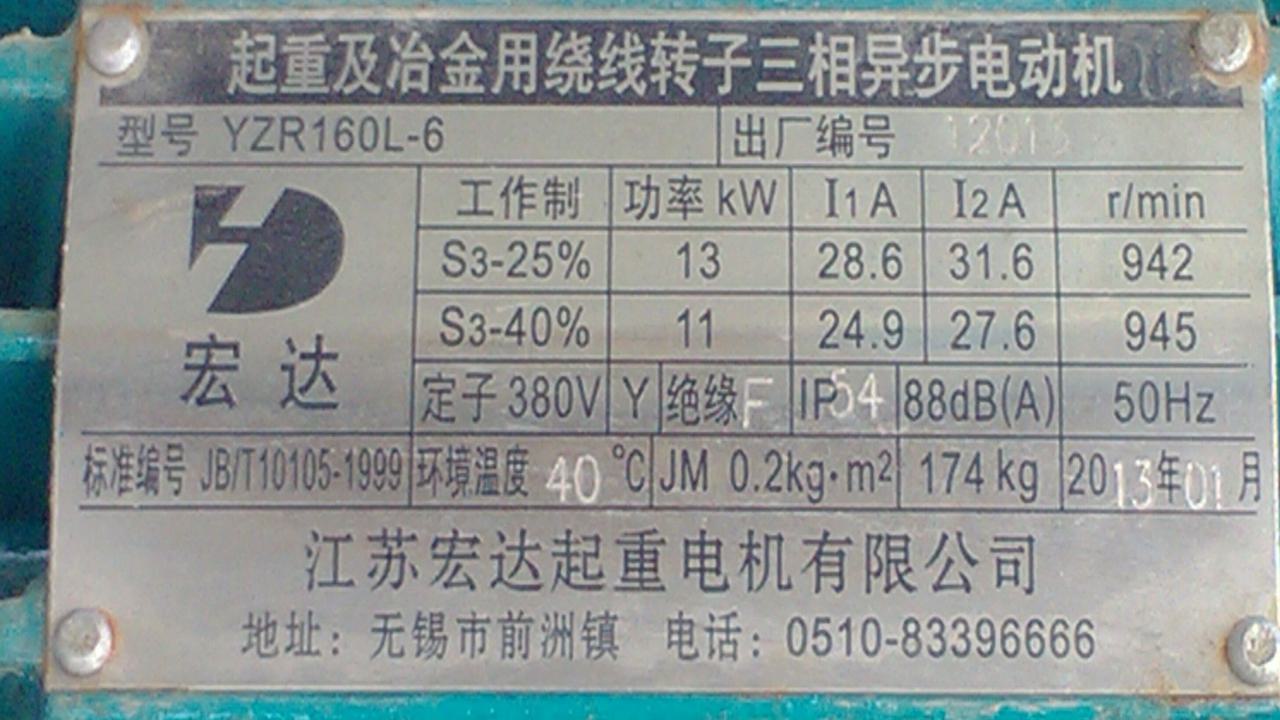

Supplement: S1 Dataset — (ZIP) [file pone.0300792.s001.zip › minimal data set/gt_img_0015_0.jpg]

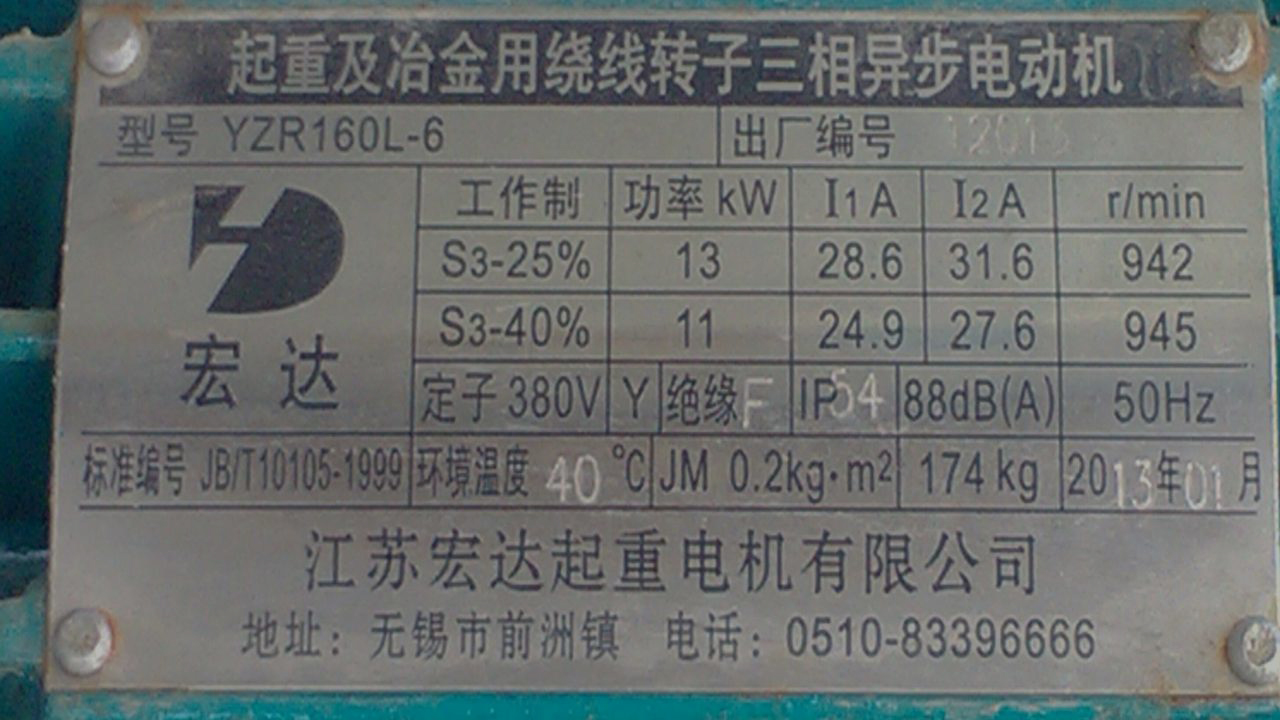

Supplement: S1 Dataset — (ZIP) [file pone.0300792.s001.zip › minimal data set/gt_img_0015_N1.0.jpg]

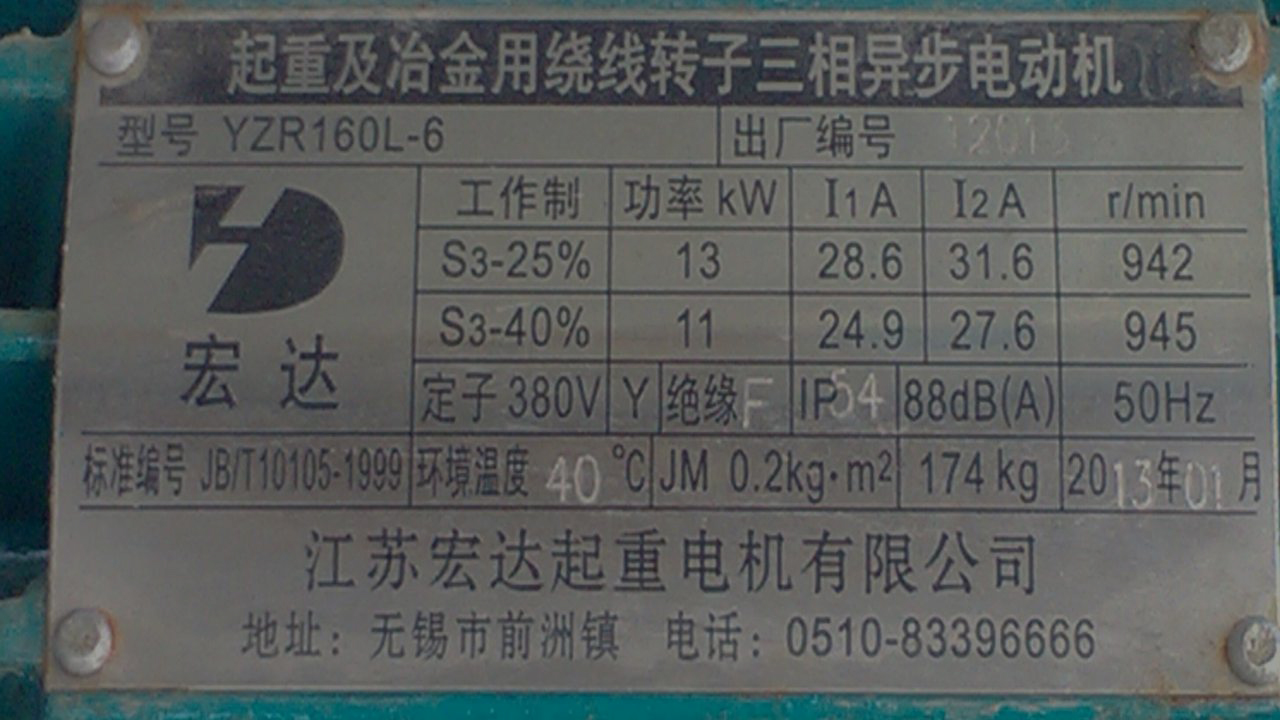

Supplement: S1 Dataset — (ZIP) [file pone.0300792.s001.zip › minimal data set/gt_img_0015_N1.5.jpg]

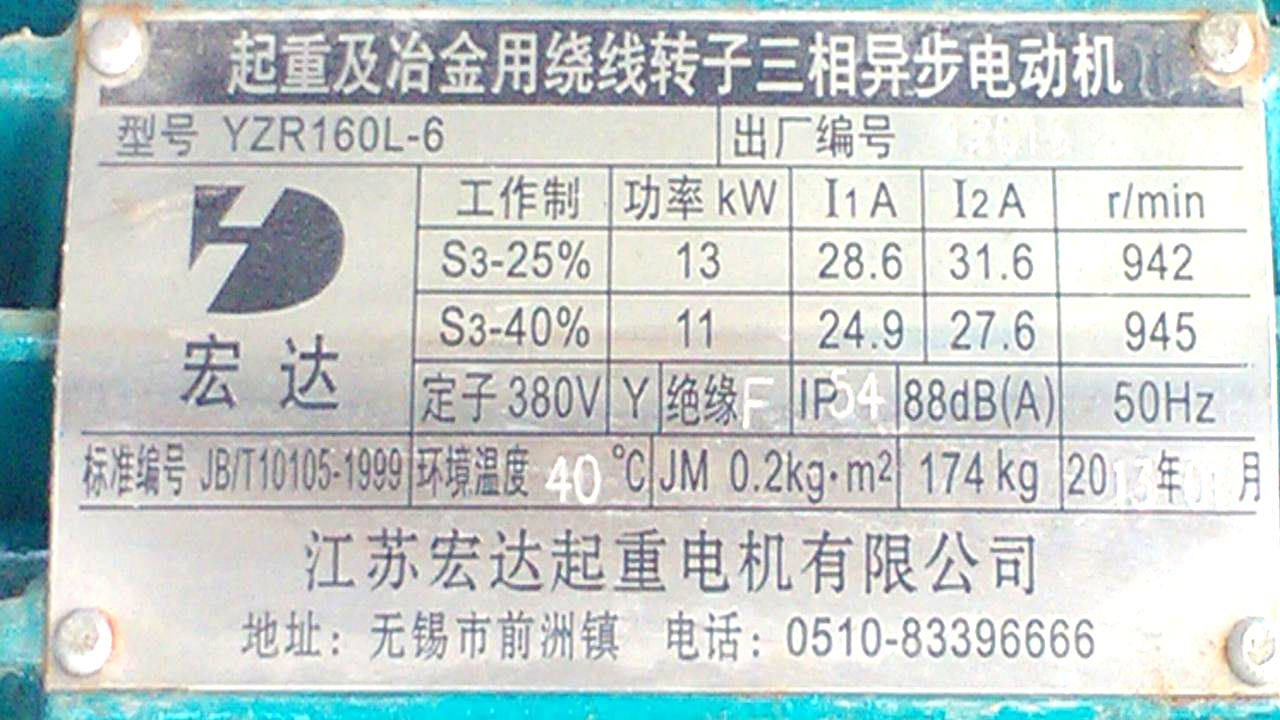

Supplement: S1 Dataset — (ZIP) [file pone.0300792.s001.zip › minimal data set/gt_img_0015_P1.0.jpg]

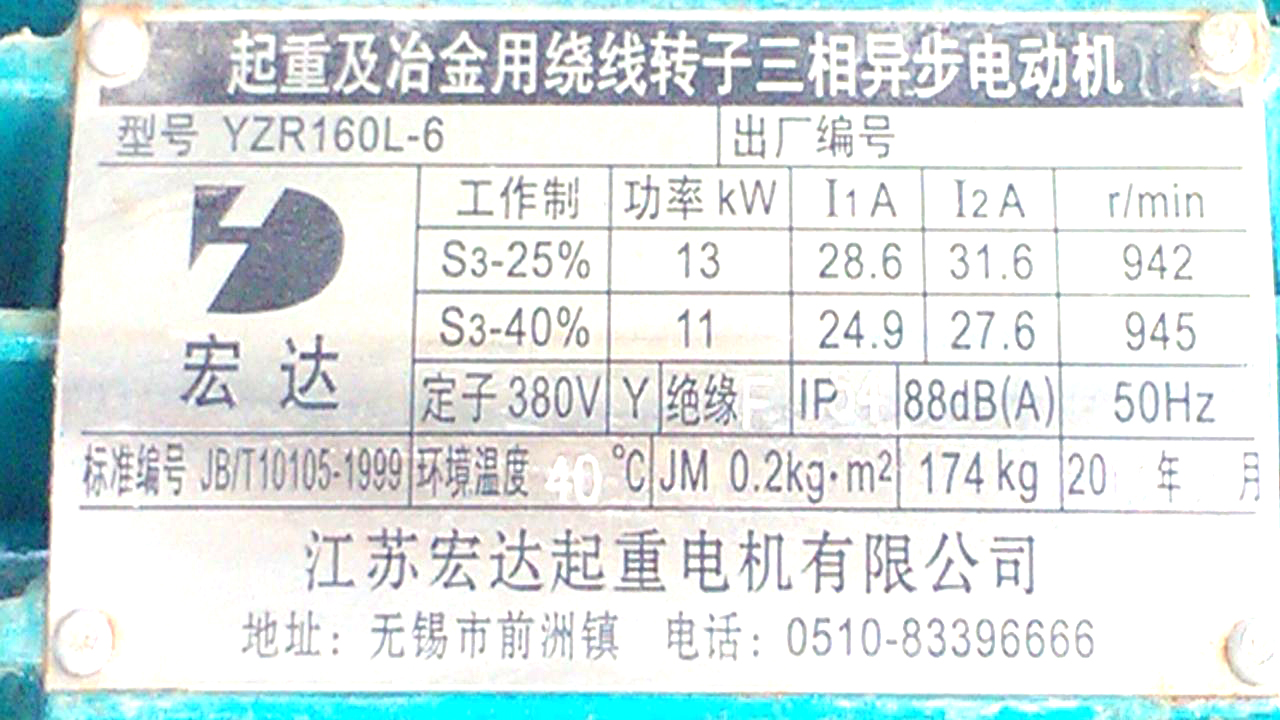

Supplement: S1 Dataset — (ZIP) [file pone.0300792.s001.zip › minimal data set/gt_img_0015_P1.5.jpg]

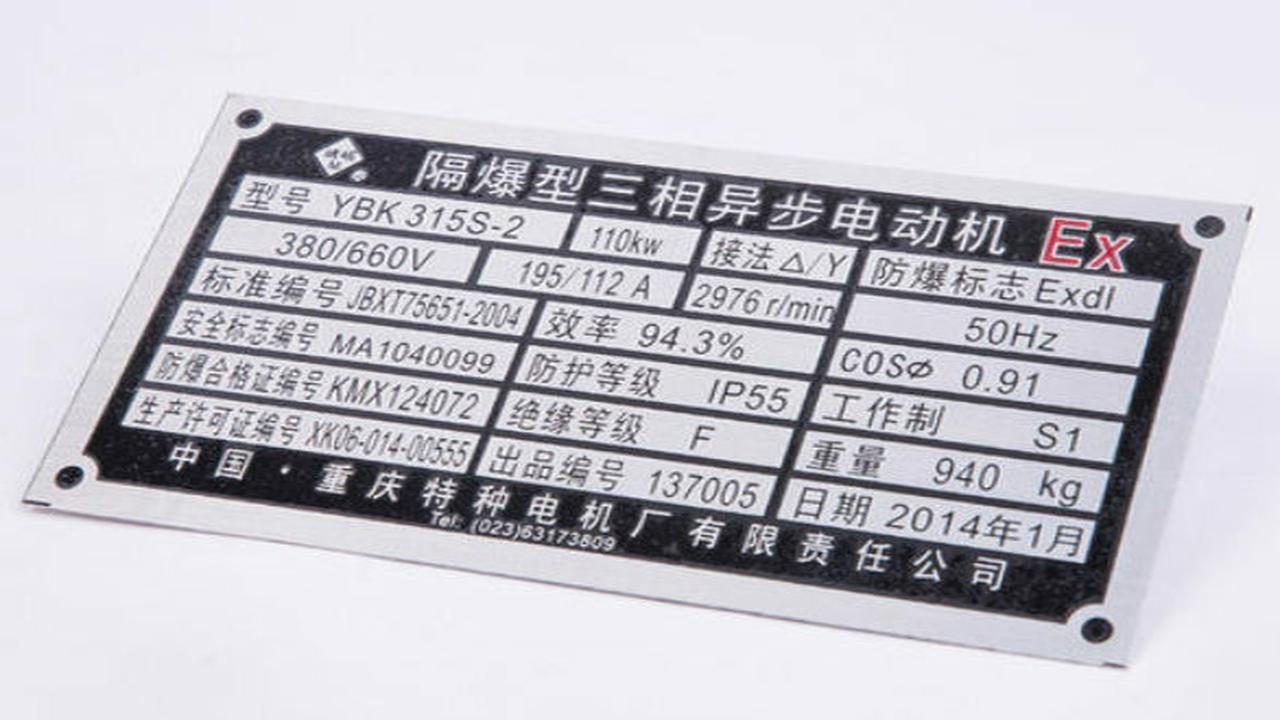

Supplement: S1 Dataset — (ZIP) [file pone.0300792.s001.zip › minimal data set/gt_img_0016_0.jpg]

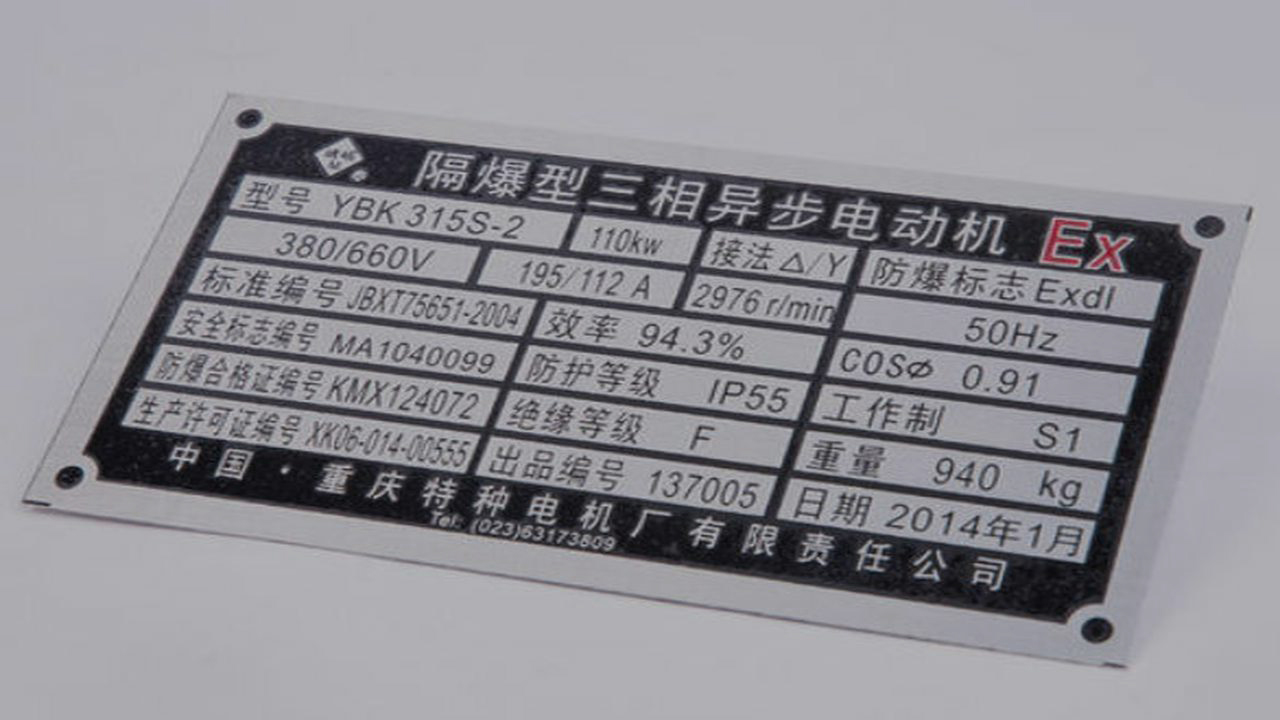

Supplement: S1 Dataset — (ZIP) [file pone.0300792.s001.zip › minimal data set/gt_img_0016_N1.0.jpg]

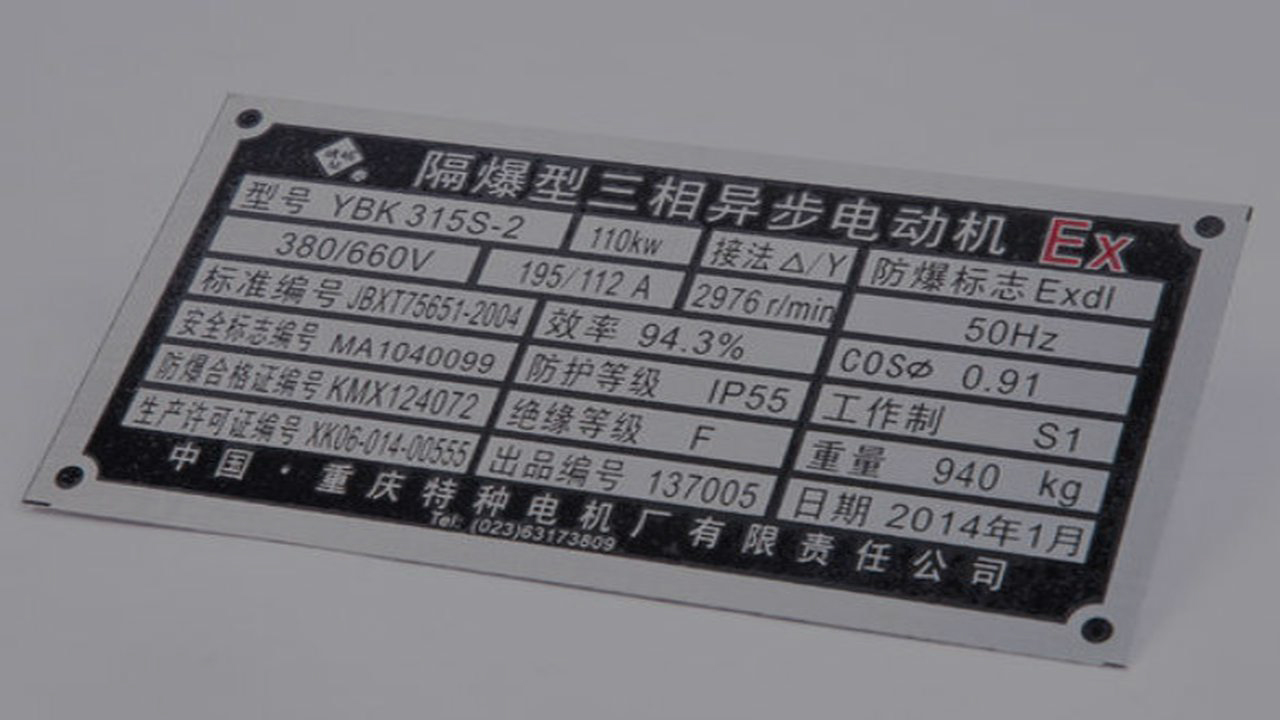

Supplement: S1 Dataset — (ZIP) [file pone.0300792.s001.zip › minimal data set/gt_img_0016_N1.5.jpg]

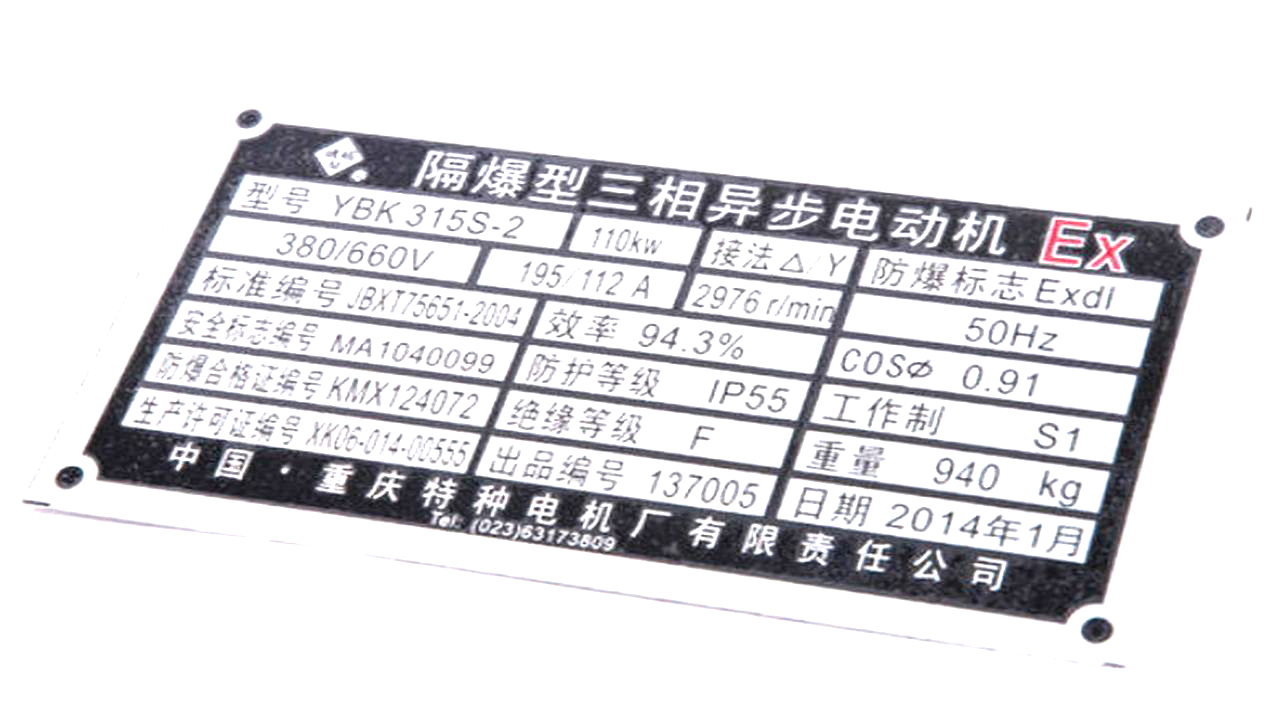

Supplement: S1 Dataset — (ZIP) [file pone.0300792.s001.zip › minimal data set/gt_img_0016_P1.0.jpg]

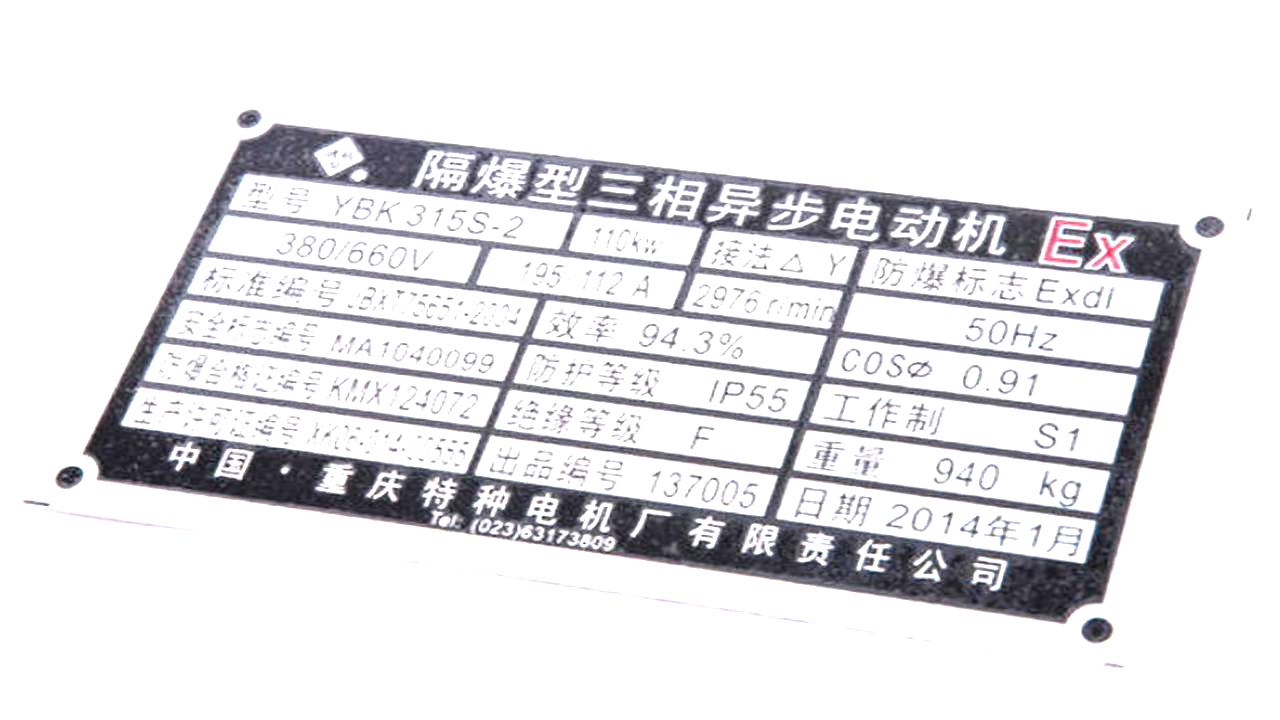

Supplement: S1 Dataset — (ZIP) [file pone.0300792.s001.zip › minimal data set/gt_img_0016_P1.5.jpg]

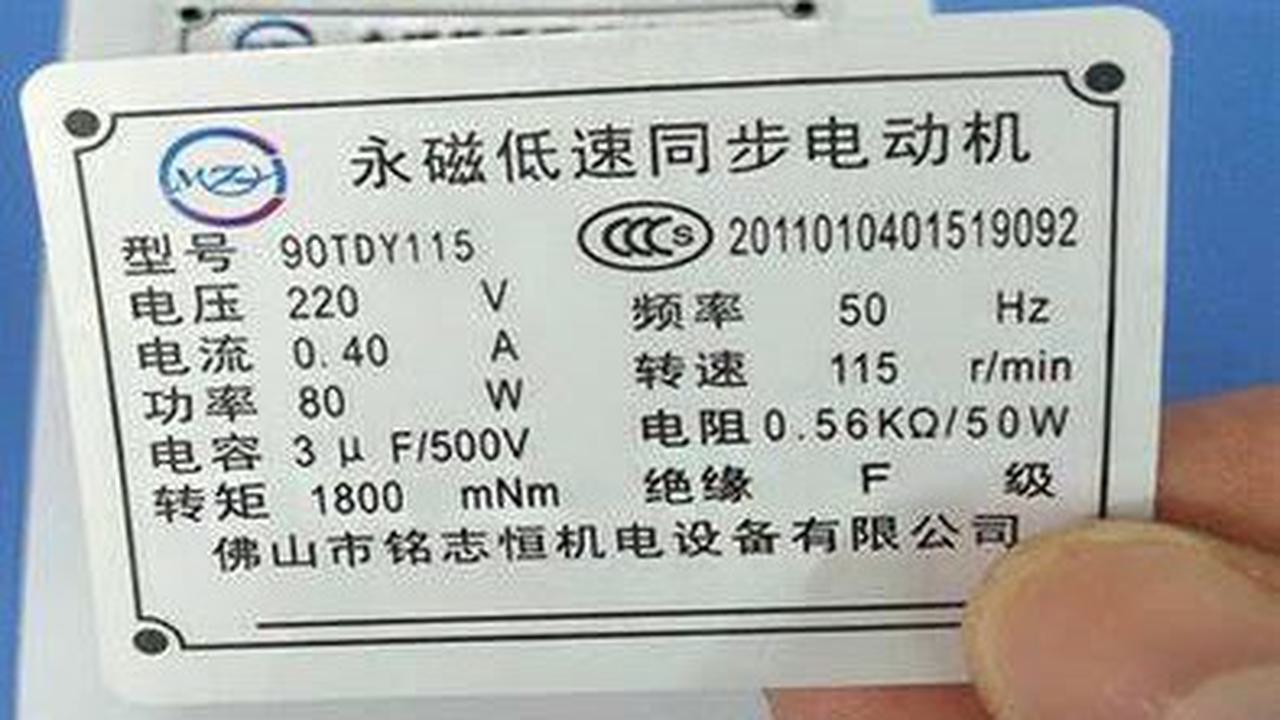

Supplement: S1 Dataset — (ZIP) [file pone.0300792.s001.zip › minimal data set/gt_img_0017_0.jpg]

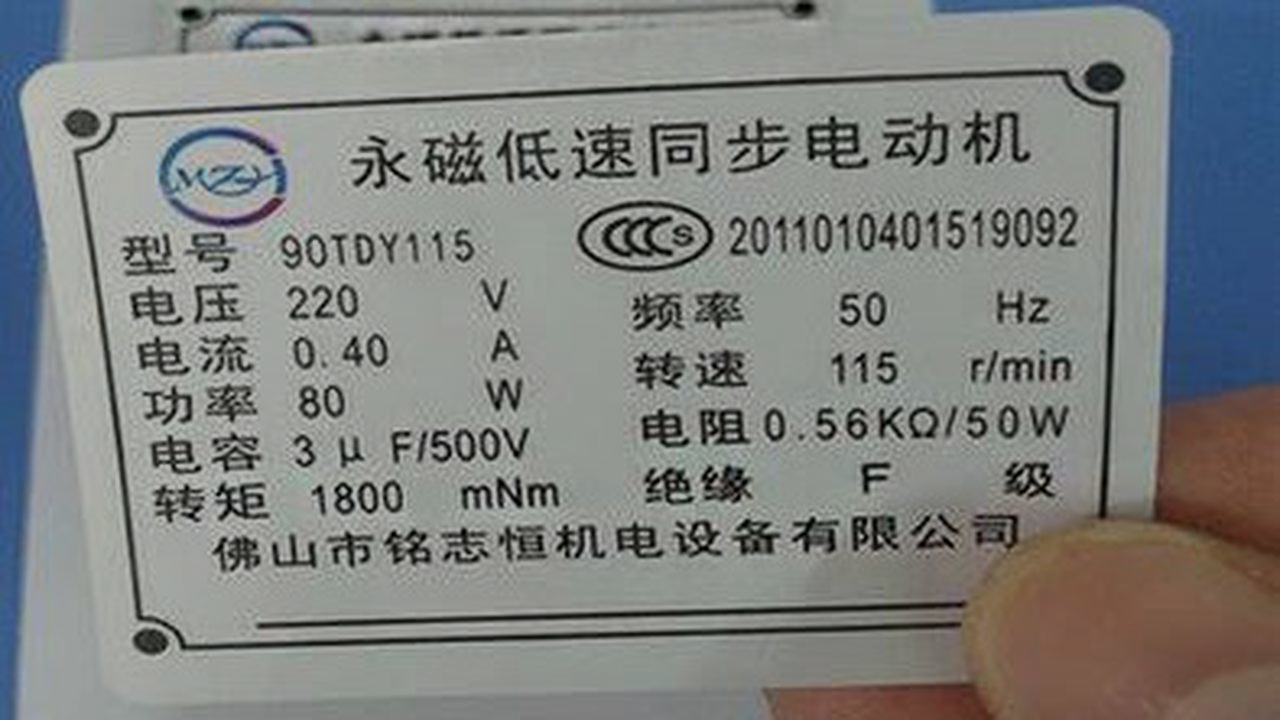

Supplement: S1 Dataset — (ZIP) [file pone.0300792.s001.zip › minimal data set/gt_img_0017_N1.0.jpg]

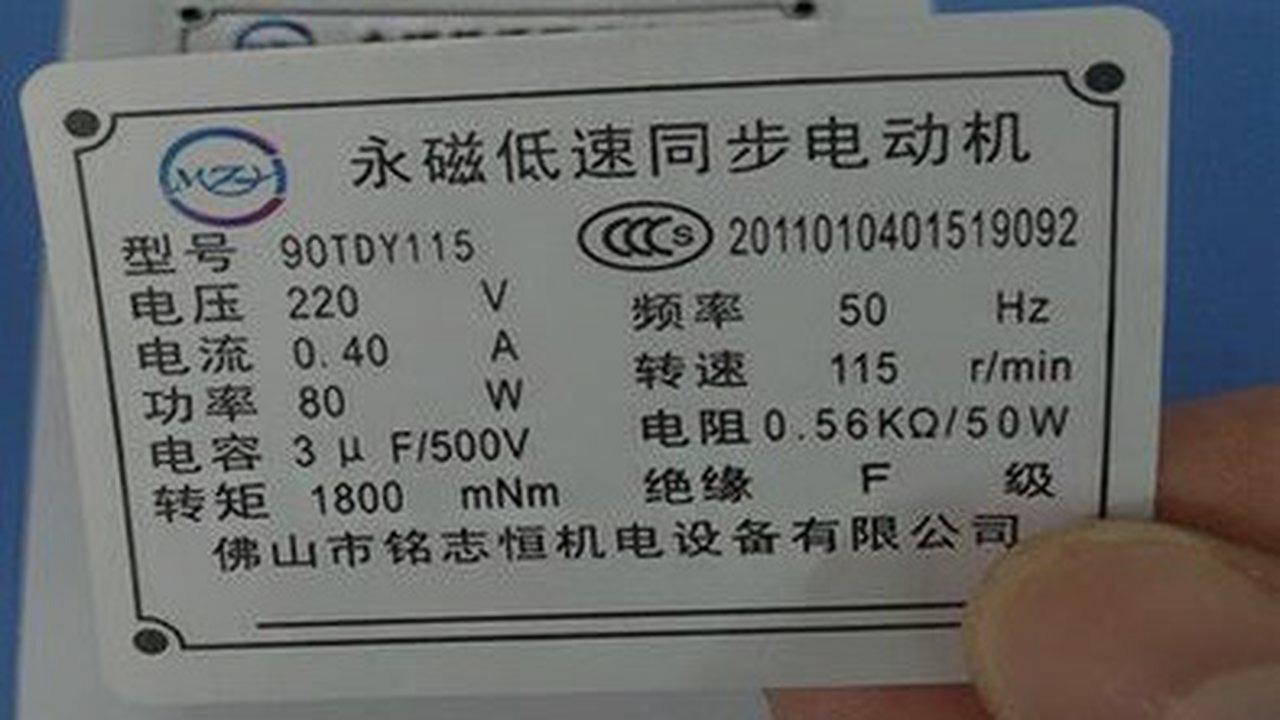

Supplement: S1 Dataset — (ZIP) [file pone.0300792.s001.zip › minimal data set/gt_img_0017_N1.5.jpg]

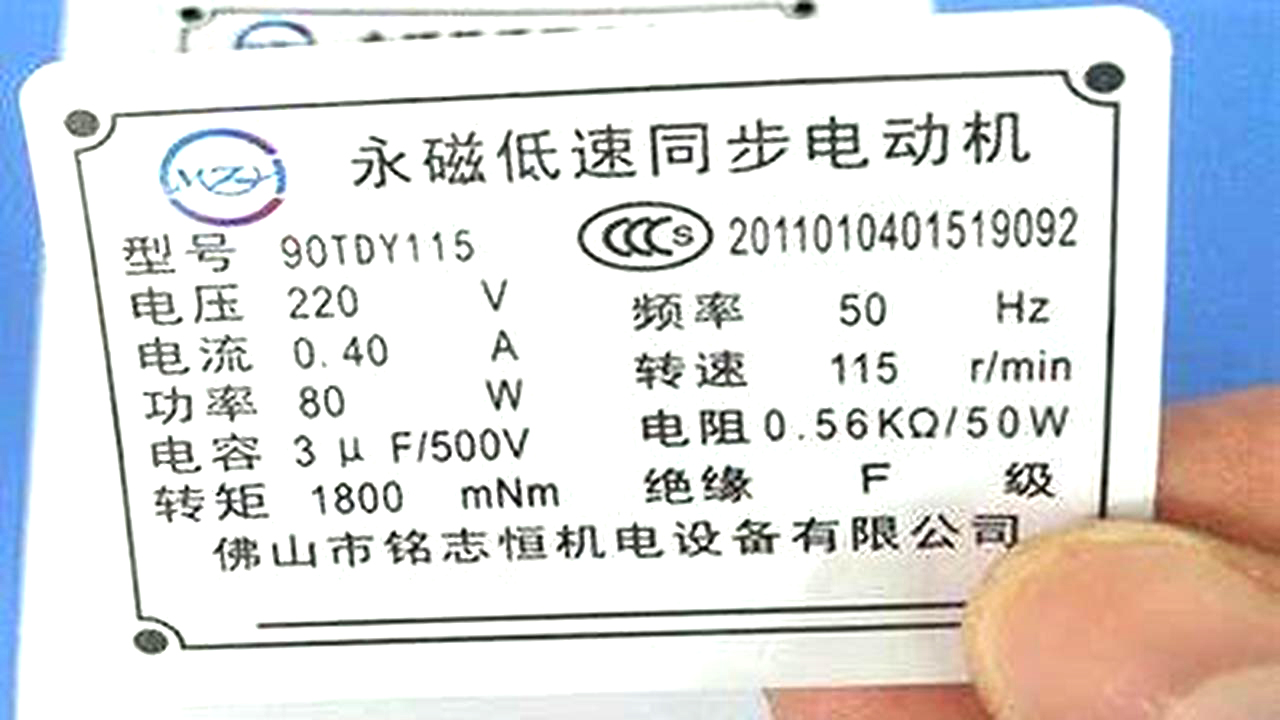

Supplement: S1 Dataset — (ZIP) [file pone.0300792.s001.zip › minimal data set/gt_img_0017_P1.0.jpg]

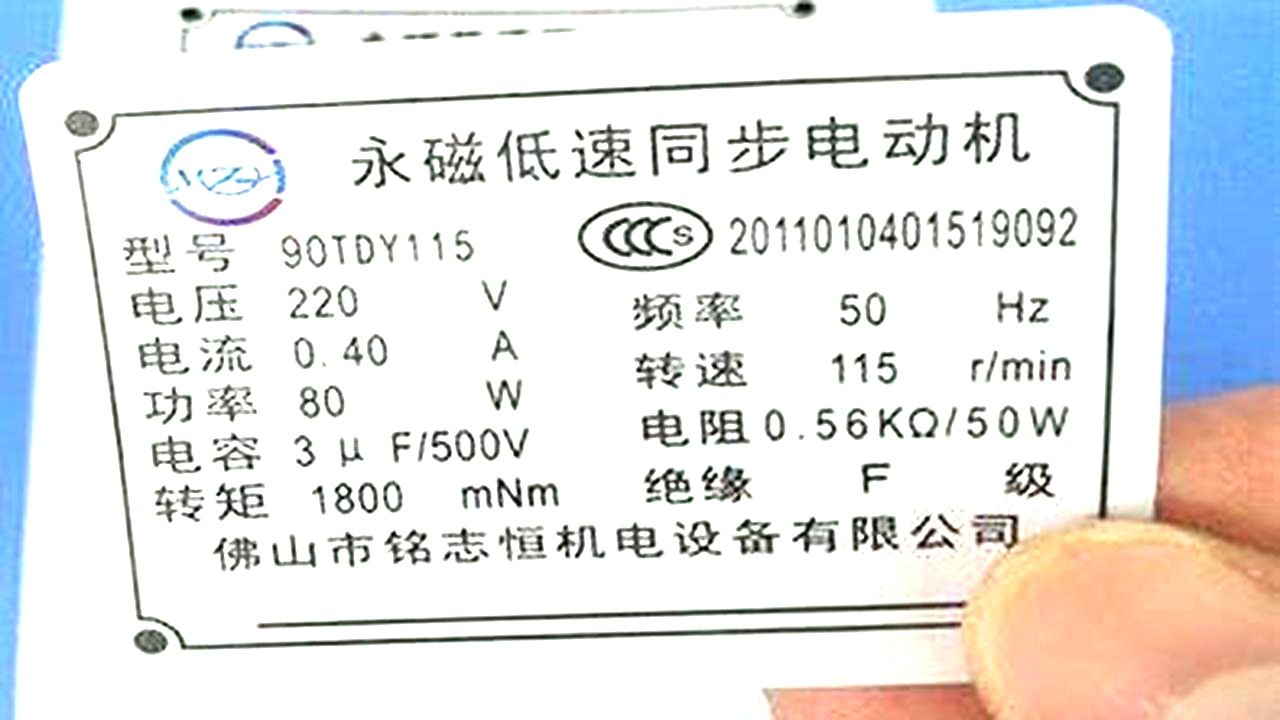

Supplement: S1 Dataset — (ZIP) [file pone.0300792.s001.zip › minimal data set/gt_img_0017_P1.5.jpg]

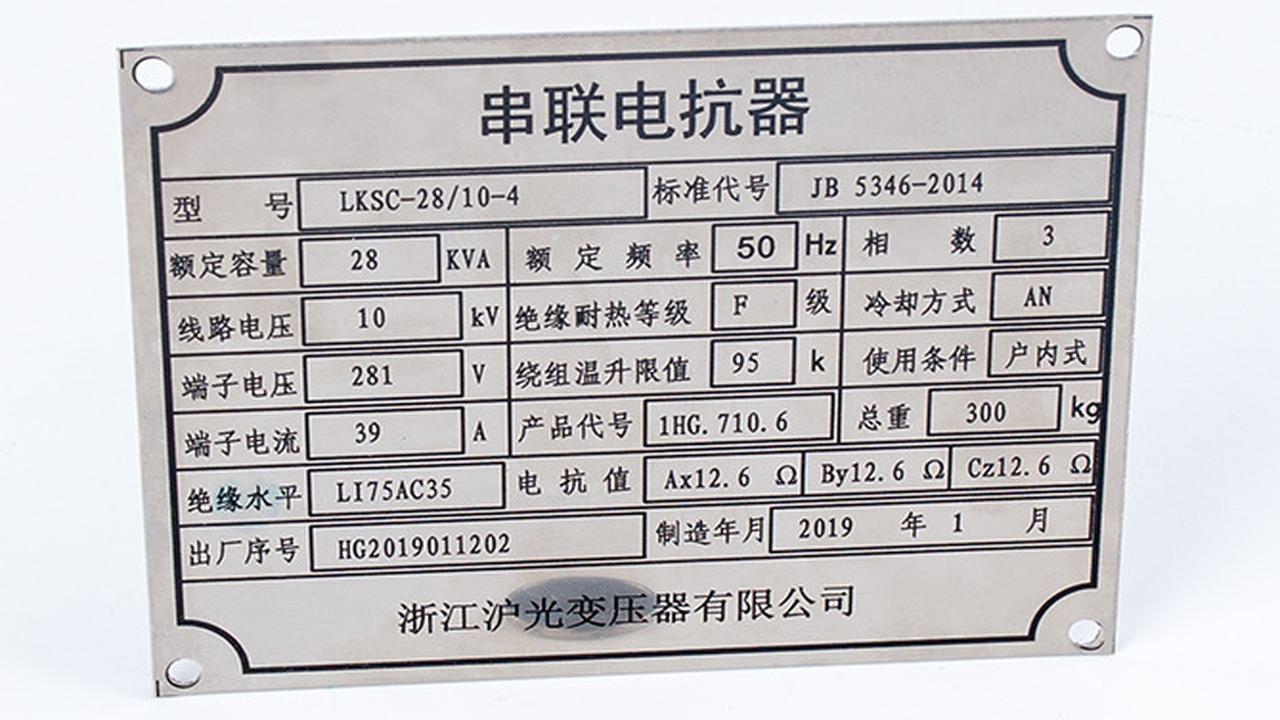

Supplement: S1 Dataset — (ZIP) [file pone.0300792.s001.zip › minimal data set/gt_img_0018_0.jpg]

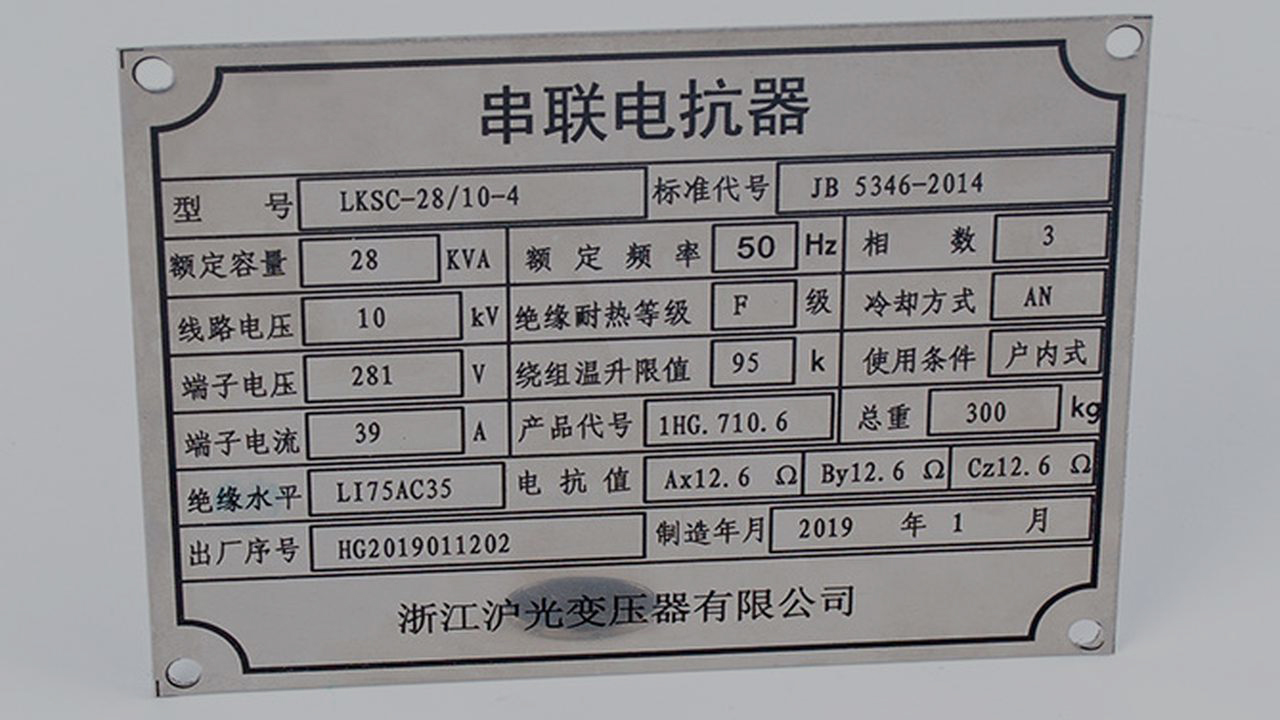

Supplement: S1 Dataset — (ZIP) [file pone.0300792.s001.zip › minimal data set/gt_img_0018_N1.0.jpg]

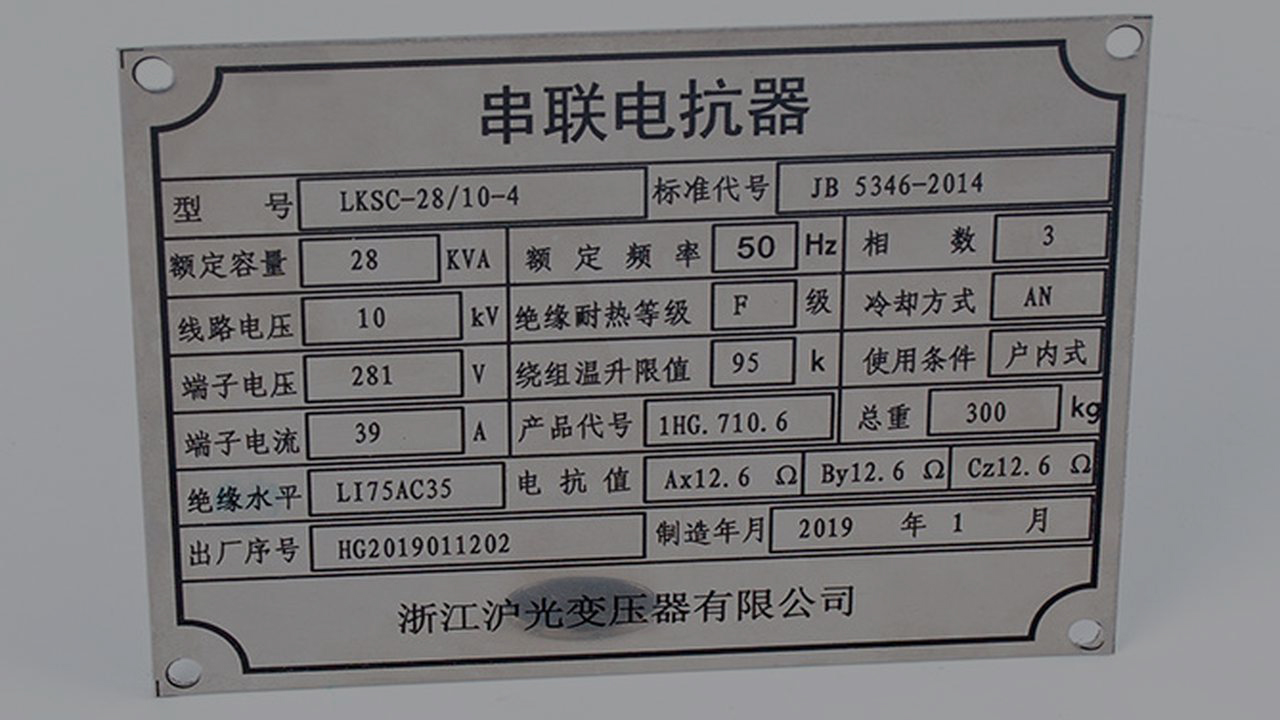

Supplement: S1 Dataset — (ZIP) [file pone.0300792.s001.zip › minimal data set/gt_img_0018_N1.5.jpg]

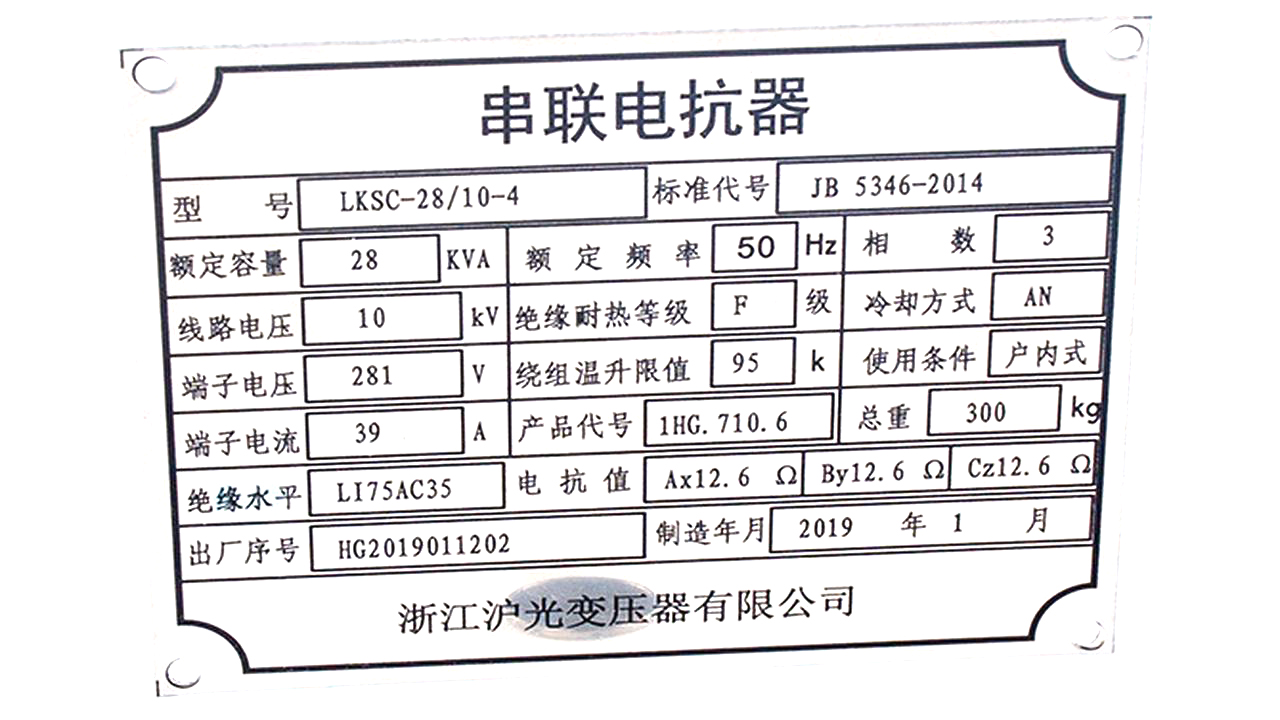

Supplement: S1 Dataset — (ZIP) [file pone.0300792.s001.zip › minimal data set/gt_img_0018_P1.0.jpg]

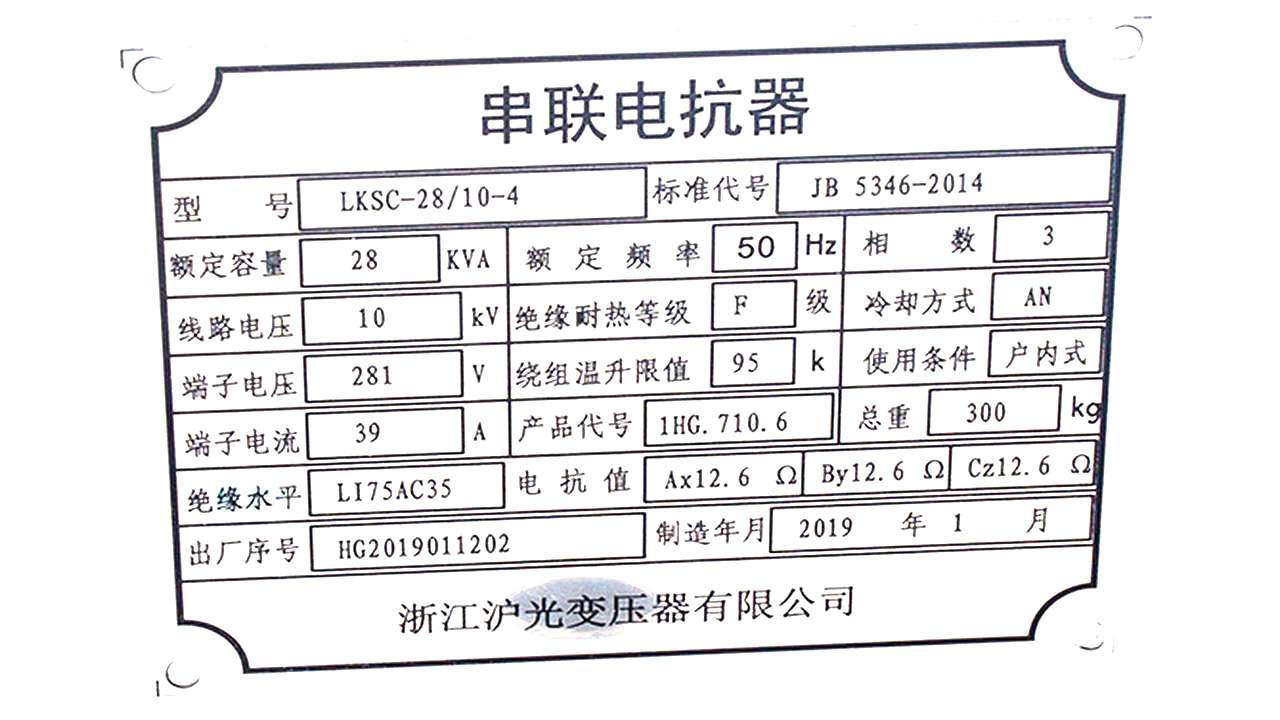

Supplement: S1 Dataset — (ZIP) [file pone.0300792.s001.zip › minimal data set/gt_img_0018_P1.5.jpg]

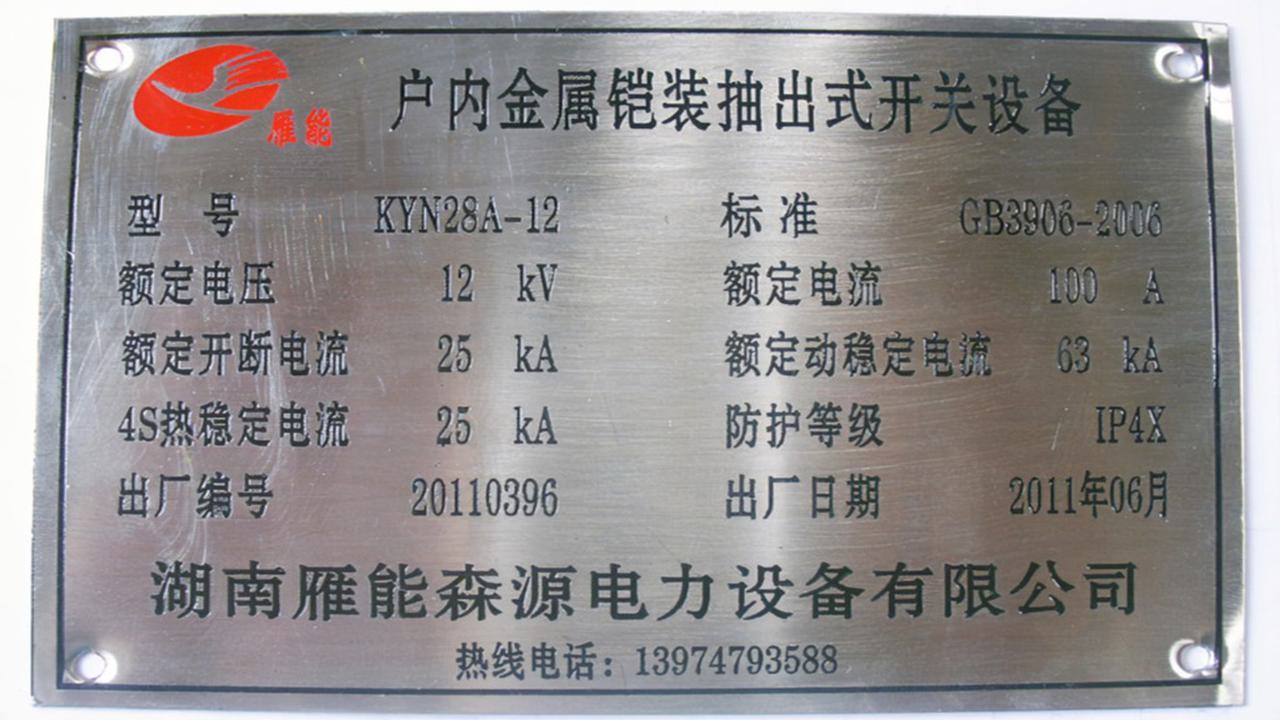

Supplement: S1 Dataset — (ZIP) [file pone.0300792.s001.zip › minimal data set/gt_img_0019_0.jpg]

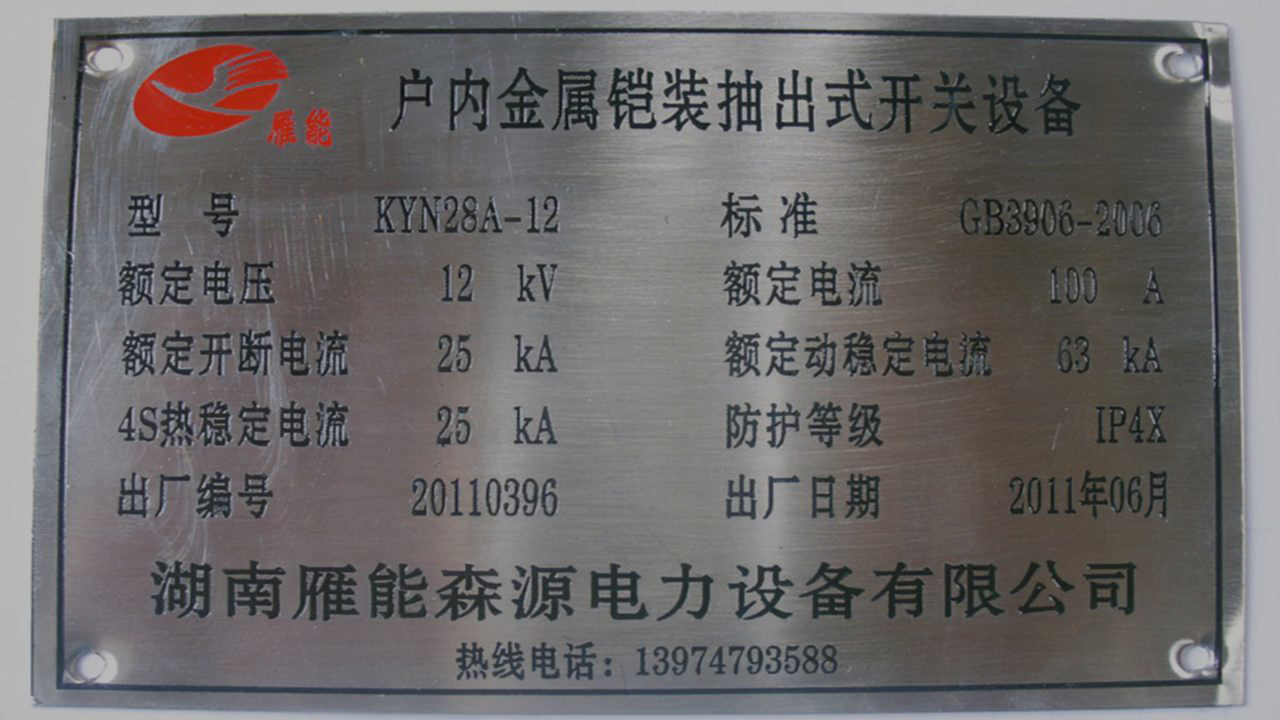

Supplement: S1 Dataset — (ZIP) [file pone.0300792.s001.zip › minimal data set/gt_img_0019_N1.0.jpg]

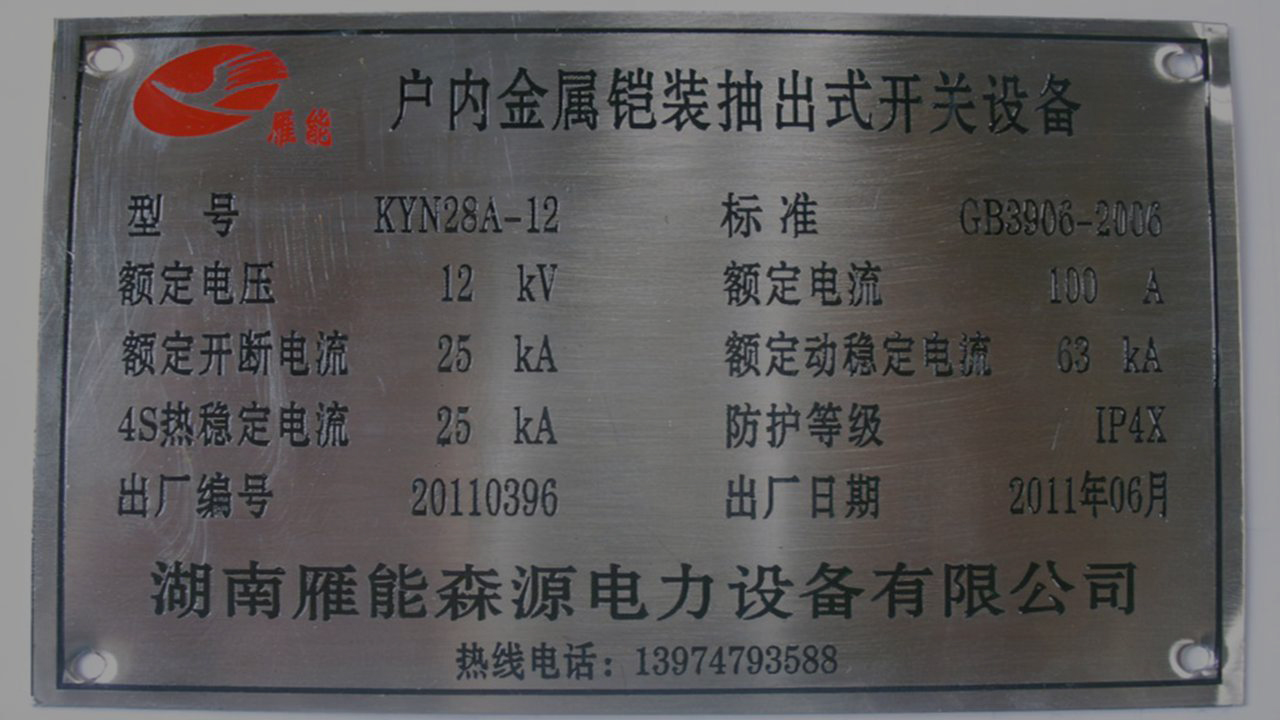

Supplement: S1 Dataset — (ZIP) [file pone.0300792.s001.zip › minimal data set/gt_img_0019_N1.5.jpg]

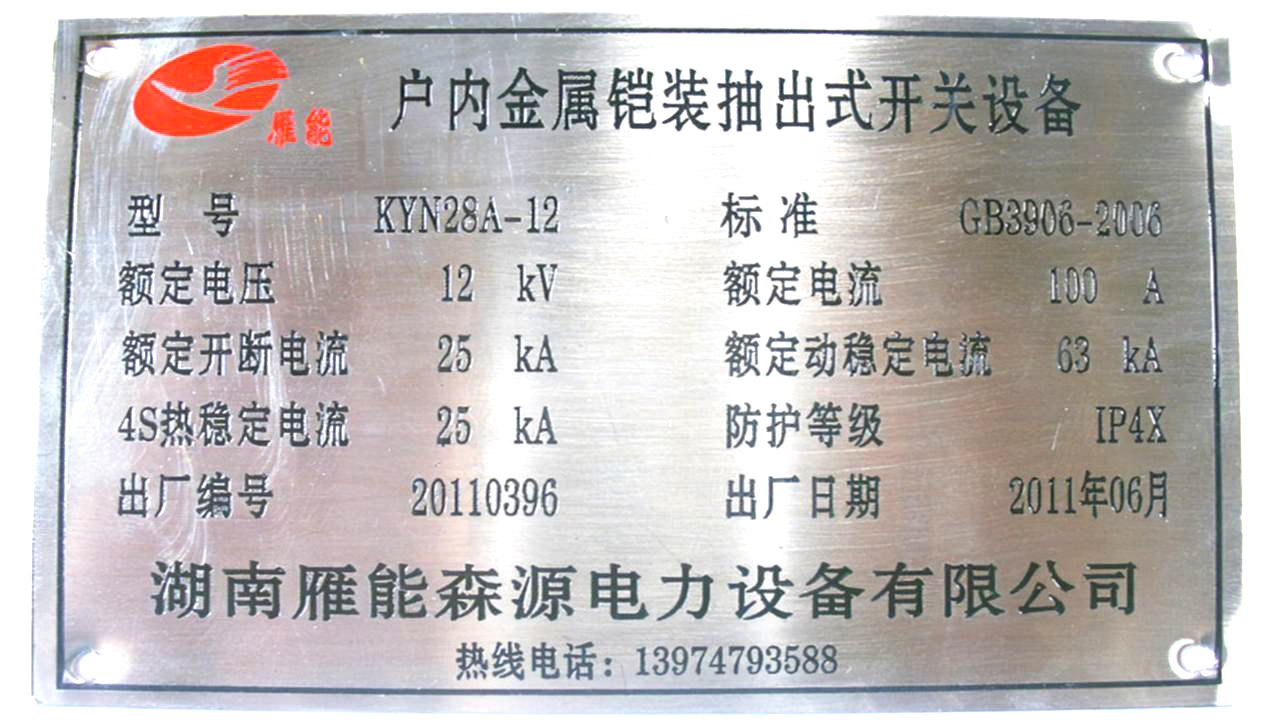

Supplement: S1 Dataset — (ZIP) [file pone.0300792.s001.zip › minimal data set/gt_img_0019_P1.0.jpg]

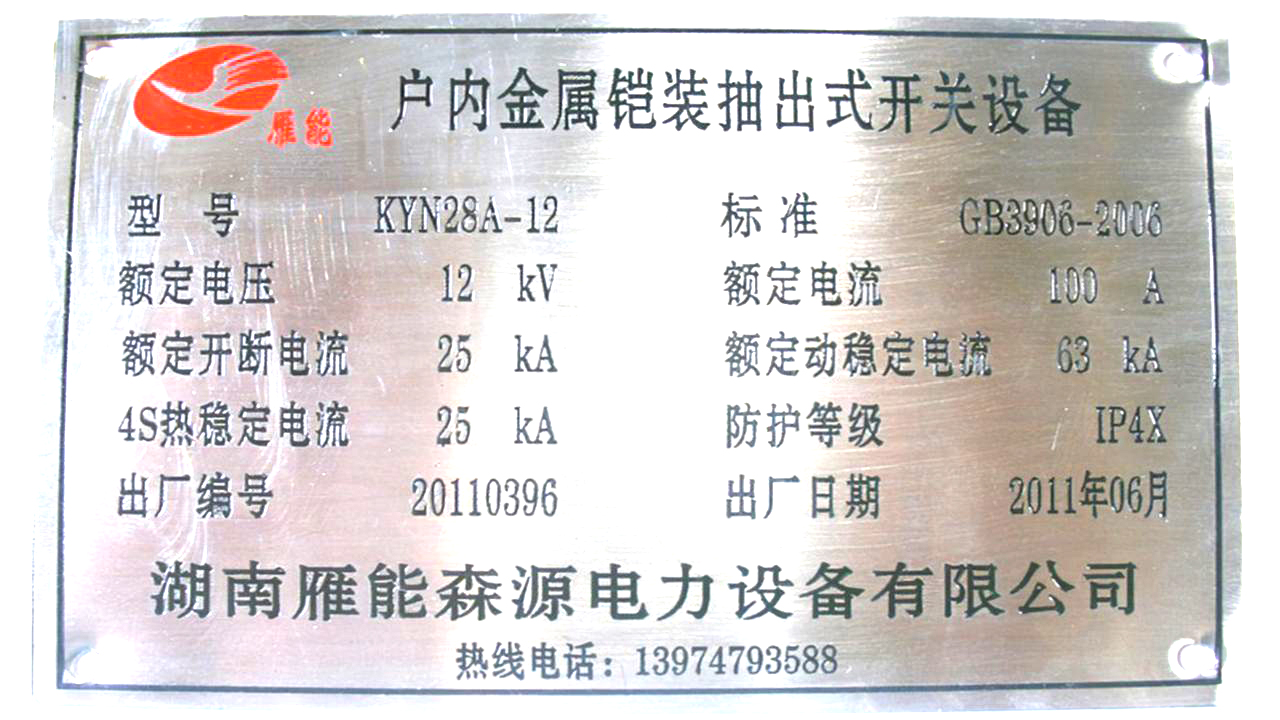

Supplement: S1 Dataset — (ZIP) [file pone.0300792.s001.zip › minimal data set/gt_img_0019_P1.5.jpg]

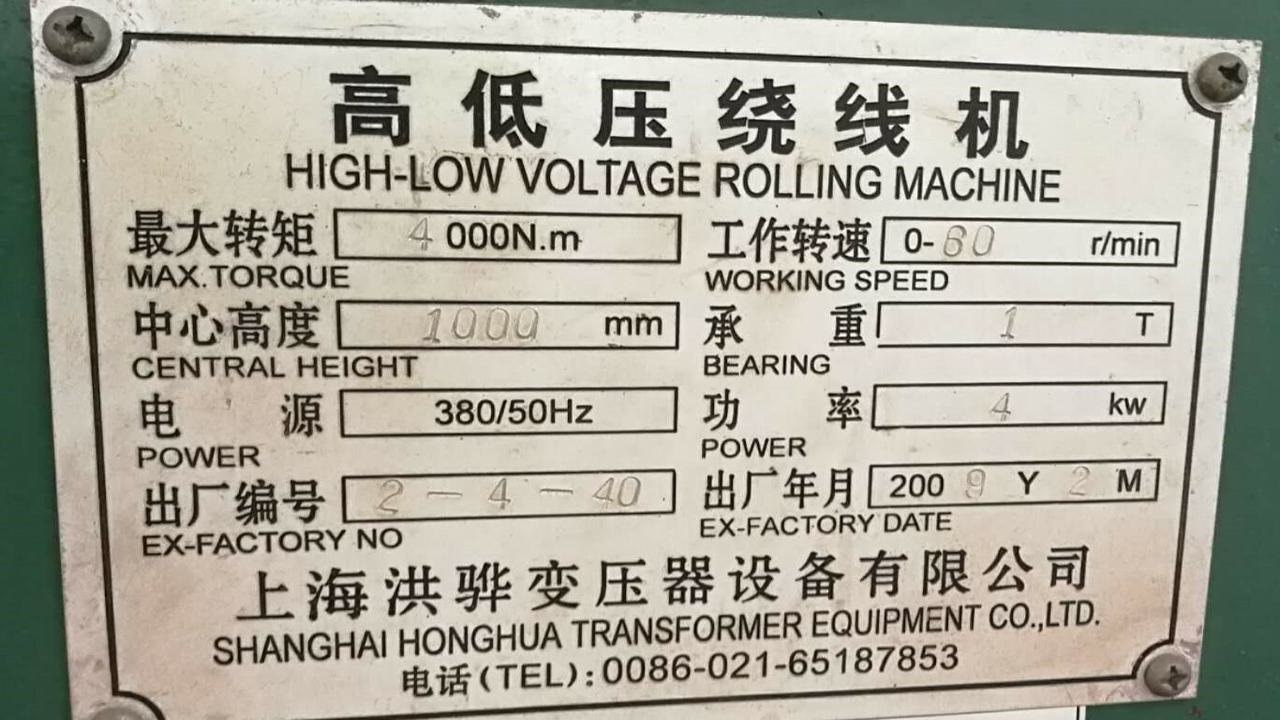

Supplement: S1 Dataset — (ZIP) [file pone.0300792.s001.zip › minimal data set/gt_img_0020_0.jpg]

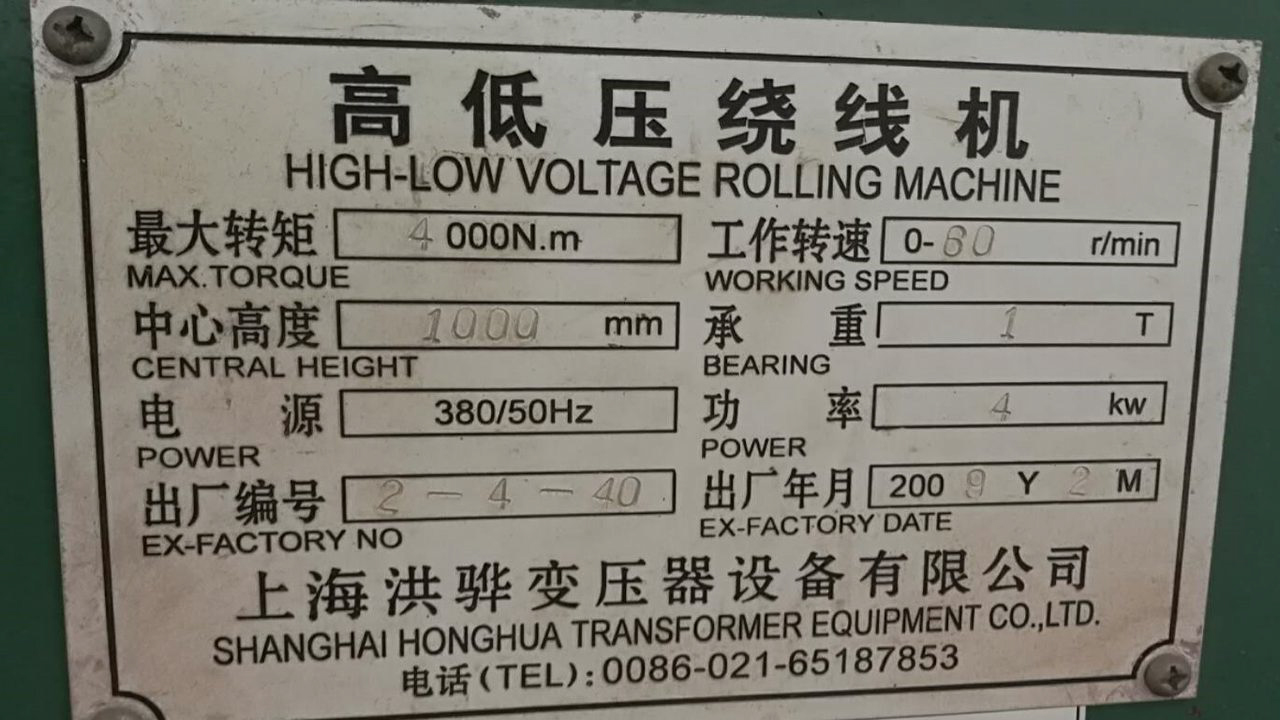

Supplement: S1 Dataset — (ZIP) [file pone.0300792.s001.zip › minimal data set/gt_img_0020_N1.0.jpg]

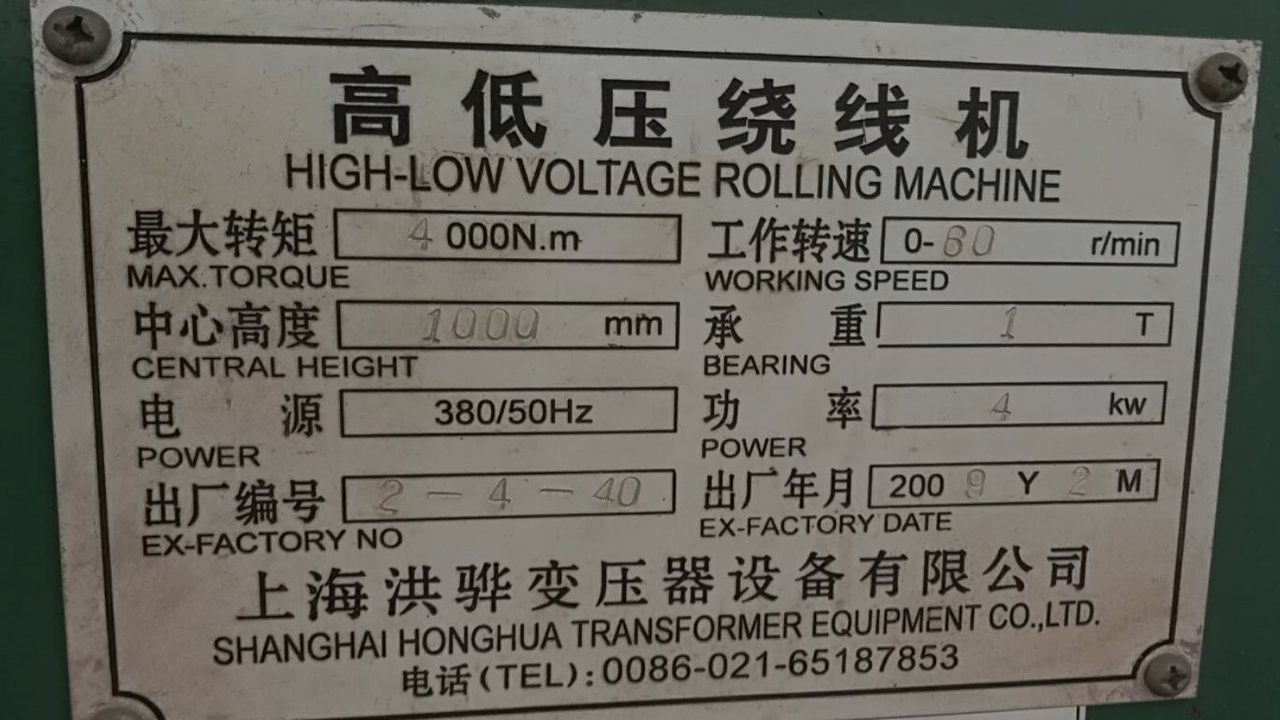

Supplement: S1 Dataset — (ZIP) [file pone.0300792.s001.zip › minimal data set/gt_img_0020_N1.5.jpg]

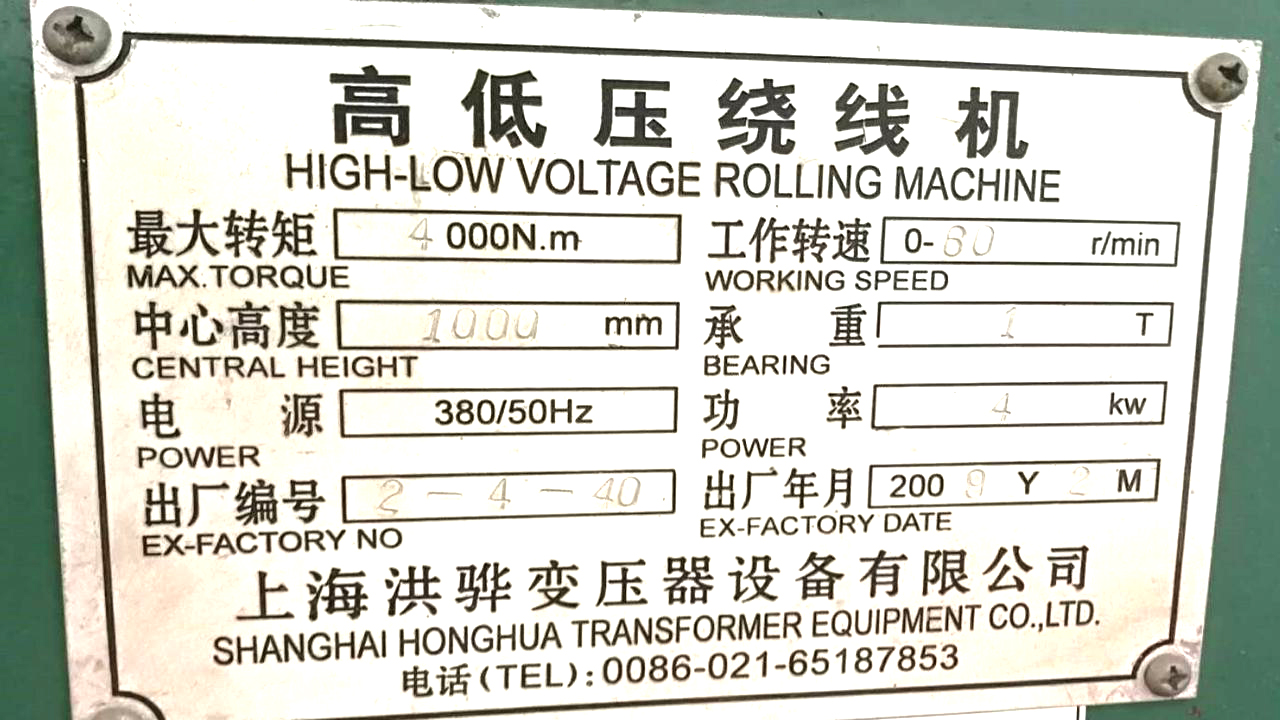

Supplement: S1 Dataset — (ZIP) [file pone.0300792.s001.zip › minimal data set/gt_img_0020_P1.0.jpg]

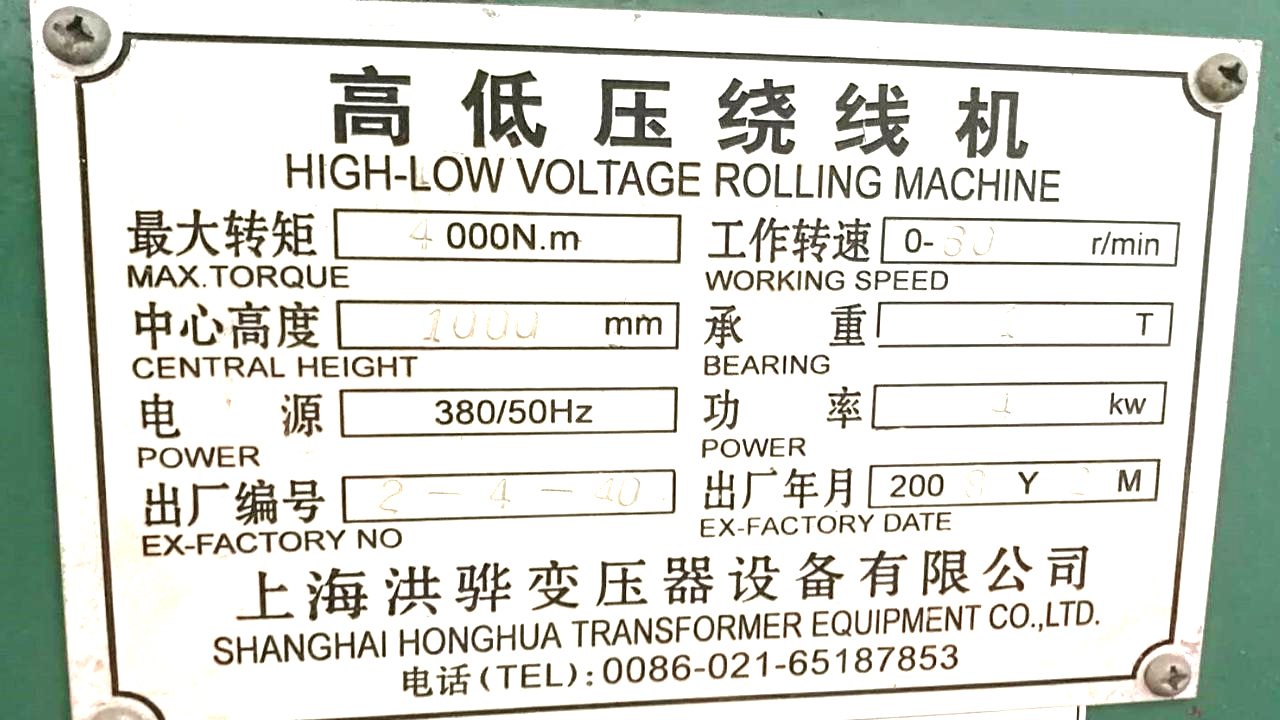

Supplement: S1 Dataset — (ZIP) [file pone.0300792.s001.zip › minimal data set/gt_img_0020_P1.5.jpg]
