# Supplementary material for: Exposure image correction of electrical equipment nameplate based on the LMPEC algorithm
Source: PLoS One. 2024 Jun 27;19(6):e0300792. doi: 10.1371/journal.pone.0300792 (PMC11210806; doi:10.1371/journal.pone.0300792)
Supplement: Attachment — Submitted filename: Response to Reviewers.docx [file pone.0300792.s002.docx]

Dear Editor and Reviewers:

Thank you for your letter and for the Review experts’ comments concerning our manuscript. Those comments are all valuable and very helpful for revising and improving our paper, as well as the important guiding significance to our research. We have studied comments carefully and have made corrections which we hope meet with approval. Revised portions are highlighted in yellow on the Revised Manuscript with Track Changes. The main corrections in the paper and responses to the reviewers' comments are as follows:

**Response to Academic Editor:**

1. Please ensure that your manuscript meets PLOS ONE's style requirements, including those for file naming.

All the documents I have submitted are in accordance with the requirements of PLOE ONE.

2. In your Methods section, please provide additional information regarding the permits you obtained for the work. Please ensure you have included the full name of the authority that approved the field site access and, if no permits were required, a brief statement explaining why.

I am a graduate student at Sichuan University of Science & Engineering, and my work in this paper has been approved by the Artificial Intelligence Laboratory of Sichuan University of Science & Engineering.

3. Please note that PLOS ONE has specific guidelines on code sharing for submissions in which author-generated code underpins the findings in the manuscript. In these cases, all author-generated code must be made available without restrictions upon publication of the work.

The complete code and version of the manuscript have been uploaded to the system together.

4. We note that the grant information you provided in the ‘Funding Information’ and ‘Financial Disclosure’ sections do not match.

I will ensure the correct funding number when resubmitting.

5. Please state what role the funders took in the study.

The funders had no role in study design, data collection and analysis, decision to publish, or preparation of the manuscript.

6. Please upload a copy of Figures 1, 2, 3, 4, 5, 6, 7, 8, 9 and 10 to which you refer in your text. If the figure is no longer to be included as part of the submission please remove all reference to it within the text.

I have re uploaded all the images in the correct format.

7. We note that Supplementary figures 1, 7, 8, 9 and 10 in your submission contain copyrighted images.

The images mentioned by the academic editor were all taken by me under the permission of the relevant factory, and the copyright of all the images in the paper belongs to me. So there is no copyright issue with the images I used in my paper.

8. Please include captions for your Supporting Information files at the end of your manuscript, and update any in-text citations to match accordingly.

All supporting information related to the article has been uploaded, and all references have been updated.

Based on the LMPEC Algorithm

**Response to expert reviewer comments：**

**Reviewer #1's comments：**

1. Starting with the second paragraph of each section, the first sentence should be indented by 2 characters.

The format of this paper strictly follows the writing requirements of PLOS ONE, and there is no requirement from PLOS ONE to indent the first sentence of each paragraph in the original manuscript.

2. In section 3.3, please explain the reason for selecting the 7 m width of the pit slope as the subject of the study, i.e., how the value of the slope width was determined.

There is no section 3.3 and research content on mine slope here, which may be an incorrect comment.

3. In Figure 8, the exposure parameters for each row of images should be indicated.

The revised version of the manuscript has added a description of the input image exposure parameters before Figure 8.

4. The image before correction should be added in Figure 9 and used to compare with the effect image after correction in order to analyze the correction effect in both underexposure and overexposure cases.

Figure 9 contains the image before correction, which is in the second row of Figure 9.

5. The content of Table 5 shows that the optimization algorithm proposed in this paper improves the evaluation indices, but the evaluation indices of this algorithm in the table do not differ much from the improved values of other algorithms, while the complexity and computational volume of this algorithm increases relatively, is it possible to assess whether this algorithmic improvement is meaningful in relation to the increase in complexity?

Compared to the algorithm before optimization, the model in this paper has a certain improvement in complexity and computational complexity. The model in this paper is mainly composed of several UNet++ networks. Compared with most other network models, the UNet++ network has a simple structure and low computational complexity. Therefore, even though the improved model has increased computational complexity compared to the original model, overall, all the complexity and computational complexity of the model are still relatively small, and the model can still be easily trained. It is worthwhile to exchange a slight increase in complexity for an average performance improvement of about 6%.

**Reviewer #2's comments：**

1. The writing should be improved to add the readability.

The revised version of the manuscript has adjusted some sentences in the paper to increase readability.

2. The motivation of this paper should be summarized in the introduction clearly.

The revised version of the manuscript has added explanatory sentences on writing motivation in the introduction.

3. The contributions of this paper should be summarized in the introduction clearly.

The revised version of the manuscript has an introduction section that has added the contribution of this paper.

4. Explain the rationality of the chosen data sets.

The revised version of the manuscript has added paragraphs on the sources and processing methods of the dataset to demonstrate its rationality.

**Reviewer #3's comments：**

1. There are duplicates in the name of the paper.

The revised version of the manuscript has removed "optimization" from the title, and the revised title is Exposure Image Correction of Electrical Equipment Nameplate Based on LMPEC Algorithm.

2. In the introduction, the core problem of image recognition is not clearly explained. It is difficult to judge the theoretical contribution of this paper.

The revised version of the manuscript has an introduction section that has added the contribution of this paper.

3. There is some unreason ability in the structure of the paper, such as separating the pictures and words of the paper, which brings some difficulties to the reading.

The format of this paper strictly follows the writing requirements of PLOS ONE, and PLOS ONE requires that papers and images be submitted separately.

4. The data source of the images adopted in the paper is not clear enough. At the same time the paper lacks the analysis of the recognition image. The paper uses some methods, but these methods from the point of view of solving the problem, whether necessary. It is suggested that the author should make further explanation.

For the question with datasets, the revised version of the manuscript has added paragraphs on the sources and processing methods of the dataset to demonstrate its rationality.

For the question of the necessity of this study, the main research content of this paper is the exposure correction of electrical equipment nameplates. However, in addition to this study, the author's research team has also conducted relevant research on detecting and recognizing text information on electrical equipment nameplates. We found that when there is insufficient or overexposure in the image of the electrical equipment nameplate, it can have a certain impact on detecting nameplate text. As shown in the figure below, our team uses an improved algorithm based on the EAST algorithm to perform text detection on images with exposure parameters of -1.5, 0,and +1.5, respectively. From the figure, the nameplate image under standard exposure has fewer missed and false detections compared to under exposure and over exposure images. Therefore, we conducted related research on exposure image correction of electrical equipment nameplates. In addition, the revised version of the manuscript has added relevant explanations on the necessity of this study in the introduction section.

**Figure1 Nameplate image with an exposure parameter of -1.5**

**Figure2 Nameplate image with an exposure parameter of 0**

**Figure3 Nameplate image with an exposure parameter of +1.5**
